# Identification of a New, Potent, and Stable Tetrahydroquinoline-Based 5‑LOX/sEH Dual Inhibitor with Polymodal Anti-Inflammatory Activity in Acute Pancreatitis

**DOI:** 10.1021/acs.jmedchem.6c01458

**Published:** 2026-07-27

**Authors:** Danilo D’Avino, Tania Ciaglia, Paul M. Jordan, Simone Di Micco, Simona Musella, Veronica Di Sarno, Fabrizio Merciai, Sara Perna, Gerardina Smaldone, Francesca Di Matteo, Valeria Napolitano, Rosario Stimoli, Ignazio Sardo, Ornella Ricci, Giuseppe Bifulco, Eduardo Maria Sommella, Manuela Giovanna Basilicata, Giovanni Ferrara, Fiorentina Roviezzo, Anna Di Dio, Giovanna Aquino, Sofia Pellecchia, Giacomo Pepe, Isabel M. Gomez-Monterrey, Pietro Campiglia, Carmine Ostacolo, Michele Manfra, Oliver Werz, Antonietta Rossi, Alessia Bertamino

**Affiliations:** † Department of Pharmacy, School of Medicine and Surgery, 9307 University of Naples Federico II, Via D. Montesano 49, 80131 Naples, Italy; ‡ Department of Pharmacy, University of Salerno, Via G. Paolo II 132, 84084 Fisciano, Salerno Italy; § Department of Pharmaceutical/Medicinal Chemistry, Institute of Pharmacy, Friedrich Schiller University, Philosophenweg 14, D-07743 Jena, Germany; ∥ European Biomedical Research Institute of Salerno (EBRIS), Via S. De Renzi 50, 84125 Salerno, Italy; ⊥ National PhD Program in ″RNA Therapeutics and Gene Therapy″, 80131 Naples, Italy; # Department of Advanced Medical and Surgical Sciences,University of Campania “Luigi Vanvitelli”, P.zza L. Miraglia, 2, 80138 Naples, Italy; ∇ LUNAVET LAB S.a.s., Via Giovanni Andrea Aurofino, 20, 84127 Salerno, Italy; ○ AREA Science Park, Laboratorio di Multi-omica Area Sud (LAAS), 84081 Baronissi, Salerno Italy; ◆ PhD Program in Drug Discovery and Development, Department of Pharmacy, University of Salerno, Via G. Paolo II 132, 84084 Fisciano, Salerno, Italy; ¶ Department of Health Sciences, University of Basilicata, Via dell’Ateneo Lucano 10, 85100 Potenza, Italy

## Abstract

Acute pancreatitis (AP) is a common, serious, pancreatic inflammation representing an unmet therapeutical need. Here, we report the design and synthesis of a series of compounds as dual 5-lipoxygenase (LOX)/soluble epoxide hydrolase (sEH) inhibitors aimed at favorably modulating inflammatory lipid mediator pathways. The tetrahydroquinoline-based compound **11** emerged as the most promising candidate, showing potent *in vitro* inhibition of both enzymes and satisfactory metabolic stability. Compound **11** was evaluated in a cerulein-induced murine model of AP, where it significantly reduced pancreatic damage, cellular infiltration, and edema formation. These effects led to beneficial modulation of LMs and attenuation of ferroptosis, concomitant with activation of the Nrf2 antioxidant pathway and upregulation of cytoprotective markers. Compound **11** ameliorated lung, liver, and kidney secondary damage, displaying an improved *in vivo* pharmacokinetic profile. Overall, these findings reveal compound **11** as a promising anti-inflammatory agent supporting dual 5-LOX/sEH inhibition, coupled with Nrf2 activation, as a viable strategy for AP.

## Graphical abstract

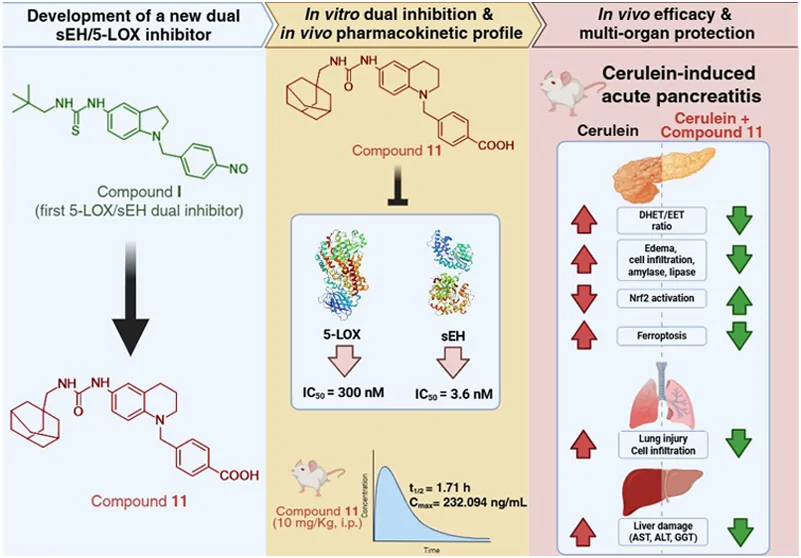

## Introduction

Acute pancreatitis (AP), a severe pancreas inflammation, is among the most common inflammatory diseases involving the gastrointestinal tract with an incidence of 5 to 80 per 100,000 population worldwide and a growing forecast.^1,2^ Although most cases are mild and self-limiting, severe AP is characterized by sudden onset, rapid progression to pancreatic necrosis, and the development of multiorgan dysfunction, with mortality rates reaching 20–30% and a significant socio-economic burden. The first report about this disorder dates back to 1579 and since then symptoms and causes behind AP have been deeply characterized, but its diagnosis, staging and treatment are still limited.^3^ Indeed, AP treatment is based on supportive care and consists of the combination of several interventions including fluid resuscitation, nutritional support, pain management, antibiotic treatment to fight infections at necrotic tissues, and anti-inflammatory therapy.^4^ Specifically, the use of nonsteroidal anti-inflammatory drugs has been proven to ameliorate the clinical outcome of AP, especially cyclooxygenase (COX)-2 selective inhibitors, however they are plagued by serious unwanted side effects at the cardiovascular level.^5^ AP is characterized by the premature release of digestive enzymes into the pancreatic interstitium and the systemic circulation, which triggers a cascade of local and systemic inflammatory responses, resulting in extensive tissue damage and systemic complications.^6^ Symptoms start with epigastric pain, nausea, vomiting, and increased levels of serum amylase or lipase that are considered useful diagnostic biomarkers in early or late stage of AP.^2,7^ At the molecular level, AP is associated with severe oxidative stress driven by excessive reactive oxygen species (ROS) production and weakened antioxidant defenses, which not only exacerbate pancreatic acinar cell injury and inflammatory signaling, but also promote ferroptosis, a regulated form of cell death characterized by iron-dependent lipid peroxidation.^8^ The local recruitment and activation of inflammatory cells (e.g., neutrophils and macrophages) further exacerbate tissue damage through the release of pro-inflammatory mediators, such as arachidonic acid (AA) metabolites. AA can be metabolized by three pathways, catalyzed by different enzymes, namely COXs, 5-lipoxygenases (5-LOX) and cytochrome P450 (CYP450). COXs are responsible for prostaglandin (PG) and thromboxane A_2_ production, 5-LOX catalyzes the formation of leukotrienes (LTs), while CYP450 is implicated in epoxyeicosatrienoic acid (EET) biosynthesis.^9^ These lipid mediators (LMs) play important roles in homeostasis, but also in promoting inflammation.^10^ In particular, the pivotal involvement of 5-LOX in the pathogenesis of AP has been widely reported, and inhibition of this enzyme has been shown to alleviate pancreatic tissue damage.^11^ Notably, 5-LOX inhibition promotes apoptosis and suppresses neutrophil activation in pancreatic tissue during AP, indicating that 5-LOX inhibitors may represent promising therapeutic agents for the treatment of AP.^11,12^ Similarly, the EET pathway that supports the natural resolution of inflammatory conditions, is considered a valuable target to treat phlogistic pathologies. Indeed, EETs function as key signaling molecules in biological systems, contributing to vasodilation, attenuation of inflammation, and reduction of pain.^13^ However, they are rapidly metabolized by soluble epoxide hydrolase (sEH) into dihydroxyeicosatrienoic acids (DHETs), which are known to exert pro-inflammatory effects.^14^ Previous research has demonstrated that pharmacological inhibition of sEH markedly limits the conversion of EETs into DHETs, leading to a reduction in inflammatory responses and pain.^15^ Considering the crucial involvement of LMs in AP, together with the necessity to expand the therapeutic options in this serious pathology, here we describe the development of a series of dual 5-LOX/sEH inhibitors and the identification of a lead compound that was characterized for its activity in a murine model of cerulein (CER)-induced AP.

We have been engaged in the discovery of new anti-inflammatory agents for many years and we have developed substantial expertise in identifying and discriminating the molecular determinants that selectively direct compounds toward key enzymes involved in the AA cascades. In particular, we studied the importance of the indoline nucleus and its specific decorations to simultaneously inhibit 5-LOX and sEH, identifying compound **I** (Figure 1) as potent anti-inflammatory agent, validated in zymosan-induced peritonitis and experimental asthma models, but in the meanwhile plagued by a poor pharmacokinetic profile.^16^ Afterward, we showed that the shift from an indoline to an indole core allowed to selectively direct the inhibitory activity against sEH, with compound **II** (Figure 1), the most promising among the series, characterized in CER-induced AP mouse model.^17^ Finally, in our recent work we were able to highlight the structural elements required to inhibit 5-LOX-activating protein (FLAP), describing one of the few identified FLAP/sEH dual inhibitors (compound **III**, Figure 1), profiled by *in vivo* lipidomic analysis.^18^ Here, we present the discovery of compound **11**, an adamantyl-tetrahydroquinoline derivative (Figure 1) as a dual 5-LOX/sEH inhibitor along with *in vitro* and *in vivo* biological and pharmacokinetic characterization.

**Figure 1:**
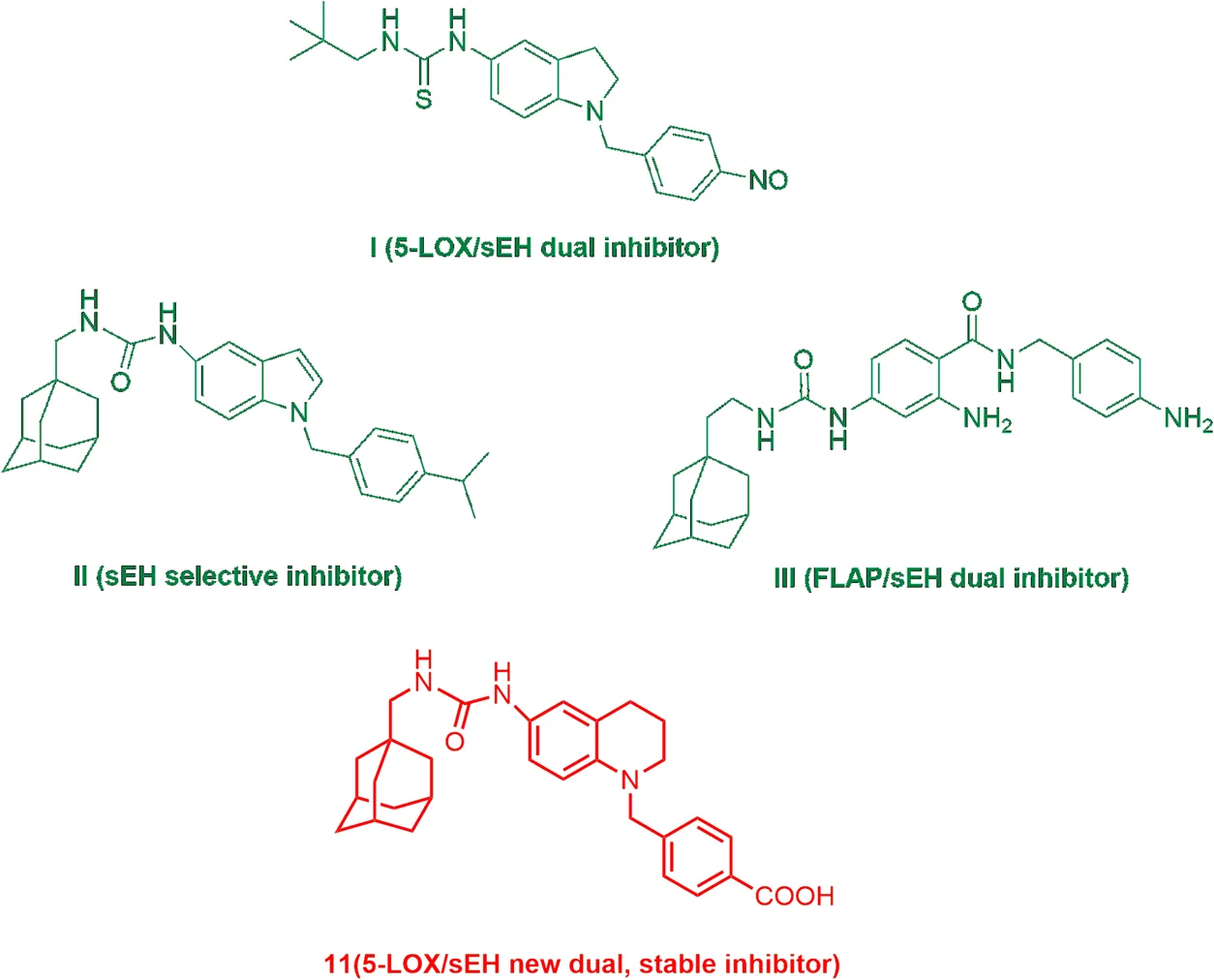
Chemical structures of our previously synthesized 5-LOX/sEH dual inhibitor, sEH selective inhibitor and FLAP/sEH dual inhibitor (green) and the new 5-LOX/sEH dual inhibitor (red).

We pursued three main objectives: (i) to highlight the enhanced potential of a dual 5-LOX/sEH inhibition in the CER-induced AP model; (ii) to evaluate pharmacokinetic suitability, improving the stability of the new lead compound with respect to our previous hit, derivative **I**; (iii) to explore both 5-LOX and sEH enzymatic pockets to improve knowledge helpful to trace specific pharmacophoric models. With respect to the latter aspect, we investigated different scaffolds, as well as various side chains, retaining the unmodified adamantyl-ureido function, which had already been proven to maximize compound/sEH inhibitory interactions (Figure 2).

**Figure 2:**
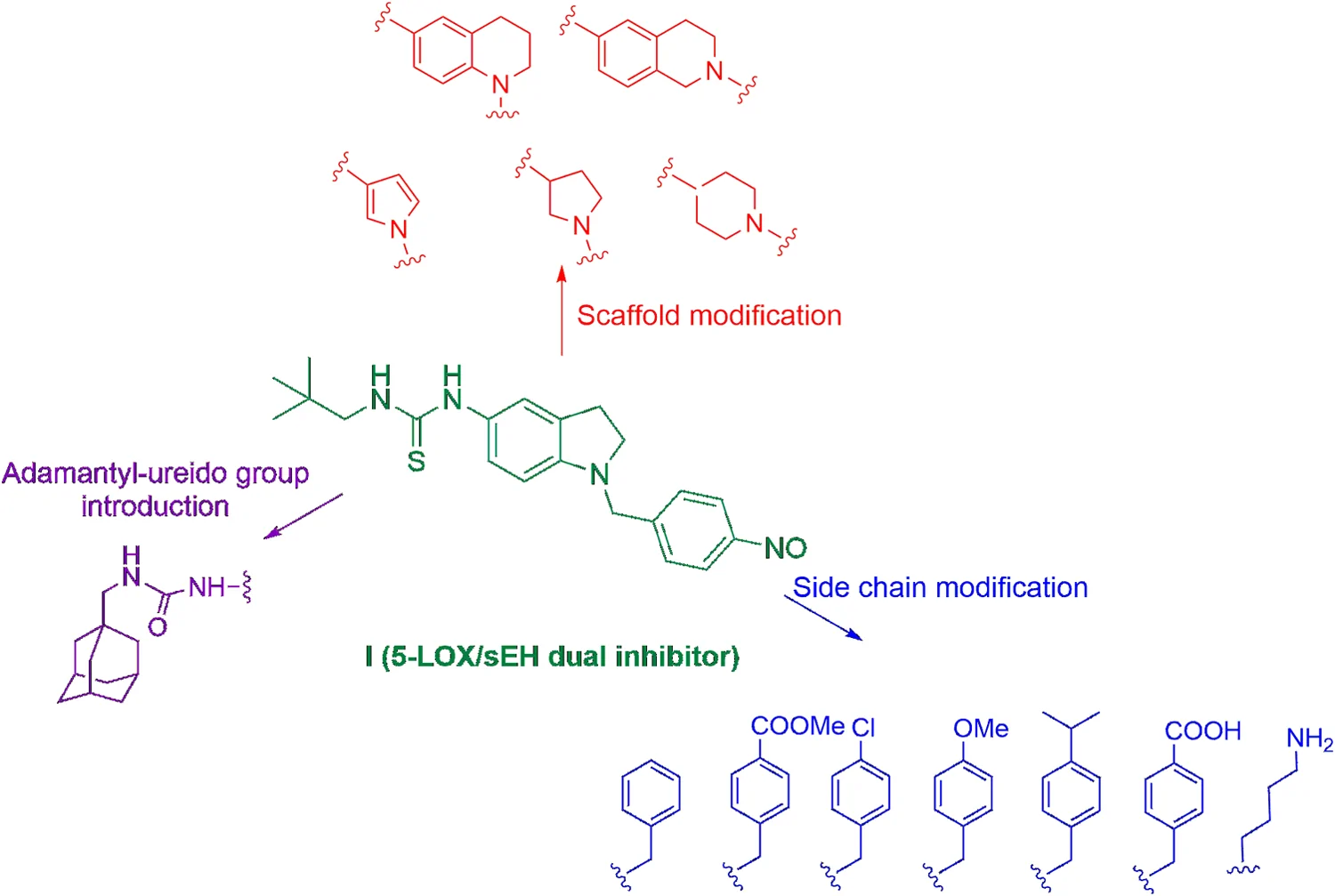
Chemical modifications on compound **I** leading to the new series of 5-LOX/sEH dual inhibitors.

From this investigation, compound **11** emerged as the most potent dual 5-LOX/sEH inhibitor, deserving deeper pharmacological characterization which was carried out by means of the CER-induced AP murine model. Histological analysis, LM quantification, and biomarker analysis highlight the remarkable potency of compound **11**. Moreover, the protective effect against ferroptosis injury and an improved pharmacokinetic profile demonstrate the success of the present medicinal chemistry campaign.

## Results and Discussion

### Chemistry

Tetrahydroquinoline derivatives **5–9**, **11**, and **12** were synthesized according to Scheme 1. 6-Nitro-1,2,3,4-tetrahydroquinoline was first protected at the amino group under microwave irradiation using di-*tert*-butyl dicarbonate in basic medium, furnishing the corresponding carbamate **1** (75% yield). The following reduction of the nitro group in the presence of ammonium formate and Pd/C as catalyst gave the aminic intermediate **2** in almost quantitative yield. The subsequent reaction with 1-adamantanemethylamine using triethylamine as base and triphosgene as carbonyl group donor led to the ureidic intermediate **3** (55% yield), which was deprotected at N-1 using trifluoroacetic acid in dichloromethane, giving **4** in almost quantitative yield. The latter was subjected to microwave-assisted alkylation reaction using benzyl bromide, methyl 4-(bromomethyl)­benzoate, 4-chlorobenzyl bromide, 4-methoxybenzyl bromide, and 4-isopropylbenzyl bromide under basic conditions, furnishing, respectively, the final derivatives **5–9** (40–48% yield). Furthermore, **4** was reacted with methyl 4-(chlorocarbonyl)­benzoate in alkaline medium, yielding intermediate **10** with 50% of yield. Basic hydrolysis of **6** and **10** furnished, quantitatively, the corresponding carboxylic acid **11** and **12**.

**Scheme 1:**
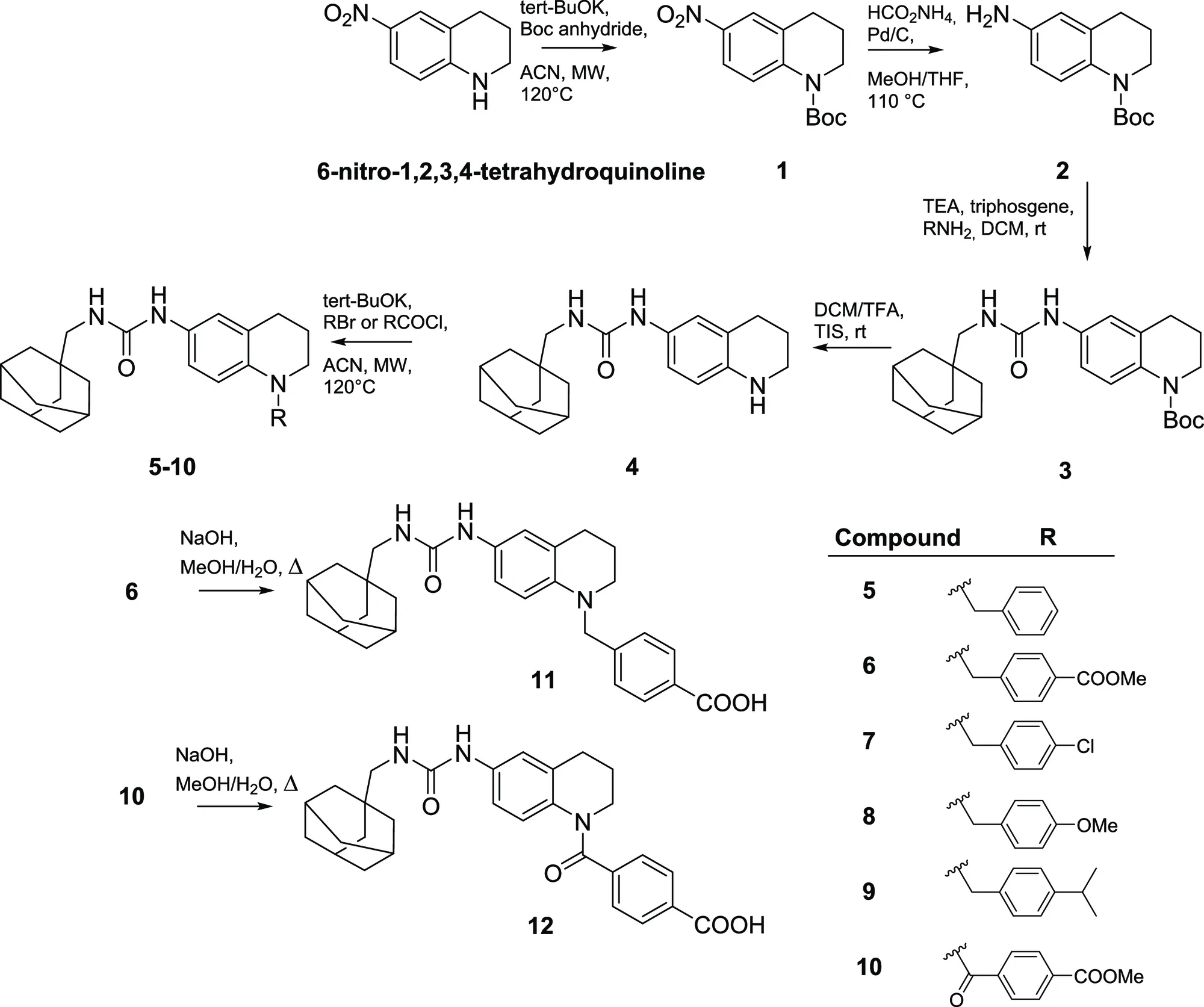
Synthesis of the Derivatives **5–9**, **11**, and **12**

The synthesis of final compounds **20–22** was performed as depicted in Scheme 2. Commercially available 3-nitropyrrole was derivatized at position N-1 by a microwave-assisted reaction with methyl 4-(bromomethyl)­benzoate and 4-(Boc-amino)­butyl bromide, leading to intermediates **13** and **14** (45 and 38% yield), respectively. The following reduction reaction in the previously mentioned Pd-catalyzed conditions gave the corresponding amino intermediates **15** and **16**, which were converted into ureas **17–19**, by reaction with cyclohexanemethylamine and 1-adamantanemethylamine (32–38% yield). Then, **17** and **18** were subjected to alkaline hydrolysis, generating, respectively, the final derivatives **20** and **21**, while **19** underwent Boc removal in an acidic medium leading, almost quantitatively, to the amino-free compound **22**.

**Scheme 2:**
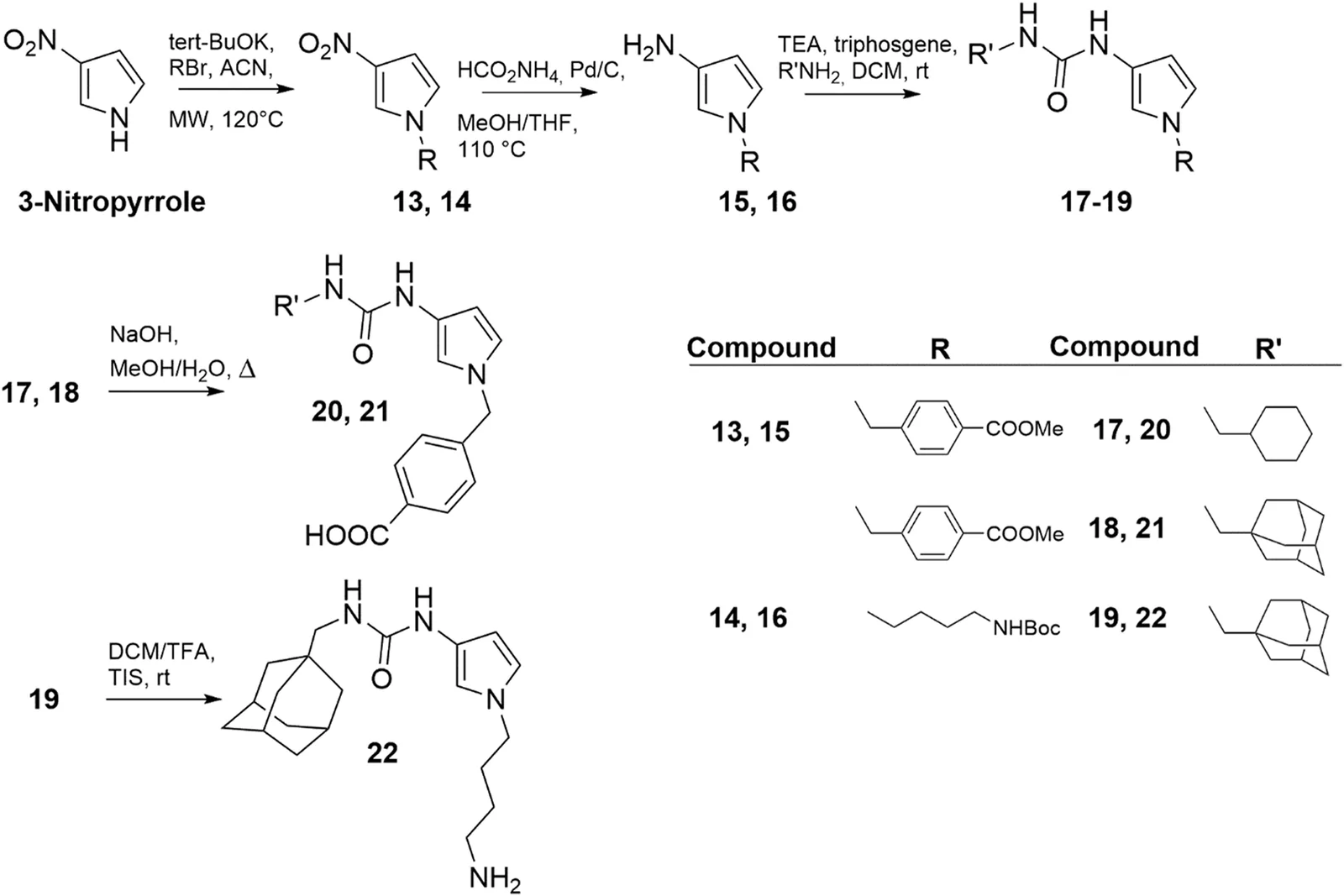
Synthesis of the Derivatives **20–22**

Derivatives **28** and **29** were synthesized as reported in Scheme 3. 6-Nitro-1,2,3,4-tetrahydroisoquinoline reacted with di-*tert*-butyl dicarbonate under the conditions described above leading to the protected intermediate **23** (83% yield), which was reduced at the nitro group and coupled with 1-adamantanemethylamine, furnishing the urea **25** (48% yield). Then, it was subjected to deprotection at N-1 under acidic conditions and further alkylation reaction using, respectively, methyl 4-(bromomethyl)­benzoate and 4-chlorobenzyl bromide, yielding intermediate **27** in 47% yield and final compound **28** in 50% yield. The following basic hydrolysis of **27** led to 4-((6-(3-(adamantan-1-ylmethyl)­ureido)-3,4-dihydroisoquinolin-2­(1*H*)-yl)­methyl)­benzoic acid (**29**) almost quantitatively.

**Scheme 3:**
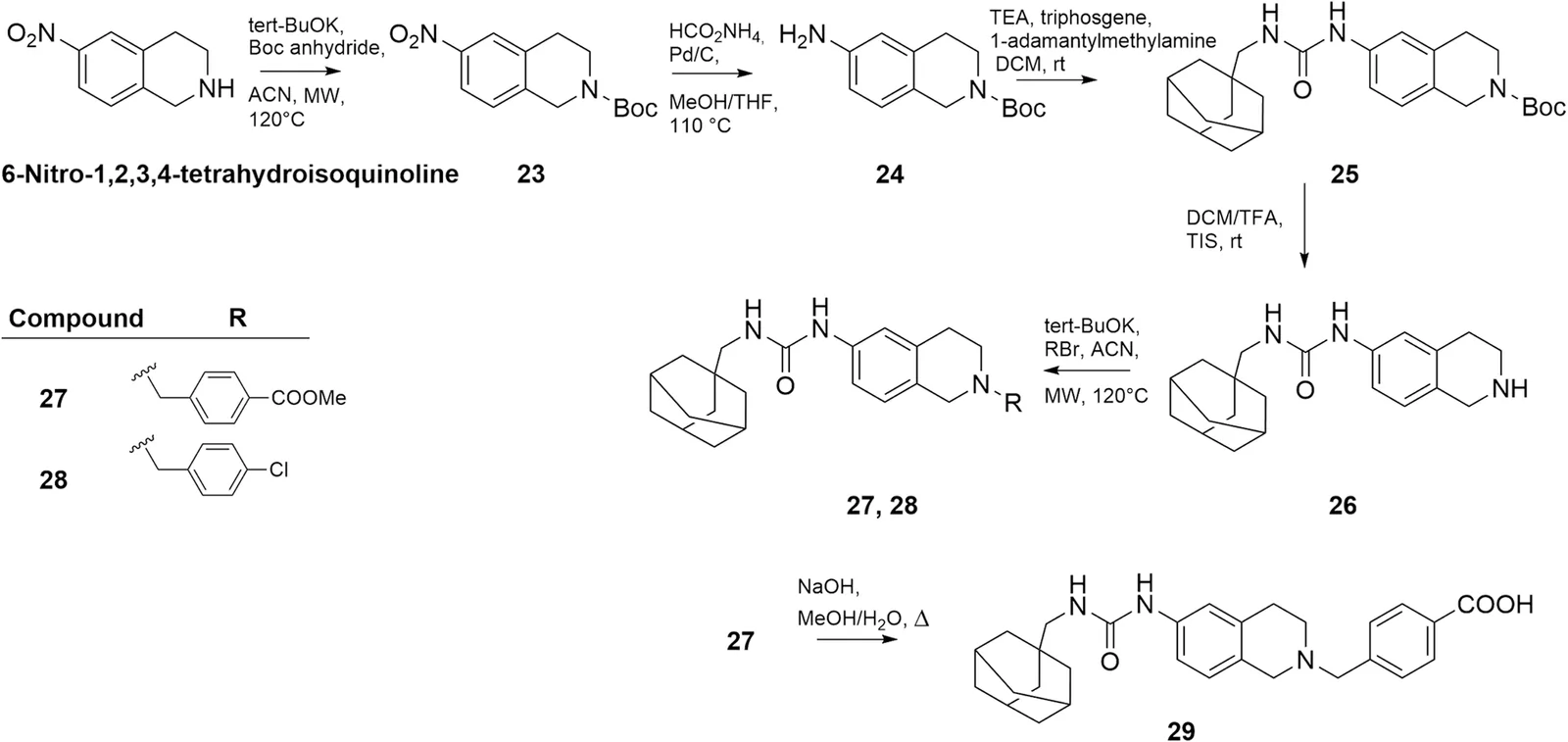
Synthesis of the Derivatives **28–29**

Derivatives **32**, **33**, **37**, and **38** were synthesized according to Scheme 4. For the synthesis of **33**, 1-Boc-3-aminopyrrolidine was employed as starting material. It was first converted into the urea **30** (yield 78%), using triphosgene and 1-adamantanemethylamine in basic medium, and then deprotected at position 1 in a mixture of trifluoroacetic acid and dichloromethane, as previously reported, giving **31** in quantitative yield. The following treatment with methyl 4-(bromomethyl)­benzoate under microwave irradiation furnished **32** in 66% yield. The tertiary amine obtained was then subjected to alkaline hydrolysis, leading quantitatively to **33**.

**Scheme 4:**
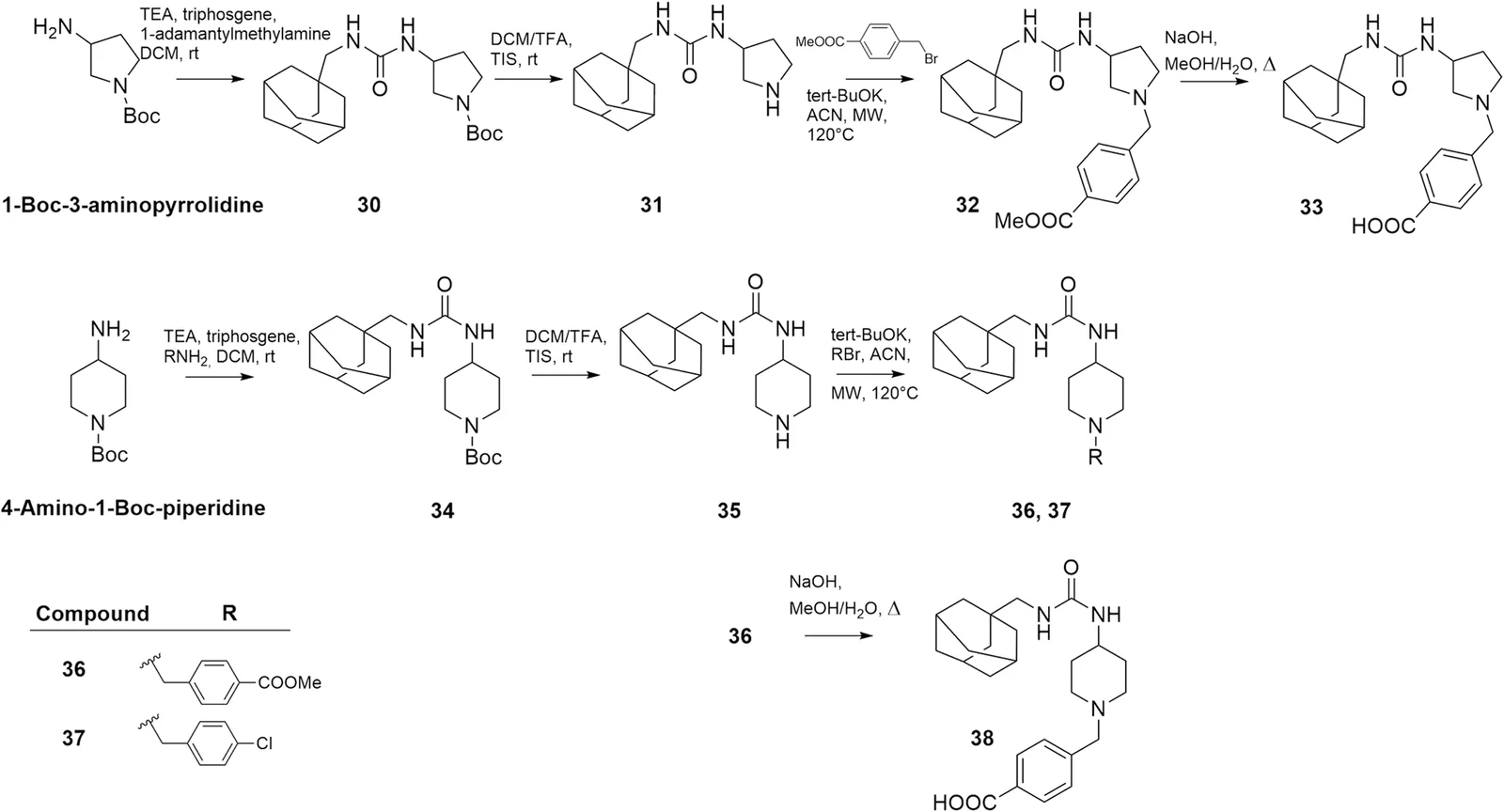
Synthesis of the Derivatives **32**, **33**, **37**, and **38**

Intermediate **36** and final compound **37** were obtained from 4-amino-1-Boc-piperidine, applying the same synthetic procedure reported for the pyrrolidine derivatives. The final compound **38** was obtained by basic hydrolysis of **36** (38% overall yield) as previously described.

### Molecular Docking and Evaluation of 5-LOX and sEH Inhibition

Recently, we reported an indoline-based derivative as new lead compound for rational design of dual 5-LOX/sEH inhibitors.^16^ Despite its valuable efficacy, pharmacokinetics concerns raised from our experimental investigation. This prompted us to further explore new chemical modifications to improve the pharmacokinetic profile while preserving biological effectiveness. Thus, a new collection of 16 compounds was designed by chemically replacing the indoline moiety by other cyclic scaffolds, namely: tetrahydroquinoline, tetrahydroisoquinoline, piperidine, pyrrole and pyrrolidine. Upon the chemical synthesis, this new library was evaluated *in vitro* for its putative inhibitory activity against human isolated sEH and 5-LOX in cell-free assays (Table 1). The inhibitory activity of most of the compounds spans in the nanomolar range against sEH, with the bicyclic-based compounds standing out the monocyclic ones. Specifically, the tetrahydroquinoline-based compound **11** showed the best inhibition (IC_50_ = 3.6 ± 0.7 nM, Table 1) of sEH activity. Indeed, molecular docking pose showed that the tetrahydroquinoline core gives π-π interaction with Y383 and H524, along with an aromatic H-bond with backbone CO of L408 (Figure 3A, Tables S1, S3). The urea moiety accepts two H-bonds and two aromatic H-bonds from side chains of Y383 and Y466 and donates two H-bonds to D335. The adamantyl side provides van der Waals contacts with Y336, M339, T360, I375, F381, Q384, Y466, L499, M503. Intriguingly, all these found intermolecular contacts are recognizable in reported cocrystallized ligands with sEH.^19,20^ The benzyl group also contributes with a π-stacking with W525, while the carboxylate is hydrogen bonded to backbone NH of F497 and H524. Interestingly, **11** shares, with known sEH inhibitor t-AUCB,^20^ the carboxyphenyl group but diverges for the interaction given by the carboxylate. Indeed, in t-AUCB, the carboxylate is hydrogen bonded to S415. This different orientation of the carboxyphenyl group in **11** is mainly ascribable to the tetrahydroquinoline scaffold respect to the cyclohexyl of t-AUCB. In respect to **11**, the congeners **5–9** give the same network of ligand-protein interactions by their common structural moieties. The compounds **5–9** structurally diverge from **11** for the substituent in *para* position of the benzyl group, showing less potency to inhibit sEH (Table 1). Indeed, **8** preserves only the H-bond with the main chain NH of F497 through the methoxy substituent (Figure 3B) while the unsubstituted analogue **5** and the ester derivative **6** do not give hydrogen bonds. The chlorine in **7** does not allow the formation of a proper polar interaction. On the contrary, the isopropyl group in **9** compensates in terms of affinity contribution through van der Waals contacts with surrounding amino acids (Figure 3C), leading to a slightly better inhibitory activity than **5–7** (Table 1). Unlike **5–9** and **11**, compound **12** is endowed with a carbonyl linker between tetrahydroquinoline and phenyl rings instead of a methylene. This structural modification widens the planar geometry of the N-substituent, and the consequent conformational rigidity does not favor the right adaptation into the binding site of sEH. Notably, the tetrahydroisoquinoline **28** also displays potent inhibition of sEH (6.5 ± 1.4 nM, Table 1). Indeed, **28** keeps the same intermolecular contacts formed by the 1-(adamantan-1-ylmethyl)-3-phenylurea moiety as observed for **11** (Figure 3D). Moreover, **28** establishes two potential polar interactions with side chain of S415 and the backbone CO of A411. The benzyl group gives two aromatic H-bonds with main chain carbonyl of L408 and R410. However, compared to **11**, the tetrahydroisoquinoline isomer increases the ligand length. For **29**, the larger carboxylic substituent than chlorine hampers the proper accommodation into the catalytic pocket, leading to loss of activity. The structural switch from bicyclic to monocyclic hydrogenated scaffolds (**32**, **33**, **37**, and **38**) shortens the span of the small molecule and accordingly a more extended conformation is only permitted to reduce the intermolecular steric hindrances. This conformational adaptation is respected by using ester and chlorine substituents in **32** and **37**. Differently, the negatively charged carboxylate in **33** and **38**, attempting to find a macromolecular partner into the desolvated binding site, induces inter and intramolecular steric clashes, which are detrimental for the binding. Unlike the saturated five and six-membered rings, the rigidity and planarity of pyrrole confer more relaxed conformations, even in the presence of a carboxylate, assuring an inhibitory activity of **20–22** in high nanomolar range (Table 1).

**Figure 3:**
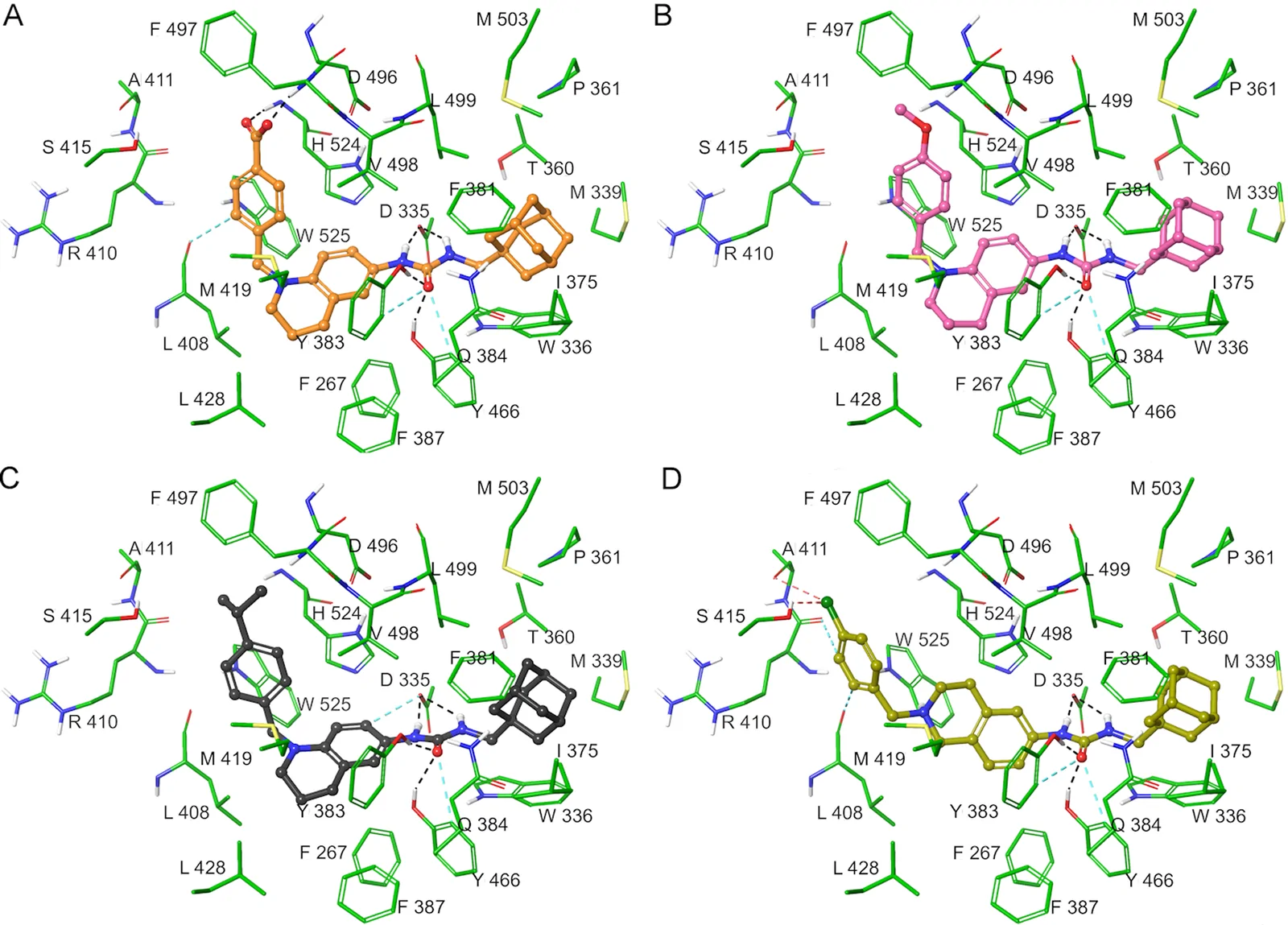
Three-dimensional model of the interactions given by **11** (A), **8** (B), **9** (C), and **28** (D) with sEH. The protein is represented by green tube with the atom color code: C, green; polar H, white; N, dark blue; O, red; S, yellow. The ligands are depicted by sticks (orange for **11**, faded salmon for **8**, dark gray for **9**, kiwi for **28**) and balls (with the atom color code used for the protein except for C, as for the sticks). The dashed black, cyan and faded red lines indicate respectively the hydrogen-, aromatic H- and halogen bonds between the ligand and protein.

**Table 1:**
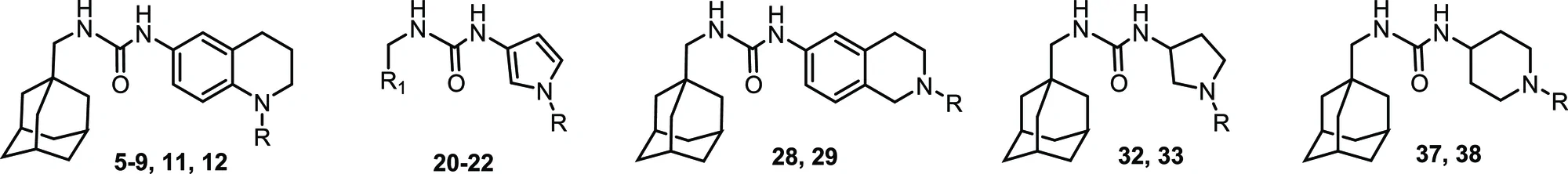
Inhibition of Human Isolated sEH and Human Isolated 5-LOX^a^

Compound	R	R_1_	IC_50_ for sEH (nM)	IC_50_ for 5-LOX (nM)
**5**	Ph–CH_2_–	-	28.3 ± 23.9	94.1 ± 28.3
**6**	4-COOMe-PhCH_2_–	-	32.8 ± 17.4	93.7 ± 25.0
**7**	4-Cl-PhCH_2_–	-	32.0 ± 22.6	51.7 ± 27.0
**8**	4-OMe-PhCH_2_–	-	17.2 ± 15.0	105.1 ± 29.9
**9**	4–CH(CH_3_)_2_-Ph-	-	21.3 ± 20.0	209.4 ± 62.9
**11**	4-COOH-PhCH_2_–	**-**	**3.6 ± 0.7**	**300 ± 0.14**
**12**	4-COOH-PhCO-	-	>100	>10,000
**20**	4-COOH-PhCH_2_–	Cyclohexil-CH_2_–	102.8 ± 76.2	>10,000
**21**	4-COOH-PhCH_2_–	AdamantylCH_2_–	289.2 ± 78.9	>10,000
**22**	NH_2_(CH_2)4_-	AdamantylCH_2_–	379.3 ± 229.3	>10,000
**28**	4-Cl-PhCH_2_–	-	6.5 ± 1.4	>10,000
**29**	4-COOH-PhCH_2_–	-	>100	>10,000
**32**	4-COOMe-PhCH_2_–	-	68.1 ± 4.6	>10,000
**33**	4-COOH-PhCH_2_–	-	>100	>10,000
**37**	4-Cl-PhCH_2_–	-	34.2 ± 25.1	>10,000
**38**	4-COOH-PhCH_2_–	-	>100	>10,000

a All values are given as mean ± S.E.M. of single determinations obtained in 3–4 independent experiments.

Only the tetrahydroquinoline-based compounds inhibit isolated 5-LOX effectively (Table 1). The theoretical investigation highlighted that chlorine of **7** interacts with Fe^2+^. The tetrahydroquinoline moiety gives van der Waals with F177, I406, K409, L607, I673 (Figure 4A). The urea group accepts a hydrogen bond from side chain of N180 and donates two H-bonds to the CO of A606. The adamantyl group establishes van der Waals bonds with K183, W605, A606, Q609, and Q611. The phenyl group gives an aromatic H-bond with side chain of Q363 and van der Waals interactions with H367, H372, and L414. The larger ester substituent in **6** is H-bonded and induces also the loss of a hydrogen bond from the urea group and some van der Waals interactions mediated by the adamantyl moiety (Figure 4B). Similarly, **5** and **8** show reduced numbers of contacts with protein (Figure 4C,D). Compared to **7**, the bulkier isopropyl group of **9** further moved the whole conformation toward the protein surface, reducing the van der Waals contacts with macromolecular counterparts with complete loss of H-bond network of the urea (Figure 4E). In respect to **7**, the carboxylate group in *para* position of phenyl in **11** does not interact with Fe^2+^ but it is hydrogen bonded to side chain of H367, moving apart also the phenyl ring from the ion (Figure 4F). These differences could explain the higher IC_50_ of **11** than **7** (Table 1). The reduced flexibility of **12**, due to the conversion of methylene into a carbonyl linker, causes a distorted docked pose, advocating an unfavorable inhibition as corroborated by the enzyme inhibition experiments (Table 1). The theoretical outcomes of **20–22**, **28**, **29**, **32**, **33**, **37**, **38** revealed distorted predicted poses or no docked conformations. In comparison with the tetrahydroquinoline-based congeners, the introduction of tetrahydroisoquinoline and monocycle scaffolds alters the global linker length and the conformational plasticity of the small molecule to adapt into the catalytic channel of 5-LOX. Thus, the occupancy of the open position of Fe^2+^ coordination sphere could not be properly reached, resulting in disappearance of inhibitory activity. To evaluate the stability over the time of the key interactions found by molecular docking investigation of **11** against sEH and 5-LOX, we carried out molecular dynamics (MD) simulations (100 ns, 310 K), illustrated in Figure 5.^21–23^ For each trajectory investigated, most of the contacts with macromolecular residues, observed from the docked poses of **11** with both biological targets, were maintained during the whole simulation (>30%), confirming their crucial contribution to ligand-protein complex stability.^24–26^

**Figure 4:**
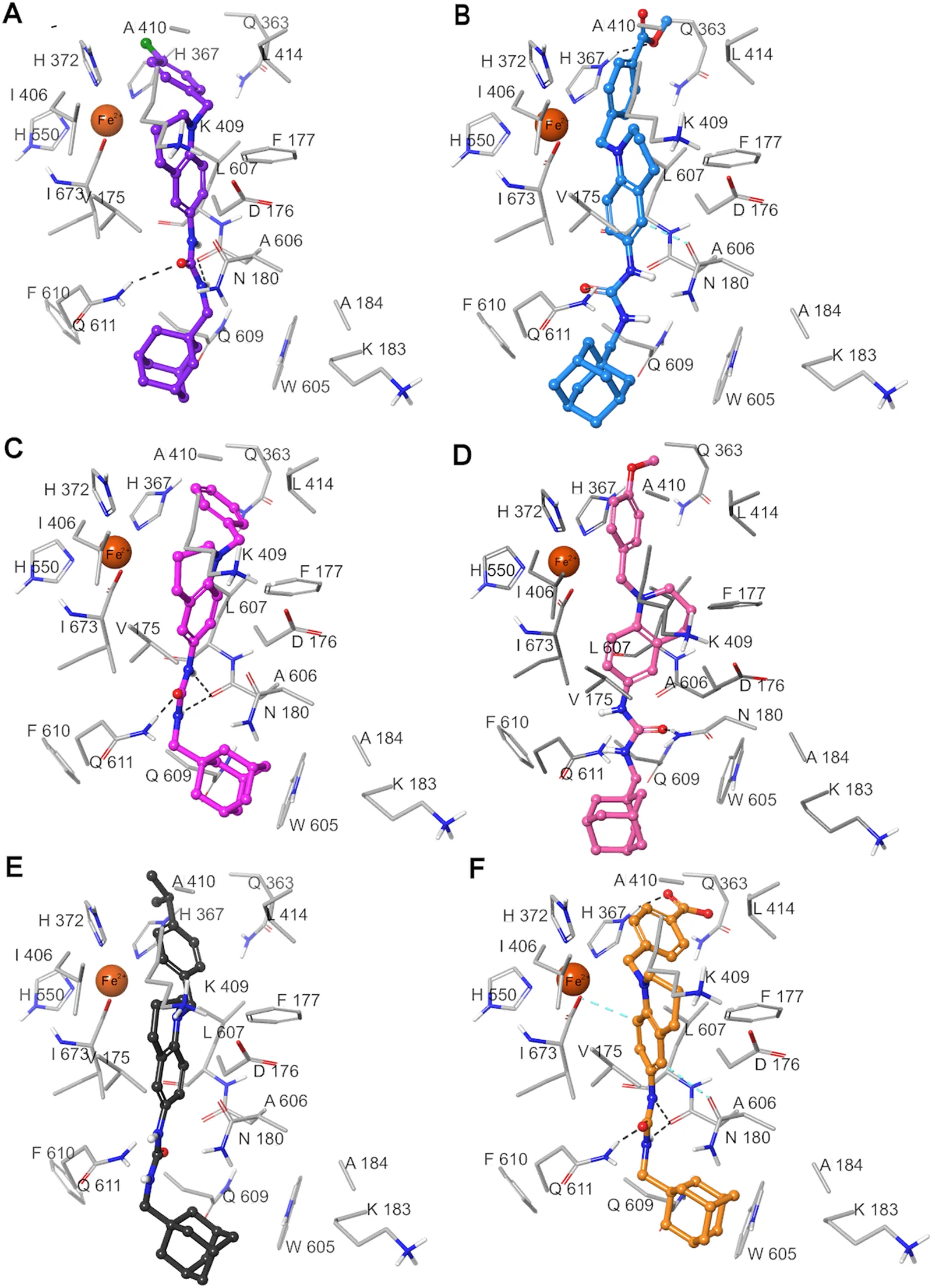
Three-dimensional model of the interactions given by **7** (A), **6** (B), **5** (C), **8** (D), **9** (E), and **11** (F) with 5-LOX. The protein is depicted by tube (colored: C, light gray; polar H, white; N, dark blue; O, red). The ligands are represented by sticks (violet for **7**, azure for **6**, magenta for **5**, faded salmon for **8**, dark gray for **9**, orange for **11**) and balls (with the atom color code used for the protein except for C, as for the sticks). The dashed black and cyan lines indicate the respectively the hydrogen- and aromatic H-bonds between the ligand and protein.

**Figure 5:**
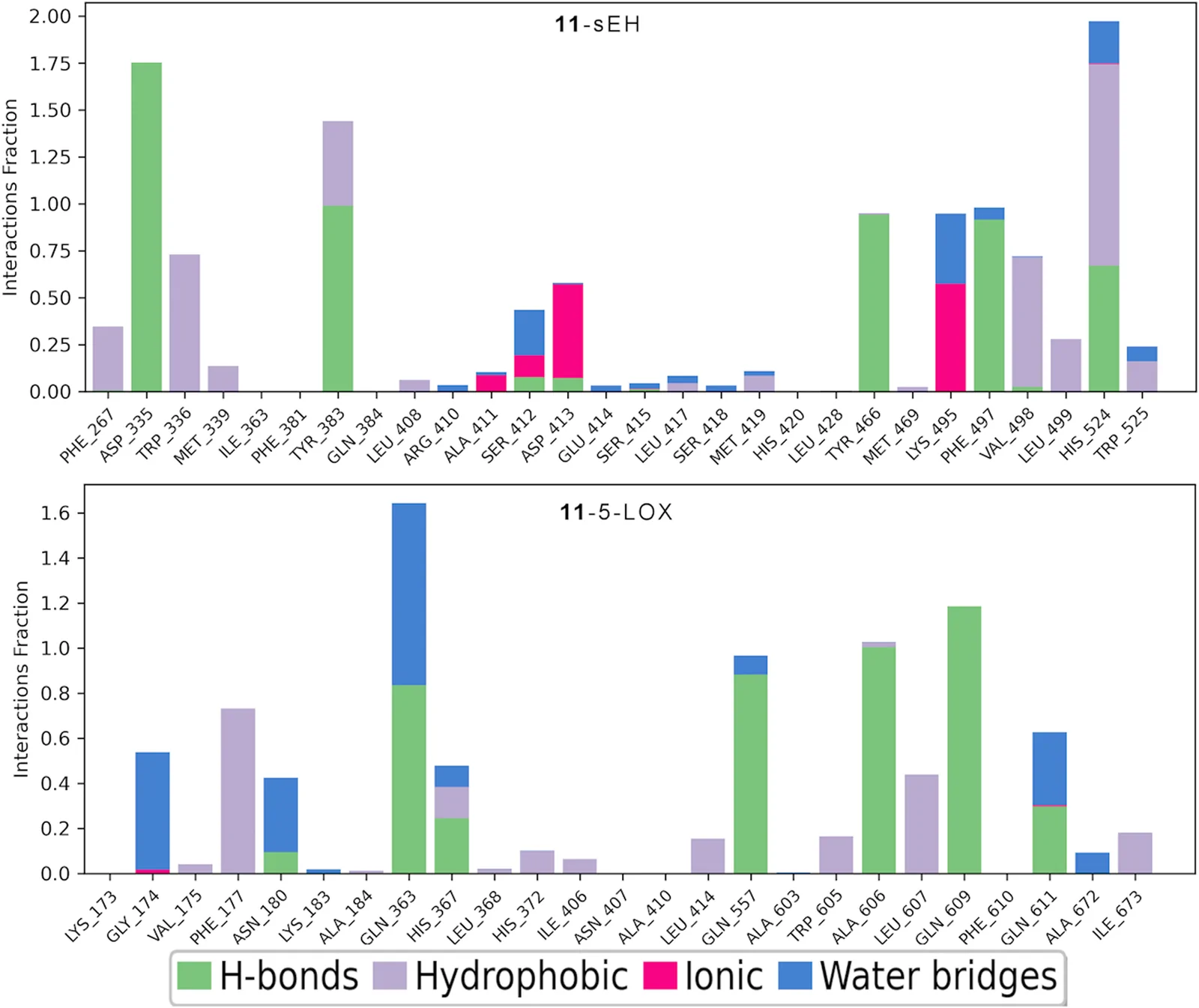
Contact histograms from MD simulations of **11** with sEH (top) and 5-LOX (bottom).

Overall, the present work shows the crucial role of the central scaffold in leading the dual inhibitory profile. Indeed, the tetrahydroquinoline-based compounds are uniquely able to inhibit both aimed biological targets. The conversion of methylene linker to carbonyl one between the tetrahydroquinoline and phenyl group is detrimental for inhibition of both targeted enzymes. The structural switch to tetrahydroisoquinoline and/or to saturated and unsaturated five- and six-membered monocycles drives to a binding preference toward sEH. However, these cycles should be coupled with the right choice of the *para* substituent of benzyl group to preserve the sEH inhibition.

Together, the compound characterization highlights nanomolar inhibitory potencies against sEH for the most synthesized derivatives, with compound **11** at the top ranking of the series (IC_50_ = 3.6 ± 0.7 nM), while derivatives **5–11** emerged as the most powerful inhibitors of 5-LOX (IC_50_ ≤ 51.7 ± 27.0 ≤ 300 ± 0.14 nM). So, balancing 5-LOX and sEH activities, and at the same time knowing that sEH blocking potentiates the endogenous anti-inflammatory pathway, while 5-LOX reduces both pro-inflammatory and anti-inflammatory LM biosynthesis, we considered compound **11** worthy of further investigation.^27^

It should be noted that *in vitro* assays were conducted on human enzymes, whereas *in vivo* studies were performed in mice. Although these enzymes are highly conserved, species-related differences cannot be excluded and should be considered when interpreting the translational relevance of the results.

### *In Vitro* Pharmacokinetic Evaluation of Compound **11**

Prior to *in vivo* evaluation of compound **11**, its *in vitro* metabolic stability was assessed using human liver microsomes (HLMs), which are widely used as early stage screening tools because of their robustness, simplicity, and low cost.^28,29^ HLMs provide a suitable *in vitro* system to model major microsomal hepatic biotransformation pathways, as they contain membrane-bound phase I enzymes, such as CYPs and flavin-containing monooxygenases (FMOs), which primarily catalyze NADPH-dependent oxidative reactions,^30^ as well as phase II enzymes, including UDP-glucuronosyltransferase (UGTs), which are responsible for glucuronidation.^31^

Accordingly, **11** was incubated with NADPH or UDP-glucuronic acid (UDP-GlcUA) to evaluate oxidative metabolism mediated by NADPH-dependent enzymes and UGT-mediated glucuronidation, respectively. Compound **11** displayed moderate metabolic stability in HLMs, with half-lives of approximately 61 min in the presence of NADPH and 230 min in the presence of UDP-GlcUA (Figure 6A,E). In cofactor-free control incubations, no appreciable parent compound depletion was observed, supporting cofactor-dependent metabolic turnover (Figure 6B,F). Moreover, incubation with the broad-spectrum CYP inhibitor 1-aminobenzotriazole (ABT) further supported the involvement of CYP-mediated oxidative metabolism (Figure 6B).^32^

**Figure 6:**
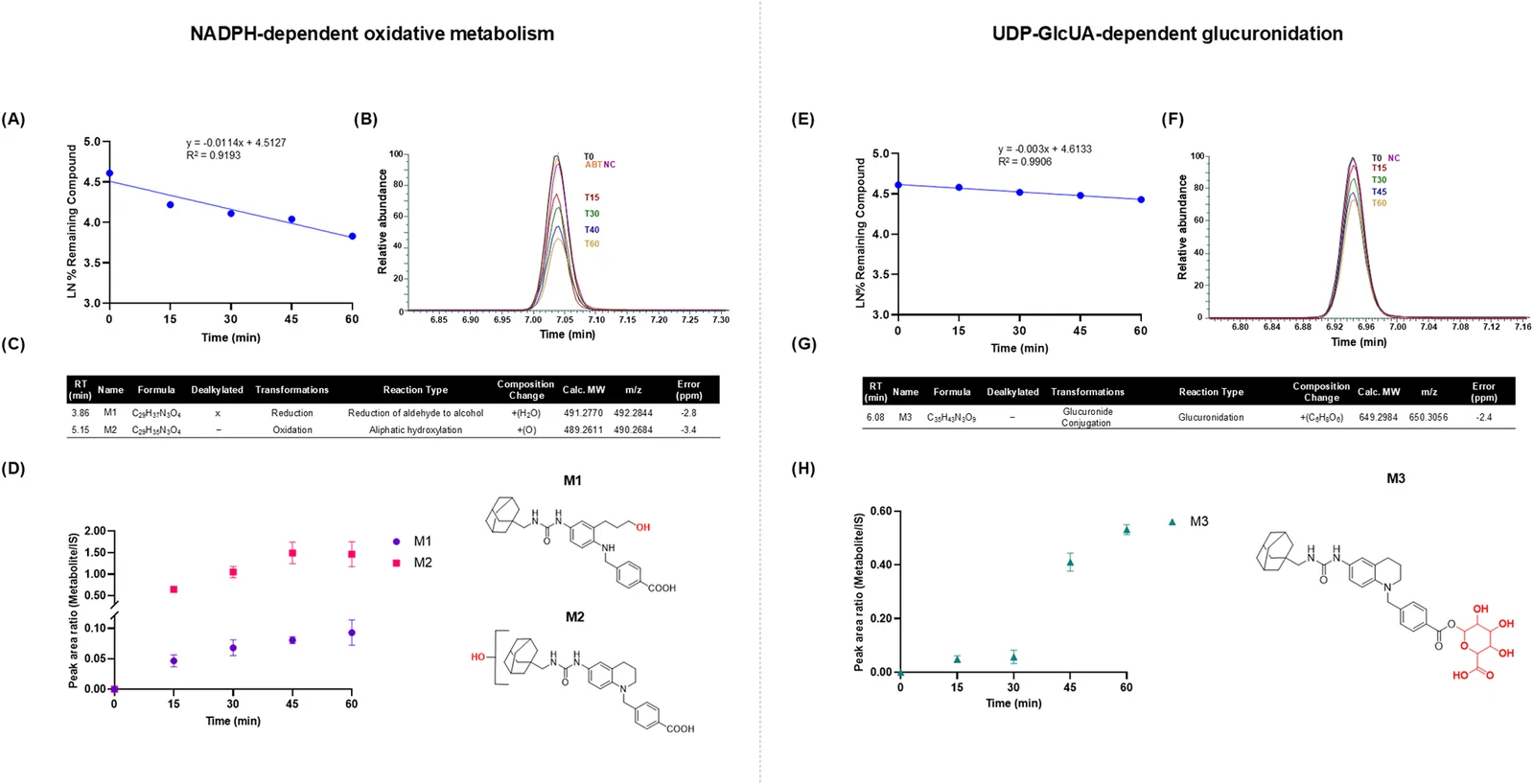
Assessment of the metabolic stability of compound **11** following incubation with HLMs and LC-HRMS/MS characterization of metabolites. (A, E) The plot reports the natural logarithm of the percentage of parent compound remaining versus incubation time. Metabolic stability of compound 11 was evaluated after incubation with HLMs at 37 °C in the presence of NADPH or UDP-GlcUA as cofactors. Compound 11 (10 μM) was prepared with a final organic solvent concentration of <1% (v/v) to prevent CYP inhibition. Low microsomal protein concentrations were employed to minimize nonspecific binding. Using a protein precipitation (crash-in) protocol, incubations were quenched at predefined time points with ice-cold methanol containing IS and analyzed by LC-MS/MS. A 0 min control was prepared by immediately quenching the reaction with solvent. Cofactor-free incubations served as negative controls (NC; B, F), while 1-aminobenzotriazole (ABT)-supplemented incubations were used to assess the contribution of CYP-mediated metabolism; (C, G) List of metabolites formed after microsomal incubation of compound 11. Metabolites were identified by LC-HRMS/MS; fragmentation spectra were processed using Compound Discoverer 3.3 software with a customized workflow for the detection and structural elucidation of both predicted and unexpected metabolites; (D, H) Semiquantitative analysis of metabolites over incubation time based on LC-HRMS/MS data. Metabolite levels (M1 and M2, panel A; M3, panel B) are reported as normalized peak areas relative to IS.

These metabolic stability data were used to derive intrinsic clearance parameters, which are key predictors of human hepatic clearance and systemic exposure. CYP- and UGT-mediated microsomal intrinsic clearances of **11** were calculated from *in vitro* half-life data, normalized to microsomal protein content, and corrected for microsomal binding (*f*_u,mic_ = 0.501) to obtain unbound intrinsic clearance values. These values were then scaled to whole-liver intrinsic clearance using physiological scaling factors, yielding CL_int,scaled_ values of 37.3 and 9.9 mL min^–1^ kg^–1^ for CYP- and UGT-mediated metabolism, respectively.^33,34^ The corresponding fractions metabolized (fm_,CYP_ = 0.79 and fm_,UGT_ = 0.21) confirmed that **11** is predominantly metabolized *via* CYP-mediated pathways.^35^ Hepatic clearance (CL_h_) was estimated using the well-stirred model, incorporating hepatic blood flow (*Q*_h_ = 20.7 mL min^–1^ kg^–1^), plasma unbound fraction (*f*_u,p_ = 0.235), and the combined scaled intrinsic clearances for CYP- and UGT-mediated metabolism, yielding CL_h_ = 7.2 mL min^–1^ kg^–1^.^36,37^

Parent compound **11** depletion was accompanied by time-dependent metabolite formation. CYP-mediated metabolism generated two metabolites: **M1**, formed by dealkylation followed by aldehyde reduction, and **M2**, formed through adamantane hydroxylation (Figure 6C and Figure S50A, B), with **M2** being the predominant product (Figure 6D). In contrast, UGT-mediated metabolism yielded the glucuronide conjugate **M3** (Figure 6G, H and Figure S50C).

Because metabolic turnover can generate reactive intermediates, **11** was evaluated for its propensity to form glutathione (GSH)-trappable metabolites.^38,39^ Accordingly, **11** was incubated with HLMs in the presence of NADPH and GSH, and potential GSH adducts were monitored by LC-MS/MS. No GSH conjugates were detected, indicating low electrophilic reactivity and limited GSH-trapping liability, thereby supporting a low propensity for reactive metabolite formation.

As a final component of the *in vitro* stability assessment beyond hepatic metabolism, **11** was evaluated in human plasma. No appreciable degradation of **11** was observed after incubation for up to 120 min, indicating good plasma stability and limited susceptibility to plasma-mediated degradation (Figure S51).

### *In Vivo* Effects of Compound **11** in Cerulein-Induced Pancreatitis

To assess the effects of compound **11** *in vivo*, we used a well-established model of AP namely CER-induced pancreatitis in mice.^17^ CER is a homologue of the intestinal hormone cholecystokinin which, at high concentrations, induces acinar cell death, pancreatic edema and cell infiltration, hallmark features of human pancreatitis. To this aim, the mice were treated intraperitoneally with **11** (10 mg/kg), AUDA (a sEH inhibitor, 10 mg/kg) and zileuton (ZILE, a 5-LOX inhibitor, 10 mg/kg) 0.5 and 2.5 h after the first administration of CER (Figure 7A). The mice received a total of 6 intraperitoneal injections of CER at 1-h intervals; at the end of the experiment, the mice were euthanised 1 h after the last CER injection, and the pancreas, lungs and blood were collected (Figure 7A). Assessment of AP requires the evaluation of amylase and lipase plasma levels as well as histological parameters, as they reflect distinct but complementary aspects of the disease. Amylase and lipase levels provide information on early acinar cell injury, while histological analysis captures the extent of tissue inflammation and structural damage.^40^ Following administration of CER, a significant increase in amylase (Figure 7B) and lipase (Figure 7C) levels was observed in the plasma of mice in respect to the sham mice, indicating that the disease had been induced. Treatment of the mice with **11** significantly reduced the amylase and lipase levels compared to the vehicle group (Figures 7B, C), suggesting a protective role for **11** against pancreatic damage. Amylase and lipase levels were also reduced following treatment with ZILE, whereas no effect was observed with AUDA (Figure 7B, C).

**Figure 7:**
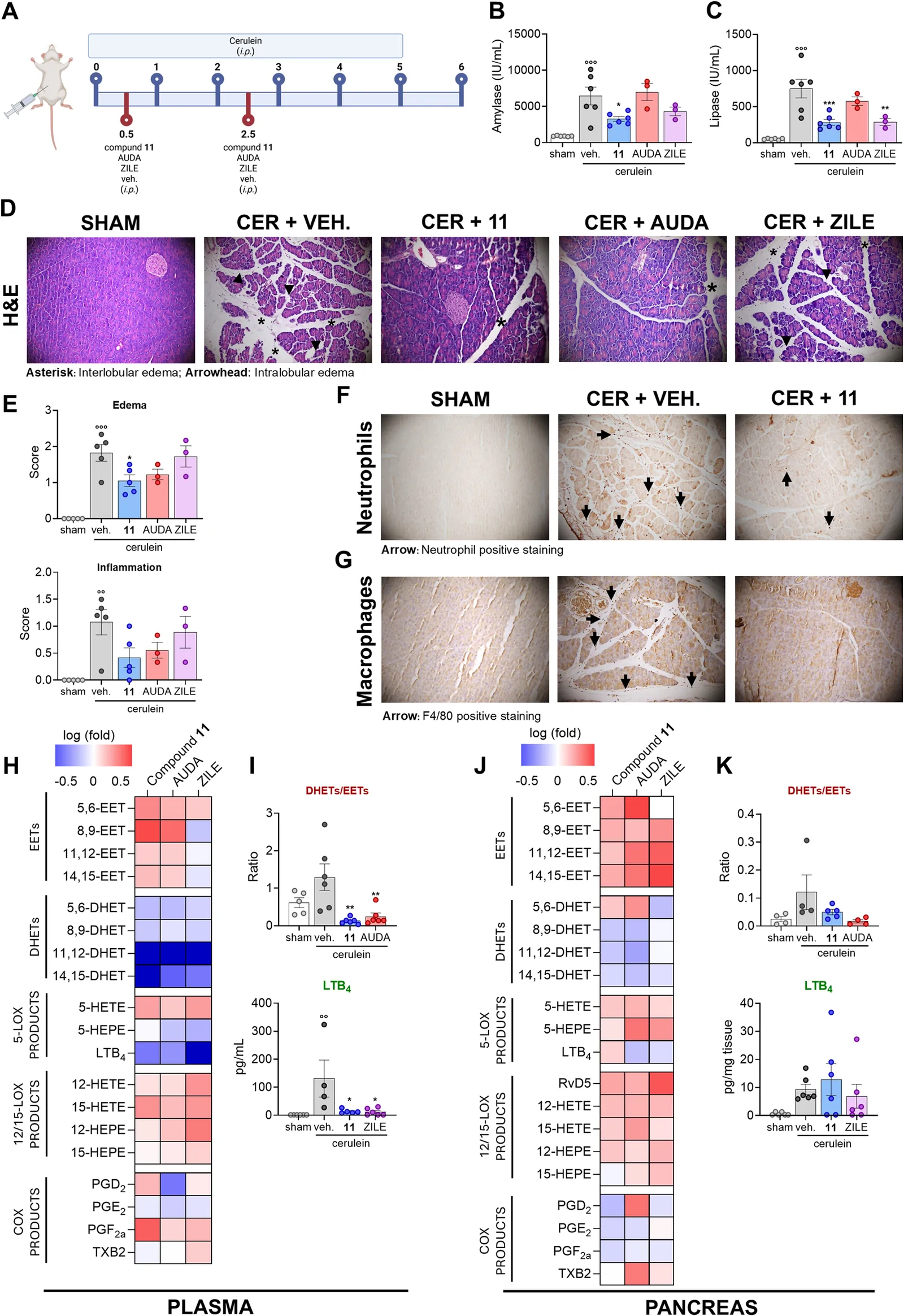
Effect of compound **11** in a murine model of acute pancreatitis. (**A**) Time scale for cerulein (CER)-induced murine pancreatitis. AP was induced in mice by i.p. injections of cerulein (CER, 50 μg/kg) hourly (6 times). Mice received compound **11** (10 mg/kg, i.p.), AUDA (10 mg/kg, i.p), Zileuton (ZILE, 10 mg/kg, i.p.) or vehicle (veh., 2% DMSO, i.p.), 0.5 and 2.5 h after the first CER injection and were euthanized 6 h after the first CER injection. (B) Amylase and (C) lipase levels were measured in plasma 6 h after the first CER injection. (D) Pancreas slices were stained for H&E. (E) Evaluation of pancreatic edema and inflammatory infiltration was performed by three-point scoring system. Edema: 0 absent or rare; 1, in the interlobular space; 2, in the intralobular space; 3, the isolated-island shape of pancreatic acinus. Inflammation: 0, absent; 1, mild (infiltration in ducts); 2, moderate (infiltration in parenchyma <50%); 3, severe (infiltration in parenchyma >50%). Asterisk = interlobular edema, arrowhead = intralobular edema. (F) Neutrophil and (G) macrophage immunohistochemical analysis in pancreas sections. LMs were extracted from (H and I) plasma and (J and K) pancreas and analyzed by UPLC-MS/MS. Ratio of the sum of DHETs against the sum of EETs and LTB_4_ levels in (I) plasma and (K) pancreas. Values represent mean ± standard error of the mean (S.E.M.); *n* = 4–6 mice for each group. Heatmap of (H) plasma and (J) pancreas LMs levels, shown as log of fold changes against the vehicle-treated mice; *n* = 4–6 mice for each group. Data were analyzed by one-way analysis of variance (ANOVA) plus Bonferroni. Statistical significance is reported as follows: °°*p* < 0.01 and °°°*p* < 0.001 vs sham; *p < 0.05 and **p < 0.01 vs veh.

Pancreatic tissue from the sham group showed a preserved exocrine architecture, with acinar cells showing basally located nuclei and apical acidophilic cytoplasm, as well as intact islets of Langerhans (Figure 7D). In contrast, CER-treated animals displayed extensive pancreatic injury, including acinar cell hypertrophy, necrosis, and ductal alterations (Figure 7D). Additionally, marked dilation of interlobular spaces and disruption of islet organization with intra- and interlobular edema were observed in CER-treated mice (Figure 7D, E). Pancreatic sections from the **11**-treated group showed a significant attenuation of histopathological damage (Figure 7D), with reduced acinar hypertrophy, necrosis, and pancreatic edema (Figure 7D, E). In perfect tone, **11** reduced immune cell infiltration induced by CER injections (Figure 7D, E), in particular neutrophil (Figure 7F) and macrophage (Figure 7G) infiltration. Of note, the treatment with AUDA, similarly to compound 11, attenuated pancreatic damage at the histopathological level, whereas ZILE provided only partial protection, with residual histopathological alterations still evident (Figure 7D, E). These findings suggest that the dual inhibition of sEH and 5-LOX achieved with 11 affords superior protection compared to targeting a single pathway, acting both at the biochemical level (reduction of amylase and lipase), as observed with ZILE, and at the histopathological level, as observed with AUDA.

To evaluate the *in vivo* modulation of 5-LOX and sEH by **11**, targeted LM profile was analyzed in plasma and pancreas of treated mice by UPLC–MS/MS. Compound **11** selectively modulated pro-inflammatory and anti-inflammatory LM profiles after AP induction. Consistent with the capacity of **11** to inhibit *in vitro* sEH enzyme, LM profiling revealed that treatment with **11** decreased the DHET/EET *ratio* in plasma (Figure 7I) and pancreas (Figure 7K). This shift was driven by elevated levels of anti-inflammatory EETs (Figure 7H, J), along with a concomitant reduction in pro-inflammatory DHETs (Figure 7H, J), as observed and expected for AUDA,^41^ confirming effective sEH inhibition by **11** *in vivo*. In addition, **11** suppressed CER-induced formation of LTB_4_ in plasma (Figure 7H, I), but no inhibitory effects were observed in pancreas (Figure 7J, K), likely reflecting limited tissue exposure/penetration under inflammatory conditions, nevertheless, dedicated and properly optimized PK studies, including reliable quantification of the compound in pancreatic tissue, would be required to confirm this hypothesis. A comparable pattern was observed for ZILE, which markedly reduced LTB_4_ levels in plasma (Figure 7H, I) while showing minimal effects in pancreatic tissue (Figure 7J, K). These findings support a predominantly systemic modulation of 5-LOX-derived mediators.

This limited modulation of 5-LOX-derived mediators at the pancreatic level may be explained, at least in part, by PK considerations. Based on the *in vivo* PK profile of **11** (see above *in vivo* PK paragraph), the observed *C*_max_ (232.094 ng/mL) corresponds to approximately 490 nM, which is only moderately above the *in vitro* EC_50_ value for 5-LOX inhibition (300 nM, ≈143 ng/mL). These data suggest that, while systemic 5-LOX inhibition is pharmacologically plausible, the relatively narrow exposure margin may result in partial target engagement, particularly in pancreatic tissues where higher local concentrations are required.

At 6 h following CER injection, treatment with **11** resulted in a weak increase in plasma (Figure 7H) and pancreatic (Figure 7J) levels of 12-/15-LOX products derived from AA or eicosapentaenoic acid (EPA), along with a concomitant reduction in COX products, most notably PGE_2_ (Figure 7H, J), which was not due to the direct inhibition of isolated COXs enzymes (at 1 and 10 μM) as represented in Figure S52.

Elevated levels of anti-inflammatory EETs accompanied by reduced formation of their less active DHET metabolites shift the lipid redox balance toward decreased lipid peroxidation, thereby limiting susceptibility to ferroptosis, an iron-dependent form of regulated cell death implicated in pancreatic acinar cell damage and necrosis during severe AP.^8^ Immunohistochemical analysis showed that CER administration induced a strong increase of 4-hydroxynonenal (4-HNE) levels in the pancreas (Figure 8A), a toxic aldehydic byproduct of lipid peroxidation that reflects iron-dependent oxidative membrane damage and is considered a hallmark of ferroptotic cell death.^42^ Treatment of mice with **11** **(10 mg/kg, i.p.)**markedly reduced the immunohistochemical localization of 4-HNE, indicating a suppression of lipid peroxidation and suggesting an inhibitory effect on ferroptosis. This interpretation was further supported by changes in the ferroptosis-regulating proteins ferritin heavy chain-1 (FTH-1) and glutathione peroxidase-4 (GPX4). FTH-1 limits iron-driven lipid peroxidation through iron sequestration,^43^ whereas GPX4 detoxifies lipid hydroperoxides,^44^ thereby preventing the formation of toxic lipid peroxidation byproducts such as 4-HNE. Consistently, CER treatment significantly reduced FTH-1 and GPX4 levels compared with sham animals, while **11** increased the expression of both proteins (Figure 8B,C). Notably, these effects were accompanied by enhanced nuclear translocation of nuclear factor erythroid 2-related factor 2 (Nrf2) due to **11**, consistent with the activation of an endogenous antioxidant and ferroptosis-suppressive transcriptional program.^45^ Indeed, as showed in Figure 8D in the sham group, Nrf2 immunoreactivity was predominantly cytoplasmic, showing limited overlap with the nuclear counterstain (blue). CER treatment induced only a modest increase in Nrf2 nuclear localization. In contrast, pancreas sections from mice treated with CER plus **11** displayed a marked increase in nuclear Nrf2, as evidenced by the strong overlap of the brown Nrf2 signal with the blue nuclear staining (arrowheads). This enhanced nuclear localization indicates activation of the Nrf2 pathway and supports a protective effect of **11** through the induction of an antioxidant and cytoprotective response. Collectively, the activation of Nrf2 by compound **11** coordinates the induction of GPX4 and FTH-1, limits 4-HNE accumulation, and ultimately suppresses ferroptosis-associated pancreatic injury.

**Figure 8:**
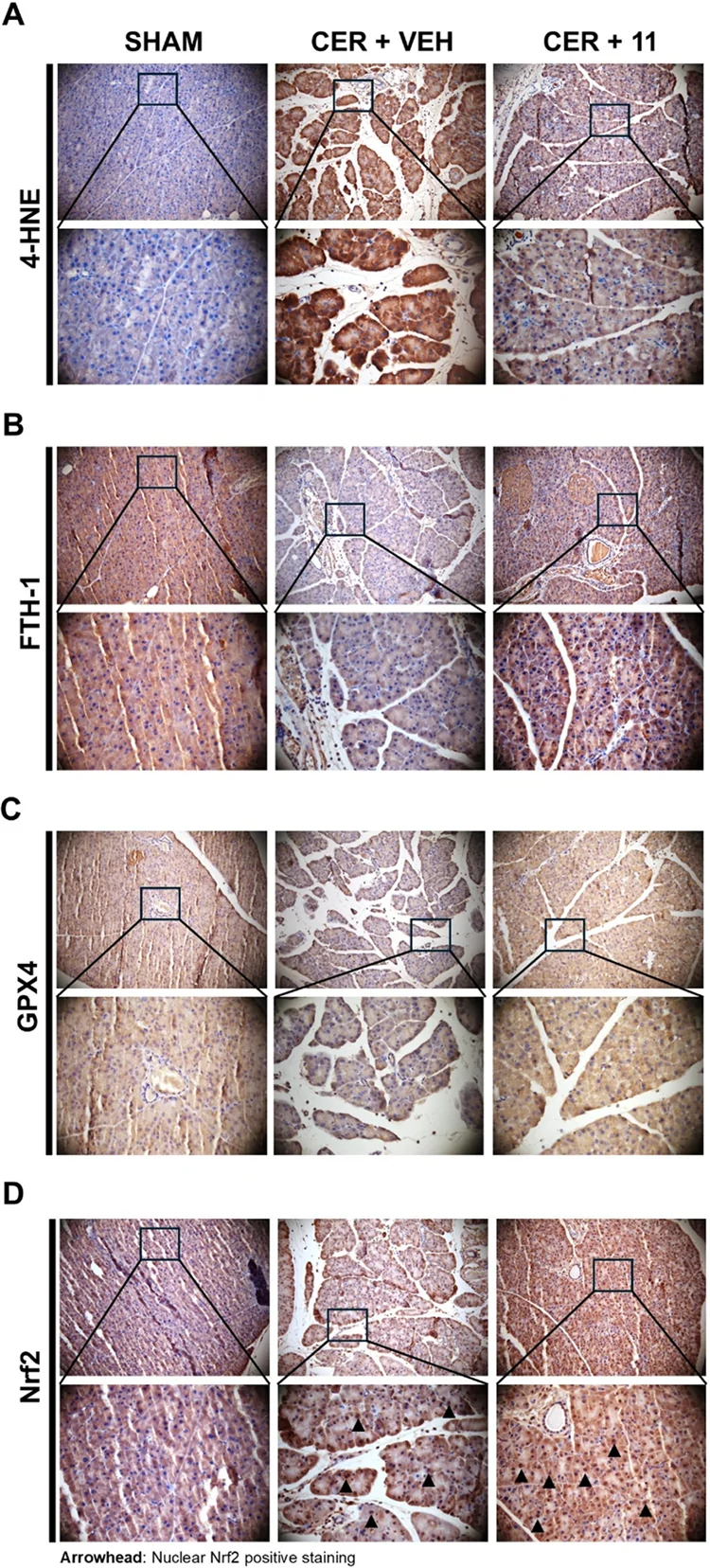
Effect of compound **11** on ferroptosis in cerulein-induced pancreatitis. AP was induced in mice by i.p. injections of cerulein (CER, 50 μg/kg) hourly (6 times). Mice received compound **11** (10 mg/kg, i.p.), or vehicle (veh., 2% DMSO, i.p.) 0.5 and 2.5 h after the first CER injection and were euthanized 6 h after the first CER injection. Representative images of (A) 4-HNE, (B) FTH-1, (C) GPX4 and (D) Nrf2 immunohistochemical analysis in pancreas sections. *n* = 4–5.

Patients with AP may die as a consequence of multiorgan failure driven by systemic inflammation, which is accompanied by secondary damage to distant organs, particularly lungs, liver and kidneys.^46^ Consistent with this scenario, CER administration in our mouse experimental model induced marked lung injury, as evidenced by alveolar edema and inflammatory infiltrates in H&E-stained sections (Figure 9A), together with a significant increase in neutrophil accumulation in lung tissue (Figure 9B). Importantly, treatment with **11** markedly attenuated CER-induced pulmonary damage, reducing both histological signs of lung injury (Figure 9A) and neutrophil infiltration (Figure 9B). Accordingly, **11** reduced pulmonary 5-LOX product levels induced by CER administration (Figure 9D), particularly the chemotactic LTB_4._ Unlike what was observed in plasma and pancreas, **11** reduced the pulmonary levels of EETs and DHETs (Figure 9C). The concurrent decrease in EETs and DHETs observed in lung tissue likely reflects reduced inflammatory-driven LM biosynthesis following treatment with **11**, rather than impaired sEH inhibition, highlighting tissue-specific regulation of epoxygenase pathways during systemic inflammation. These findings underscore the importance of tissue-specific LM dynamics and indicate that absolute EET levels may not directly reflect sEH activity under conditions of reduced inflammatory burden. In the AP model, CER administration also induced hepatic injury, as evidenced by increased plasma levels of aspartate aminotransferase (AST) (Figure 9D), alanine aminotransferase (ALT) (Figure 9E), and γ-glutamyltransferase (GGT) (Figure 9F), as well as renal dysfunction, indicated by elevated urea levels (Figure 9G), compared with sham animals. Treatment with **11** significantly reduced AST, ALT, GGT, and urea levels, restoring them toward values observed in the sham group (Figure 9D–G). Overall, compound **11** emerges as a mechanistically well-defined dual sEH/5-LOX inhibitor capable of reprogramming inflammatory LM networks and activating Nrf2-dependent cytoprotective pathways, resulting in marked attenuation of pancreatic and extra-pancreatic damage in AP.

**Figure 9:**
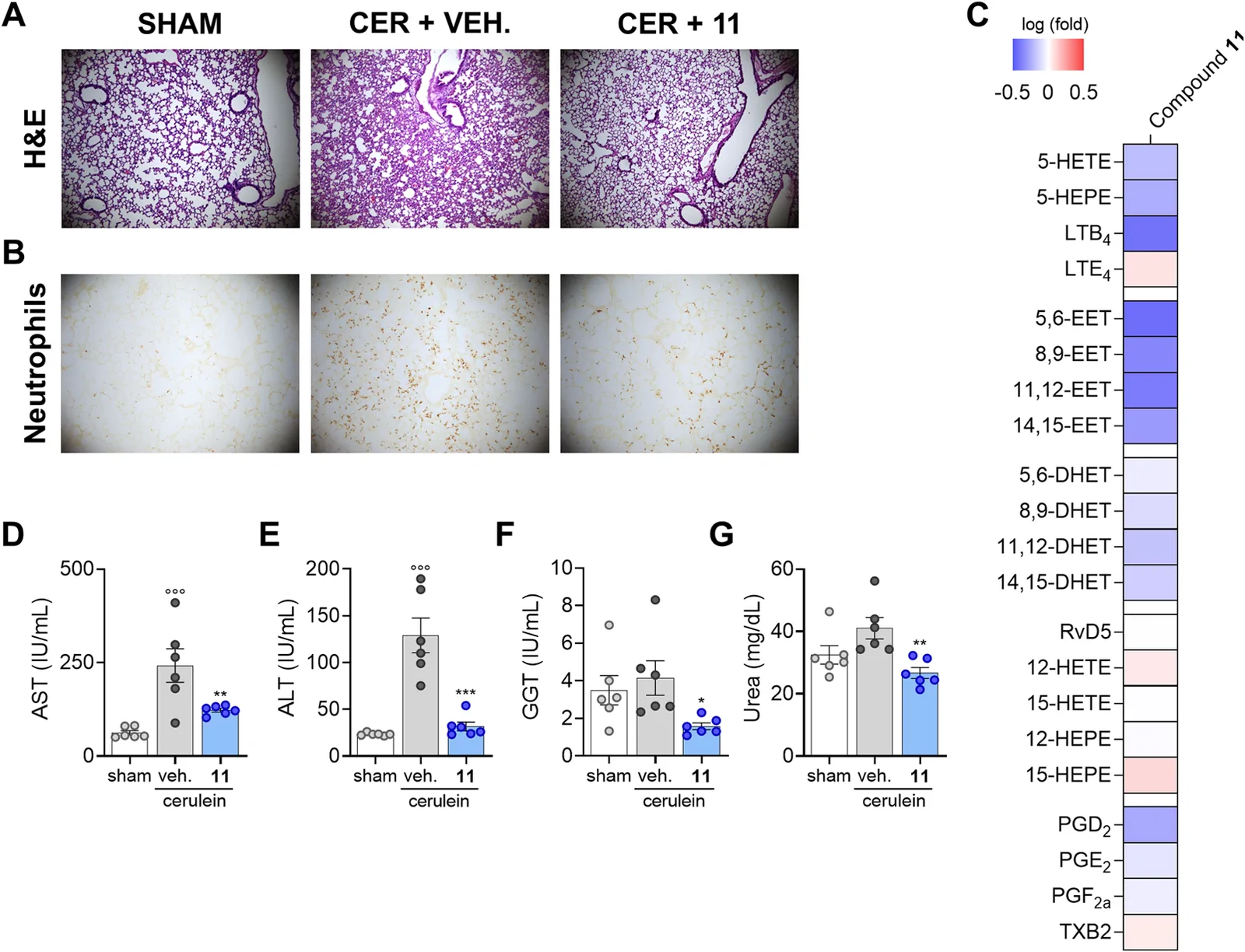
Effects of compound **11** in AP-induced multiorgan injury. Pancreatitis was induced in mice by i.p. injections of cerulein (CER, 50 μg/kg) hourly (( times). Mice received compound **11** (10 mg/kg, i.p.) or vehicle (veh., 2% DMSO, i.p.) 0.5 and 2.5 h after the first CER injection and were killed 6 h after the first CER injection. (A) H&E staining of lung sections. (B) Immunohistochemical analysis of neutrophils in lung slices. (C) Lung LMs were extracted and analyzed by UPLC-MS/MS. LMs levels are shown as log of fold changes against the vehicle-treated mice in a heatmap, *n* = **5**. Plasma levels of AST (D), ALT (E), GGT (F) and Urea (G). Values represent mean ± SEM; *n* = 6 mice for each group. Data were analyzed by one-way ANOVA plus Bonferroni. Statistical significance is reported as follows: °°°*p* < 0.001 vs sham; *p < 0.05, **p < 0.01 and ***p < 0.001 vs veh.

The overall pharmacodynamic profile of **11** appears to be compartment-dependent, with clear evidence of sEH inhibition within pancreatic tissue contributing to local protection, while modulation of 5-LOX-derived mediators is observed primarily systemically. This differential engagement likely contributes to limiting systemic inflammation while also preserving CER-induced multiorgan damage. Taken together, these alterations in LM pathways suggest a protective role for **11** in AP, likely driven by coordinated modulation of 5-LOX/sEH-related eicosanoid networks that promotes resolution of inflammation.

### *In Vivo* Pharmacokinetic Evaluation of Compound **11**

The *in vivo* pharmacokinetic properties of **11** were also assessed. To this end, plasma samples were collected from mice at predetermined time points (0, 0.5, 1, 2, 4, and 24 h) after i.p. administration of compound **11** (10 mg/kg) and were analyzed by high-performance liquid chromatography-triple quadrupole mass spectrometry (HPLC-QqQ-MS/MS, Tables S21–22) to quantify **11** and metabolites identified in the *in vitro* MetID studies. Compound **11** was relatively stable (Figure 10), exhibiting a plasma half-life of 1.71 h in mice (*T*_max_: 0.5 h; *C*_max_: 232.094 ng/mL; AUC_0–∞_: 362.2 ng/mL h), and confirmed that the main metabolites were M2 and M3 (Figure S51).

**Figure 10:**
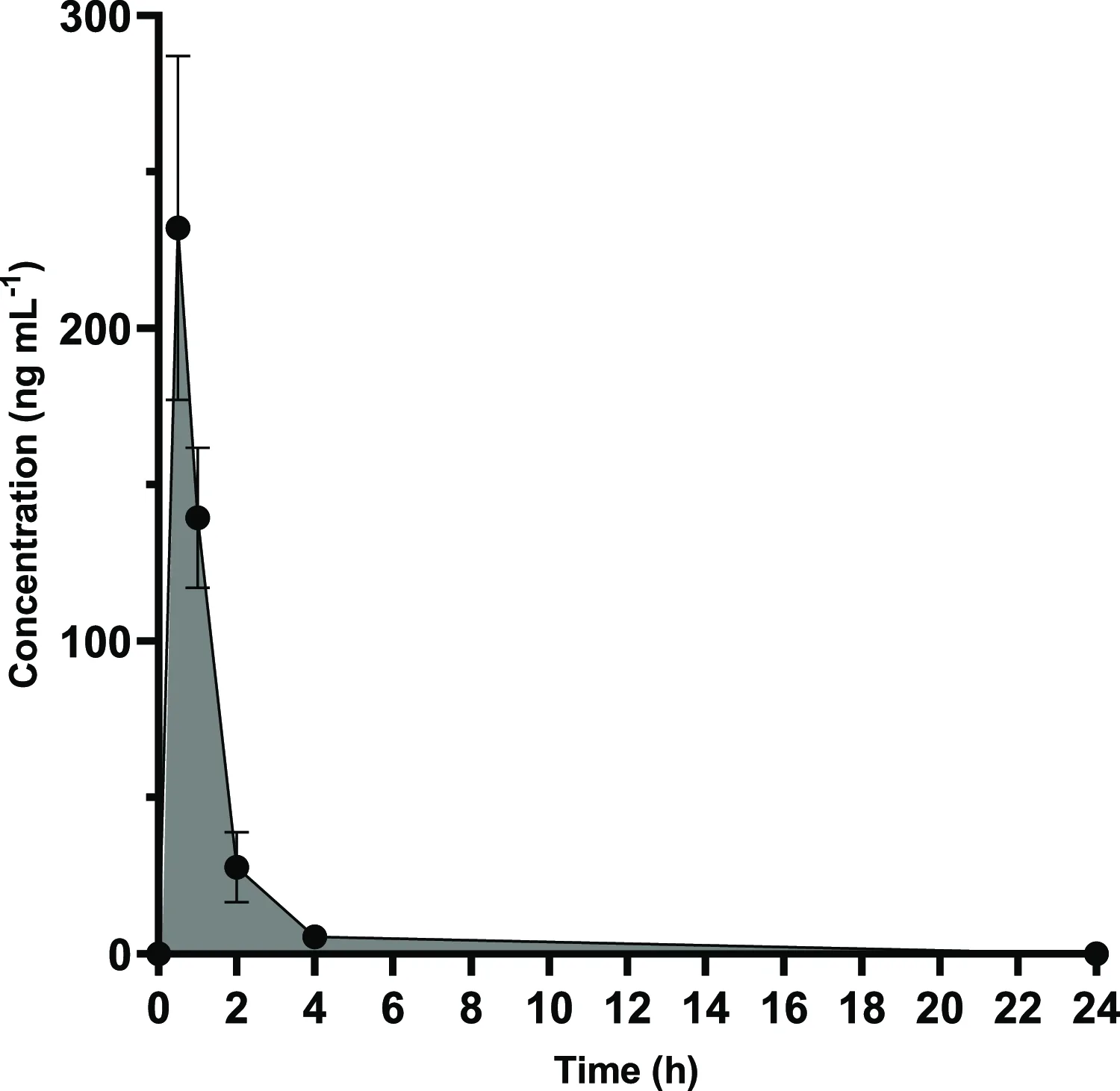
Plasma concentration–time profile of compound **11** following a single i.p. dose (10 mg/kg) in mice. Values are expressed as mean ± SD (*n* = 6).

## Conclusions

AP is among the most common causes of hospitalization due to gastrointestinal issues, imposing an economic burden higher than $2.5 billion per year, making this pathology a global health emergency.^47,48^ In addition, AP can frequently evolve to chronic pancreatitis and eventually to pancreatic cancer, with worsening of prognosis and a high rate of mortality.^49^ This disease is currently faced with a supportive treatment including fluid resuscitation, pancreatic enzymes, antibiotic and anti-inflammatory medications. Considering the important repercussions as on public health, as well as on economic expenses, AP represents an urgent need, especially in terms of new and divergent pharmacological intervention. Here, we show the design, the synthesis and the pharmacological validation of the new dual 5-LOX/sEH inhibitor compound **11**. We designed a series of molecules based on both scaffolds and appendages diversity, bearing in mind the previous structural information that we collected about these targets. All compounds were screened against the human 5-LOX and sEH isolated enzymes showing in most cases remarkable activities, in particular compound **11** emerged as the most promising candidate. To predict its pharmacokinetic profile, it was subjected to a pharmacokinetic analysis which completely traced its metabolic destiny and confirmed its suitability. In the CER-induced AP model, compound **11** strongly reduced pancreatic acinar cell hypertrophy, necrosis, and edema, decreasing levels of amylase and lipase, and favorably modulated LM signature profiles. Contextually, it also protected pancreas tissue from damages induced by ferroptosis, reducing 4-HNE, increasing FTH-1 and GPX4 levels, and promoting nuclear translocation of Nrf2. Moreover, compound **11** reduced lung, hepatic and kidney secondary injuries.

Importantly, the overall biological efficacy of **11** should be interpreted in a functional and pharmacological sense, rather than as the result of perfectly balanced inhibition at the enzymatic level of both sEH and 5-LOX. Indeed, **11** effectively engages both targets in a biologically meaningful manner, combining 5-LOX-mediated modulation of early enzymatic damage (reduction of amylase and lipase) with sEH-associated protection of tissue integrity. This coordinated and complementary activity across distinct pathological components likely contributes to limiting both early acinar cell injury and subsequent tissue damage, ultimately supporting the protective effect observed *in vivo*.

Although *in vitro* activity was assessed on human enzymes and *in vivo* efficacy was demonstrated in a murine model, where species-related differences cannot be completely excluded, the overall findings strongly support the translational potential of compound **11**.

Overall, these results, together with a compatible *in vivo* pharmacokinetic profile, highlight the therapeutic potential of compound **11**, which proved to be worthy of consideration for its complete development as dual 5-LOX/sEH inhibitor with not only application in AP, but also in other acute and chronic inflammatory pathologies. Compound **11** also paves the way for a more robust and compete SAR study, exploring other different decorations to fine-tune the targeting of the proposed proteins.

## Experimental Section

### General

All the other reagents and solvents were purchased from Merck (Milan, Italy) or Fluorochem Limited (Derbyshire, England) unless otherwise stated. Reactions were performed under magnetic stirring in round-bottomed flasks unless otherwise noted. Moisture-sensitive reactions were conducted in oven-dried glassware under nitrogen stream, using freshly distilled solvents. TLC analysis of reaction mixtures was performed on precoated glass silica gel plates (F254, 0.25 mm, VWR International), while crude products were purified by the Isolera Spektra One automated flash chromatography system (Biotage, Uppsala, Sweden), using commercial silica gel cartridges (SNAP KP-Sil, Biotage). NMR spectra were recorded on a Bruker Avance 400 MHz apparatus, at room temperature. Chemical shifts were reported in δ values (ppm) relative to internal Me_4_Si for ^1^H and ^13^C NMR. J values were reported in hertz (Hz). ^1^H NMR peaks were described using the following abbreviations: s (singlet), d (doublet), t (triplet), and m (multiplet). HR-MS spectra were recorded by LTQ-Orbitrap-XL-ETD mass spectrometer (Thermo Scientific, Bremen, Germany), equipped with an ESI source. All the final compounds showed a purity ≥ 95% as assessed by RP-UHPLC-PDA analysis, performed using a Nexera UHPLC system (Shimadzu, Kyoto, Japan) consisting of a CBM-40 lite controller, two LC-40B X3 pumps, an SPD-M 40 photo diode array detector, a CTO-30A column oven and, a SIL-40C X3 autosampler. The chromatographic analysis was accomplished on a Kinetex Evo C18 column, 150 × 2.1 mm × 2.6 μm (Phenomenex, Bologna, Italy) maintained at 40 °C. The optimal mobile phase consisted of 0.1% HCOOH/H_2_O v/v (A) and 0.1% HCOOH/ACN v/v (B) delivered at constant flow rate of 0.3 mL/min^–1^. Analysis was performed in gradient elution as follows: 0–20.00 min, 5–95% B; 20.00–25.00 min, isocratic to 95% B; then five minutes for column re-equilibration. Data acquisition was set in the range 190–800 nm and chromatograms were monitored at 254 nm. Melting points (mp) were determined on a Stuart SMP30 melting point apparatus and are uncorrected.

#### General Procedure A: Alkylation and Acylation Reaction (**1, 5–10, 13, 14, 23, 27, 28, 32, 36**, and **37**)

6-Nitro-1,2,3,4-tetrahydroquinoline, 3-nitropyrrole, 6-nitro-1,2,3,4-tetrahydroisoquinoline, intermediate **4**, **26**, **31**, or **35** (0.1 mmol) was dissolved in acetonitrile and added with 2 equiv of potassium *tert*-butoxide and 2 equiv of the proper alkyl halide, Boc anhydride or methyl 4-(chlorocarbonyl)­benzoate. Afterward, the mixture was heated to 120 °C in a microwave reactor for 1 h, and, after completion of the reaction, it was quenched by 10% aqueous solution of citric acid and diluted with dichloromethane. The organic layer was separated, dried over anhydrous Na_2_SO_4_, filtered, and concentrated under vacuum. Flash chromatography of the crude mixture using *n*-hexane/ethyl acetate as mobile phase furnished the purified intermediates.

#### General Procedure B: Pd-Catalyzed Reduction and Deprotection Reaction (**2, 15, 16**, and **24**)

The starting material (1.0 mmol) dissolved in a mixture of THF/MeOH (1/:1 v/:v), was added with ammonium formate (10 equiv) and Pd/C (10% mol). The reaction mixture was mixed at 110 °C until the complete disappearance of the starting material, monitored by TLC. After completion, the reaction solution was filtered through Celite 503 (Merck Millipore, Burlington, USA) and evaporated under vacuum, furnishing the final compounds or the corresponding intermediates without further purification.

#### General Procedure C: Urea Formation (**3, 17–19, 25, 30**, and **34**)

A solution of **2**, **15**, **16**, **24**, 1-Boc-3-aminopyrrolidine or 4-amino-1-Boc-piperidine (0.1 mmol) in dichloromethane, was added with triphosgene (0.4 equiv), triethylamine (1.2 equiv) and the proper amine (1.2 equiv), and the mixture was stirred for 1 h at room temperature. After the completion of the reaction, monitored by TLC, water (30 mL) was added and the mixture was extracted with dichloromethane (3 × 10 mL); the organic layer was dried over Na_2_SO_4_, filtered and evaporated. Flash chromatography using *n*-hexane/ethyl acetate as mobile phase furnished the corresponding urea compounds.

#### General Procedure D: Boc Removal (**4, 22, 26, 31**, and **35**)

The N-Boc protected intermediates **3**, **19**, **25**, **30**, or **34** (1 mmol) were dissolved in a mixture of TFA/DCM (1/3, v/v), and triisopropylsilane (TIS, 0.1 equiv) was added. The mixture was stirred at room temperature for 3 h. Then, a solution of NaOH (2 N) was added until pH 7. The organic layer was evaporated under vacuum, diluted with ethyl acetate, extracted, dried over Na_2_SO_4_, filtered, and concentrated *in vacuo*. The final products were obtained by precipitation from dichloromethane and diethyl ether.

#### General Procedure E: Hydrolysis (**11, 12, 20, 21, 29**, and **33**)

Compound **6**, **10**, **17**, **18**, **27**, **32**, or **36** (0.5 mmol) was dissolved in methanol (5 mL) and added with 2 mL of NaOH 1 M aqueous solution. The reaction mixture was stirred at 100 °C until the complete disappearance of the starting material, evidenced by TLC. The organic phase was evaporated, and the aqueous phase was quenched with 20 mL of HCl 2 M, diluted with ethyl acetate and extracted. Then, the organic layer was filtered, dried over anhydrous Na_2_SO_4_ and evaporated *in vacuo* affording the final derivatives without further purification.

#### *tert*-Butyl 6-Nitro-3,4-dihydroquinoline-1­(2*H*)-carboxylate (**1**)

Synthesized from 6-nitro-1,2,3,4-tetrahydroquinoline and di-*tert*-butyl dicarbonate following the general procedure A. FC in *n*-hexane/ethyl acetate 9.5/0.5, R*f*: 0.47. Yellow powder (75% yield). ^1^H NMR (400 MHz, CDCl_3_): δ: 1.57 (s, 9H, C*H*_*3*_); 1.95–2.01 (m, 2H, C*H*_*2*_); 2.86 (t, 2H, C*H*_*2*_, *J* = 6.4 Hz); 3.78 (t, 2H, C*H*_*2*_, *J* = 6.1 Hz); 7.94–8.03 (m, 3H, aryl); HR-MS *m*/*z* calcd for C_14_H_18_N_2_O_4_ [(M + H)]^+^: 279.1339; found 279.1326.

#### *tert*-Butyl 6-Amino-3,4-dihydroquinoline-1­(2*H*)-carboxylate (**2**)

Synthesized from **1** following the general procedure B. FC in *n*-hexane/ethyl acetate 2/1, R*f*: 0.45. Whitish powder (94% yield). ^1^H NMR (400 MHz, CD_3_OD): δ: 1.51 (s, 9H, C*H*_*3*_); 1.85–1.91 (m, 2H, C*H*_*2*_); 2.68 (t, 2H, C*H*_*2*_, *J* = 6.6 Hz); 3.64 (t, 2H, C*H*_*2*_, *J* = 6.2 Hz); 6.51 (bs, 1H, aryl); 6.55 (dd, 1H, aryl, *J’* = 2.5 Hz and *J’’* = 8.6 Hz); 7.26 (d, 1H, aryl, *J* = 8.5 Hz); HR-MS *m*/*z* calcd for C_14_H_20_N_2_O_2_ [(M + H)]^+^: 249.1598; found 249.1566.

#### *tert*-Butyl 6-(3-(Adamantan-1-ylmethyl)­ureido)-3,4-dihydroquinoline-1­(2*H*)-carboxylate (**3**)

Obtained from **2** and adamantan-1-ylmethanamine according to the general procedure C. FC in *n*-hexane/ethyl acetate 3/2, R*f*: 0.43. Whitish oil (55% yield). ^1^H NMR (400 MHz, CD_3_OD): δ: 1.51 (s, 9H, C*H*_*3*_); 1.54 (s, 6H, C*H*_*2*_); 1.66 (d, 3H, C*H* and C*H*_*2*_, *J* = 11.8 Hz); 1.75 (d, 3H, C*H* and C*H*_*2*_, *J* = 11.9 Hz); 1.86–1.91 (m, 2H, C*H*_*2*_); 1.96 (bs, 3H, C*H* and C*H*_*2*_); 2.70 (t, 2H, C*H*_*2*_, *J* = 3.3 Hz); 2.89 (s, 2H, C*H*_*2*_); 3.62–3.66 (m, 2H, C*H*_*2*_); 6.06 (dd, 1H, aryl, *J’* = 2.2 Hz and *J’’* = 8.8 Hz); 7.16 (bs, 1H, aryl); 7.45 (d, 1H, aryl, *J* = 8.8 Hz); HR-MS *m*/*z* calcd for C_26_H_37_N_3_O_3_ [(M + H)]^+^: 440.2908; found 440.2968.

#### 1-(Adamantan-1-ylmethyl)-3-(1,2,3,4-tetrahydroquinolin-6-yl)­urea (**4**)

Intermediate **4** was synthesized from **3** applying the general procedure D. FC in *n*-hexane/ethyl acetate 0.5/9.5, R*f*: 0.42. Whitish powder (96% yield). ^1^H NMR (400 MHz, CD_3_OD): δ: 1.54 (s, 6H, C*H*_*2*_); 1.70 (d, 3H, C*H* and C*H*_*2*_, *J* = 11.6 Hz); 1.75 (d, 3H, C*H* and C*H*_*2*_, *J* = 11.9 Hz); 1.86–1.91 (m, 2H, C*H*_*2*_); 1.96 (bs, 3H, C*H* and C*H*_*2*_); 2.70 (t, 2H, C*H*_*2*_, *J* = 3.3 Hz); 2.89 (s, 2H, C*H*_*2*_); 3.62–3.66 (m, 2H, C*H*_*2*_); 6.06 (dd, 1H, aryl, *J’* = 2.2 Hz and *J’’* = 8.8 Hz); 7.16 (bs, 1H, aryl); 7.45 (d, 1H, aryl, *J* = 8.8 Hz); HR-MS *m*/*z* calcd for C_21_H_29_N_3_O [(M + H)]^+^: 340.2383; found 340.2398.

#### 1-(Adamantan-1-ylmethyl)-3-(1-benzyl-1,2,3,4-tetrahydroquinolin-6-yl)­urea (**5**)

Final derivative **5** was obtained from **4** and benzyl bromide according to the general procedure A. FC in dichloromethane/ethyl acetate 8/2, R*f*: 0.44. Whitish oil (44% yield). ^1^H NMR (400 MHz, (CD_3_)_2_SO): δ: 1.43 (s, 6H, C*H*_*2*_); 1.58 (d, 3H, C*H* and C*H*_*2*_, *J* = 11.3 Hz); 1.68 (d, 3H, C*H* and C*H*_*2*_, *J* = 11.8 Hz); 1.87–1.93 (m, 5H, C*H* and C*H*_*2*_); 2.69 (t, 2H, C*H*_*2*_, *J* = 6.4 Hz); 2.75 (d, 2H, C*H*_*2*_, *J* = 6.1 Hz); 3.28 (t, 2H, C*H*_*2*_, *J* = 5.5 Hz); 4.41 (s, 2H, C*H*_*2*_); 5.89 (t, 1H, N*H, J* = 5.8 Hz); 6.37 (d, 1H, aryl, *J* = 8.7 Hz); 6.81 (dd, 1H, aryl, *J’* = 2.3 and *J’’* = 8.7 Hz); 6.98 (d, 1H, aryl, *J* = 2.1 Hz); 7.20–7.25 (m, 3H, aryl); 7.29–7.33 (m, 2H, aryl); 7.86 (s, 1H, N*H*). ^13^C NMR (100 MHz, (CD_3_)_2_SO) δ: 22.5, 28.2, 28.25, 49.9, 51.2, 55.0, 111.6, 118.0, 120.3, 122.5, 127.0, 127.2, 128.8, 130.1, 139.7, 140.7, 156.3. HR-MS *m*/*z* calcd for C_28_H_35_N_3_O [(M + H)]^+^: 430.2853; found 430.2313.

#### Methyl 4-((6-(3-(Adamantan-1-ylmethyl)­ureido)-3,4-dihydroquinolin-1­(2*H*)-yl)­methyl)­benzoate (**6**)

Final derivative **6** was obtained from **4** and methyl 4-(bromomethyl)­benzoate applying the general procedure A. FC in dichloromethane/ethyl acetate 8/2, R*f*: 0.42. Whitish oil (41% yield). ^1^H NMR (400 MHz, CD_3_OD): δ: 1.51 (s, 6H, C*H*_*2*_); 1.67 (d, 3H, C*H* and C*H*_*2*_, *J* = 11.9 Hz); 1.76 (d, 3H, C*H* and C*H*_*2*_, *J* = 11.8 Hz); 1.96–2.03 (m, 5H, C*H* and C*H*_*2*_); 2.78 (t, 2H, C*H*_*2*_, *J* = 6.2 Hz); 2.85 (s, 2H, C*H*_*2*_); 3.32–3.35 (m, 2H, C*H*_*2*_); 3.89 (s, 3H, C*H*_*3*_); 4.50 (s, 2H, C*H*_*2*_); 6.36 (d, 1H, aryl, *J* = 8.8 Hz); 6.82 (dd, 1H, aryl, *J’* = 2.6 and *J’’* = 8.3 Hz); 6.94 (d, 1H, aryl, *J* = 2.4 Hz); 7.37 (d, 2H, aryl, *J* = 8.3 Hz); 7.95 (d, 2H, aryl, *J* = 7.8 Hz). ^13^C NMR (100 MHz, CD_3_OD) δ: 22.4, 27.9, 28.4, 33.6, 36.7, 39.9, 49.9, 51.1, 51.2, 54.9, 111.2, 120.2, 122.4, 122.8, 126.5, 128.1, 128.4, 129.4, 141.8, 145.4, 158.1, 167.1. HR-MS *m*/*z* calcd for C_30_H_37_N_3_O_3_ [(M + Na)]^+^: 510.2727; found 510.2776.

#### 1-(Adamantan-1-ylmethyl)-3-(1-(4-chlorobenzyl)-1,2,3,4-tetrahydroquinolin-6-yl)­urea (**7**)

Compound **7** was synthesized from **4** and 4-chlorobenzyl bromide according to the general procedure A. FC in dichloromethane/ethyl acetate 8/2, R*f* = 0.51. Whitish oil (40% yield). ^1^H NMR (400 MHz, (CD_3_)_2_SO): δ: 1.43 (s, 6H, C*H*_*2*_); 1.59 (d, 3H, C*H* and C*H*_*2*_, *J* = 11.6 Hz); 1.68 (d, 3H, C*H* and C*H*_*2*_, *J* = 11.9 Hz); 1.87–1.93 (m, 5H, C*H* and C*H*_*2*_); 2.68 (t, 2H, C*H*_*2*_, *J* = 6.4 Hz); 2.75 (d, 2H, C*H*_*2*_, *J* = 6.3 Hz); 3.27 (t, 2H, C*H*_*2*_, *J* = 5.3 Hz); 4.40 (s, 2H, C*H*_*2*_); 5.87 (t, 1H, N*H*, *J* = 5.8 Hz); 6.34 (d, 1H, aryl, *J* = 9.2 Hz); 6.82 (dd, 1H, aryl, *J’* = 2.1 and *J’’* = 8.5 Hz); 6.99 (d, 1H, aryl, *J* = 2.2 Hz); 7.26 (d, 2H, aryl, *J* = 8.5 Hz); 7.36 (d, 2H, aryl, *J* = 8.7 Hz); 7.84 (s, 1H, N*H*). ^13^C NMR (100 MHz, (CD_3_)_2_SO) δ: 22.5, 28.2, 34.0, 37.1, 49.9, 51.2, 54.4, 111.6, 118.0, 120.3, 122.6, 128.8, 129.1, 130.2, 131.5, 138.8, 140.5, 156.3. HR-MS *m*/*z* calcd for C_28_H_34_ClN_3_O [(M + Na)]^+^: 486.2283; found 486.2351.

#### 1-(Adamantan-1-ylmethyl)-3-(1-(4-methoxybenzyl)-1,2,3,4-tetrahydroquinolin-6-yl)­urea (**8**)

Derivative **8** was obtained from **4** and 4-methoxybenzyl bromide following the general procedure A. FC in dichloromethane/ethyl acetate 9/1, R*f* = 0.48. Whitish oil (48% yield). ^1^H NMR (400 MHz, (CD_3_)_2_SO): δ: 1.43 (s, 6H, C*H*_*2*_); 1.59 (d, 3H, C*H* and C*H*_*2*_, *J* = 11.6 Hz); 1.68 (d, 3H, C*H* and C*H*_*2*_, *J* = 11.9 Hz); 1.85–1.94 (m, 5H, C*H* and C*H*_*2*_); 2.67 (t, 2H, C*H*_*2*_, *J* = 6.5 Hz); 2.75 (d, 2H, C*H*_*2*_, *J* = 6.3 Hz); 3.24 (t, 2H, C*H*_*2*_, *J* = 5.0 Hz); 3.72 (s, 3H, C*H*_*3*_); 4.33 (s, 2H, C*H*_*2*_); 5.90 (t, 1H, N*H*, *J* = 6.1 Hz); 6.41 (d, 1H, aryl, *J* = 8.8 Hz); 6.83 (dd, 1H, aryl, *J’* = 2.3 and *J’’* = 8.5 Hz); 6.87 (d, 2H, aryl, *J* = 8.5 Hz); 6.96 (d, 1H, aryl, *J* = 2.2 Hz); 7.16 (d, 2H, aryl, *J* = 8.6 Hz); 7.86 (s, 1H, N*H*). ^13^C NMR (100 MHz, (CD_3_)_2_SO) δ: 22.5, 28.2, 28.25, 34.0, 37.1, 49.7, 51.2, 54.4, 55.5, 111.8, 114.3, 118.0, 120.3, 122.5, 128.5, 130.0, 131.3, 140.8, 156.3, 158.5. HR-MS *m*/*z* calcd for C_29_H_37_N_3_O_2_ [(M + H)]^+^: 460.2959; found 460.3022.

#### 1-(Adamantan-1-ylmethyl)-3-(1-(4-isopropylbenzyl)-1,2,3,4-tetrahydroquinolin-6-yl)­urea (**9**)

Synthesized from **4** and 4-isopropylbenzyl bromide applying the general procedure A. FC in dichloromethane/ethyl acetate 9/1, R*f* = 0.50. Whitish oil (47% yield). ^1^H NMR (400 MHz, (CD_3_)_2_SO): δ: 1.61 (d, 6H, C*H*_*3*_, *J* = 7.0 Hz); 1.43 (s, 6H, C*H*_*2*_); 1.58 (d, 3H, C*H* and C*H*_*2*_, *J* = 11.6 Hz); 1.68 (d, 3H, C*H* and C*H*_*2*_, *J* = 11.9 Hz); 1.86–1.93 (m, 5H, C*H* and C*H*_*2*_); 2.68 (t, 2H, C*H*_*2*_, *J* = 6.2 Hz); 2.75 (d, 2H, C*H*_*2*_, *J* = 6.0 Hz); 2.81–2.88 (m, 1H, C*H*); 3.26 (t, 2H, C*H*_*2*_, *J* = 5.6 Hz); 4.37 (s, 2H, C*H*_*2*_); 5.90 (t, 1H, N*H*, *J* = 6.0 Hz); 6.39 (d, 1H, aryl, *J* = 8.5 Hz); 6.82 (dd, 1H, aryl, *J’* = 2.3 and *J’’* = 8.7 Hz); 6.97 (d, 1H, aryl, *J* = 2.3 Hz); 7.16 (dd, 4H, aryl, *J’* = 8.3 and *J’’* = 10.8 Hz); 7.86 (s, 1H, N*H*). ^13^C NMR (100 MHz, (CD_3_)_2_SO): δ: 22.5, 24.4, 28.2, 28.3, 33.5, 34.0, 37.1, 49.8, 51.2, 54.6, 111.6, 118.1, 120.3, 122.4, 126.7, 127.3, 130.0, 136.9, 140.8, 147.1, 156.3. HR-MS *m*/*z* calcd for C_31_H_41_N_3_O [(M + H)]^+^: 472.3322; found 472.3312.

#### Methyl 4-(6-(3-(Adamantan-1-ylmethyl)­ureido)-1,2,3,4-tetrahydroquinoline-1-carbonyl)­benzoate (**10**)

For the synthesis of intermediate **10**, **4** (0.4 mmol) was dissolved in tetrahydrofuran and added with TEA (1.2 equiv) and methyl 4-(chlorocarbonyl)­benzoate (1.2 equiv). The mixture was stirred at room temperature for 30 min; afterward, an aqueous solution of K_2_CO_3_ 2 M and dichloromethane were added. The organic phase was extracted twice, dried over Na_2_SO_4_, filtered and evaporated *in vacuo*. The obtained crude was purified by flash chromatography, employing *n*-hexane/ethyl acetate (*ratio* 7/3) as mobile phase. R*f* = 0.46. Whitish oil (50% yield). ^1^H NMR (400 MHz, CD_3_OD): δ: 1.54 (s, 6H, C*H*_*2*_); 1.68 (d, 3H, C*H* and C*H*_*2*_, *J* = 11.9 Hz); 1.77 (d, 3H, C*H* and C*H*_*2*_, *J* = 12.1 Hz); 1.98 (bs, 3H, C*H* and C*H*_*2*_); 2.03–2.08 (m, 2H, C*H*_*2*_); 2.83 (d, 2H, C*H*_*2*_, *J* = 6.0 Hz); 2.86–2.91 (m, 2H, C*H*_*2*_); 3.87 (t, 2H, C*H*_*2*_, *J* = 5.5 Hz); 3.91 (s, 3H, C*H*_*3*_); 6.36 (d, 1H, aryl, *J* = 8.7 Hz); 6.83 (d, 1H, aryl, *J* = 6.3 Hz); 7.32 (d, 1H, aryl, *J* = 2.1 Hz); 7.44 (d, 1H, aryl, *J* = 7.6 Hz); 7.97 (d, 1H, aryl, *J* = 7.8 Hz); HR-MS *m*/*z* calcd for C_30_H_35_N_3_O_4_ [(M + H)]^+^: 502.2700; found 502.2678.

#### 4-((6-(3-(Adamantan-1-ylmethyl)­ureido)-3,4-dihydroquinolin-1­(2*H*)-yl)­methyl)­benzoic Acid (**11**)

Obtained from **6** applying the general procedure E. Precipitated from MeOH/diethyl ether. White powder (92% yield); mp 220 °C (decomp.). ^1^H NMR (400 MHz, CD_3_OD): δ: 1.53 (s, 6H, C*H*_*2*_); 1.69 (d, 3H, C*H* and C*H*_*2*_, *J* = 11.7 Hz); 1.77 (d, 3H, C*H* and C*H*_*2*_, *J* = 12.0 Hz); 1.98–2.04 (m, 4H, C*H*_*2*_); 2.80 (bs, 2H, C*H*_*2*_); 2.87 (s, 2H, C*H*_*2*_); 4.53 (s, 2H, C*H*_*2*_); 6.41 (d, 1H, aryl, *J* = 8.1 Hz); 6.83 (d, 1H, aryl, *J* = 8.5 Hz); 6.95 (s, 1H, aryl); 7.37 (d, 2H, aryl, *J* = 8.2 Hz); 7.97 (d, 1H, aryl, *J* = 8.1 Hz). ^13^C NMR (100 MHz, CD_3_OD): δ: 22.2, 28.4, 36.7, 39.9, 51.2, 54.9, 111.3, 120.4, 122.6, 126.1, 126.3, 127.8, 129.4, 129.6, 142.0, 144.7, 169.1. HR-MS *m*/*z* calcd for C_29_H_35_N_3_O_3_ [(M + H)]^+^: 474.2751; found 474.2742.

#### 1-(Adamantan-1-ylmethyl)-3-(1-(4-isopropylbenzyl)-1,2,3,4-tetrahydroquinolin-6-yl)­urea (**12**)

Final derivative **12** was obtained from **10** applying the general procedure E. Precipitated from MeOH/diethyl ether. Whitish powder (93% yield); mp 205 °C (decomp.). ^1^H NMR (400 MHz, (CD_3_)_2_SO): δ: 1.43 (s, 6H, C*H*_*2*_); 1.58 (d, 3H, C*H* and C*H*_*2*_, *J* = 11.6 Hz); 1.68 (d, 3H, C*H* and C*H*_*2*_, *J* = 11.9 Hz); 1.91–1.97 (m, 5H, C*H* and C*H*_*2*_); 2.75–2.78 (m, 4H, C*H*_*2*_); 3.72 (t, 2H, C*H*_*2*_, *J* = 6.6 Hz); 6.13 (t, 1H, N*H*, *J* = 6.9 Hz); 6.56 (bs, 1H, aryl); 7.35 (d, 1H, aryl, *J* = 2.7 Hz); 7.41 (d, 2H, aryl, *J* = 7.5 Hz); 7.88 (d, 2H, aryl, *J* = 7.7 Hz); 8.35 (s, 1H, N*H*). ^13^C NMR (100 MHz, (CD_3_)_2_SO): δ: 24.1, 27.1, 28.2, 34.0, 37.1, 51.2, 115.1, 117.2, 125.6, 128.0, 129.2, 132.3, 138.1, 156.1, 168.6, 169.0. HR-MS *m*/*z* calcd for C_31_H_41_N_3_O [(M + K)]^+^: 510.2881; found 510.2404.

#### Methyl 4-((3-Nitro-1*H*-pyrrol-1-yl)­methyl)­benzoate (**13**)

Synthesized from 3-nitropyrrole and methyl 4-(bromomethyl)­benzoate following the general procedure A. FC in *n*-hexane/dichloromethane 9/1, R*f* = 0.41. Yellowish oil (45% yield). ^1^H NMR (400 MHz, CDCl_3_): δ: 3.95 (s, 3H, *CH*_*3*_); 5.15 (s, 2H, *CH*_*2*_); 6.61 (t, 1H, aryl, *J* = 2.8 Hz); 6.79–6.80 (m, 1H, aryl); 7.25 (d, 2H, aryl, *J* = 8.2 Hz); 7.56 (t, 1H, aryl, *J* = 1.9 Hz); 8.07 (d, 1H, aryl, *J* = 8.2 Hz); HR-MS *m*/*z* calcd for C_13_H_12_N_2_O_4_ [(M + H)]^+^: 261.0870; found 261.0845.

#### *tert*-Butyl (4-(3-Nitro-1*H*-pyrrol-1-yl)­butyl)­carbamate (**14**)

Obtained from 3-nitropyrrole and *tert*-butyl (4-bromobutyl)­carbamate following the general procedure A. FC in *n*-hexane/ethyl acetate 1/2, R*f* = 0.40. Yellowish oil (38% yield). ^1^H NMR (400 MHz, CDCl_3_): δ: 1.44 (s, 9H, *CH*_*3*_); 1.46–1.52 (m, 2H, C*H*_*2*_); 1.79–1.82 (m, 2H, C*H*_*2*_); 3.13–3.17 (m, 2H, C*H*_*2*_); 3.94 (t, 2H, C*H*_*2*_, *J* = 7.1 Hz); 4.66 (bs, 1H, *NH*); 6.57 (t, 1H, aryl, *J* = 2.8 Hz); 6.69–6.70 (m, 1H, aryl); 7.51 (t, 1H, aryl, *J* = 2.8 Hz); HR-MS *m*/*z* calcd for C_13_H_21_N_3_O_4_ [(M + H)]^+^: 284.1605; found 284.1573.

#### Methyl 4-((3-Amino-1*H*-pyrrol-1-yl)­methyl)­benzoate (**15**)

Synthesized from **13** following the general procedure B. FC in *n*-hexane/ethyl acetate 1/1, R*f* = 0.43. Whitish powder (92% yield). ^1^H NMR (400 MHz, CDCl_3_): δ: 3.90 (s, 3H, C*H*_*3*_); 5.05 (s, 2H, *CH*_*2*_); 5.81 (d, 1H, aryl, *J* = 2.6 Hz); 6.53 (d, 1H, aryl, *J* = 2.7 Hz); 7.21 (d, 2H, aryl, *J* = 8.2 Hz); 7.95 (d, 2H, aryl, *J* = 8.3 Hz); 7.99–8.04 (m, 1H, aryl); HR-MS *m*/*z* calcd for C_13_H_14_N_2_O_2_ [(M + H)]^+^: 231.1128; found 231.1099.

#### *tert*-Butyl (4-(3-Amino-1*H*-pyrrol-1-yl)­butyl)­carbamate (**16**)

Synthesized from **14** following the general procedure B. FC in *n*-hexane/ethyl acetate 1/1, R*f* = 0.42. Whitish powder (93% yield). ^1^H NMR (400 MHz, CDCl_3_): δ: 1.53 (s, 9H, C*H*_*3*_); 1.44–1.50 (m, 2H, C*H*_*2*_); 1.78–1.80 (m, 2H, C*H*_*2*_); 3.09–3.14 (m, 2H, C*H*_*2*_); 3.88 (t, 2H, C*H*_*2*_, *J* = 7.1 Hz); 5.76 (bs, 1H, N*H*); 5.99 (t, 1H, aryl, *J* = 2.2 Hz); 6.56 (t, 1H, aryl, *J* = 2.5 Hz); 6.63 (bs, 1H, aryl); HR-MS *m*/*z* calcd for C_13_H_23_N_3_O_2_ [(M + H)]^+^: 254.1863; found 254.1847.

#### Methyl 4-((3-(3-(Cyclohexylmethyl)­ureido)-1*H*-pyrrol-1-yl)­methyl)­benzoate (**17**)

Synthesized from **15** and cyclohexanemethylamine following the general procedure C. FC in dichloromethane/ethyl acetate 3/2, R*f* = 0.45. Whitish powder (32% yield). ^1^H NMR (400 MHz, CDCl_3_): δ: 0.89–0.98 (m, 2H, *CH*_*2*_); 1.18–1.31 (m, 4H, *CH*_*2*_); 1.68–1.76 (m, 5H, *CH* and *CH*_*2*_); 3.01 (d, 2H, *CH*_*2*_, *J* = 6.6 Hz); 3.90 (s, 3H, *CH*_*3*_); 5.12 (s, 2H, *CH*_*2*_); 5.97–5.98 (m, 1H, aryl); 6.64 (t, 1H, aryl, *J* = 2.4 Hz); 6.78 (t, 1H, aryl, *J* = 1.9 Hz); 7.26 (d, 2H, aryl, *J* = 8.2 Hz); 7.97 (d, 2H, aryl, *J* = 8.3 Hz); HR-MS *m*/*z* calcd for C_21_H_27_N_3_O_3_ [(M + H)]^+^: 370.2125; found 370.2099.

#### Methyl 4-((3-(3-(((3r,5r,7r)-Adamantan-1-yl)­methyl)­ureido)-1*H*-pyrrol-1-yl)­methyl)­benzoate (**18**)

Synthesized from **15** and adamantan-1-yl-methanamine following the general procedure C. FC in dichloromethane/ethyl acetate 1/1, R*f* = 0.44. Whitish powder (36% yield). ^1^H NMR (400 MHz, CDCl_3_): δ: 1.50 (s, 6H, C*H*_*2*_); 1.67 (d, 3H, C*H* and C*H*_*2*_, *J* = 11.7 Hz); 1.76 (d, 3H, C*H* and C*H*_*2*_, *J* = 12 Hz); 1.96 (bs, 3H, C*H* and C*H*_*2*_); 2.86 (s, 2H, C*H*_*2*_); 3.90 (s, 3H, C*H*_*3*_); 5.12 (s, 2H, C*H*_*2*_); 5.98 (t, 1H, aryl, *J* = 2.4 Hz); 6.66 (t, 1H, aryl, *J* = 2.2 Hz); 6.64 (t, 1H, aryl, *J* = 2.4 Hz); 6.78 (t, 1H, aryl, *J* = 1.9 Hz); 7.26 (d, 2H, aryl, *J* = 8.2 Hz); 7.97 (d, 2H, aryl, *J* = 8.3 Hz); HR-MS *m*/*z* calcd for C_25_H_31_N_3_O_3_ [(M + H)]^+^: 422.2438; found 422.2402.

#### *tert*-Butyl (4-(3-(3-(((3r,5r,7r)-Adamantan-1-yl)­methyl)­ureido)-1*H*-pyrrol-1-yl)­butyl)­carbamate (**19**)

Synthesized from **16** and adamantan-1-yl-methanamine following the general procedure C. FC dichloromethane/ethyl acetate 1/1, R*f* = 0.42. Whitish powder (38% yield). ^1^H NMR (400 MHz, CDCl_3_): δ: 1.46 (s, 9H, C*H*_*3*_); 1.50 (s, 6H, C*H*_*2*_); 1.60–1.65 (m, 5H, *CH* and *CH*_*2*_); 1.72 (d, 3H, C*H* and C*H*_*2*_, *J* = 11.4 Hz); 1.79 (t, 2H, C*H*_*2*_, *J* = 7.7 Hz); 1.97 (bs, 3H, C*H* and C*H*_*2*_); 2.92 (d, 2H, C*H*_*2*_, *J* = 6.2 Hz); 3.11–3.15 (m, 2H, *CH*_*2*_); 3.76 (s, 1H, N*H*); 3.86 (t, 2H, *CH*_*2*_, *J* = 7 Hz); 4.57 (bs, 1H, N*H*); 4.95 (t, 1H, N*H*, *J* = 8); 5.99 (t, 1H, aryl, *J* = 2.2); 6.56 (t, 1H, aryl, *J* = 2.5 Hz); 6.64 (bs, 1H, aryl); HR-MS *m*/*z* calcd for C_25_H_40_N_4_O_3_ [(M + H)]^+^: 445.3173; found 445.3449.

#### 4-((3-(3-(Cyclohexylmethyl)­ureido)-1*H*-pyrrol-1-yl)­methyl)­benzoic Acid (**20**)

Synthesized from **17** following the general procedure E. Precipitated from MeOH/diethyl ether. White powder (95% yield); mp 210 °C (decomp.). ^1^H NMR (400 MHz, (CD_3_)_2_SO): δ: 0.80–0.89 (m, 2H, C*H*_*2*_); 1.09–1.22 (m, 3H, C*H* and C*H*_*2*_); 1.29–1.36 (m, 1H, C*H*); 1.60–1.65 (m, 5H, C*H* and C*H*_*2*_); 2.87 (t, 2H, C*H*_*2*_, *J* = 6.2 Hz); 5.06 (s, 2H, C*H*_*2*_); 5.81–5.84 (m, 2H, N*H* and aryl); 6.64 (t, 1H, aryl, *J* = 2.6 Hz); 6.79 (t, 1H, aryl, *J* = 1.8 Hz); 7.24 (d, 2H, aryl, *J* = 8.2 Hz); 7.82 (s, 1H, N*H*); 7.89 (d, 2H, aryl, *J* = 8.2 Hz). ^13^C NMR (100 MHz, (CD_3_)_2_SO): δ: 25.9, 26.6, 30.8, 38.5, 45.9, 52.5, 100.9, 109.7, 119.5, 125.3, 127.5, 130.0, 130.2, 144.7, 155.8, 167.5. HR-MS *m*/*z* calcd for C_20_H_25_N_3_O_3_ [(M + H)]^+^: 356.1969; found 356.1966.

#### 4-((3-(3-(Adamantan-1-ylmethyl)­ureido)-1*H*-pyrrol-1-yl)­methyl)­benzoic Acid (**21**)

Obtained from **18** applying the general procedure E. Precipitated from MeOH/diethyl ether. White powder (93% yield); mp 212 °C (decomp.). ^1^H NMR (400 MHz, (CD_3_)_2_SO): δ: 1.41 (s, 6H, C*H*_*2*_); 1.58 (d, 3H, C*H* and C*H*_*2*_, *J* = 11.6 Hz); 1.66 (d, 3H, C*H* and C*H*_*2*_, *J* = 11.8 Hz); 1.92 (bs, 3H, C*H* and C*H*_*2*_); 2.74 (d, 2H, C*H*_*2*_, *J* = 6.0 Hz); 5.02 (s, 2H, C*H*_*2*_); 5.80 (d, 1H, aryl, *J* = 2.5 Hz); 5.85 (t, 1H, N*H*, *J* = 6.0 Hz); 6.62 (d, 1H, aryl, *J* = 2.6 Hz); 6.78 (s, 1H, N*H*); 7.16 (d, 2H, aryl, *J* = 8.0 Hz); 7.85 (d, 2H, aryl, *J* = 8.0 Hz); 7.91 (s, 1H, aryl). ^13^C NMR (100 MHz, (CD_3_)_2_SO): δ: 28.2, 34.0, 37.1, 51.3, 52.7, 100.7, 109.6, 119.3, 125.3, 127.0, 129.8, 156.0, 168.4. HR-MS *m*/*z* calcd for C_24_H_29_N_3_O_3_ [(M + H)]^+^: 408.2282; found 408.2275.

#### 1-(Adamantan-1-ylmethyl)-3-(1-(4-aminobutyl)-1*H*-pyrrol-3-yl)­urea (**22**)

Obtained from **19** according to the general procedure D. Precipitated from MeOH/diethyl ether. White powder (96% yield); mp 74.5 °C. ^1^H NMR (400 MHz, CD_3_OD): δ: 1.54 (s, 6H, C*H*_*2*_); 1.59–1.71 (m, 5H, C*H* and C*H*_*2*_); 1.77–1.87 (m, 5H, C*H* and C*H*_*2*_); 1.99 (bs, 3H, C*H* and C*H*_*2*_); 2.83–2.88 (m, 4H, C*H*_*2*_); 3.90 (t, 2H, C*H*_*2*_, *J* = 6.5 Hz); 5.80 (d, 1H, aryl, *J* = 2.5 Hz); 5.87 (d, 1H, aryl, *J* = 6.0 Hz); 6.56 (d, 1H, aryl, *J* = 2.7 Hz). ^13^C NMR (100 MHz, CD_3_OD): δ: 24.4, 27.8, 28.4, 36.7, 38.9, 39.9, 48.5, 51.3, 101.6, 118.6, 123.1, 158.1. HR-MS *m*/*z* calcd for C_20_H_32_N_4_O [(M + H)]^+^: 345.2649; found 345.2632.

#### *tert*-Butyl 6-Nitro-3,4-dihydroisoquinoline-2­(1*H*)-carboxylate (**23**)

Synthesized from 6-nitro-1,2,3,4-tetrahydroisoquinoline and di-*tert*-butyl dicarbonate following the general procedure A. FC in *n*-hexane/ethyl acetate 8/2, R*f* = 0.45. Yellowish powder (83% yield). ^1^H NMR (400 MHz, CDCl_3_): δ: 1.50 (s, 9H, C*H*_*3*_); 2.93 (t, 2H, C*H*_*2*_, *J* = 11.3 Hz); 3.69 (t, 2H, C*H*_*2*_, *J* = 5.8 Hz); 4.66 (s, 2H, C*H*_*2*_); 7.26 (d, 1H, aryl, *J* = 9.1 Hz); 8.01 (s, 1H, aryl); 8.03 (bs, 1H, aryl); HR-MS *m*/*z* calcd for C_14_H_18_N_2_O_4_ [(M + H)]^+^: 279.1339; found 279.1325.

#### *tert*-Butyl 6-Amino-3,4-dihydroisoquinoline-2­(1*H*)-carboxylate (**24**)

Obtained from **23** applying the general procedure B. FC in *n*-hexane/ethyl acetate 2/1, R*f* = 0.43. Whitish powder (93% yield). ^1^H NMR (400 MHz, CD_3_OD): δ: 1.51 (s, 9H, C*H*_*3*_); 2.75 (t, 2H, C*H*_*2*_, *J* = 5.4 Hz); 3.62 (bs, 2H, C*H*_*2*_); 4.48 (s, 2H, C*H*_*2*_); 6.49 (bs, 1H, aryl); 6.55 (dd, 1H, aryl, *J’* = 1.8 Hz and *J’’* = 8.1 Hz); 6.91 (d, 1H, aryl, *J* = 8.0 Hz); HR-MS *m*/*z* calcd for C_14_H_20_N_2_O_2_ [(M + H)]^+^: 249.1598; found 249.1563.

#### *tert*-Butyl 6-(3-(Adamantan-1-ylmethyl)­ureido)-3,4-dihydroisoquinoline-2­(1*H*)-carboxylate (**25**)

Synthesized from **24** and adamantan-1-ylmethanamine following the general procedure C. FC in *n*-hexane/ethyl acetate 1/2, R*f* = 0.44. Whitish oil (48% yield). ^1^H NMR (400 MHz, CD_3_OD): δ: 1.56 (s, 9H, C*H*_*3*_); 1.61 (bs, 6H, C*H*_*2*_); 1.76 (d, 3H, C*H* and C*H*_*2*_, *J* = 11.5 Hz); 1.84 (d, 3H, C*H* and C*H*_*2*_, *J* = 12.1 Hz); 2.05 (bs, 3H, C*H* and C*H*_*2*_); 2.85 (t, 2H, C*H*_*2*_, *J* = 5.8 Hz); 2.95 (s, 2H, C*H*_*2*_); 4.54 (s, 2H, C*H*_*2*_); 7.07 (d, 1H, aryl, *J* = 8.3 Hz); 7.20 (d, 1H, aryl, *J* = 6.5 Hz); 7.27 (bs, 1H, aryl); HR-MS *m*/*z* calcd for C_26_H_37_N_3_O_3_ [(M + H)]^+^: 440.2908; found 440.2875.

#### 1-(Adamantan-1-ylmethyl)-3-(1,2,3,4-tetrahydroisoquinolin-6-yl)­urea (**26**)

Synthesized from **25** following the general procedure D. Whitish powder (93% yield). ^1^H NMR (400 MHz, CD_3_OD): δ: 1.54 (bs, 6H, C*H*_*2*_); 1.68 (d, 3H, C*H* and C*H*_*2*_, *J* = 10.9 Hz); 1.76 (d, 3H, C*H* and C*H*_*2*_, *J* = 12 Hz); 1.97 (bs, 3H, C*H* and C*H*_*2*_); 3.06 (t, 2H, C*H*_*2*_, *J* = 6.2 Hz); 3.45 (t, 2H, C*H*_*2*_, *J* = 6.3 Hz); 4.26 (s, 2H, C*H*_*2*_); 7.07 (d, 1H, aryl, *J* = 8.4 Hz); 7.23 (dd, 1H, aryl, *J’* = 2.0 Hz and *J’’* = 8.4 Hz); 7.32 (bs, 1H, aryl); HR-MS *m*/*z* calcd for C_21_H_29_N_3_O [(M + H)]^+^: 340.2383; found 340.2356.

#### Methyl 4-((6-(3-(Adamantan-1-ylmethyl)­ureido)-3,4-dihydroisoquinolin-2­(1*H*)-yl)­methyl)­benzoate (**27**)

Synthesized from **26** and methyl 4-(bromomethyl)­benzoate following the general procedure A. FC in *n*-hexane/ethyl acetate 1/1, R*f* = 0.44. Whitish powder (47% yield). ^1^H NMR (400 MHz, CD_3_OD): δ: 1.56 (bs, 6H, C*H*_*2*_); 1.70 (d, 3H, C*H* and C*H*_*2*_, *J* = 11.8 Hz); 1.79 (d, 3H, C*H* and C*H*_*2*_, *J* = 12.1 Hz); 1.99 (bs, 3H, C*H* and C*H*_*2*_); 2.90 (s, 2H, C*H*_*2*_); 2.95 (s, 2H, *CH*_*2*_); 3.76 (s, 2H, *CH*_*2*_); 3.93 (s, 2H, *CH*_*2*_); 3.95 (s, 2H, *CH*_*2*_); 6.94 (d, 1H, aryl, *J* = 8.3 Hz); 7.12 (dd, 1H, aryl, *J’* = 2.1 Hz and *J’’* = 8.3 Hz); 7.22 (d, 1H, aryl, *J* = 1.8 Hz); 7.58 (d, 1H, aryl, *J* = 8.2 Hz); 8.06 (d, 1H, aryl, *J* = 8.2 Hz); HR-MS *m*/*z* calcd for C_30_H_37_N_3_O3 [(M + H)]^+^: 488.2908; found 488.2889.

#### 1-(Adamantan-1-ylmethyl)-3-(2-(4-chlorobenzyl)-1,2,3,4-tetrahydroisoquinolin-6-yl)­urea (**28**)

Derivative **28** was obtained from **26** and 4-chlorobenzyl bromide, following the general procedure A. FC in *n*-hexane/ethyl acetate 1/1, R*f* = 0.47. Whitish oil (50% yield). ^1^H NMR (400 MHz, (CD_3_)_2_SO): δ: 1.44 (s, 6H, C*H*_*2*_); 1.58 (d, 3H, C*H* and C*H*_*2*_, *J* = 11.4 Hz); 1.68 (d, 3H, C*H* and C*H*_*2*_, *J* = 11.9 Hz); 1.93 (bs, 3H, C*H* and C*H*_*2*_); 2.62 (t, 2H, C*H*_*2*_, *J* = 5.8 Hz); 2.74 (t, 2H, C*H*_*2*_, *J* = 15.3 Hz); 3.18 (d, 2H, C*H*_*2*_, *J* = 5.6 Hz); 3.43 (s, 2H, C*H*_*2*_); 3.60 (s, 2H, C*H*_*2*_); 6.05 (d, 1H, N*H*, *J* = 6.1 Hz); 6.83 (d, 1H, aryl, *J* = 8.7 Hz); 7.05 (dd, 1H, aryl, *J’* = 1.6 and *J’’* = 8.1 Hz); 7.18 (s, 1H, aryl); 7.36–7.40 (m, 4H, aryl); 8.23 (s, 1H, N*H*). ^13^C NMR (100 MHz, (CD_3_)_2_SO): δ: 28.2, 29.4, 33.9, 37.1, 50.7, 51.2, 55.4, 61.4, 115.8, 116.1, 117.5, 126.9, 127.7, 128.7, 131.0, 131.9, 134.7, 139.0, 155.9. HR-MS *m*/*z* calcd for C_28_H_34_ClN_3_O [(M + H)]^+^: 464.2463; found 464.2436.

#### 4-((6-(3-(Adamantan-1-ylmethyl)­ureido)-3,4-dihydroisoquinolin-2­(1*H*)-yl)­methyl)­benzoic Acid (**29**)

Derivative **29** was obtained from **27**, according to the general procedure E. Precipitated from MeOH/diethyl ether. Whitish powder (92% yield); mp 250 °C (decomp.). ^1^H NMR (400 MHz, (CD_3_)_2_SO): δ: 1.45 (s, 6H, C*H*_*2*_); 1.59 (d, 3H, C*H* and C*H*_*2*_, *J* = 11.3 Hz); 1.67 (d, 3H, C*H* and C*H*_*2*_, *J* = 11.7 Hz); 1.93 (bs, 3H, C*H* and C*H*_*2*_); 2.77 (d, 2H, C*H*_*2*_, *J* = 6.0 Hz); 2.94 (d, 1H, C*H*, *J* = 14.3 Hz); 3.18–3.30 (m, 2H, C*H*_*2*_); 3.57–3.63 (m, 1H, C*H*); 4.12–4.23 (m, 2H, C*H*_*2*_); 4.45–4.55 (m, 2H, C*H*_*2*_); 6.68 (d, 1H, N*H*, *J* = 12.1 Hz); 6.99 (d, 1H, aryl, *J* = 6.8 Hz); 7.21 (d, 1H, aryl, *J* = 8.4 Hz); 7.33 (s, 1H, aryl); 7.81–7.83 (m, 2H, aryl); 8.01 (d, 2H, aryl, *J* = 8.0 Hz); 9.30 (d, 1H, N*H*, *J* = 16.0 Hz). ^13^C NMR (100 MHz, (CD_3_)_2_SO): δ: 25.4, 28.2, 33.9, 37.0, 51.2, 57.9, 116.3, 120.3, 127.3, 130.0, 132.1, 135.0, 140.9, 156.1, 167.3. HR-MS *m*/*z* calcd for C_29_H_35_N_3_O_3_ [(M + H)]^+^: 474.2751; found 474.2820.

#### *tert*-Butyl 3-(3-(Adamantan-1-ylmethyl)­ureido)­pyrrolidine-1-carboxylate (**30**)

Synthesized from 1-Boc-3-aminopyrrolidine and adamantan-1-ylmethanamine following the general procedure C. FC in dichloromethane/ethyl acetate 9/1, R*f* = 0.44. Whitish oil (78% yield). ^1^H NMR (400 MHz, CD_3_OD): δ: 1.48 (s, 9H, C*H*_*3*_); 1.52 (bs, 6H, C*H*_*2*_); 1.69 (d, 3H, C*H* and C*H*_*2*_, *J* = 11.9 Hz); 1.78 (d, 4H, C*H* and C*H*_*2*_, *J* = 12.4 Hz); 1.98 (bs, 3H, C*H* and C*H*_*2*_); 2.11–2.15 (m, 1H, *CH*); 2.83 (s, 2H, C*H*_*2*_); 3.15–3.20 (m, 1H, *CH*); 3.39–3.45 (m, 2H, *CH*_*2*_); 3.53–3.57 (m, 1H, *CH*); 4.18–4.22 (m, 1H, *CH*); HR-MS *m*/*z* calcd for C_21_H_35_N_3_O_3_ [(M + H)]^+^: 378.2751; found 378.2745.

#### 1-(Adamantan-1-ylmethyl)-3-(pyrrolidin-3-yl)­urea (**31**)

Synthesized from **30** following the general procedure D. Precipitated from MeOH/diethyl ether. Whitish powder (94% yield). ^1^H NMR (400 MHz, CD_3_OD): δ: 1.50 (bs, 6H, C*H*_*2*_); 1.66 (d, 3H, C*H* and C*H*_*2*_, *J* = 11.5 Hz); 1.75 (d, 3H, C*H* and C*H*_*2*_, *J* = 12.1 Hz); 1.96 (bs, 3H, C*H* and C*H*_*2*_); 2.01–2.06 (m, 1H, *CH*); 2.26–2.34 (m, 1H, *CH*); 2.79–2.83 (m, 2H, *CH*_*2*_); 3.21–3.27 (m, 1H, *CH*); 3.32–3.38 (m, 1H, *CH*); 3.43–3.50 (m, 2H, *CH*_*2*_); 4.32–4.38 (m, 1H, *CH*); HR-MS *m*/*z* calcd for C_16_H_27_N_3_O [(M + H)]^+^: 278.2227; found 278.2196.

#### Methyl 4-((3-(3-(Adamantan-1-ylmethyl)­ureido)­pyrrolidin-1-yl)­methyl)­benzoate (**32**)

Derivative **32** was obtained from **31** and methyl 4-(bromomethyl)­benzoate according to the general procedure A. FC in *n*-hexane/ethyl acetate 1/1, R*f* = 0.45. Whitish powder (66% yield); mp 75.6 °C. ^1^H NMR (400 MHz, CD_3_OD): δ: 1.49 (s, 6H, C*H*_*2*_); 1.58–168 (m, 4H, C*H* and C*H*_*2*_); 1.77 (d, 3H, C*H* and C*H*_*2*_, *J* = 11.7 Hz); 1.96 (bs, 3H, C*H* and C*H*_*2*_); 2.23–232 (m, 1H, C*H*); 2.46–2.52 (m, 2H, C*H*_*2*_); 2.74 (dd, 1H, CH, *J’* = 6.9 and *J’’* = 9.7 Hz); 2.80–2.85 (m, 3H, C*H* and C*H*_*2*_); 3.73 (dd, 2H, CH_2_, *J’* = 13.6 and *J’’* = 30.4 Hz); 3.92 (s, 3H, C*H*_*3*_); 4.15–4.22 (m, 2H, C*H*); 7.49 (d, 2H, aryl, *J* = 7.7 Hz); 8.00 (d, 2H, aryl, *J* = 8.4 Hz). ^13^C NMR (100 MHz, CD_3_OD): δ: 28.4, 31.7, 33.6, 36.7, 39.8, 49.1, 51.2, 51.3, 52.5, 59.3, 60.6, 128.8, 128.9, 129.2, 143.8, 159.6, 167.0. HR-MS *m*/*z* calcd for C_25_H_35_N_3_O_3_ [(M + H)]^+^: 426.2751; found 426.2742.

#### 4-((3-(3-(Adamantan-1-ylmethyl)­ureido)­pyrrolidin-1-yl)­methyl)­benzoic Acid (**33**)

Derivative **33** was obtained from **32**, in accordance with the general procedure E. Precipitated from MeOH/diethyl ether. Whitish powder (92% yield); mp 207 °C (decomp.). ^1^H NMR (400 MHz, CD_3_OD): δ: 1.48 (s, 6H, C*H*_*2*_); 1.64 (d, 3H, C*H* and C*H*_*2*_, *J* = 11.6 Hz); 1.74 (d, 3H, C*H* and C*H*_*2*_, *J* = 11.8 Hz); 1.93 (bs, 3H, C*H* and C*H*_*2*_); 1.97–2.03 (m, 1H, C*H*); 2.42–2.50 (m, 1H, C*H*); 2.80 (s, 2H, C*H*_*2*_); 3.14–3.24 (m, 2H, C*H*_*2*_); 3.37–3.43 (m, 1H, C*H*); 3.47–3.56 (m, 1H, C*H*); 4.29–4.39 (m, 3H, C*H* and C*H*_*2*_); 7.59 (d, 2H, aryl, *J* = 8.2 Hz); 8.04 (d, 2H, aryl, *J* = 8.6 Hz). ^13^C NMR (100 MHz, CD_3_OD): δ: 28.4, 30.2, 33.6, 36.7, 39.8, 48.6, 51.3, 52.5, 57.8, 59.0, 129.6, 129.8, 135.0, 136.1, 159.4, 171.2. HR-MS *m*/*z* calcd for C_24_H_33_N_3_O_3_ [(M + H)]^+^: 412.2595; found 412.2617.

#### *tert*-Butyl 4-(3-(adamantan-1-ylmethyl)­ureido)­piperidine-1-carboxylate (**34**)

Synthesized from *tert*-butyl 4-aminopiperidine-1-carboxylate and adamantan-1-ylmethanamine following the general procedure C. FC in dichloromethane/ethyl acetate 9/1, R*f* = 0.46. Whitish oil (68% yield). ^1^H NMR (400 MHz, CD_3_OD): δ: 1.24–1.35 (m, 4H, C*H*_*2*_); 1.47 (s, 9H, C*H*_*3*_); 1.52 (bs, 6H, C*H*_*2*_); 1.68 (d, 3H, C*H* and C*H*_*2*_, *J* = 12.0 Hz); 1.77 (d, 3H, C*H* and C*H*_*2*_, *J* = 12.9 Hz); 1.84–1.89 (m, 2H, C*H*_*2*_); 1.98 (bs, 3H, C*H* and C*H*_*2*_); 2.88 (s, 2H, C*H*_*2*_); 3.66–3.70 (m, 1H, *CH*); 3.97 (d, 2H, C*H*_*2*_, *J* = 13.5 Hz); HR-MS *m*/*z* calcd for C_22_H_37_N_3_O_3_ [(M + H)]^+^: 392.2908; found 392.2883.

#### 1-(Adamantan-1-ylmethyl)-3-(piperidin-4-yl)­urea (**35**)

Synthesized from **34** following the general procedure D. Whitish powder (94% yield). ^1^H NMR (400 MHz, CD_3_OD): δ: 1.52 (bs, 6H, C*H*_*2*_); 1.62–1.70 (m, 5H, C*H* and C*H*_*2*_); 1.77 (d, 3H, C*H* and C*H*_*2*_, *J* = 12.8 Hz); 1.97 (bs, 3H, C*H* and C*H*_*2*_); 2.83 (s, 2H, C*H*_*2*_); 2.09–2.13 (m, 2H, *CH*_*2*_); 3.07–3.13 (m, 2H, *CH*_*2*_); 3.37–3.42 (m, 2H, *CH*_*2*_); 3.75–3.82 (m, 1H, *CH*); HR-MS *m*/*z* calcd for C_17_H_29_N_3_O [(M + H)]^+^: 292.2383; found 292.2352.

#### Methyl 4-((4-(3-(Adamantan-1-ylmethyl)­ureido)­piperidin-1-yl)­methyl)­benzoate (**36**)

Synthesized from **35** and methyl 4-(bromomethyl)­benzoate applying the general procedure A. FC in dichloromethane/ethyl acetate 9.5/0.5, R*f* = 0.43. Whitish oil (52% yield). ^1^H NMR (400 MHz, CD_3_OD): δ: 1.31–1.41 (m, 2H, C*H*_*2*_); 1.51 (bs, 6H, C*H*_*2*_); 1.67 (d, 3H, C*H* and C*H*_*2*_, *J* = 11.7 Hz); 1.76 (d, 3H, C*H* and C*H*_*2*_, *J* = 12.1 Hz); 1.96 (bs, 5H, C*H* and C*H*_*2*_); 2.74–2.86 (m, 5H, C*H* and C*H*_*2*_); 2.89 (d, 2H, C*H*_*2*_, *J* = 6.1 Hz); 3.92 (s, 3H, C*H*_*3*_); 3.93 (s, 2H, C*H*_*2*_); 4.02 (d, 2H, C*H*_*2*_, *J* = 13.5 Hz); 7.51 (d, 2H, C*H*_*2*_, *J* = 8.2 Hz); 8.01 (d, 2H, C*H*_*2*_, *J* = 8.2 Hz); HR-MS *m*/*z* calcd for C_26_H_37_N_3_O_3_ [(M + H)]^+^: 440.2908; found 440.2873.

#### 1-(Adamantan-1-ylmethyl)-3-(1-(4-chlorobenzyl)­piperidin-4-yl)­urea (**37**)

Derivative **37** was obtained from **35**, in accordance with the general procedure A. FC in dichloromethane/ethyl acetate 9.5/0.5, R*f* = 0.47. Whitish oil (50% yield). ^1^H NMR (400 MHz, CD_3_OD): δ: 1.41–1.53 (m, 8H, C*H*_*2*_); 1.68 (d, 3H, C*H* and C*H*_*2*_, *J* = 11.0 Hz); 1.77 (d, 3H, C*H* and C*H*_*2*_, *J* = 12.3 Hz); 1.88 (d, 2H, C*H*_*2*_, *J* = 10.3 Hz); 1.96 (bs, 3H, C*H* and C*H*_*2*_); 2.17 (t, 2H, C*H*_*2*_, *J* = 10.1 Hz); 2.81 (s, 2H, C*H*_*2*_); 3.52 (s, 2H, C*H*_*2*_); 7.33 (s, 4H, aryl). ^13^C NMR (100 MHz, CD_3_OD): δ: 28.4, 33.6, 36.8, 39.9, 39.91, 46.5, 51.3, 51.4, 51.9, 61.7, 128.0, 130.8, 132.8, 136.1, 159.4. HR-MS *m*/*z* calcd for C_24_H_34_ClN_3_O [(M + H)]^+^: 416.2463; found 416.2459.

#### 4-((4-(3-(Adamantan-1-ylmethyl)­ureido)­piperidin-1-yl)­methyl)­benzoic Acid (**38**)

Derivative **38** was obtained from **36**, in accordance with the general procedure E. Precipitated from MeOH/diethyl ether. Whitish powder (95% yield); mp 225 °C (decomp.). ^1^H NMR (400 MHz, CD_3_OD): δ: 1.52 (s, 6H, C*H*_*2*_); 1.62 (dd, 2H, C*H*_*2*_, *J’* = 4.0 and *J’’* = 12.7 Hz); 1.68 (d, 3H, C*H* and C*H*_*2*_, *J* = 11.0 Hz); 1.78 (d, 3H, C*H* and C*H*_*2*_, *J* = 12.3 Hz); 1.97 (bs, 3H, C*H* and C*H*_*2*_); 2.24 (d, 2H, C*H*_*2*_, *J* = 9.8 Hz); 2.90 (t, 4H, C*H*_*2*_, *J* = 11.8 Hz); 3.42–3.51 (m, 1H, C*H*), 4.22 (d, 2H, C*H*_*2*_, *J* = 13.6 Hz); 4.37 (s, 2H, C*H*_*2*_); 7.68 (d, 2H, aryl, *J* = 7.6 Hz); 8.12 (d, 2H, aryl, *J* = 7.8 Hz). ^13^C NMR (100 MHz, CD_3_OD): δ: 28.2, 28.4, 34.1, 36.8, 40.0, 42.2, 51.8, 129.7, 130.1, 131.9, 136.0, 158.7, 167.7. HR-MS *m*/*z* calcd for C_25_H_35_N_3_O_3_ [(M + H)]^+^: 426.2751; found 426.2748.

### Computational Details

The initial geometries of the small molecules were obtained by using the Build Panel of Maestro (version 14.3)^50^ and then refined by MacroModel module^51–53^ by means of: OPLS4 force field,^54^ GB/SA (generalized Born/surface area)^55^ solvent model for H_2_O, Polak-Ribier conjugate gradient minimization method (maximum derivative less than 0.001 kcal/mol). Both enantiomers of two pyrrolidine-based compounds (**32** and **33**) were considered in the calculations. The so-refined structures were processed by means of LigPrep^56,57^ generating the protonation states at pH = 7.0 ± 1.0 and verifying for possible tautomers. Moreover, in the LigPrep stage, appropriate states for binding to metals and their relative penalty terms were generated to be accounted by Glide. The X-ray crystallographic structures of sEH (PDB ID: 3i28)^19^ and 5-LOX (PDB ID: 3O8Y)^58^ were employed as protein models and managed by Protein Preparation Wizard:^59–61^ checking for missing side chains and loops, residue alternate positions, bond order assignment, all hydrogen addition, side chain charges were assigned accounting for on their p*K*_a_ at pH 7.0. All hydrogen bonds were refined by the optimize option. Co-crystallized ligands and water molecules were removed. Molecular docking was performed by Glide software.^62–65^ The conformational search parameters, applied for molecular docking calculations, were formerly described for both sEH and 5-LOX.^16,17^ Moreover, to account for 5-LOX cavity shaping upon ligand binding, the Induced Fit Docking approach (IFD)^66–70^ was employed. Maestro (version 14.3) was employed for in silico investigation and figure creation. The criteria used to define protein–ligand interactions in the docking poses derived from interaction analysis software and visual inspection.

### Molecular Dynamics

The docked structures of **1** bound to sEH and 5-LOX were inputted for molecular dynamics simulation through a prior preparation by means of by System Builder^71,72^ in Desmond, using an orthorhombic box with a 10 Å distance of the buffer from the solute, OPLS4 force field,^54^ the TIP3P^73^ solvation model, Na^+^ and Cl^–^ ions for electroneutrality, along with a supplementary NaCl solution (0.15 M). The zero-order bonds between the Fe^2+^ and coordinating atoms of amino acids were added. The so generated molecular system were relaxed as follows: (1) 100 ps of NVT (method = ″Brownie″) simulation at 10 K, with restrained solute heavy atoms (50 kcal/mol); (2) 12 ps of NVT (method = Langevin) simulation at 10 K, with restrained solute heavy atoms (50 kcal/mol) simulation at 10 K, with restrained solute heavy atoms (50 kcal/mol); (3) 0.12 ns of NPT (method = Langevin) simulation (10 K) with restrained solute heavy atoms (10 kcal/mol); (4) 0.12 ns of NPT (method = Langevin) simulation (310 K) with restrained solute heavy atoms (10 kcal/mol); (5) 0.24 ns of NPT (method = Langevin) simulation (310 K) with no restraints. Each equilibration phase of three systems was evaluated by the Simulation Quality Analysis tool of Desmond, examining pressure, volume, temperature, total and potential energies. Unrestrained molecular dynamics (100 ns, 310 K) with NPT (1.01 bar) ensemble class were run, with 2.0 fs of integration time step and 1.2 ps of recording time.

### *In Vitro* Pharmacological Characterization

#### Cell-Free 5-LOX Activity Assay

Human recombinant 5-LOX was expressed in *Escherichia coli* Bl21 (DE3) cells, transformed with the plasmid pT3-5LO and 5-LOX was isolated using an ATP-agarose column by affinity chromatography.^74^ *E. coli* were lysed in 50 mM triethanolamine/HCl pH 8.0 with EDTA (5 mM), soybean trypsin inhibitor (60 μg/mL), phenylmethanesulfonyl fluoride (1 mM), dithiothreitol (1 mM), and lysozyme (1 mg/mL) and sonified 3 times for 15 s. The homogenate was centrifuged at 40,000*g* for 20 min at 4 °C and the supernatant was transferred to an ATP-agarose column (Sigma-Aldrich, Deisenhofen, Germany), which was sequentially washed with PBS pH 7.4, 1 mM EDTA, 50 mM phosphate buffer pH 7.4, 0.5 M NaCl, 1 mM EDTA, and finally 50 mM phosphate buffer pH 7.4 plus 1 mM EDTA. The enzyme was eluted with 50 mM phosphate buffer pH 7.4, 1 mM EDTA, and 20 mM ATP.

Isolated 5-LOX (0.5 μg in PBS pH 7.4 containing 1 mM EDTA) was preincubated with vehicle (DMSO) or test compounds for 15 min on ice. 5-LOX product formation was started by addition of 20 μM arachidonic acid (Sigma-Aldrich) and CaCl_2_ (2 mM) and stopped by addition of an equal volume of ice-cold methanol containing PGB_1_ (200 ng) as internal standard after 10 min at 37 °C. Major 5-LOX products (all-*trans* isomers of LTB_4_ and 5-HETE) were extracted on Sep-Pak C18 35 cc Vac Cartridges (Waters, Milford, MA), separated by RP-HPLC on a Nova-Pak C18 Radial-Pak Column (4 μm, 5 × 100 mm, Waters) under isocratic conditions (73% methanol/27% water/0.007% trifluoroacetic acid) at a flow rate of 1.2 mL/min and detected at 235 and 280 nm.^74^

#### Analysis of Cell-Free sEH Activity

Human recombinant sEH was expressed in Sf9 insect cells and isolated by affinity chromatography. In brief, Sf9 cells were infected with a recombinant baculovirus and lysed after 72 h in 50 mM NaHPO_4_ pH 8, 300 mM NaCl, 10% glycerol, 1 mM EDTA, 1 mM phenylmethanesulfonyl fluoride, 10 μg/mL leupeptin, and 60 μg/mL STI by sonication (3 × 10 s, 4 °C). Sequential centrifugation at 20,000*g* (10 min, 4 °C) and 100,000*g* (60 min, 4 °C) yielded a supernatant, which was then subjected to benzylthio-sepharose affinity chromatography. Elution with 4-fluorochalcone oxide in PBS pH 7.4 with 1 mM dithiothreitol and 1 mM EDTA yielded sEH, which was dialyzed and concentrated. Isolated sEH (60 ng) in 25 mM Tris HCl pH 7 with 0.1 mg/mL bovine serum albumin (BSA) was preincubated with the vehicle (DMSO) or test compounds for 1 min at room temperature. The sEH substrate PHOME (20 μM, Cayman Chemicals) was added to start the enzymatic reaction, which was stopped after 60 min in the darkness by addition of ZnSO_4_ (200 mM). The formation of the fluorescent product 6-methoxynaphtaldehyde was measured using a NOVOstar fluorescence microplate reader (BMG Labtech, Ortenberg, Germany), with excitation at 330 and emission at 465 nm.

#### Activity Assays of Isolated COX-1 and COX-2

Purified ovine COX-1 (Cayman Chemicals; 50 units) or human recombinant COX-2 (Cayman Chemicals; 20 units) were diluted in 100 mM Tris buffer pH 8, containing 5 mM glutathione, 5 μM hemoglobin, and 100 μM EDTA. Samples were preincubated with test compounds or vehicle (0.1% DMSO) for 5 min at 4 °C, followed by 1 min at 37 °C. Then, AA (5 μM for COX-1 and 2 μM for COX-2) was added, and incubations were continued for another 10 min at 37 °C. Formation of COX-derived 12-hydroxyheptadecatrienoic acid (12-HHT) from AA was analyzed by RP-HPLC as described previously.^75^

### Pharmacokinetic Evaluation

#### LC-MS Instrumentation and Analytical Conditions

LC-MS/MS analyses were performed using a Vanquish UHPLC coupled to an Orbitrap Exploris 120 mass spectrometer (Thermo Fisher Scientific, Bremen, Germany) equipped with a HESI II source operating in positive ion mode. Chromatographic separation was achieved on a Kinetex 2.6 μm EVO C18 (100 × 2.1 mm) column thermostated at 40 °C using a binary gradient of water (A) and acetonitrile (B) (both containing 0.1% v/v formic acid, 5–95% B over 10 min) at a flow rate of 0.4 mL·min^–1^.

Full-scan MS spectra were acquired at 60,000 resolution (*m*/*z* 100–1500), with data-dependent MS/MS at 15,000 resolution, using a normalized collision energy (HCD) of 30%.

Calibration curves were constructed from working solutions prepared by serial dilution in methanol of a DMSO stock solution, using tolbutamide (1 μM) as the internal standard.^76^ Each of the six calibration levels was analyzed in triplicate. Linear regression of analyte-to-IS peak area ratios produced highly linear curves (*R* ^2^ ≥ 0.99), described by the following equation: *y* = 0.4305*x* + 0.2324.

#### Plasma Stability Assay

Plasma stability was assessed by incubating compound **11** (10 μM) in human plasma (VWR International s.r.l., Milan, Italy) at 37 °C. At 60 and 120 min, aliquots were quenched with an ice-cold methanol containing IS. Samples were analyzed by LC-MS to quantify the remaining parent compound, relative to *time-zertime-zero (t*_0_), which was obtained by incubating the tested compound in plasma followed by immediate quenching with organic solvent. Procaine and procainamide were used as reference compounds for low and high plasma stability, respectively. All experiments were performed in triplicate.

#### Assessment of Plasma and Microsomal Protein Binding

The extent of plasma or microsomal protein binding of compound **11** was assessed by dialysis experiments using the Pierce Rapid Equilibrium Dialysis (RED) plate (Thermo Fisher Scientific, Bremen, Germany).^77^

Briefly, compound **11** (10 μM) was spiked into human plasma or HLMs and loaded into the donor chamber, while isotonic PBS (100 mM sodium phosphate, 150 mM sodium chloride) was added to the receiver chamber. The system, based on a semipermeable membrane separating the two compartments, allows diffusion of low-molecular-weight compounds while retaining plasma or microsomal proteins. The plate was sealed and incubated at 37 °C under gentle shaking to enable equilibrium, which was typically reached within 4 h. At the end of the incubation, aliquots were collected from both donor (plasma or HLMs) and receiver (buffer) chambers and subjected to protein precipitation using ice-cold methanol containing IS. Samples were centrifuged, and the resulting supernatants were analyzed by LC-MS to quantify compound concentrations and determine the fraction unbound in plasma (*f*_u,p_) and in microsomes (*f*_u,mic_). The percentage of free compound was calculated as follows: % Free = (concentration in the buffer chamber/concentration in the plasma or HLMs chamber) × 100; % Bound = 100 – % Free.

Propranolol and atenolol were included as reference compounds, representing high and low protein binding, respectively. All experiments were performed in triplicate.

#### Metabolic Stability in Liver Microsomes Supplemented with NADPH

HLMs (Thermo Fisher Scientific, Bremen, Germany) were used for the microsomal stability assay of compound **11**. Reactions were initiated by adding of NADPH and the tested compound (10 μM) was incubated at 37 °C for 15, 30, 45, and 60 min in a Thermomixer (Eppendorf, Hamburg, Germany). The reaction was quenched with ice-cold methanol containing IS, followed by centrifugation at 14,000 rpm for 7 min at 4 °C (Eppendorf, Hamburg, Germany). The supernatants were then analyzed by LC-MS.

The t_0_ control was obtained by immediate quenching with ice-cold methanol after addition of the test compound. Testosterone served as a positive control, while the negative control consisted of incubation in the absence of cofactor for up to 60 min.

Finally, we evaluated the microsomal stability of compound **11** in HLMs supplemented with NADPH in the presence of the pan-CYP inhibitor ABT to evaluate whether hepatic biotransformation was CYP-mediated.^78^ Briefly, HLMs were preincubated with ABT and NADPH in phosphate buffer (pH 7.4) for 30 min at 37 °C under gentle shaking. Following preincubation, the test compound or testosterone (positive control) was added, and samples were incubated at 37 °C for 60 min. Reactions were quenched with ice-cold methanol containing IS, centrifuged, and the supernatants were analyzed by LC-MS.

#### Metabolic Stability in HLMs Supplemented with UDP-GlcUA

The extent of **UGT**-mediated glucuronidation of the tested compound was assessed in HLMs using uridine UDP-GlcUA as cofactor. Alamethicin was added to permeabilize the microsomal membrane and allow access of cofactors and substrates to UGT active sites. Incubations of compound **1** **1** (10 μM) were performed at 37 °C for 15, 30, 45, and 60 min, and reactions were terminated by addition of ice-cold methanol containing IS. Samples were subsequently centrifuged, and the resulting supernatants were analyzed by LC-MS to quantify parent compound depletion. A time-zero­(*t*_0_) control was prepared by immediate quenching with organic solvent, while 7-hydroxycoumarin and incubations performed in the absence of UDP-GlcUA were used as positive and negative controls, respectively.

#### Hepatic Clearance Prediction by IVIVE

A common approach to hepatic clearance prediction is the use of the *in vitro-in vivo* extrapolation (IVIVE) initially proposed by Rane et al. and now widely adopted in the field.^29,79^

Briefly, the IVIVE approach involves four steps: (*i*) determining *in vitro* intrinsic clearance (CL_in vitro_) of test compound in liver microsomes; (*ii*) converting measured *in vitro* intrinsic clearance (CL_in vitro_) to unbound ***in vitro*** intrinsic clearance (CL_in vitro,u_) using unbound fraction of drug (*f*_u,mic_) in liver microsomes (CL_in vitro,u_ = CL_in vitro_/*f*_u,mic_); (*iii*) scaling the unbound intrinsic clearance (CL_in vitro,u_) to*in vivo* unbound intrinsic clearance (CL_int,u_) by applying physiologically based scaling factors and the unbound fraction of drug in plasma (*f*_u,p_); and (*iv*) inputting the scaled *in vivo* CL_int,u_ value into a mathematical liver model such as the “well-stirred model” (WSM), to calculate *in vivo*hepatic clearance.^80^

*In vitro* half-life (*t*_1/2_, min) was calculated using eq 1:

1 $$t_{1/2}=0.693/\kappa$$

where κ is the slope found in the linear fit of the natural logarithm of the fraction remaining of the parent compound vs incubation time.

Microsomal intrinsic clearance (CL_int,micr_, μL min^–1^ mg^–1^) was determined using eq 2:

2 $${\mathrm{CL}}_{\mathrm{int},\mathrm{micr}}=1000\times \left(0.693/t_{1/2}\right)/0.5$$

where the microsomal protein concentration in the incubation mixture was 0.5 mg mL^–1^, and 1000 is the unit-scaling factor for volume (μL).

The unbound intrinsic clearance in microsomes (CL_int,u_, μL min^–1^ mg^–1^) was calculated according to eq 3.

3 $${\mathrm{CL}}_{\mathrm{int},u}={\mathrm{CL}}_{\mathrm{int},\mathrm{micr}}/f_{u,\mathrm{mic}}$$

Where *f*_u,mic_ is the fraction unbound in liver microsomes.

The intrinsic clearance was then scaled to the whole liver to obtain*in vivo*intrinsic hepatic clearance (CL_int,scaled_, mL min^–1^ kg^–1^) using eq 4:

4 $${\mathrm{CL}}_{\mathrm{int},\mathrm{scaled}}={\mathrm{CL}}_{\mathrm{int},u}\times \mathrm{SF}$$

where SF represents the physiological scaling factors, including milligrams of microsomal protein per gram of liver and the liver-to-body weight ratio.^81^

Finally, the total hepatic clearance (CL_h_) was estimated using the well-stirred model, as represented by eq 5:

5 $${\mathrm{CL}}_{h}=\left(Q_{h}\times f_{u,p}\times {\mathrm{CL}}_{\mathrm{int},\mathrm{scaled}}\right)/\left(Q_{h}+f_{u,p}\times {\mathrm{CL}}_{\mathrm{int},\mathrm{scaled}}\right)$$

where *Q*_h_ is the hepatic blood flow, *f*_u,p_ is the fraction unbound in plasma, and CL_int,scaled_ is the scaled unbound *in vivo* intrinsic clearance determined from liver microsomal incubations.

#### Glutathione (GSH) Trapping Assay

The GSH trapping assay was performed under the same experimental conditions as the liver microsomal stability assay, except for the inclusion of NADPH and GSH (1:1, v/v). After 60 min of incubation with HLMs at 37 °C, the reaction was quenched with water followed by ice-cold acetonitrile/methanol (9:1, v/v) containing IS. Samples were centrifuged, and the supernatants were concentrated under a nitrogen stream at 35 °C (Concentrator Plus, Eppendorf, Milan, Italy) and analyzed by LC-MS.^82^ Diclofenac and acetaminophen were used as positive controls.

#### Metabolite Identification (MetID) Following Microsomal Incubation

MetID data processing was performed using Compound Discoverer 3.3 software (Thermo Fisher Scientific Bremen, Germany) using a customized MetID workflow for the detection and structural elucidation of both expected and novel metabolites, following the procedure reported by Carullo et al.,^83^ with minor modifications.

In silico biotransformation predictions were generated using GLORYx (NERDD portal, University of Vienna, https://nerdd.univie.ac.at/gloryx), and the resulting compound list (including name, molecular formula, exact mass, and SMILES) was imported into the workflow.^84^ Processing steps included retention time alignment, isotope pattern confirmation, compound grouping, FISh scoring, fragment annotation, and gap filling.

For GSH trapping experiments, adduct identification was based on the detection of characteristic mass shifts (+305 Da), diagnostic fragment ions, and neutral losses typical of glutathione conjugates, along with isotopic pattern matching and high-resolution mass accuracy.

### *In Vivo* Pharmacokinetic Study

#### Animals

Male CD-1 mice (35–40 g, 8 weeks, Charles River Laboratories; Calco, Italy) were fed with standard rodent chow and water and acclimated for 4 days at a 12 h light and 12 h dark schedule in a constant air-conditioned environment (21 ± 2 °C). Mice were randomly assigned to groups, and experiments were carried out during the light phase. Experimental procedures were conducted in conformity with Italian (D.L. 26/2014) and European (directive 2010/63/EU) regulations on the protection of animals used for scientific purposes and approved by the Italian Ministry (No. 479/2025-PR).

#### Sample Preparation

CD-1 mice received compound **11** (10 mg/kg i.p.) injection and after selected time points, the mice were euthanized and blood was collected by intracardiac puncture. Plasma was obtained by centrifugation at 800g at 4 °C for 10 min and immediately frozen at −80 °C. CD-1 mice received compound 11 (10 mg/kg i.p.) injection and after selected time points, the mice were euthanized and blood was collected by intracardiac puncture. Plasma was obtained by centrifugation at 800g at 4 °C for 10 min and immediately frozen at −80 °C.For the extraction of compound **11** from mouse plasma, protein precipitation was employed. Briefly, frozen plasma samples were thawed at room temperature. Ice-cold acetonitrile (200 μL) containing IS (compound **33**) was added to 100 μL of plasma. The extraction was performed by vortex mixing for 5 min, followed by centrifugation for 10 min at 14,000 rpm and 4 °C. The supernatants were collected and analyzed by LC-MS.

#### LC-MS Instrumentation and Analytical Conditions

Analyses were performed using a Nexera UHPLC system (Shimadzu, Kyoto, Japan) consisting of a SCL-40 system controller, two LC-40D X3 pumps, a CTO-40C column oven, and an SIL-40C X3 autosampler coupled online to a SCIEX Triple Quad 7500 LC-MS/MS System - QTRAP (SCIEX, Framingham, Massachusetts, USA). A SCIEX OptiFlow ionization source was used. The chromatographic separation was performed on a Kinetex 2.6 μm EVO C18 100 Å (50 × 2.1 mm) column (Phenomenex, Bologna, Italy). The column temperature and flow rate were set at 40 °C and 0.4 mL min^–1^, respectively. The mobile phases were water (A) and acetonitrile (B), both containing 0.1% (v/v) formic acid, using the following gradient: 0–5 min, 5–95% B; 5.01–6 min, isocratic to 95% B; 6.01–6.5 min, 95–5% B; followed by 1.5 min for column re-equilibration.

MS data were acquired in positive ionization mode with curtain gas at 40 psi, gas 1 at 60 psi, gas 2 at 55 psi, collision-activated dissociation (CAD) gas set to 9, ion spray voltage of 5500 V, and interface temperature of 400 °C. Scheduled multiple reaction monitoring (sMRM) was employed for data acquisition by monitoring the precursor–to-product ion transitions within defined retention time windows (±50 s tolerance), as reported in Table S21. sMRM acquisition was performed using a triggering threshold of >1.0 × 10^3^ cps, with dynamic background subtraction enabled.

The same sMRM parameters were applied to metabolites identified in MetID analyses to evaluate their presence in *in vivo* plasma samples.

#### Bioanalytical Method Validation

Method validation was performed in accordance with the U.S. FDA Bioanalytical Method Validation Guidance for Industry. The analytical platform was evaluated in terms of linearity, sensitivity, accuracy, precision, recovery, and carryover Table S22.

Stock solutions of compound **11** and IS were prepared in DMSO (1 μg mL^–1^). To prepare working calibration standards, the stock solution of compound **11** was diluted with acetonitrile and spiked into mouse plasma (100 μL) to achieve a concentration range of 1–50 ng mL^–1^, using six concentration levels, each analyzed in triplicate. An equal amount of IS (compound **33**) was added to each level to obtain a final concentration of 5 ng mL^–1^.

Peak area ratios (analyte/IS) were plotted against corresponding concentrations (ng mL^–1^), and calibration curves were generated by linear regression with *R* ^2^ values ≥0.999. The calibration curve included a blank sample (plasma processed without IS), a zero calibrator (plasma processed with IS), and nonzero calibrators covering the expected concentration range.

The lowest standard on the calibration curve which could be measured with accuracy and precision was accepted as the lower limit of quantification (LLOQ). The limit of detection (LOD) and limit of quantification (LOQ) were calculated by using the standard deviation (SD) and the slope of the calibration curve, applying factors of 3.3 and 10, respectively.

The acceptance criterion of each standard concentration was ±15% deviation from the nominal concentration, except for the LLOQ (±20%). Intraday precision and accuracy were estimated by analyzing three replicates at three quality control (QC) levels: low (LQC), medium (MQC), and high (HQC) concentrations. Interday precision was determined by analyzing the same QC levels across three independent runs. Acceptance criteria required accuracy within 85–115% of nominal values (80–120% for LLOQ) and precision ≤ 15% coefficient of variation (CV) (≤20% for LLOQ).

The obtained data demonstrated acceptable accuracy, precision, and robustness of the developed analytical method.

#### Cerulein-Induced Acute Pancreatitis

A murine model of CER-induced AP was established as previously described.^17^ Briefly, male CD-1 mice received intraperitoneal (i.p.) injections of CER (50 μg/kg, suspended in saline) hourly for a total of 6 injections. Mice were randomly divided into: (a) Sham group which received saline; (b) CER + vehicle (veh.) group, treated with CER and received the veh. (0.5 mL of DMSO 2%, i.p.) 30 min and 2.5 h after first cerulein administration (Figure 3A); (c) CER + compound **11** group, treated with CER and **11** (10 mg/kg, i.p.) 30 min and 2.5 h after first CER administration; (d) CER + AUDA (10 mg/kg, i.p.), a known sEH inhibitor, 30 min and 2.5 h after first CER administration; (e) CER + zileuton (ZILE) (10 mg/kg, i.p.), a known 5-LOX inhibitor, 30 min and 2.5 h after first CER administration. The mice were euthanized 6 h after the first CER injection. Pancreases and lungs were removed immediately, frozen, and stored at −80 °C until assayed.

#### Histological Examination

Pancreas tails and the upper right lung lobes were fixed in formaldehyde for histological and immunohistochemical examination. The sections (5 μm) were processed to remove paraffin, rehydrated, and stained with hematoxylin and eosin (H&E). Evaluation of pancreas edema and inflammation signs were performed with a three-point scoring system. Edema: 0, absent or rare; 1, in the interlobular space; 2, in the intralobular space; 3, the isolated-island shape of pancreatic acinus. Inflammation: 0, absent; 1, mild (infiltration in ducts); 2, moderate (infiltration in parenchyma < 50%); 3, severe (infiltration in parenchyma > 50%). Analysis was performed in a blinded manner.

#### Immunohistochemistry

Pancreas and lung slices, after rehydration, were incubated with 3% hydrogen peroxide (Merck, Milan, Italy) for 15 min to quench the endogenous peroxidase activity. Tissue sections were then incubated with normal goat serum (Elabscience, Houston, Texas) for 1 h to reduce nonspecific antibody interactions. Sections were incubated with primary antibody diluted in 0.01 M PBS, 2.5% normal serum (anti-Neutrophils rat monoclonal antibody, dilution 1:100, Abcam) for 30 min. After rinsing with PBS 0.01 M for 5 min, slides were incubated with biotinylated goat antirat IgG (Vector Laboratories, Newark, California), diluted 1:200, for 30 min. After rinsing with PBS 0.01 M for 5 min, slides were incubated with VECTASTAIN ABC Reagent (Vector Laboratories) for 30 min. Other pancreas slice were incubated with the following primary antibodies: anti-F4/80 (dilution 1:100, Proteintech, Manchester, UK), anti-4-HNE (dilution 1:250, Bioss Antibodies, Woburn, Massachusetts), anti-FTH-1 (dilution 1:200, Affinity Biosciences, USA), anti-GPX4 (dilution 1:600, Affinity Biosciences), anti-Nrf2 (dilution 1:200, Affinity Biosciences, USA). After rinsing with PBS 0.01 M, slides were incubated with Peroxidase Affinity Pure Goat anti-rabbit (Jackson ImmunoResearch, 1:500) for 1 h at room temperature. Color development was performed using 3,3-*N*-Diaminobenzidine Tertrahydrochloride solution (Elabscience), and sections were analyzed using Leica Microsystem with a magnification of 20x and 40x.

#### Biochemical Assays

Blood samples were collected by direct intracardiac puncture 6 h after the first CER injection and plasma was obtained by centrifugation at 800 g at 4 °C for 10 min and immediately frozen at −80 °C. Levels of amylase, lipase, ALT, AST, GGT and urea were measured in the plasma by a clinical laboratory (LUNAVET LAB S.a.s., Salerno, Italy).

#### Data Analysis

A noncompartmental analysis (NCA) of PK of compound **11** was performed using PK solver software. The maximum concentration (*C*_max_) and time to reach *C*_max_ (*T*_max_) were determined from the plasma concentration–time data. The area under the plasma concentration–time curve from time 0 to infinity (AUC_0–∞_) was calculated using the linear trapezoidal rule. The elimination half-life (*t*_1/2_) was calculated as ln(2)/*k*_e_, where *k*_e_ is the terminal elimination rate constant.

The *in vivo* results are expressed as mean ± SEM of the mean of n observations, where n represents the number of animals. Statistical evaluation was performed by one-way ANOVA using GraphPad Prism (GraphPad Software, Inc., San Diego, CA) followed by a Bonferroni post hoc test for multiple comparisons, respectively. Post hoc tests were run only when F achieved *P* < 0.05, and there was no significant variance in the homogeneity. P value <0.05 was used to define statistically significant differences between mean values.

### Lipid Mediators UPLC-MS/MS Analysis *In Vivo*

#### Extraction of Lipid Mediators from Plasma, Lung, and Pancreas

The extraction of targeted LM from plasma was performed as follows: 400 μL of a 10% v/v methanol solution was added in a sample tube, then 65 μL of plasma and a mix of deuterated standards were spiked in each sample tube. After 10 min of centrifugation at 15,000 rpm and 4 °C, supernatants were diluted with 500 μL of PBS prior loading on solid-phase extraction cartridges.

The extraction of targeted lipid mediators from lung and pancreas tissues was performed on 5 mg of freeze-dried tissue prior to the extraction protocol. 500 μL of a 10% v/v methanol solution were added to each sample and subsequently mixed. After 3 min of sonication in an ultrasonic bath, the samples were diluted with 500 μL of PBS, spiked with a mix of deuterated standards, then mixed, sonicated in an ultrasonic bath and finally centrifugated for 10 min at 4 °C. For solid-phase extraction cartridges, Strata-X Polymeric reversed-phase SPE columns (100 mg/3 mL) (Phenomenex, Bologna, Italy) were employed. Cartridges were conditioned with 3 mL of MeOH and equilibrated with 3 mL of ddH_2_O. Following loading, washing was performed with 1 mL of 10% MeOH v/v and finally eluted with 1.5 mL of MeOH. Eluted samples were dried with a SpeedVac (Savant, Thermo Scientific, Milan, Italy) and dissolved prior to UPLC-MS/MS analyses in 50 μL of Methanol LC-MS grade.

#### UPLC-MS/MS Analysis

UPLC-MS/MS analyses were acquired using an ACQUITY UPLC I-Class PLUS (Waters, Milford, MA, USA), coupled online to a triple quadrupole MS Xevo TQ-XS (Waters, Milford, MA, USA) equipped with an electrospray ionization (ESI) source operating in ESI negative mode, the acquisition was performed in scheduled multiple reaction monitoring (sMRM). The separation was performed on a Kinetex EVO C18 150 mm × 2.1 mm, 2.6 μm (100 Å) (Phenomenex, Bologna, Italy). The flow rate was set to 0.4 mL/min, and the column oven was 40 °C. The mobile phases were (A) H_2_O/ACN (70:30) acidified with 0.02% CH_3_COOH v/v and (B) ACN/IPA (50:50). The following gradient was used: 0 min, 10%B, 0–11 min 55%B, 11.10 min, 55–99%B, 11.10–12.50 isocratic at 99% B, returning to 10% B in 0.1 min. Injection volume was 4 μL for plasma, 3 μL for tissues. MS source parameters: capillary voltage 3.5 kV, desolvation gas flow and temperature were set to 1200 (L/h) and 500 °C respectively, source temperature 150 °C, sample cone gas flow 150 L/h and collision gas (argon) to 0.18 mL/min. Each analyte was optimized by direct infusion, using standard solution at 0.2 μg/mL. MRM details, such as cone voltages, dwell time, collision energies, parents and daughter ions are reported in Table S23.

Authentic and deuterated LMs standards (Cayman Chemicals, Ann Arbor, USA) were used for the quantitative analysis as external and internal standards respectively. Stock solutions (1 mg/mL) were prepared in MeOH, and the calibration curves were obtained in a concentration range of 0.01–1800 ng/mL using seven concentration levels with triplicate injections for each level. Linear regression was used to generate the calibration curves with *R* ^2^ values ≥0.997 generating calibration curves in the general form of *y* = *ax* + *b*. Extracted ion chromatograms (XIC) area ratios between authentic and deuterated standard (*y*) were plotted against corresponding concentrations (ng/mL) (*x*). The samples analyses were performed randomly on three technical replicates. The quantification of analytes was evaluated in terms of average and expressed in nM for plasma samples and pmol/mg of tissues. LOD and LOQ were calculated by using the standard deviation (SD) and the slope of the calibration curve, multiplied by 3.3 and 10, repeatability was assessed by evaluating peak area and RT of pooled Quality Control (QC) samples across three independent runs and reported as CV% values, all results are reported in Table S24.

## Supplementary Material

























## Notes

11-5LOX (PDB)
11-sEH (PDB)
28-sEH (PDB)
5-5LOX (PDB)
6-5LOX (PDB)
7-5LOX (PDB)
8-5LOX (PDB)
8-sEH (PDB)
9-5LOX (PDB)
9-sEH (PDB)
Molecular strings (CSV)
Figures S1–S48: NMR spectra and HPLC traces of synthesized compounds; Figure S49: MS/MS spectra of metabolites derived from compound 11; Figure S50: Human plasma stability of compound 11 at 37 °C; Figure S51: Plasma concentration–time profiles of metabolites M2 and M3; Figure S52: Effects of compound 11 on isolated COX-1 and COX-2 enzymes; Table S1: Docking scores for best ligand poses vs sEH and selection criteria; Table S2: IFD scores for best ligand poses vs 5-LOX and selection criteria; Tables S3–S14: Distances of the most relevant ligand-sEH interactions; Tables S15–S20: Distances of the most relevant ligand-5-LOX interactions; Table S21: Selected MRM (sMRM) parameters for compound 11, metabolites, and internal standard; Table S22: Intra- and interday precision and accuracy for compound 11 at four QC levels; Table S23: MRM complete parameters; and Table S24: LOD, LOQ, CV% Intraday, and CV % Interday (PDF)

## Abbreviations Used

4-HNE: 4-hydroxynonenal
ABT: 1-aminobenzotriazole
ALT: alanine aminotransferase
AP: acute pancreatitis
AST: aspartate aminotransferase
CAD: collision-activated dissociation
CER: cerulein
CL: clearance
DHET: dihydroxyeicosatrienoic acids
EET: epoxyeicosatrienoic acid
EPA: eicosapentaenoic acid
FLAP: 5-lipoxygenase-activating protein
FMO: flavin-containing monooxygenase
FTH-1: ferritin heavy chain-1
GGT: γ-glutamyltransferase
GPX4: glutathione peroxidase-4
GSH: glutathione
H&E: hematoxylin and eosin
HLM: human liver microsomes
HPLCQqQ- MS/MS: high-performance liquid chromatography-triple quadrupole mass spectrometry
IVIVE: *in vitro*-*in vivo* extrapolation
LLOQ: lower limit of quantification
LM: lipid mediators
LOD: limits of detection
LOQ: limits of quantification
LOX: lipoxygenase
LT: leukotriene
MetID: metabolite Identification
Nrf2: nuclear factor erythroid 2-related factor 2
PG: prostaglandin
sEH: soluble epoxide hydrolase
sMRM: scheduled multiple reaction monitoring
UDP-GlcUA: UDP-glucuronic acid
UGT: UDP-glucuronosyltransferase
XIC: extracted ion chromatograms
ZILE: zileuton

## References

[1] Ouyang G., Pan G., Liu Q., Wu Y., Liu Z., Lu W., Li S., Zhou Z., Wen Y. (2020). The global, regional, and national
burden of pancreatitis in 195 countries and territories, 1990–2017:
a systematic analysis for the Global Burden of Disease Study 2017. BMC Med..

[2] Sindato E. M. (2025). Acute Pancreatitis:
A Narrative Review. Cureus.

[3] Pannala R., Kidd M., Modlin I. M. (2009). Acute Pancreatitis. Pancreas.

[4] Hamesch K., Hollenbach M., Guilabert L., Lahmer T., Koch A. (2025). Practical
management of severe acute pancreatitis. Eur.
J. Int. Med..

[5] Vargas A., Dutta P., Hawa F., Quingalahua E., Marin R., Vilela A., Nix T., Mendoza-Ladd A., Wilcox C. M., Chalhoub J. M., Machicado J. D. (2025). Effect
of selective COX-2 inhibitors and non-selective non-steroidal anti-inflammatory
drugs on severity of acute pancreatitis: A systematic review and meta-analysis. Pancreatology.

[6] Beij A., Verdonk R. C., van Santvoort H. C., de-Madaria E., Voermans R. P. (2025). Acute Pancreatitis: An Update of
Evidence-Based Management
and Recent Trends in Treatment Strategies. United
Eur. Gastroenterol. J..

[7] Ismail O. Z., Bhayana V. (2017). Lipase or amylase for
the diagnosis of acute pancreatitis?. Clin.
Biochem..

[8] Brahadeeswaran S., Sreedhar S., Ramesh S., Tamizhselvi R. (2026). Targeting
ferroptosis mediated cell death: a novel strategy for mitigating acute
pancreatitis. Mol. Biol. Rep..

[9] Dennis E. A., Norris P. C. (2015). Eicosanoid storm
in infection and inflammation. Nat. Rev. Immunol..

[10] Shimizu T. (2009). Lipid Mediators
in Health and Disease: Enzymes and Receptors as Therapeutic Targets
for the Regulation of Immunity and Inflammation. Annu. Rev. Pharmacol. Toxicol..

[11] Liao D., Qian B., Zhang Y., Wu K., Xu M. (2018). Inhibition
of 5-lipoxygenase represses neutrophils activation and activates apoptosis
in pancreatic tissues during acute necrotizing pancreatitis. Biochem. Biophys. Res. Commun..

[12] Polito F., Bitto A., Irrera N., Squadrito F., Fazzari C., Minutoli L., Altavilla D. (2010). Flavocoxid,
a dual inhibitor of cyclooxygenase-2 and 5-lipoxygenase, reduces pancreatic
damage in an experimental model of acute pancreatitis. Br. J. Pharmacol..

[13] Shi, Z. ; He, Z. ; Wang, D. W. CYP450 Epoxygenase Metabolites, Epoxyeicosatrienoic Acids, as Novel Anti-Inflammatory Mediators. Molecules 2022, 27 (12). 3873 10.3390/molecules27123873. 35744996 PMC9230517

[14] Turnbull, J. ; Chapman, V. Targeting the soluble epoxide hydrolase pathway as a novel therapeutic approach for the treatment of pain. Curr. Opin. Pharmacol. 2024, 78. 102477 10.1016/j.coph.2024.102477. 39197248

[15] Fromel T., Hu J., Fleming I. (2023). Lipid mediators
generated by the cytochrome P450-Epoxide
hydrolase pathway. Adv. Pharmacol. Pharma. Sci..

[16] Cerqua I., Musella S., Peltner L. K., D’Avino D., Di Sarno V., Granato E., Vestuto V., Di Matteo R., Pace S., Ciaglia T. (2022). Discovery
and Optimization
of Indoline-Based Compounds as Dual 5-LOX/sEH Inhibitors: In Vitro
and In Vivo Anti-Inflammatory Characterization. J. Med. Chem..

[17] Musella S., D’Avino D., Peltner L. K., Di Sarno V., Cerqua I., Merciai F., Vestuto V., Ciaglia T., Smaldone G., Di Matteo F. (2023). Design, Synthesis, and Pharmacological Characterization
of a Potent Soluble Epoxide Hydrolase Inhibitor for the Treatment
of Acute Pancreatitis. J. Med. Chem..

[18] Ciaglia, T. ; D’Avino, D. ; Jordan, P. M. ; Di Micco, S. ; Musella, S. ; Engelbrecht, H. ; Peltner, L. K. ; Di Sarno, V. ; Merciai, F. ; Perna, S. ; An adamantylureido-benzylamide aniline as FLAP/sEH dual inhibitor: Rational design, in vitro and in vivo lipidomic profiling. Eur. J. Med. Chem. 2026, 302. 118338 10.1016/j.ejmech.2025.118338. 41197472

[19] Eldrup A. B., Soleymanzadeh F., Taylor S. J., Muegge I., Farrow N. A., Joseph D., McKellop K., Man C. C., Kukulka A., De Lombaert S. (2009). Structure-Based
Optimization of Arylamides as Inhibitors
of Soluble Epoxide Hydrolase. J. Med. Chem..

[20] Amano Y., Yamaguchi T., Tanabe E. (2014). Structural insights into binding
of inhibitors to soluble epoxide hydrolase gained by fragment screening
and X-ray crystallography. Bioorg. Med. Chem..

[21] Di Micco, S. ; Musella, S. ; Sala, M. ; Scala, M. C. ; Andrei, G. ; Snoeck, R. ; Bifulco, G. ; Campiglia, P. ; Fasano, A. Peptide Derivatives of the Zonulin Inhibitor Larazotide (AT1001) as Potential Anti SARS-CoV-2: Molecular Modelling, Synthesis and Bioactivity Evaluation. Int. J. Mol. Sci. 2021, 22 (17). 9427 10.3390/ijms22179427. 34502335 PMC8431481

[22] Di
Micco S., Musella S., Scala M. C., Sala M., Campiglia P., Bifulco G., Fasano A. (2021). In silico Analysis
Revealed Potential Anti-SARS-CoV-2 Main Protease Activity by the Zonulin
Inhibitor Larazotide Acetate. Front. Chem..

[23] Di
Micco S., Renga B., Carino A., D’Auria M. V., Zampella A., Riccio R., Fiorucci S., Bifulco G. (2014). Structural
insights into Estrogen Related Receptor-β modulation: 4-Methylenesterols
from Theonella swinhoei sponge as the first example of marine natural
antagonists. Steroids.

[24] Di
Micco S., Terracciano S., Pierri M., Cantone V., Liening S., König S., Garscha U., Hofstetter R. K., Koeberle A., Werz O. (2022). Identification of 2,4-Dinitro-Biphenyl-Based
Compounds as MAPEG Inhibitors. ChemMedChem.

[25] Giordano A., Forte G., Terracciano S., Russo A., Sala M., Scala M. C., Johansson C., Oppermann U., Riccio R., Bruno I., Di Micco S. (2019). Identification of the
2-Benzoxazol-2-yl-phenol Scaffold as New Hit for JMJD3 Inhibition. ACS Med. Chem. Lett..

[26] Di
Micco S., Masullo M., Bandak A. F., Berger J. M., Riccio R., Piacente S., Bifulco G. (2019). Garcinol and Related
Polyisoprenylated Benzophenones as Topoisomerase II Inhibitors: Biochemical
and Molecular Modeling Studies. J. Nat. Prod..

[27] Bettaieb A., Chahed S., Bachaalany S., Griffey S., Hammock B. D., Haj F. G. (2015). Soluble Epoxide Hydrolase Pharmacological Inhibition
Ameliorates Experimental Acute Pancreatitis in Mice. Mol. Pharmacol..

[28] Nageswara Rao Gajula, S. ; Asgar Vora, S. ; G Dikundwar, A. ; Sonti, R. In Vitro Drug Metabolism Studies Using Human Liver Microsomes. In Dosage Forms - Innovation and Future Perspectives; IntechOpen, 2023. 10.5772/intechopen.108246.

[29] Sodhi J. K., Benet L. Z. (2021). Successful and Unsuccessful
Prediction of Human Hepatic
Clearance for Lead Optimization. J. Med. Chem..

[30] Cashman J. R. (2005). Some distinctions
between flavin-containing and cytochrome P450 monooxygenases. Biochem. Biophys. Res. Commun..

[31] Miners J. O., Mackenzie P. I. (1991). Drug glucuronidation
in humans. Pharmacol. Ther..

[32] Montellano P. R. O. d. (2018). 1-Aminobenzotriazole:
A Mechanism-Based Cytochrome P450 Inhibitor and Probe of Cytochrome
P450 Biology. Med. Chem..

[33] Naritomi Y., Terashita S., Kimura S., Suzuki A., Kagayama A., Sugiyama Y. (2001). Prediction
of human hepatic clearance from in vivo
animal experiments and in vitro metabolic studies with liver microsomes
from animals and humans. Drug Metab. Dispos..

[34] Pelkonen O., Turpeinen M. (2007). In vitro–in
vivoextrapolation of hepatic clearance:
Biological tools, scaling factors, model assumptions and correct concentrations. Xenobiotica.

[35] Cubitt H. E., Houston J. B., Galetin A. (2011). Prediction
of Human Drug Clearance
by Multiple Metabolic Pathways: Integration of Hepatic and Intestinal
Microsomal and Cytosolic Data. Drug Metab. Dispos..

[36] Davies B., Morris T. (1993). Physiological Parameters in Laboratory Animals and
Humans. Pharm. Res..

[37] Kilford P. J., Stringer R., Sohal B., Houston J. B., Galetin A. (2009). Prediction
of Drug Clearance by Glucuronidation from in Vitro Data: Use of Combined
Cytochrome P450 and UDP-Glucuronosyltransferase Cofactors in Alamethicin-Activated
Human Liver Microsomes. Drug Metab. Dispos..

[38] Madsen K. G., Skonberg C., Jurva U., Cornett C., Hansen S. H., Johansen T. N., Olsen J. (2008). Bioactivation of Diclofenacin Vitroandin
Vivo: Correlation to Electrochemical Studies. Chem. Res. Toxicol..

[39] Zhang X., Li R., Hu W., Zeng J., Jiang X., Wang L. (2018). A reliable
LC-MS/MS method for the quantification of N-acetyl-p-benzoquinoneimine,
acetaminophen glutathione and acetaminophen glucuronide in mouse plasma,
liver and kidney: Method validation and application to a pharmacokinetic
study. Biomed. Chromatogr..

[40] Yadav D., Timmons L., Benson J. T., Dierkhising R. A., Chari S. T. (2011). Incidence, prevalence, and survival
of chronic pancreatitis:
a population-based study. Am. J. Gastroenterol..

[41] Kim I. H., Morisseau C., Watanabe T., Hammock B. D. (2004). Design, synthesis,
and biological activity of 1,3-disubstituted ureas as potent inhibitors
of the soluble epoxide hydrolase of increased water solubility. J. Med. Chem..

[42] Chen X., Tsvetkov A. S., Shen H. M., Isidoro C., Ktistakis N. T., Linkermann A., Koopman W. J. H., Simon H. U., Galluzzi L., Luo S. (2024). International consensus guidelines for the definition,
detection, and interpretation of autophagy-dependent ferroptosis. Autophagy.

[43] Di Sanzo, M. ; Cozzolino, F. ; Battaglia, A. M. ; Aversa, I. ; Monaco, V. ; Sacco, A. ; Biamonte, F. ; Palmieri, C. ; Procopio, F. ; Santamaria, G. ; Ferritin Heavy Chain Binds Peroxiredoxin 6 and Inhibits Cell Proliferation and Migration. Int. J. Mol. Sci. 2022, 23 (21). 12987 10.3390/ijms232112987. 36361777 PMC9654362

[44] Seibt T. M., Proneth B., Conrad M. (2019). Role of GPX4 in ferroptosis and its
pharmacological implication. Free Radical Biol.
Med..

[45] Yan, R. ; Lin, B. ; Jin, W. ; Tang, L. ; Hu, S. ; Cai, R. NRF2, a Superstar of Ferroptosis. Antioxidants 2023, 12 (9). 1739 10.3390/antiox12091739. 37760042 PMC10525540

[46] Boxhoorn L., Voermans R. P., Bouwense S. A., Bruno M. J., Verdonk R. C., Boermeester M. A., van Santvoort H. C., Besselink M. G. (2020). Acute pancreatitis. Lancet.

[47] Siregar G. A., Siregar G. P. (2019). Management of Severe
Acute Pancreatitis. Open Access Macedonian J.
Med. Sci..

[48] Sikora
Kessler A., Soffer D. E., Abramovitz L., Vera-Llonch M., Kutrieb E., Moynahan A., Weycker D., Baum S. J. (2026). Episodic and Long-term Costs of Acute Pancreatitis
Requiring Hospitalization Among Adults in US Clinical Practice. Pancreas.

[49] Roberts-Thomson I. C. (2021). Progression
from acute to chronic pancreatitis. JGH Open.

[50] Schrödinger Release 2025–1: Maestro; Schrödinger, LLC: New York, NY, 2025.

[51] Schrödinger Release 2025–4: MacroModel; Schrödinger, LLC: New York, NY, 2025.

[52] Mohamadi F., Richards N. G. J., Guida W. C., Liskamp R., Lipton M., Caufield C., Chang G., Hendrickson T., Still W. C. (1990). Macromodel—an integrated software system for
modeling organic and bioorganic molecules using molecular mechanics. J. Comput. Chem..

[53] Watts K. S., Dalal P., Tebben A. J., Cheney D. L., Shelley J. C. (2014). Macrocycle
Conformational Sampling with MacroModel. J.
Chem. Inf. Model..

[54] Lu C., Wu C., Ghoreishi D., Chen W., Wang L., Damm W., Ross G. A., Dahlgren M. K., Russell E., Von Bargen C. D. (2021). OPLS4: Improving Force Field Accuracy on Challenging Regimes of Chemical
Space. J. Chem. Theory Comput..

[55] Still W. C., Tempczyk A., Hawley R. C., Hendrickson T. (1990). Semianalytical
treatment of solvation for molecular mechanics and dynamics. J. Am. Chem. Soc..

[56] Schrödinger Release 2025–1: LigPrep; Schrödinger, LLC: New York, NY, 2025.

[57] Johnston R. C., Yao K., Kaplan Z., Chelliah M., Leswing K., Seekins S., Watts S., Calkins D., Chief Elk J., Jerome S. V. (2023). Epik: pKa and Protonation State Prediction
through Machine Learning. J. Chem. Theory Comput..

[58] Gilbert N. C., Bartlett S. G., Waight M. T., Neau D. B., Boeglin W. E., Brash A. R., Newcomer M. E. (2011). The Structure of Human 5-Lipoxygenase. Science.

[59] Schrödinger Release 2025–1: Protein Preparation Workflow; Epik; Schrödinger, LLC: New York, NY, 2025.

[60] Impact, Prime; Schrödinger, LLC: New York, NY, 2025.

[61] Madhavi
Sastry G., Adzhigirey M., Day T., Annabhimoju R., Sherman W. (2013). Protein and ligand preparation: parameters, protocols,
and influence on virtual screening enrichments. J. Comput.-Aided Mol. Des..

[62] Yang Y., Yao K., Repasky M. P., Leswing K., Abel R., Shoichet B. K., Jerome S. V. (2021). Efficient
Exploration of Chemical Space with Docking
and Deep Learning. J. Chem. Theory Comput..

[63] Friesner R. A., Murphy R. B., Repasky M. P., Frye L. L., Greenwood J. R., Halgren T. A., Sanschagrin P. C., Mainz D. T. (2006). Extra Precision
Glide: Docking and Scoring Incorporating a Model of Hydrophobic Enclosure
for Protein–Ligand Complexes. J. Med.
Chem..

[64] Halgren T. A., Murphy R. B., Friesner R. A., Beard H. S., Frye L. L., Pollard W. T., Banks J. L. (2004). Glide: A New Approach for Rapid,
Accurate Docking and Scoring. 2. Enrichment Factors in Database Screening. J. Med. Chem..

[65] Friesner R. A., Banks J. L., Murphy R. B., Halgren T. A., Klicic J. J., Mainz D. T., Repasky M. P., Knoll E. H., Shelley M., Perry J. K. (2004). Glide:
A New Approach for Rapid, Accurate Docking
and Scoring. 1. Method and Assessment of Docking Accuracy. J. Med. Chem..

[66] Miller E. B., Murphy R. B., Sindhikara D., Borrelli K. W., Grisewood M. J., Ranalli F., Dixon S. L., Jerome S., Boyles N. A., Day T. (2021). Reliable
and Accurate Solution to the Induced Fit Docking
Problem for Protein–Ligand Binding. J.
Chem. Theory Comput..

[67] Xu T., Zhu K., Beautrait A., Vendome J., Borrelli K. W., Abel R., Friesner R. A., Miller E. B. (2022). Induced-Fit Docking Enables Accurate
Free Energy Perturbation Calculations in Homology Models. J. Chem. Theory Comput..

[68] Coskun D., Lihan M., Rodrigues J. P. G. L.
M., Vass M., Robinson D., Friesner R. A., Miller E. B. (2024). Using AlphaFold
and Experimental Structures for the Prediction of the Structure and
Binding Affinities of GPCR Complexes via Induced Fit Docking and Free
Energy Perturbation. J. Chem. Theory Comput..

[69] Schrödinger Release 2025–4: Induced Fit Docking protocol, Glide; Schrödinger, LLC: New York, NY, 2024; 2024.

[70] Prime, Schrödinger, LLC: New York, NY, 2025; Vol. 2025.

[71] Schrödinger Release 2025–1: Desmond Molecular Dynamics System; D.E.S.R.: New York, NY, 2024; 2024.

[72] Maestro-Desmond Interoperability Tools; Schrödinger: New York, NY, 2025.

[73] Jorgensen W. L., Chandrasekhar J., Madura J. D., Impey R. W., Klein M. L. (1983). Comparison
of simple potential functions for simulating liquid water. J. Chem. Phys..

[74] Fischer L., Szellas D., Rådmark O., Steinhilber D., Werz O. (2003). Phosphorylation- and stimulus-dependent
inhibition of cellular 5-lipoxygenase
activity by nonredox-type inhibitors. FASEB
J..

[75] Koeberle A., Muñoz E., Appendino G. B., Minassi A., Pace S., Rossi A., Weinigel C., Barz D., Sautebin L., Caprioglio D. (2014). SAR studies on curcumin’s pro-inflammatory
targets: discovery of prenylated pyrazolocurcuminoids as potent and
selective novel inhibitors of 5-lipoxygenase. J. Med. Chem..

[76] Carione, P. Cytochrome P450-Mediated Metabolic Stability Assay in Liver Microsomes. In Cytochrome P450, Methods in Pharmacology and Toxicology, 2021; pp 229–242.

[77] Chen Y., Zhu F., Hammill J., Holbrook G., Yang L., Freeman B., White K. L., Shackleford D. M., O’Loughlin K.
G., Charman S. A. (2021). Selecting an anti-malarial clinical candidate
from two potent dihydroisoquinolones. Malaria
J..

[78] El-Kattan A., Poe J., Buchholz L., Thomas H., Brodfuehrer J., Clark A. (2008). The Use of 1-Aminobenzotriazole in
Differentiating the Role of CYPMediated
First Pass Metabolism and Absorption in Limiting Drug Oral Bioavailability:
A Case Study. Drug Metab. Lett..

[79] Rane A., Wilkinson G. R., Shand D. G. (1977). Prediction of hepatic extraction
ratio from in vitro measurement of intrinsic clearance. J. Pharmacol. Exp. Ther..

[80] Kerins A., Butler P., Riley R., Koszyczarek M., Smith C., Cruickshank F., Madgula V., Naik N., Redinbo M. R., Wilson I. D. (2024). In vitro
and in vivo studies on the
metabolism and pharmacokinetics of the selective gut microbial β-glucuronidase
targeting compound Inh 1. Xenobiotica.

[81] Yang X., Liu A., Yang L., Wen T., Wang J., Shi J., Zhou H., Chen Z., Lei M., Zhu Y. (2022). Preclinical
Pharmacokinetics, Tissue Distribution and in vitro Metabolism of FHND6091,
a Novel Oral Proteasome Inhibitor. Drug Design,
Dev. Ther..

[82] Wang Q., Liu H., Slavsky M., Fitzgerald M., Lu C., O’Shea T. (2019). A high-throughput
glutathione trapping assay with combined high sensitivity and specificity
in high-resolution mass spectrometry by applying product ion extraction
and data-dependent neutral loss. J. Mass Spectrom..

[83] Carullo G., Falbo F., Tudino V., Song J., Fava M., Fontana A., Koch A., Heiß A., Erlenbach-Wuensch K., Panzeca G. (2026). Targeting
Class I Histone
Deacetylases Triggers Antitumor Responses in Colorectal Cancer In
Vitro and In Vivo. J. Med. Chem..

[84] de
Bruyn Kops C., Šícho M., Mazzolari A., Kirchmair J. (2021). GLORYx: Prediction of the Metabolites Resulting from
Phase 1 and Phase 2 Biotransformations of Xenobiotics. Chem. Res. Toxicol..

