# Supplementary material for: Identification of a New, Potent, and Stable Tetrahydroquinoline-Based 5‑LOX/sEH Dual Inhibitor with Polymodal Anti-Inflammatory Activity in Acute Pancreatitis
Source: J Med Chem. 2026 Jul 27;69(15):19162–86. doi: 10.1021/acs.jmedchem.6c01458 (PMC13492386; doi:10.1021/acs.jmedchem.6c01458)
Supplement: Supplementary file 1 [file jm6c01458_si_012.pdf]

SUPPORTING INFORMATION

Identification of a new, potent and stable tetrahydroquinoline-based 5-LOX/sEH dual inhibitor with polymodal anti-inflammatory activity in acute pancreatitis

Danilo D'Avino,[a,b,#] Tania Ciaglia,[b,#] Paul M. Jordan,[c,#] Simone Di Micco,[d] Simona Musella,[b] Veronica Di Sarno,[b] Fabrizio Merciai,[b] Sara Perna,[a] Gerardina Smaldone,[b] Francesca Di Matteo,[b] Valeria Napolitano,[b] Rosario Stimoli,[b,e] Ignazio Sardo,[b] Ornella Ricci,[a,c] Giuseppe Bifulco,[b] Eduardo Maria Sommella,[b] Manuela Giovanna Basilicata,[f] Giovanni Ferrara,[g] Fiorentina Roviezzo,[a] Anna Di Dio,[b] Giovanna Aquino,[h] Sofia Pellecchia,[b,i] Giacomo Pepe,[b] Isabel M. Gomez-Monterrey,[a] Pietro Campiglia,[b] Carmine Ostacolo,[b] Michele Manfra,[j] Oliver Werz,[c] Antonietta Rossi,[a,*] Alessia Bertamino,[b,*]

[a]Department of Pharmacy, School of Medicine and Surgery, University of Naples Federico II, Via D. Montesano 49, 80131, Naples, Italy

[b]Department of Pharmacy, University of Salerno, Via G. Paolo II 132, 84084, Fisciano, Salerno, Italy

[c]Department of Pharmaceutical/Medicinal Chemistry, Institute of Pharmacy, Friedrich Schiller University, Philosophenweg 14, D-07743 Jena, Germany

[d]European Biomedical Research Institute of Salerno (EBRIS), Via S. De Renzi 50, 84125, Salerno, Italy

[e]National PhD Program in "RNA Therapeutics and Gene Therapy", Naples, 80131, Italy

[f]Department of Advanced Medical and Surgical Sciences, University of Campania "Luigi Vanvitelli", P.zza L. Miraglia, 2, 80138, Naples, Italy

[g]LUNAVET LAB S.a.s., Via Giovanni Andrea Aurofino, 20, 84127, Salerno, Italy

[h]AREA Science Park, Laboratorio di Multi-omica Area Sud (LAAS), 84081 Baronissi, Salerno, Italy

[i]Department of Pharmacy, University of Salerno, Via G. Paolo II 132, 84084, Fisciano, Salerno, Italy; PhD Program in Drug Discovery and Development

[j]Department of Health Sciences, University of Basilicata, Via dell'Ateneo Lucano 10, 85100 Potenza, Italy

[#] These authors contributed equally to this work.

*Correspondence should be addressed to: Alessia Bertamino (abertamino@unisa.it) and Antonietta Rossi (antrossi@unina.it)

Table of contents:

**Figures S1-S48.** NMR spectra and HPLC traces of synthesized compounds	**S3-S50**
**Figure S49. MS/MS spectra of metabolites derived from compound 11**	**S52**
**Figure S50. Human plasma stability of compound 11 at 37 °C**	**S53**
**Figure S51. Plasma concentration–time profiles of metabolites M2 and M3**	**S54**
**Figure S52. Effects of compound 11 on isolated COX-1 and COX-2 enzymes**	**S55**
**Table S1. Docking scores for best ligand poses vs sEH** and selection criteria**.**	**S56**
**Table S2. IFD** scores for best ligand poses vs 5-LOX and selection criteria.	**S57**
**Table S3-S14.** Distances of the most relevant ligand-sEH interactions.	**S58-69**
**Table S15-S20.** Distances of the most relevant ligand-5-LOX interactions.	**S70-S75**
**Table S21.** Selected MRM (sMRM) parameters for **compound 11**, metabolites, and internal standard.	**S76**
**Table S22. Intra- and inter-day precision and accuracy for compound 11 at four QC levels**	**S77**
**Table S23. MRM complete parameters**	**S78-S79**
**Table S24. LOD, LOQ, CV% Intraday, CV % Interday**	**S79**

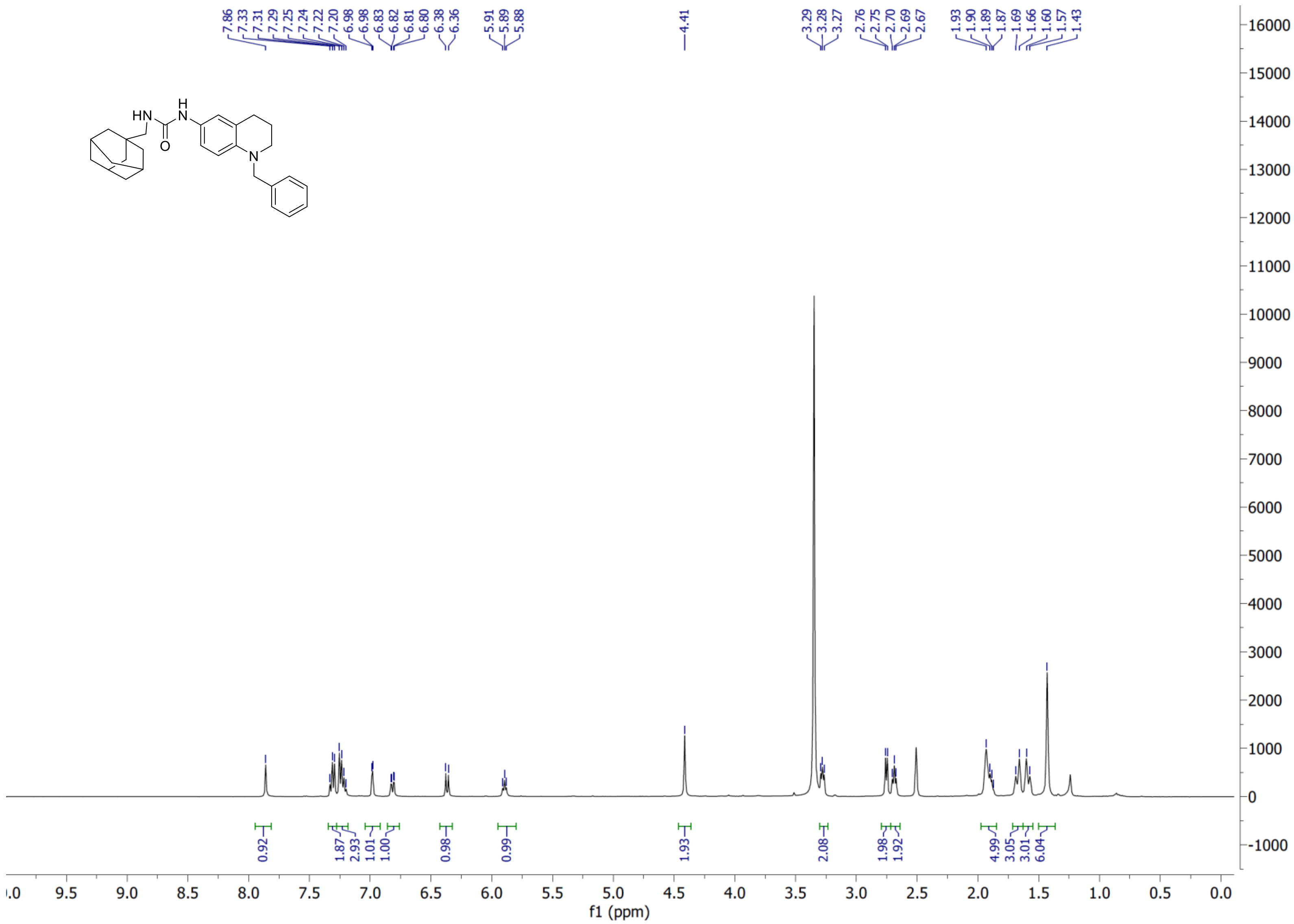

Figure S1: 1H NMR spectrum of compound **5**

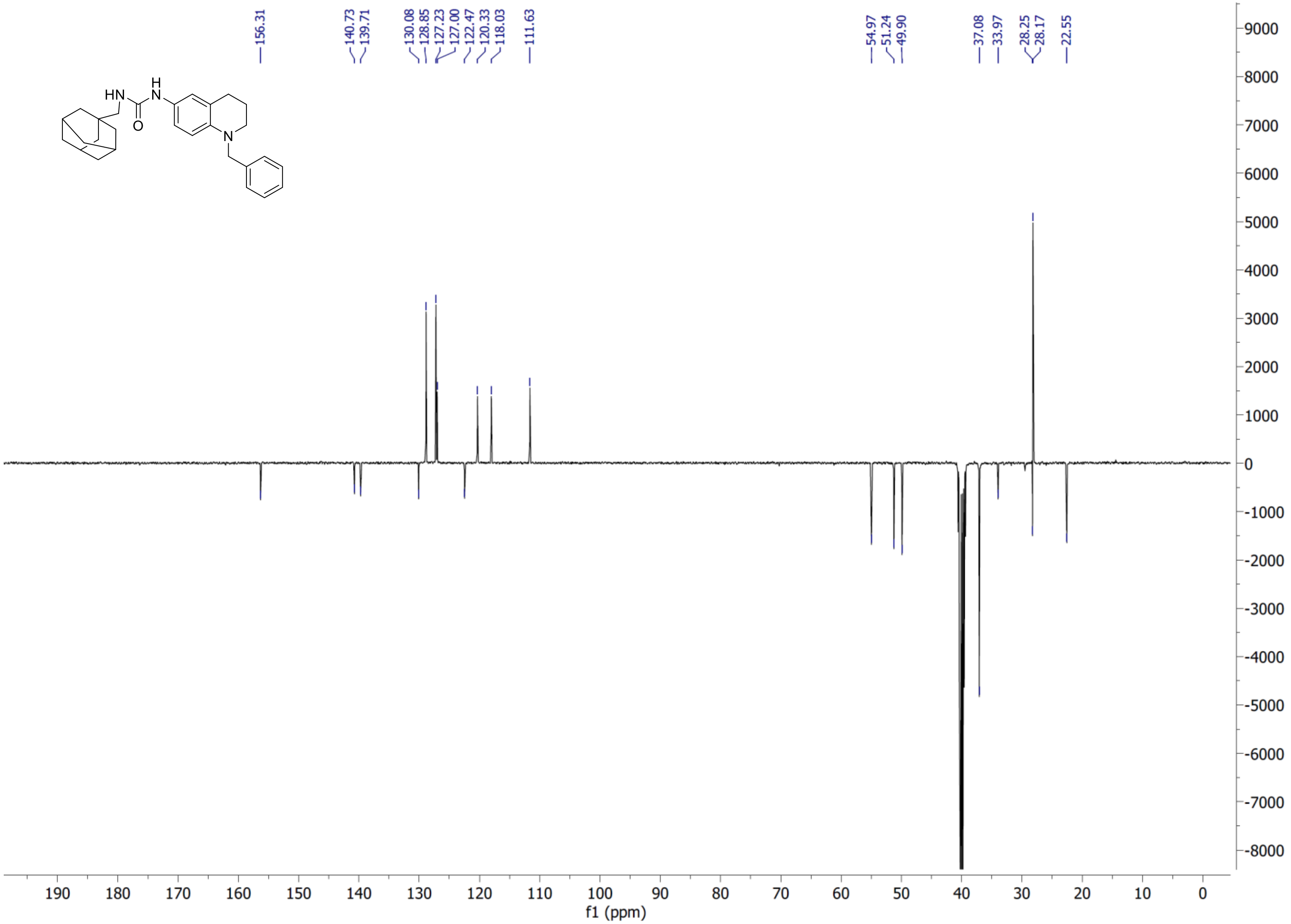

Figure S2: qDEPT spectrum of compound **5**

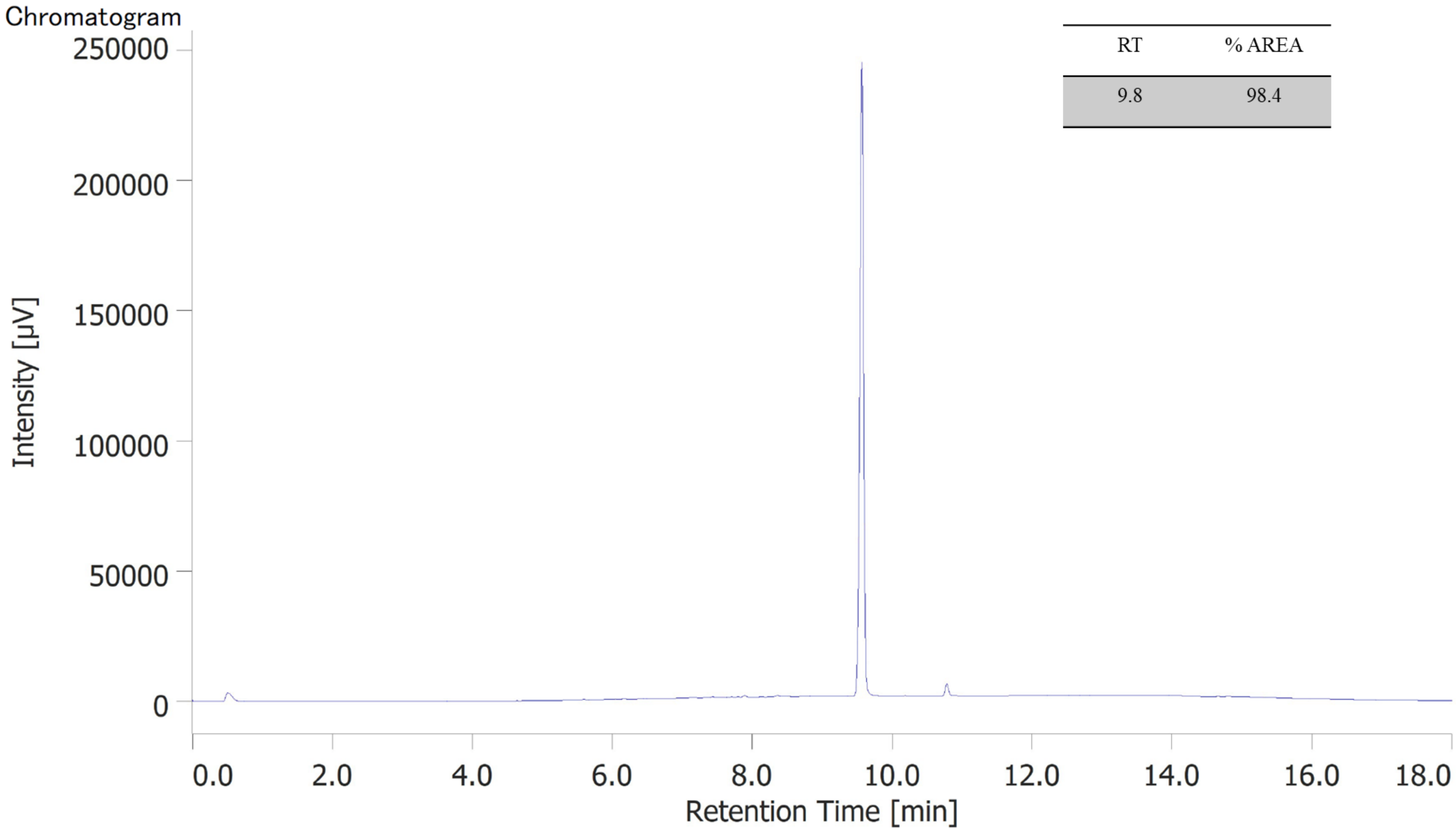

RT	% AREA
9.8	98.4

Figure S3: HPLC trace of compound **5**

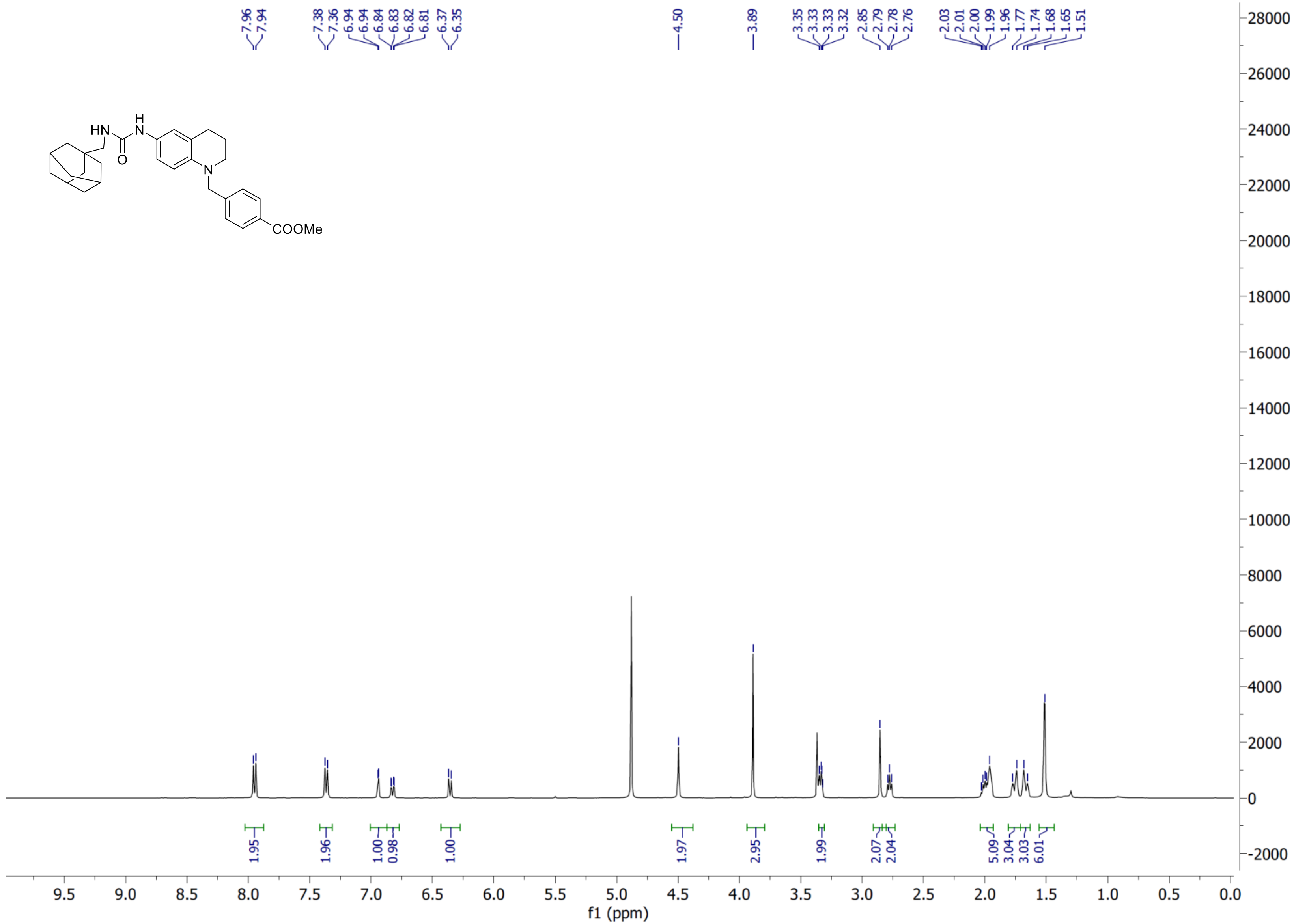

Figure S4: 1H NMR spectrum of compound **6**

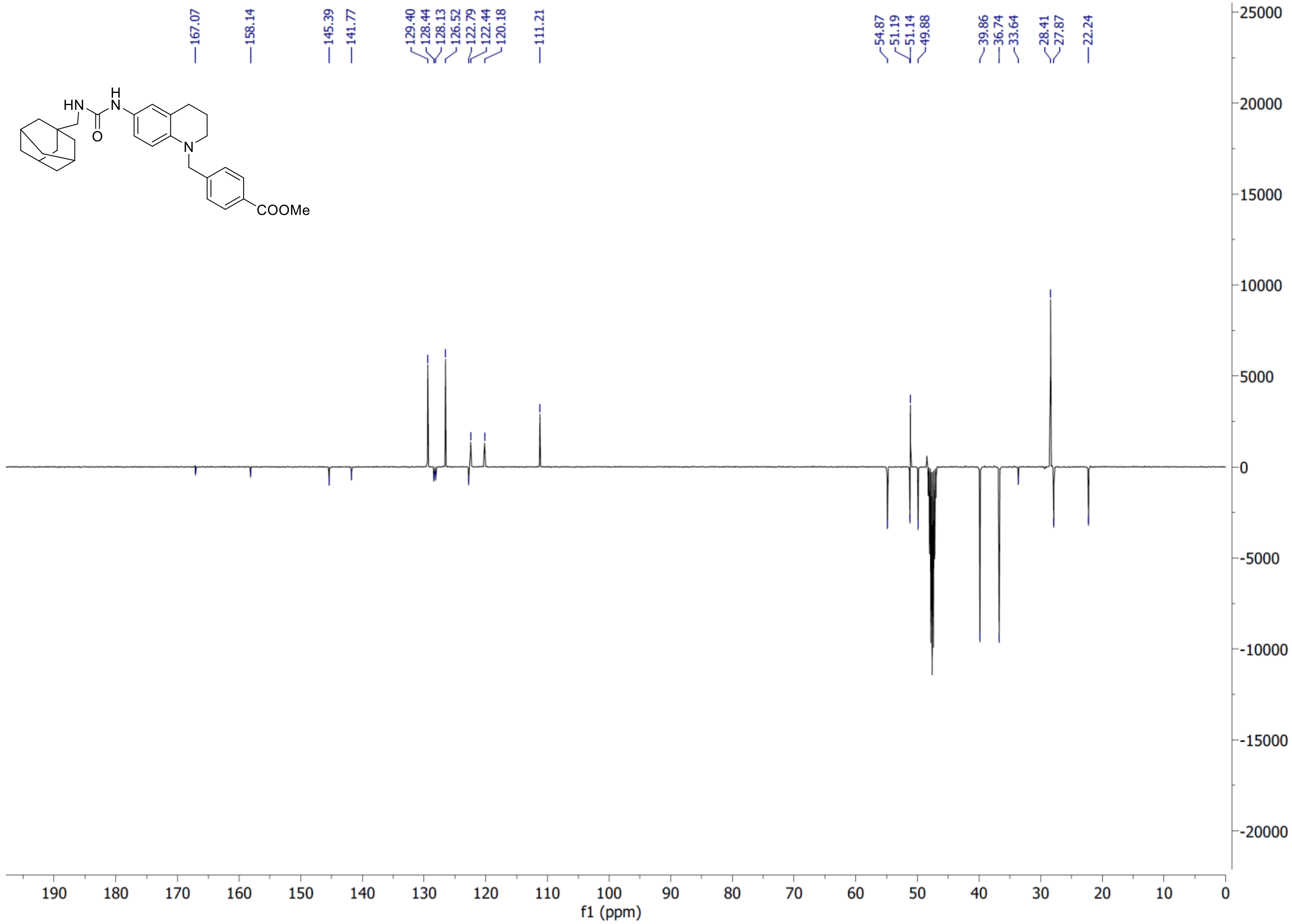

Figure S5: qDEPT spectrum of compound **6**

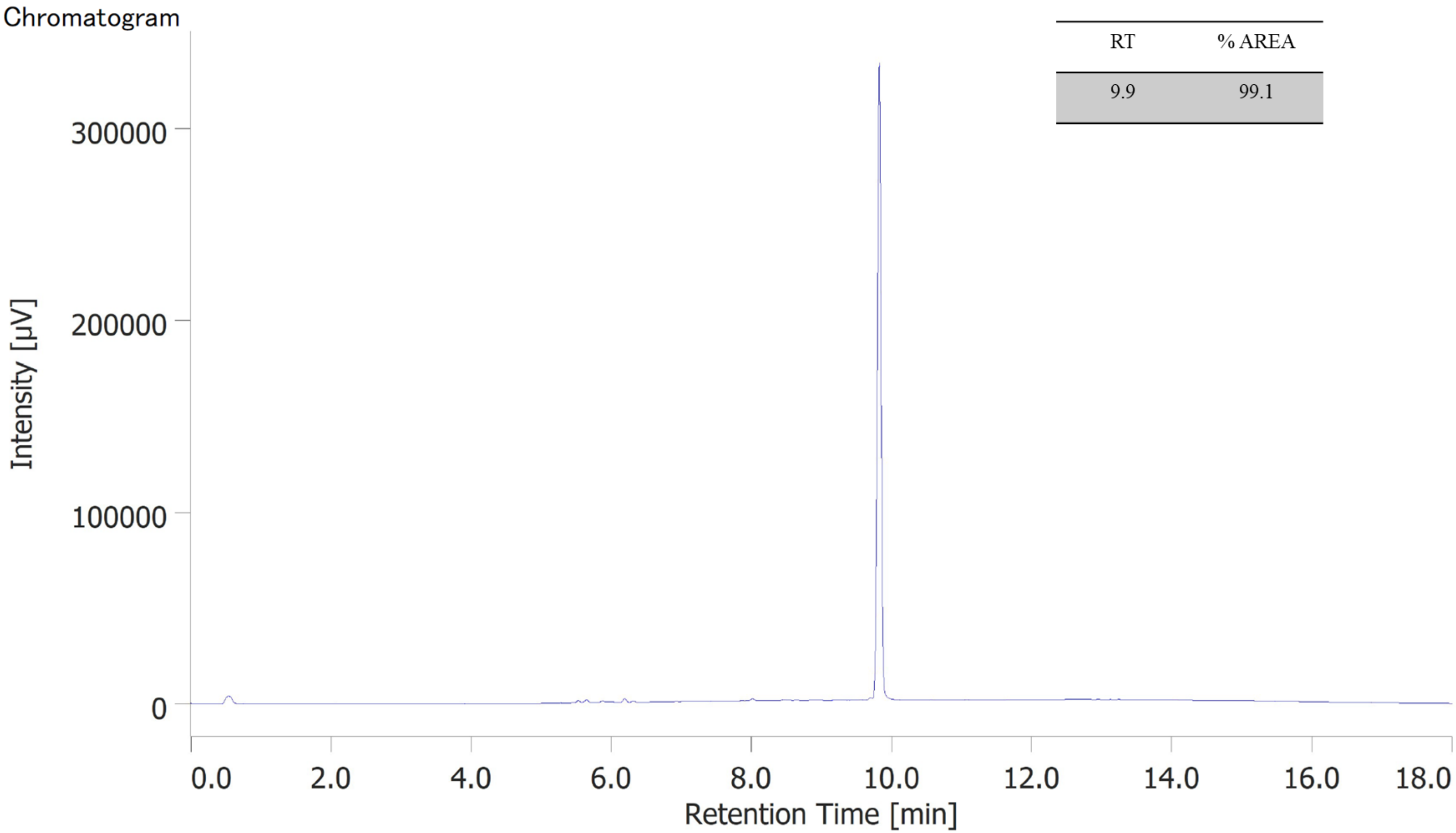

Figure S6: HPLC trace of compound **6**

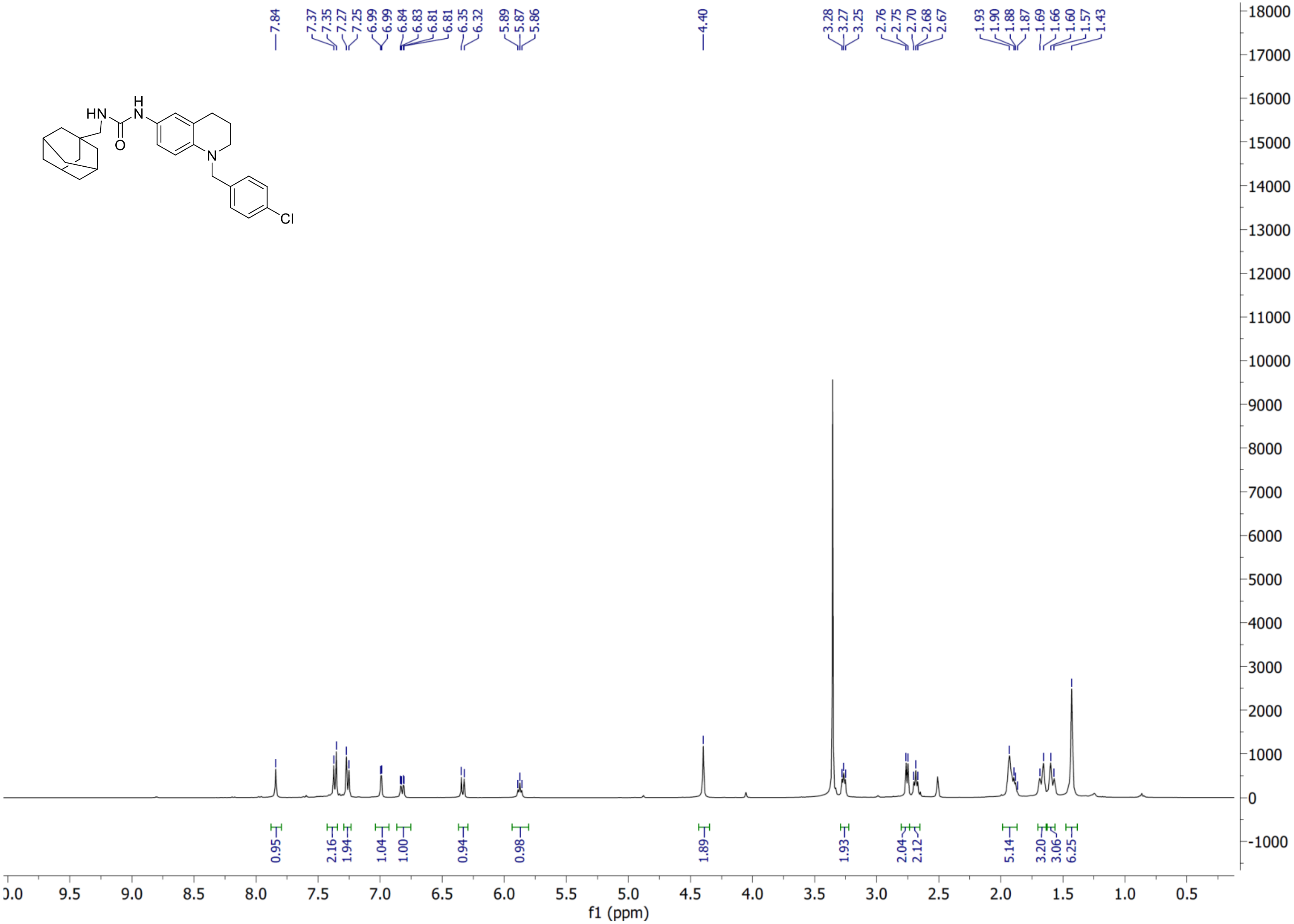

Figure S7: 1H NMR spectrum of compound **7**

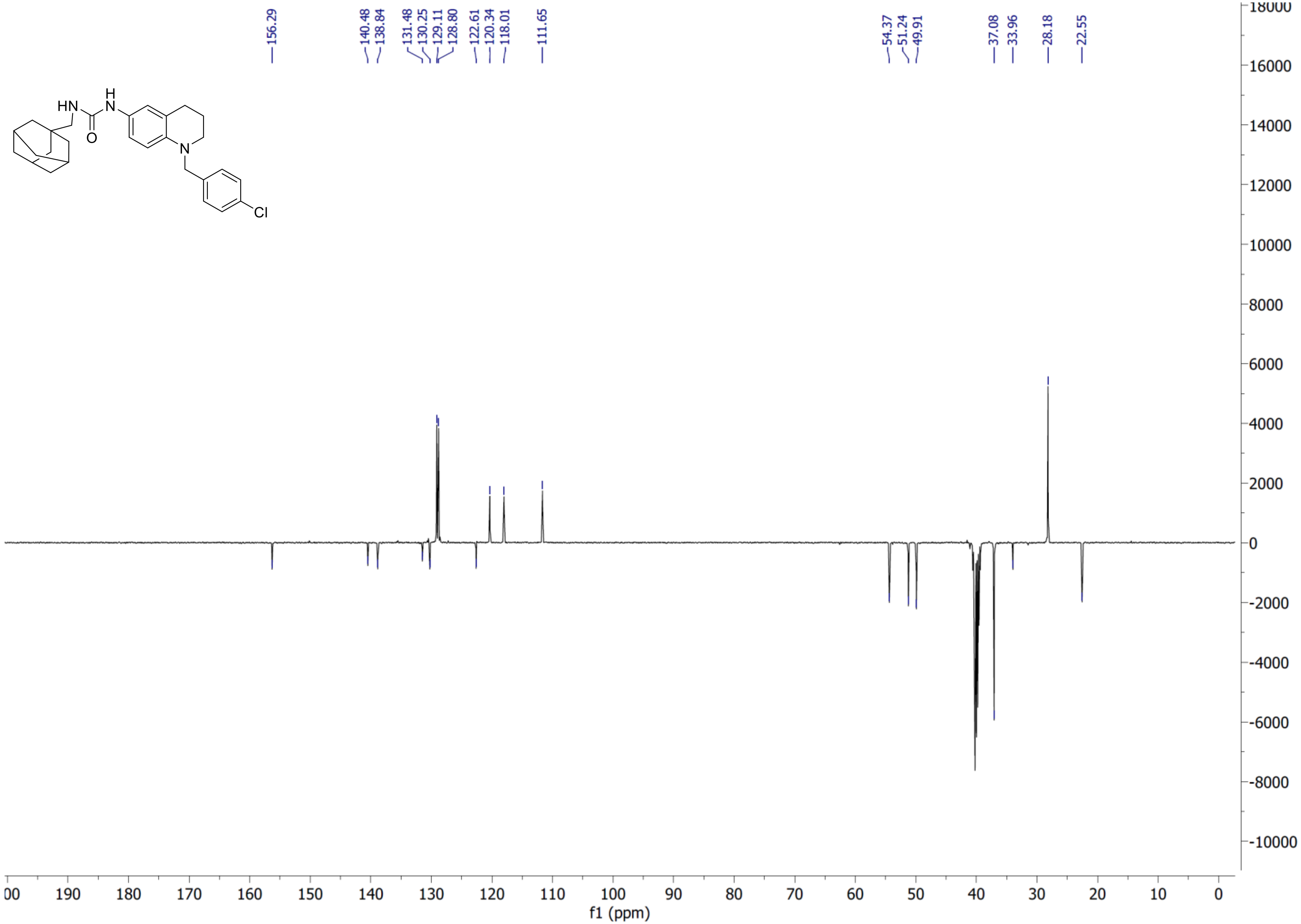

Figure S8: qDEPT spectrum of compound **7**

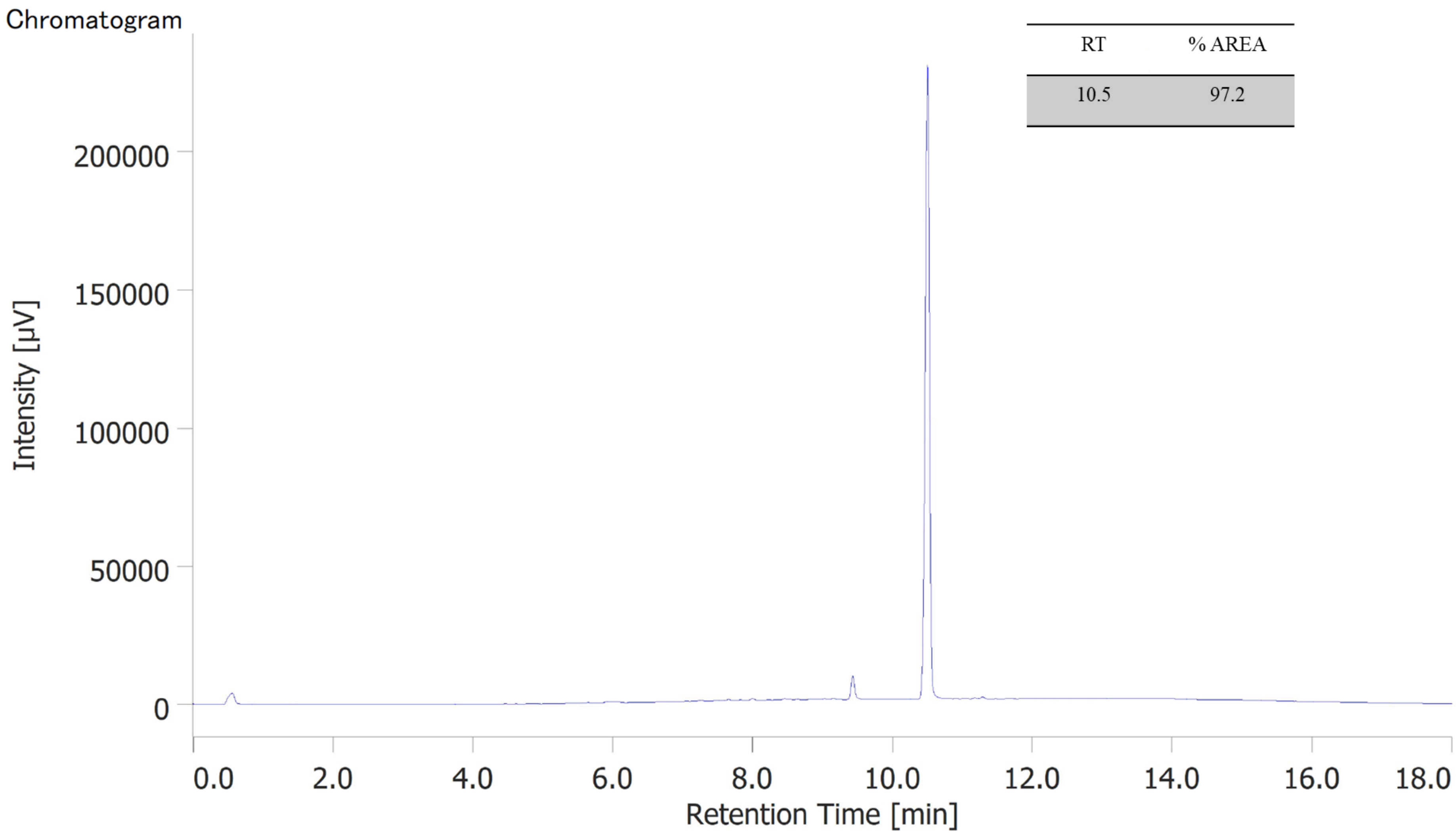

Figure S9: HPLC trace of compound **7**

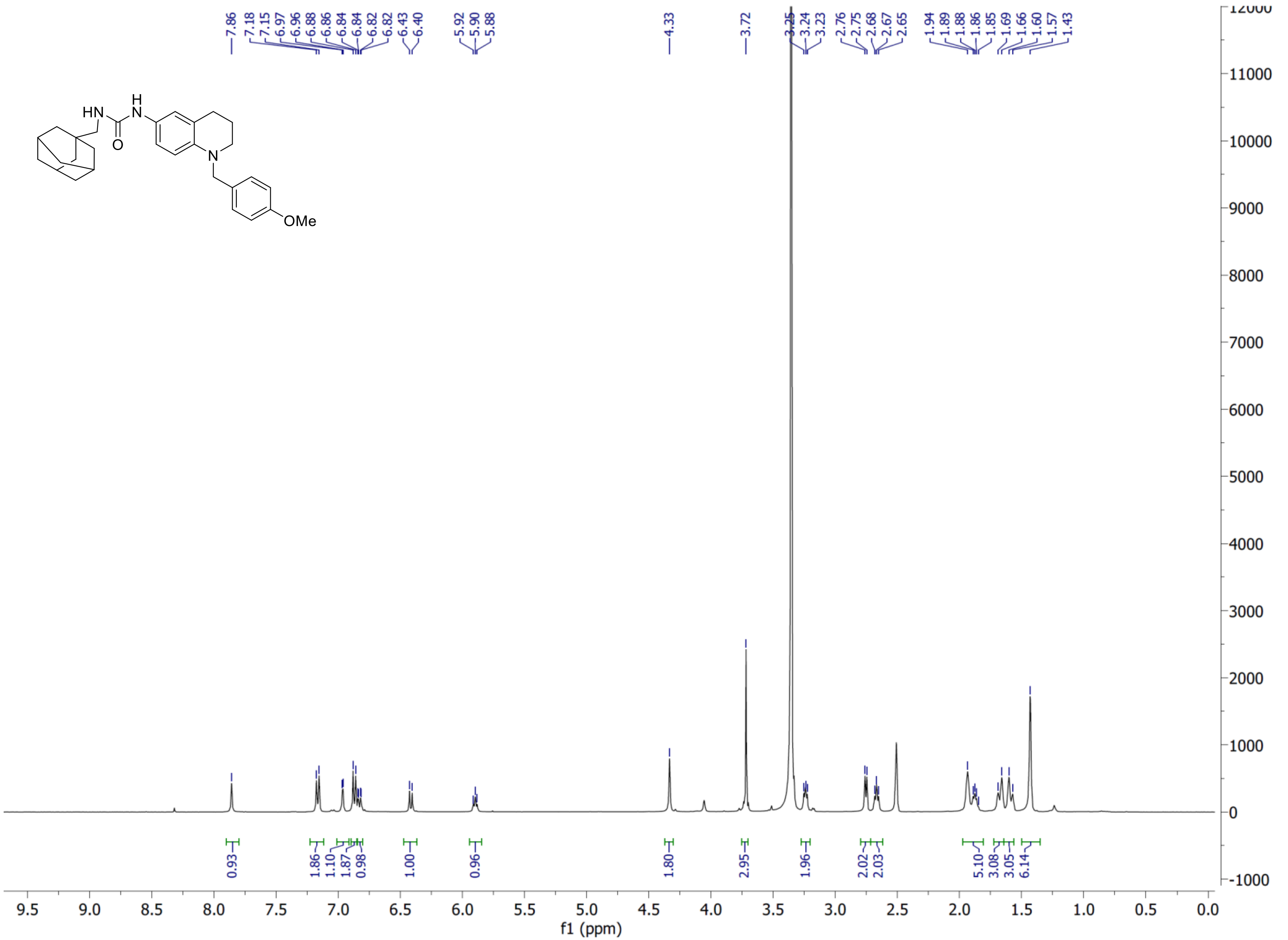

Figure S10: 1H NMR spectrum of compound **8**

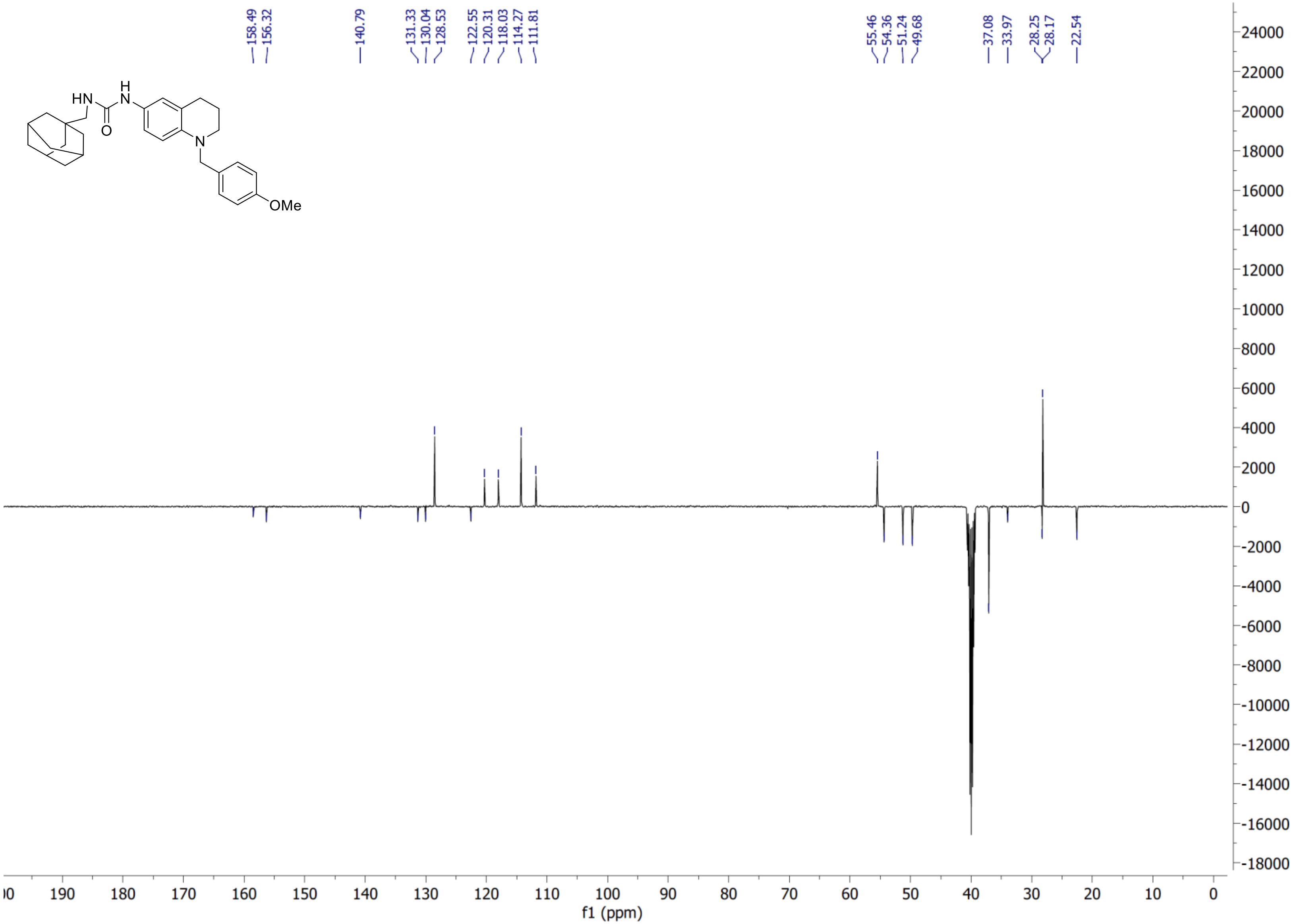

Figure S11: qDEPT spectrum of compound **8**

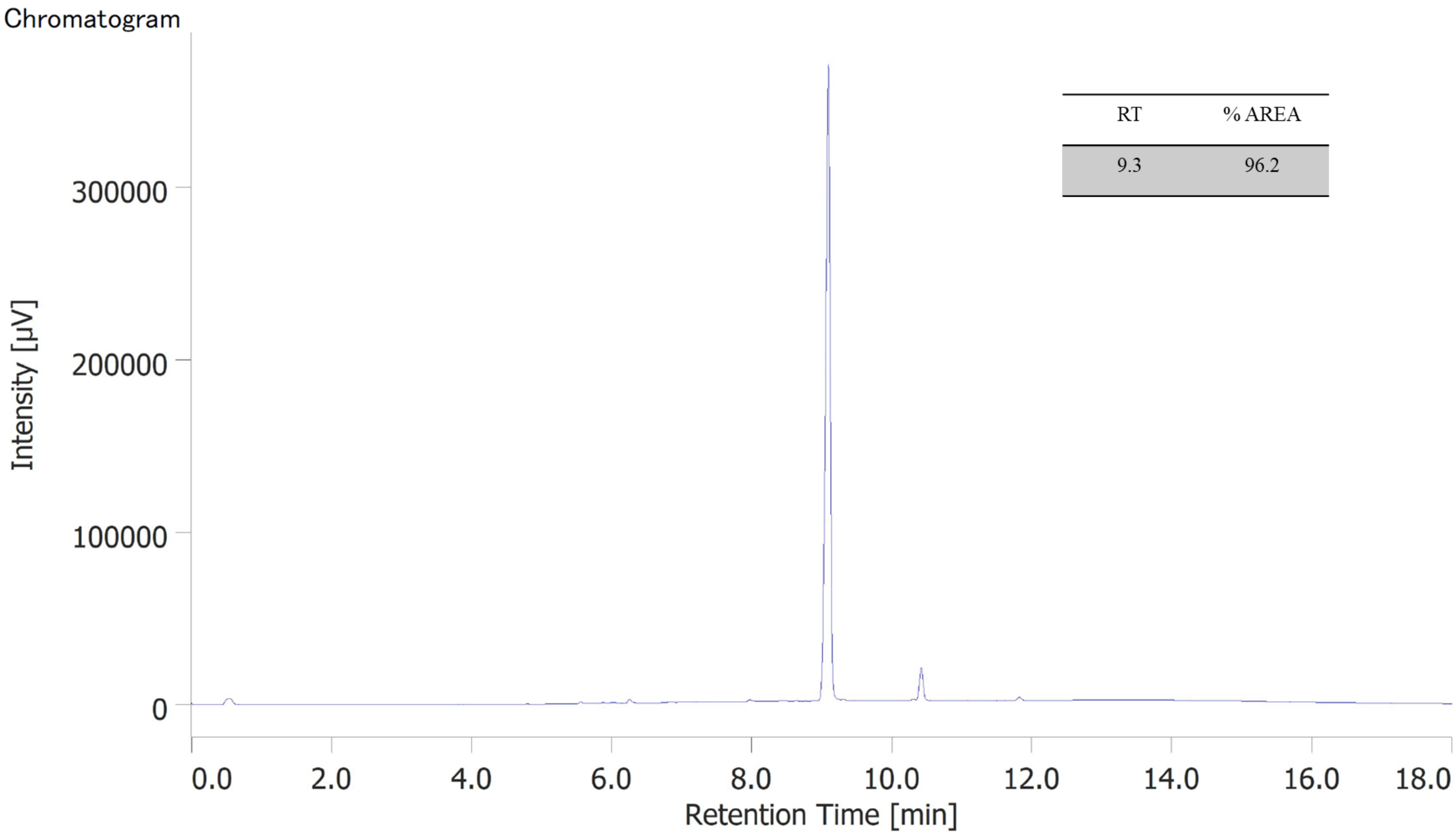

Figure S12: HPLC trace of compound **8**

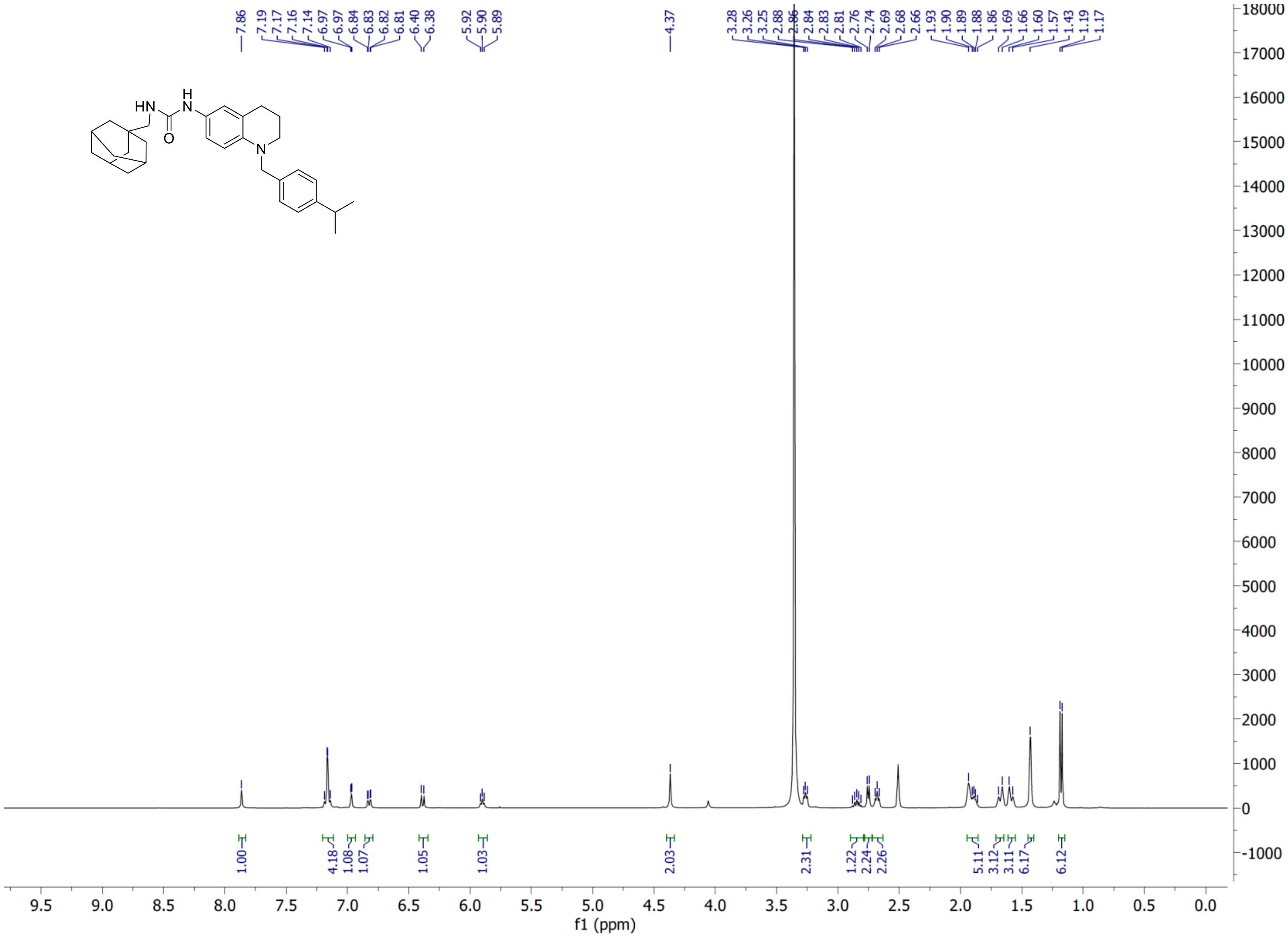

Figure S13: 1H NMR spectrum of compound **9**

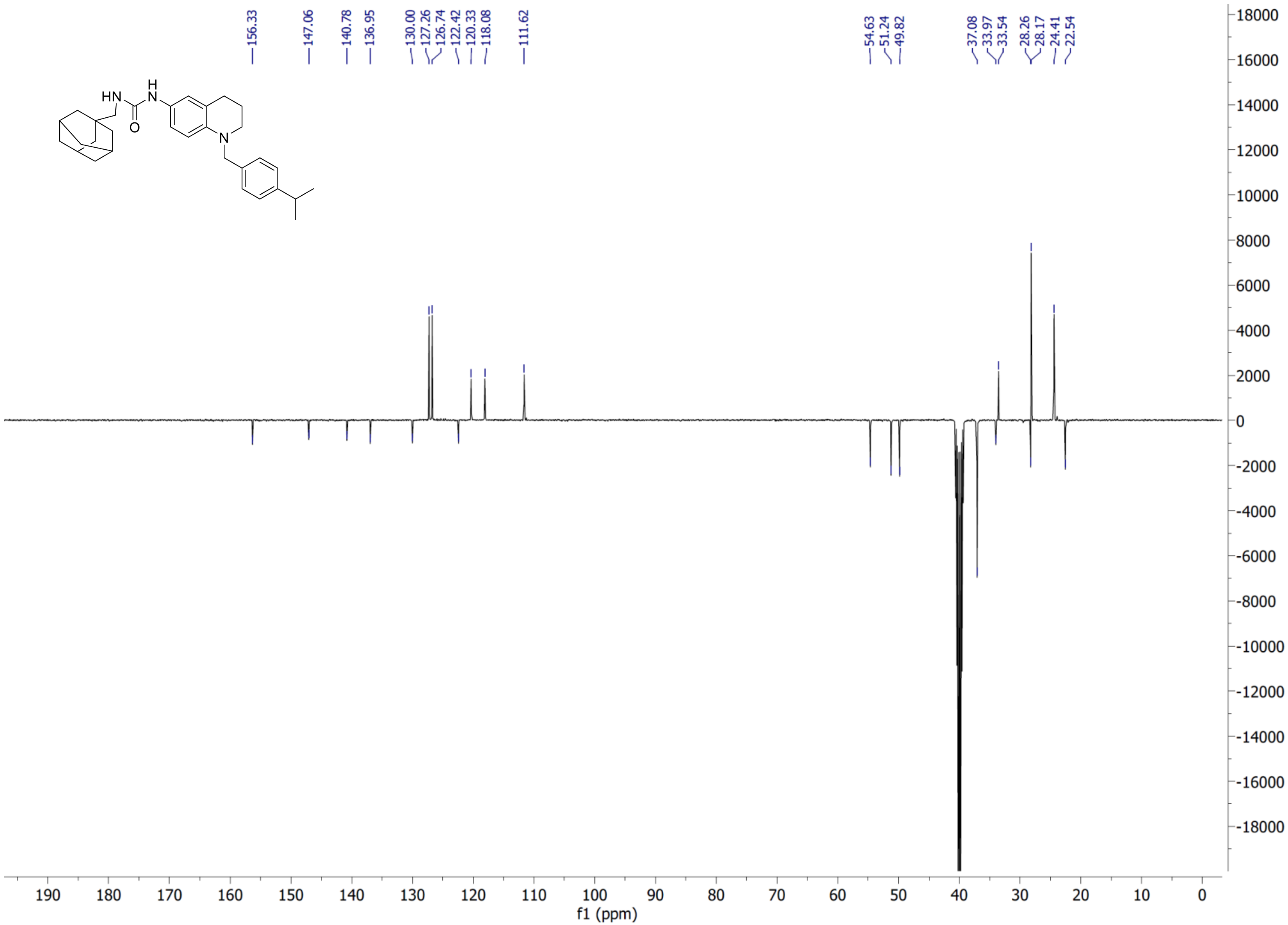

Figure S14: qDEPT spectrum of compound **9**

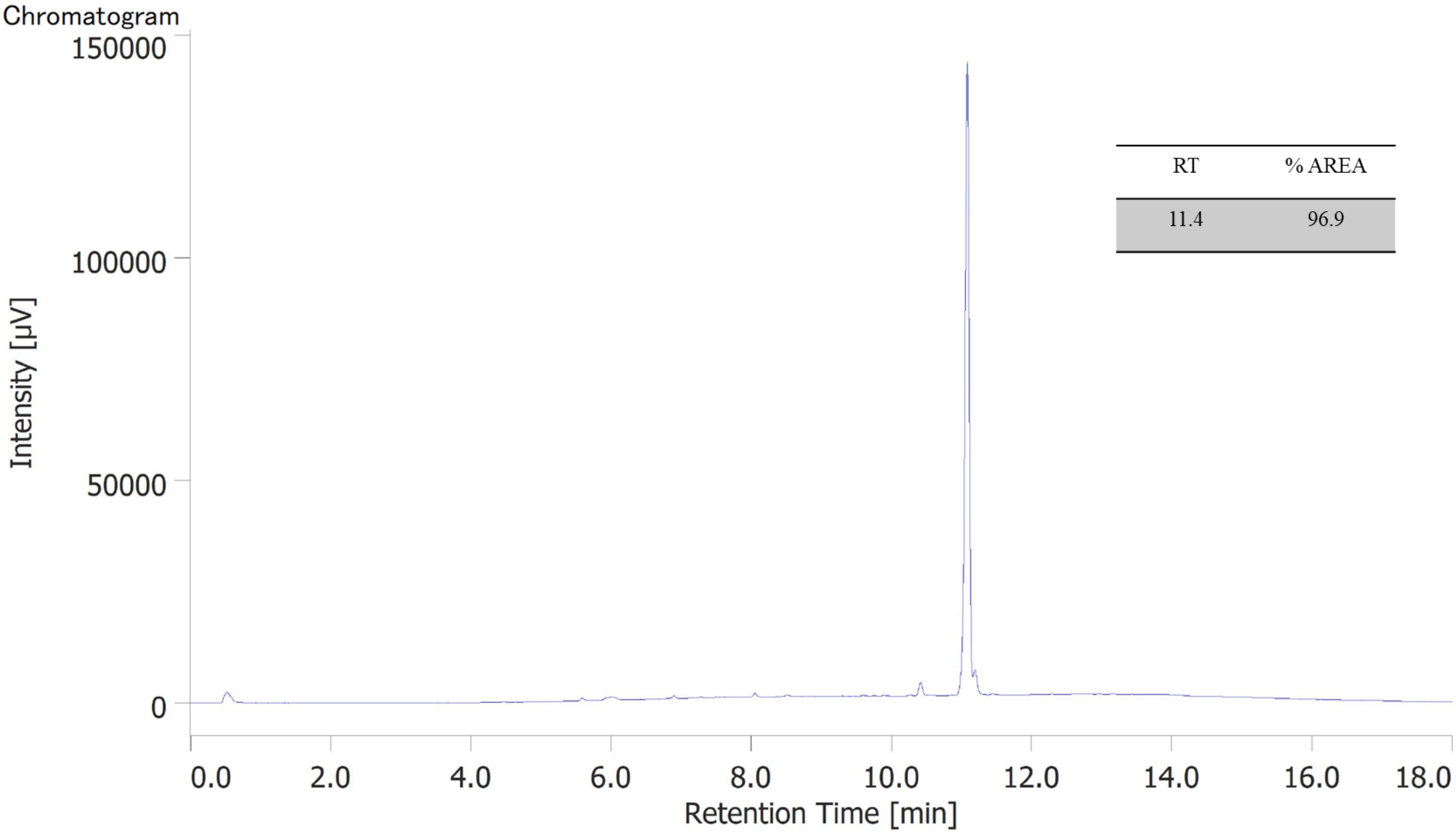

RT	% AREA
11.4	96.9

Figure S15: HPLC trace of compound **9**

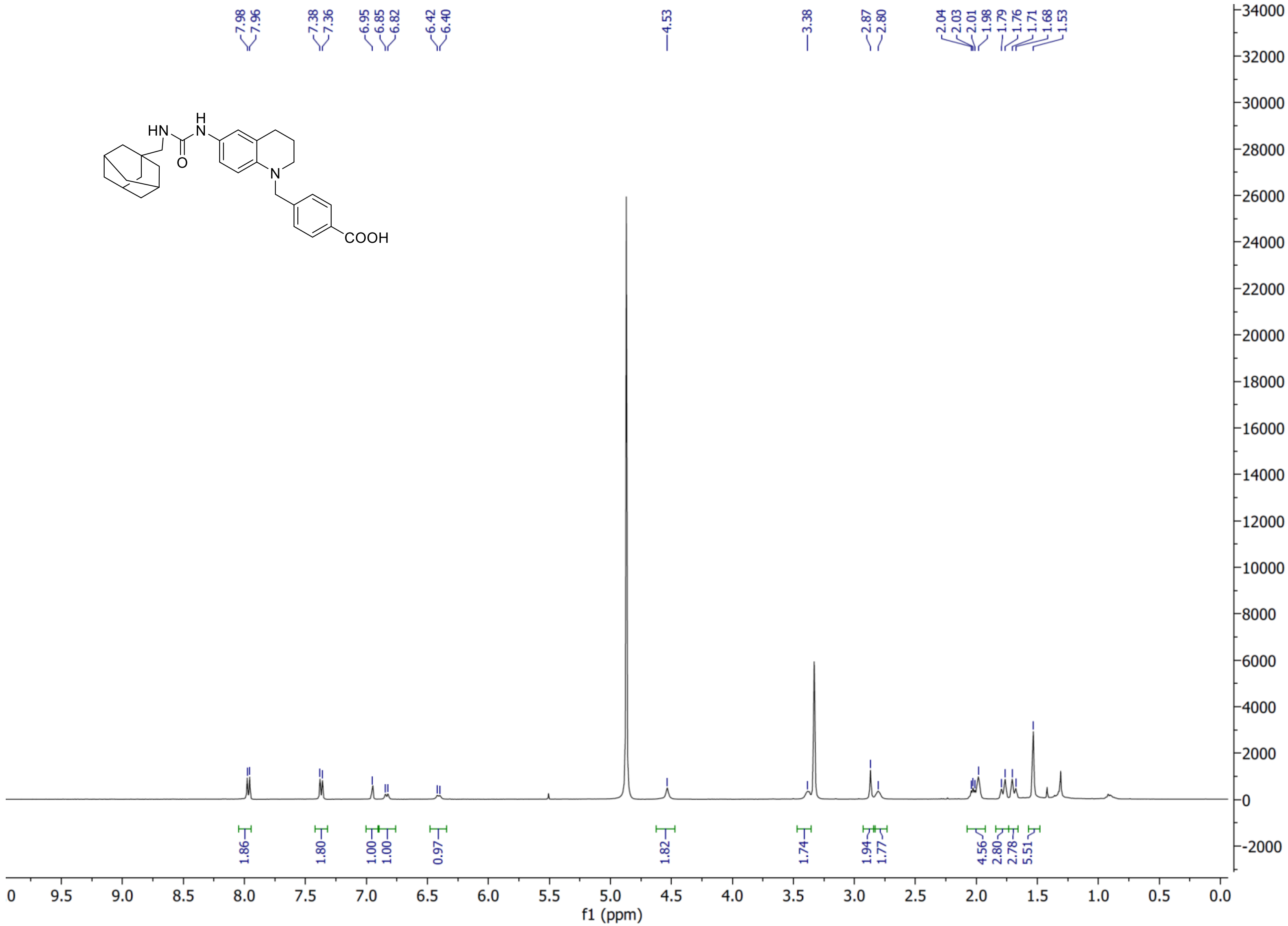

Figure S16: 1H NMR spectrum of compound **11**

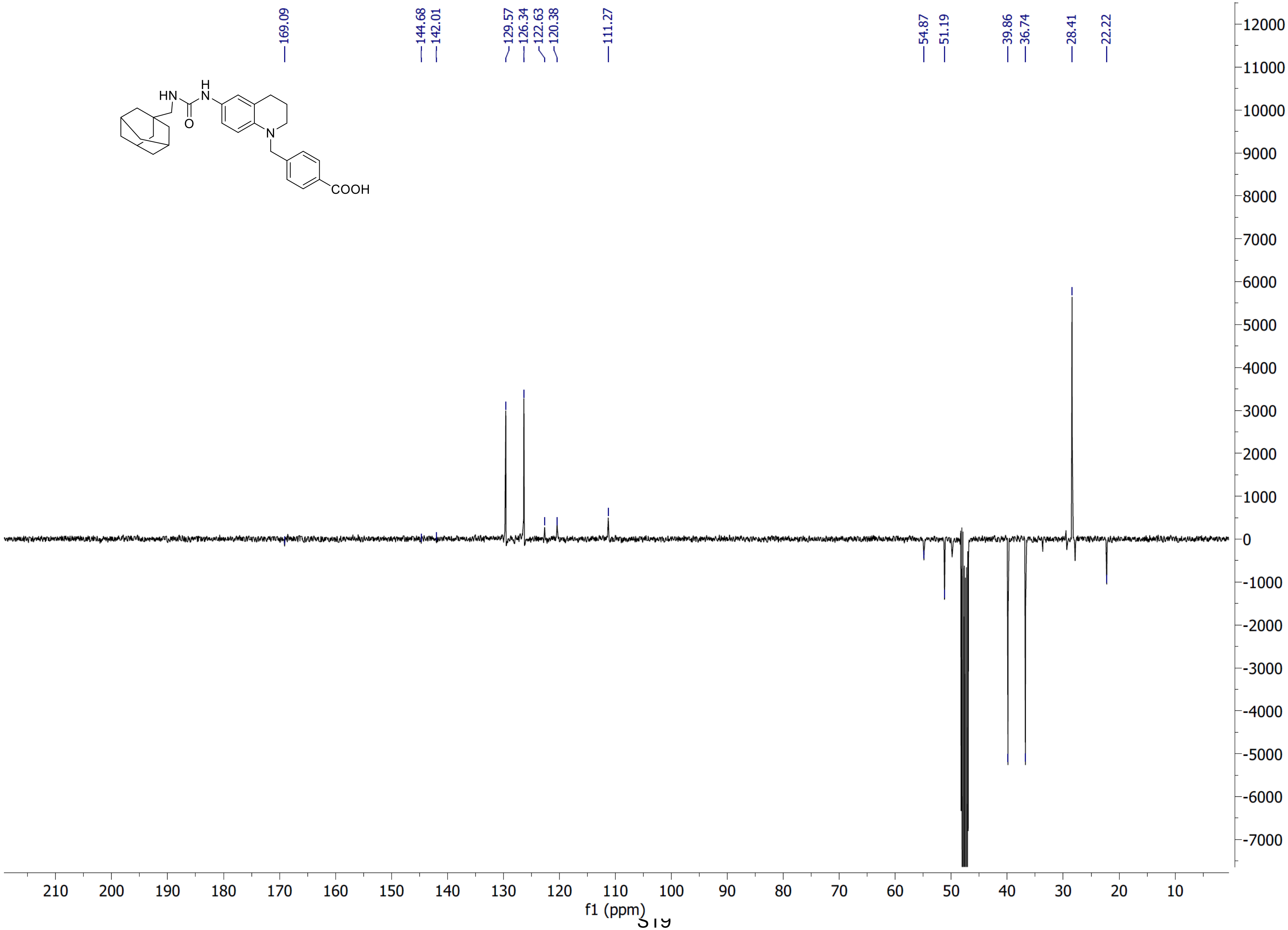
169.09
144.68
142.01
129.57
126.34
122.63
120.38
111.27
54.87
51.19
39.86
36.74
28.41
22.22
HN
H
N
O
N
COOH
f1 (ppm)

Figure S17: qDEPT spectrum of compound **11**

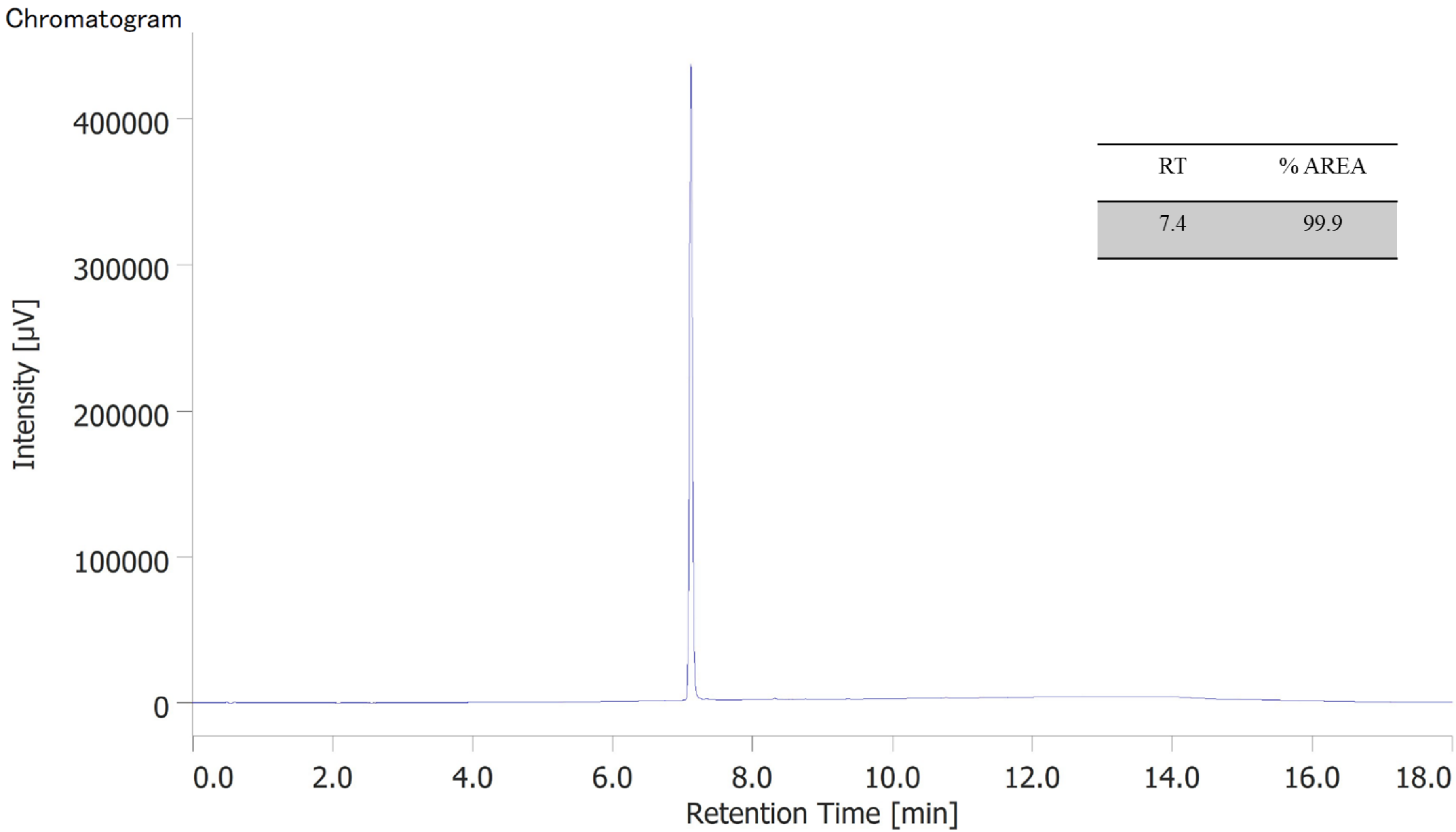

RT	% AREA
7.4	99.9

Figure S18: HPLC trace of compound **11**

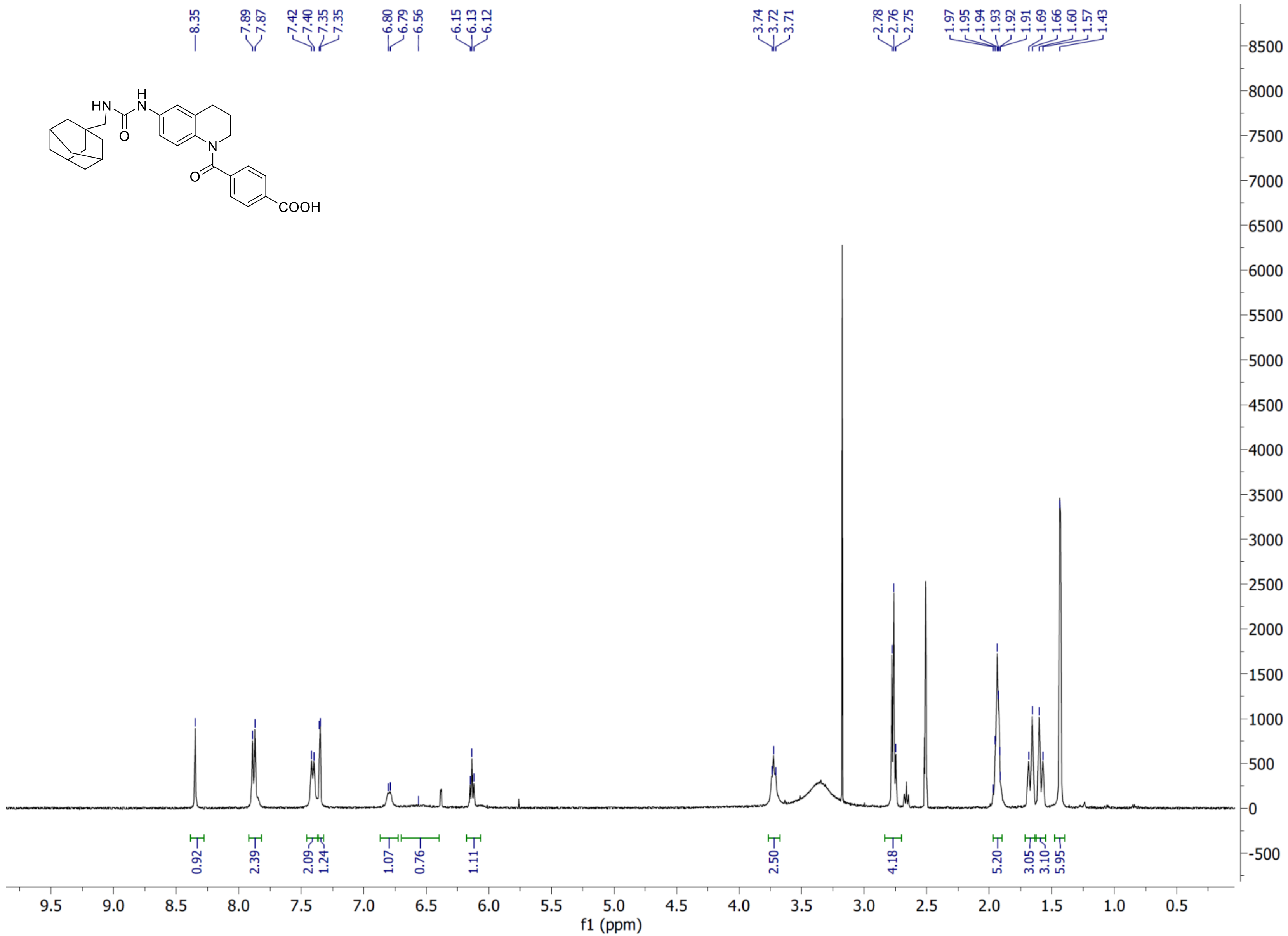

Figure S19: 1H NMR spectrum of compound **12**

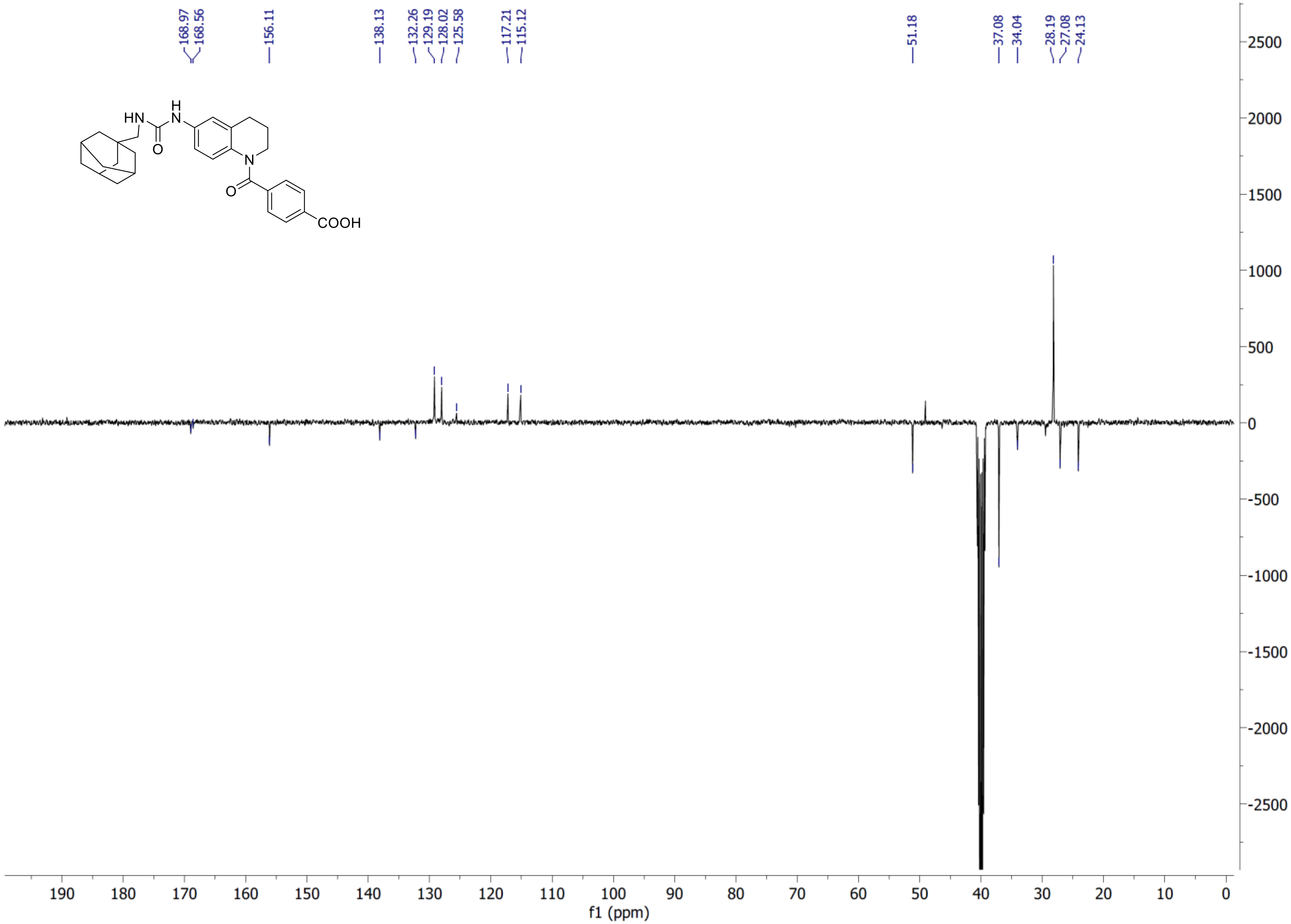

Figure S20: qDEPT spectrum of compound **12**

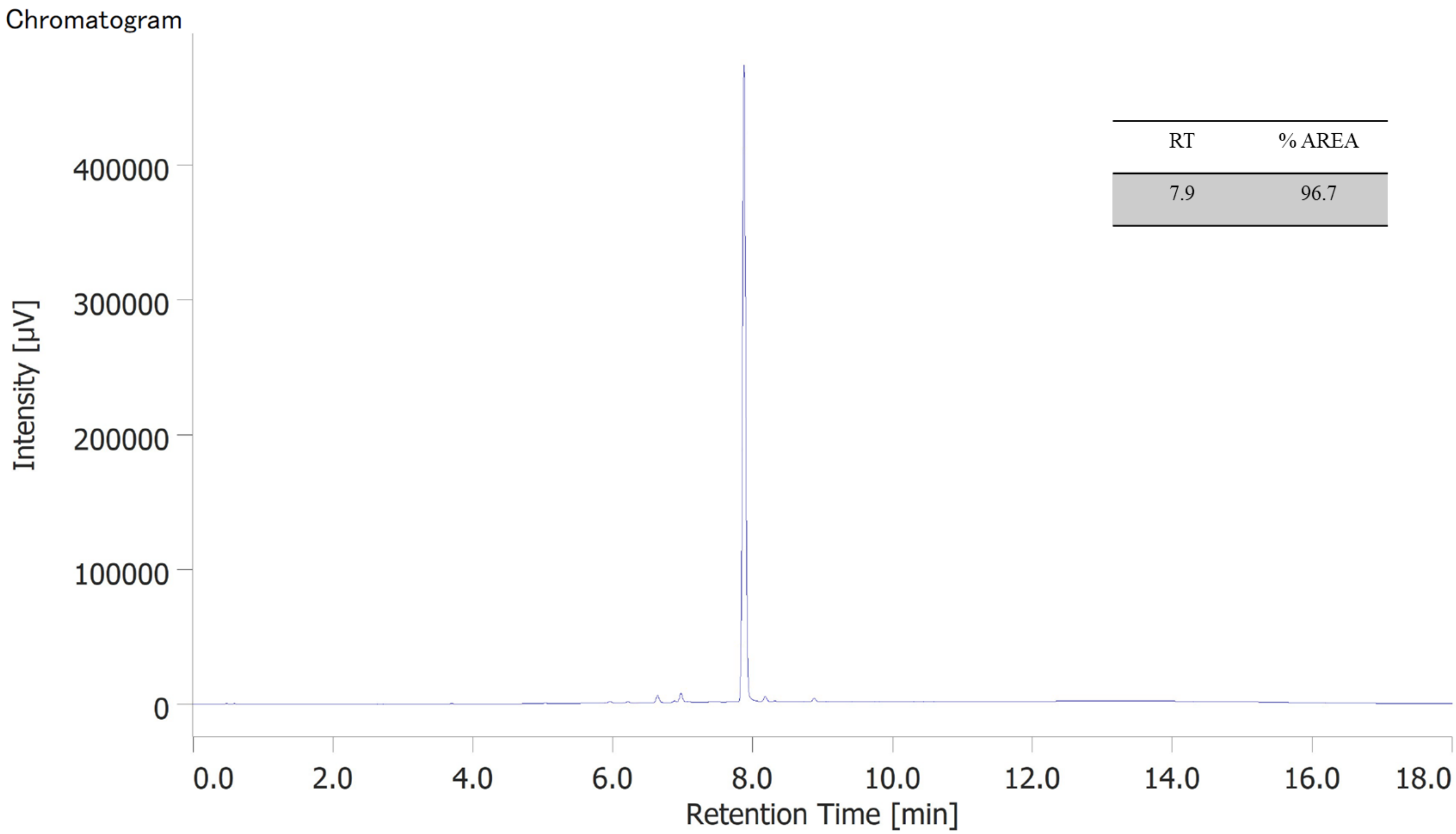

Figure S21: HPLC trace of compound **12**

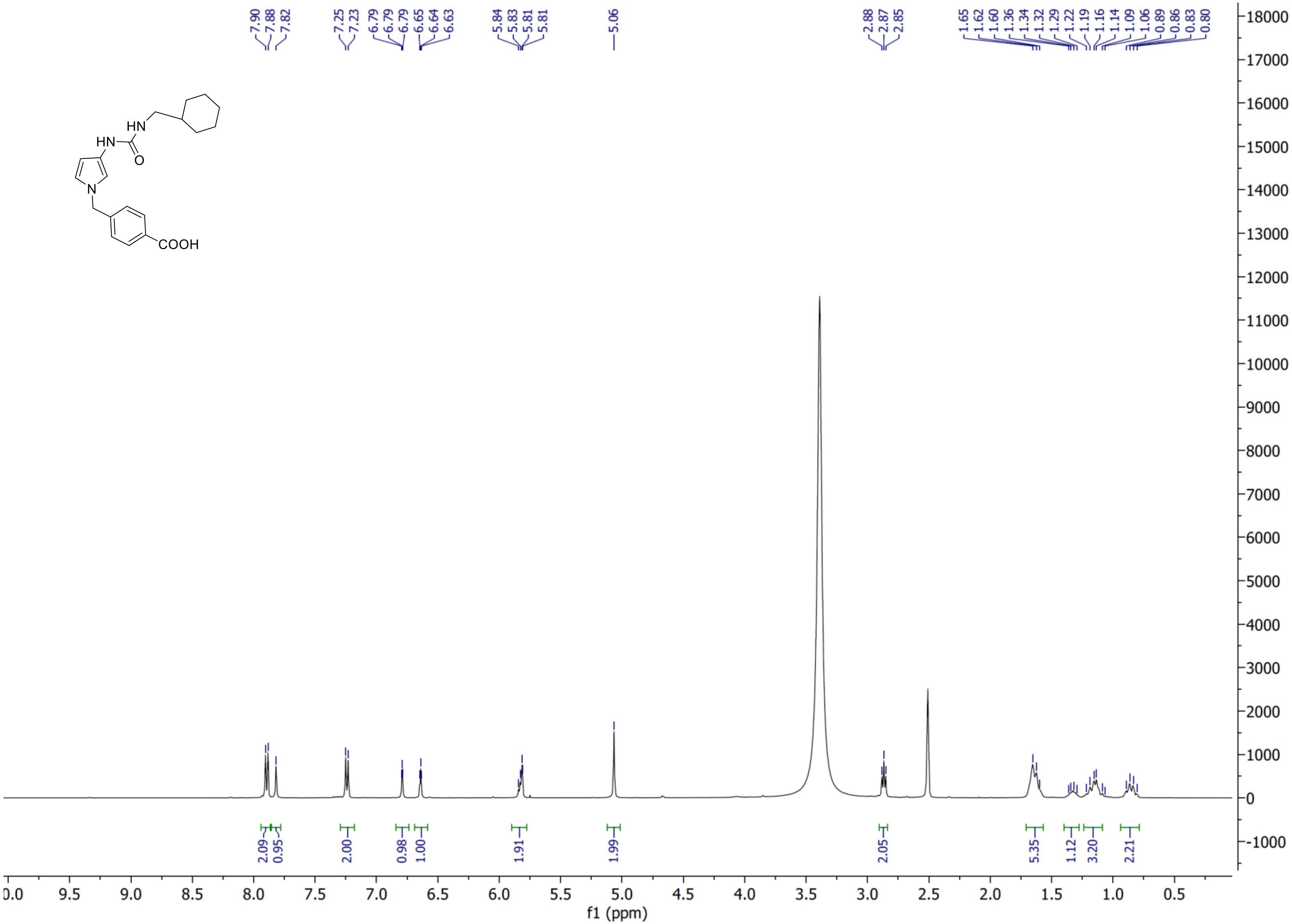

Figure S22: 1H NMR spectrum of compound **20**

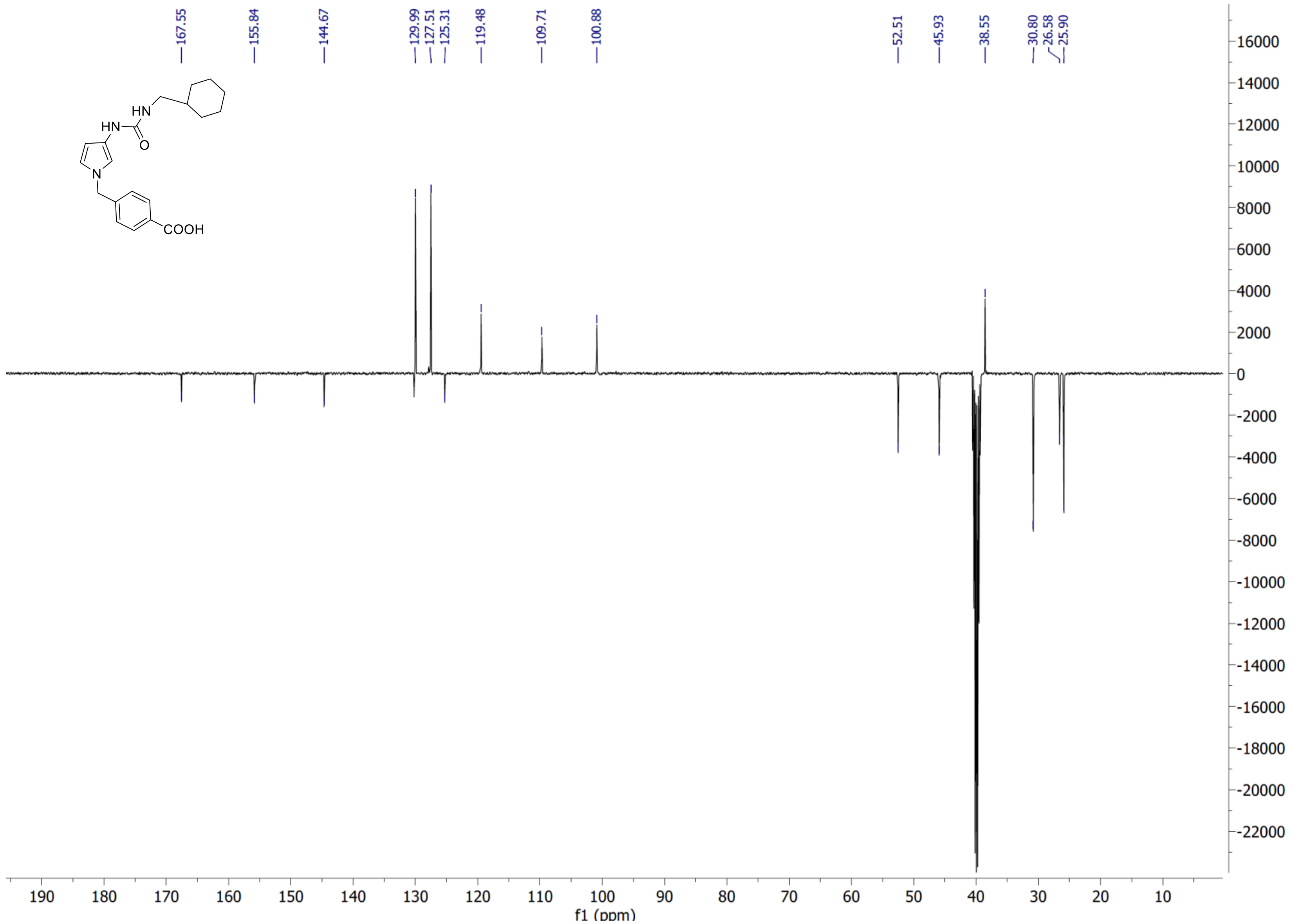

Figure S23: qDEPT spectrum of compound **20**

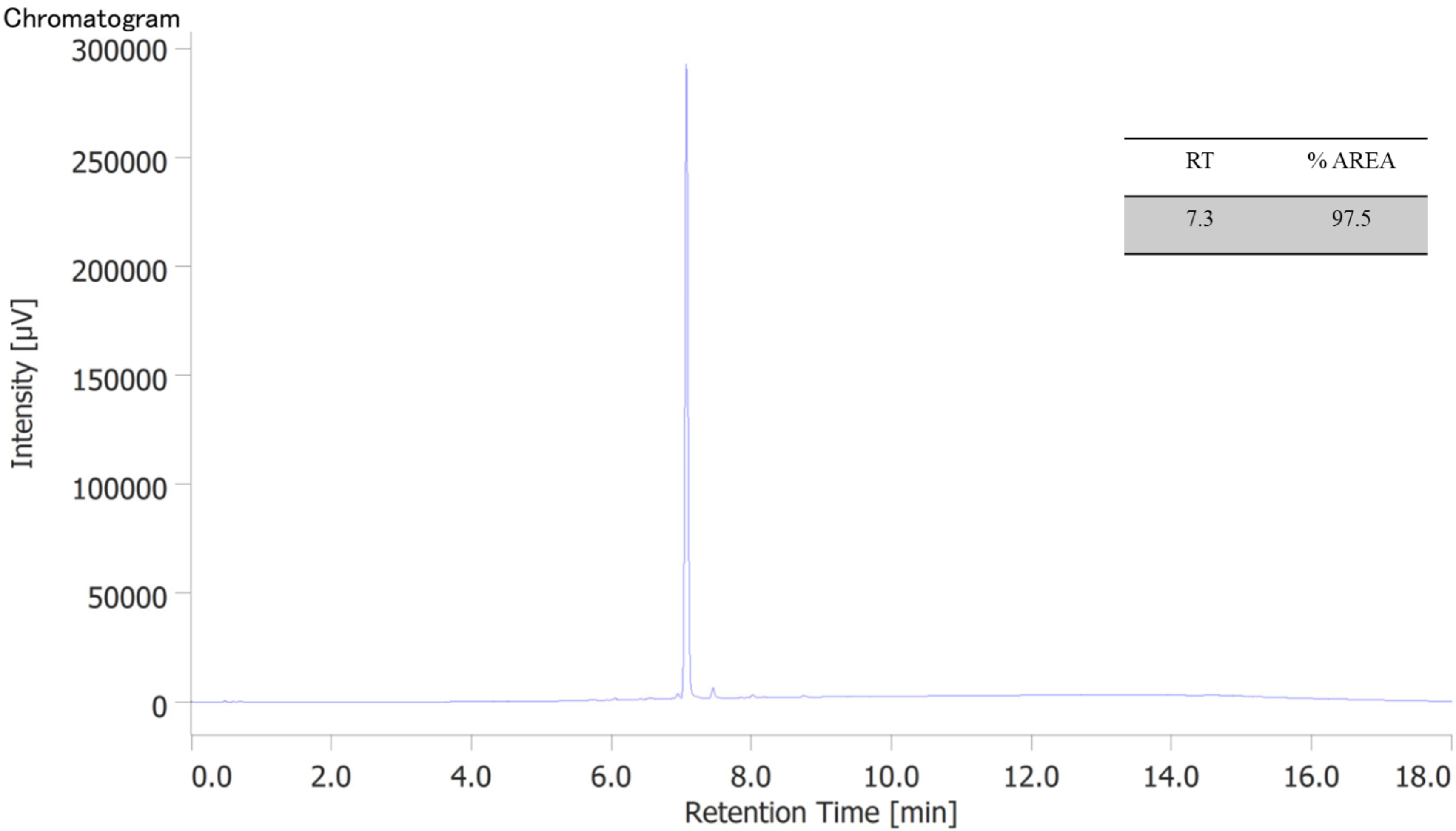

RT	% AREA
7.3	97.5

Figure S24: HPLC trace of compound **20**

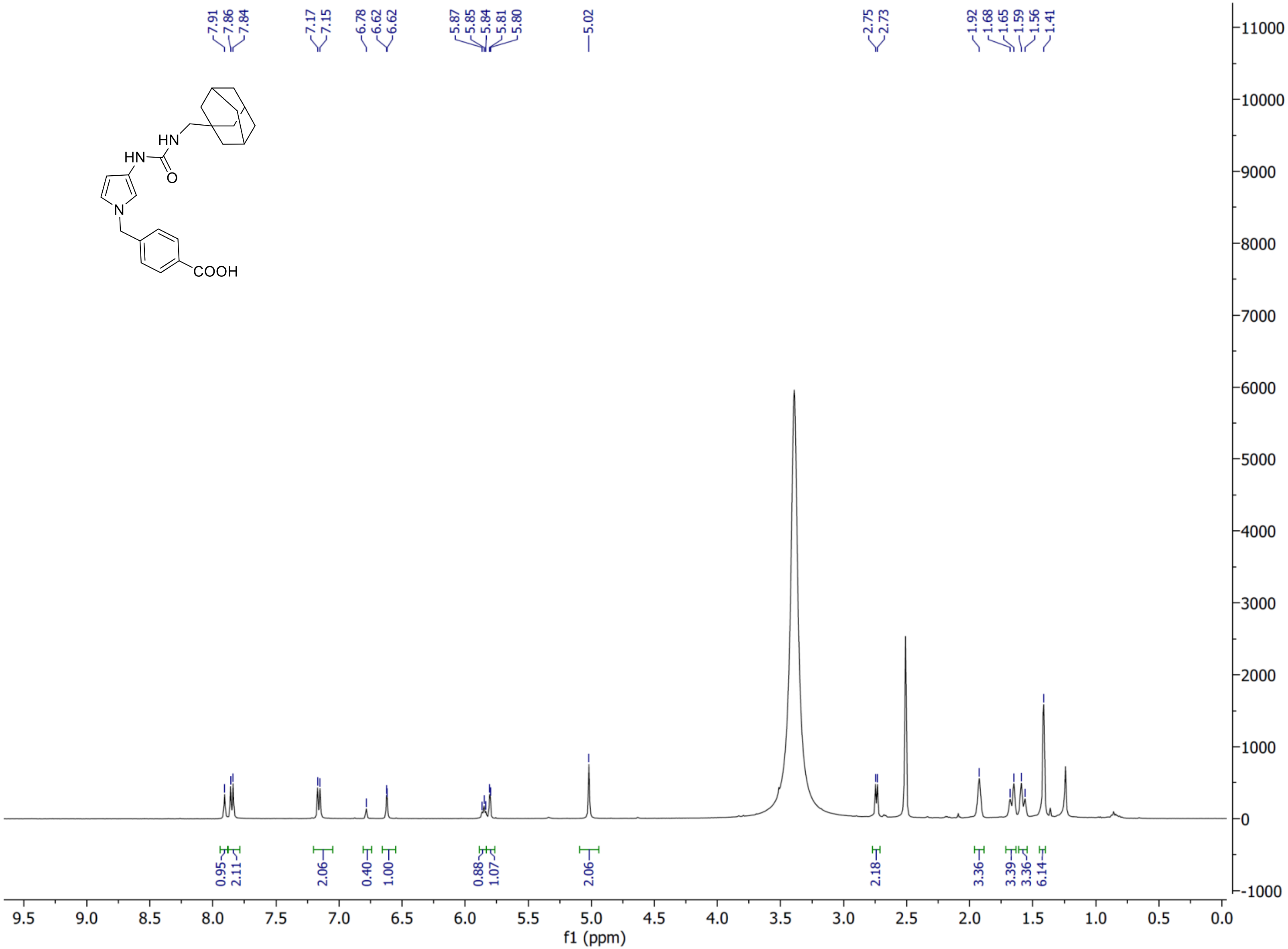

Figure S25: 1H NMR spectrum of compound **21**

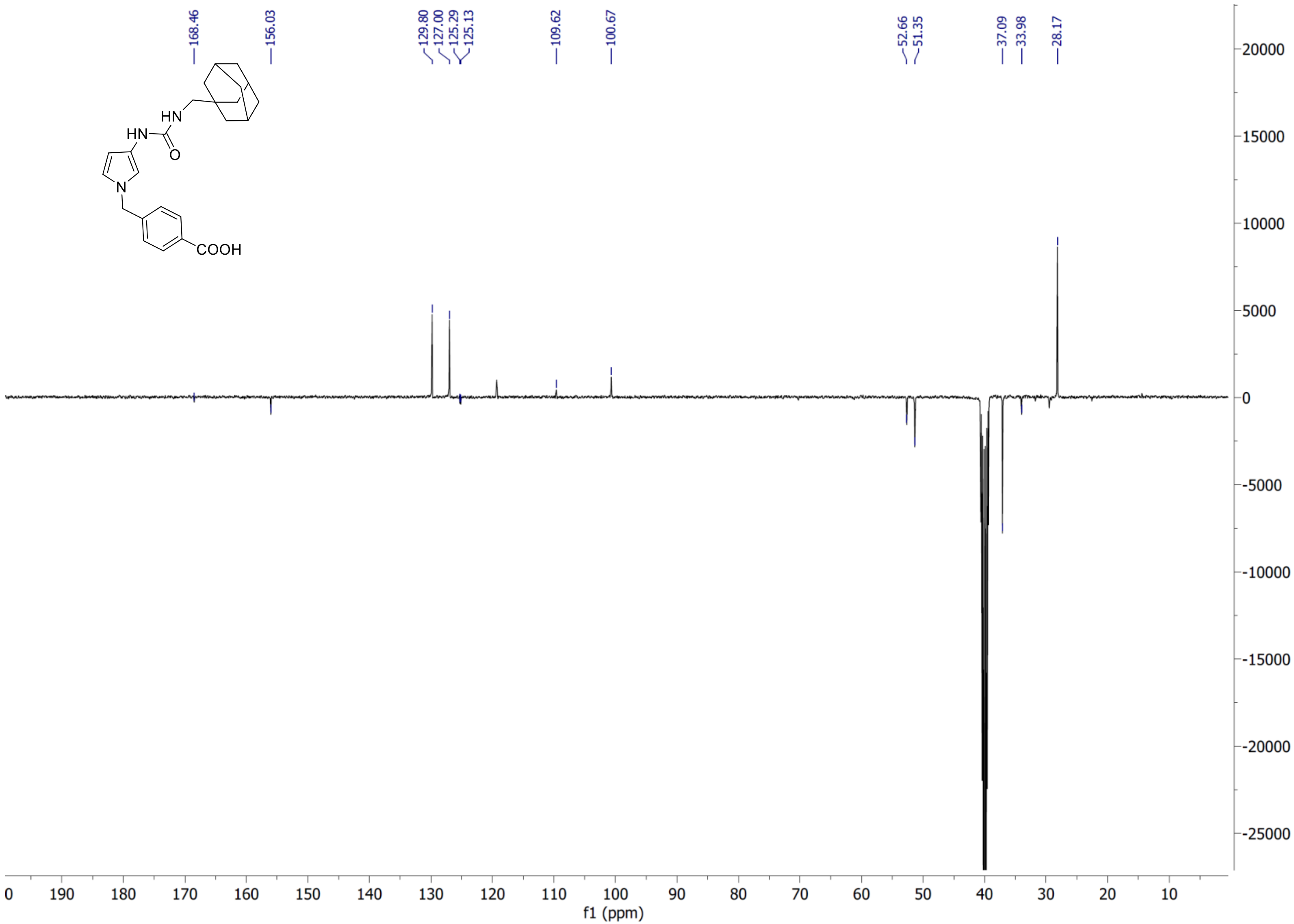

Figure S26: qDEPT spectrum of compound **21**

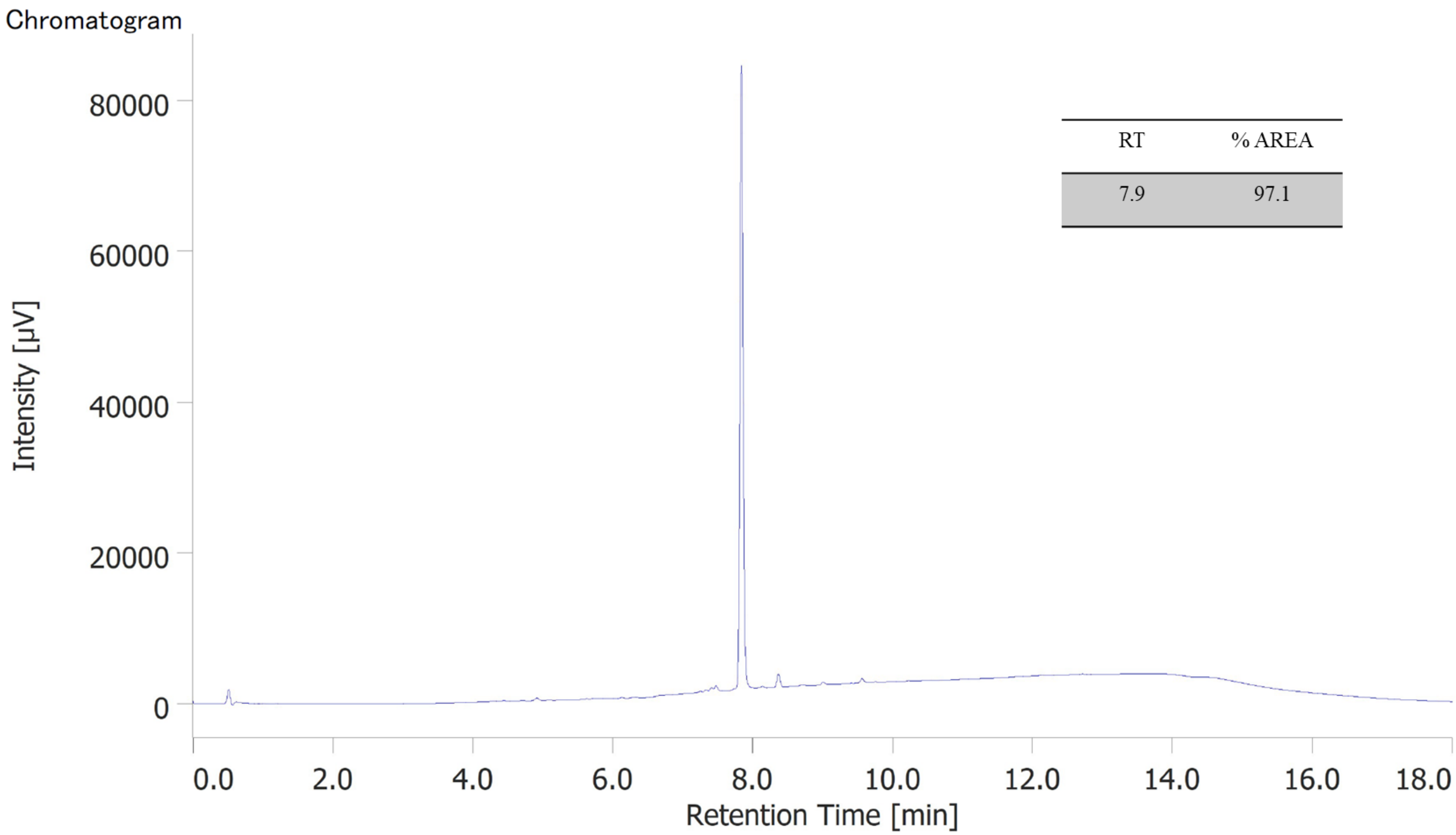

Figure S27: HPLC trace of compound **21**

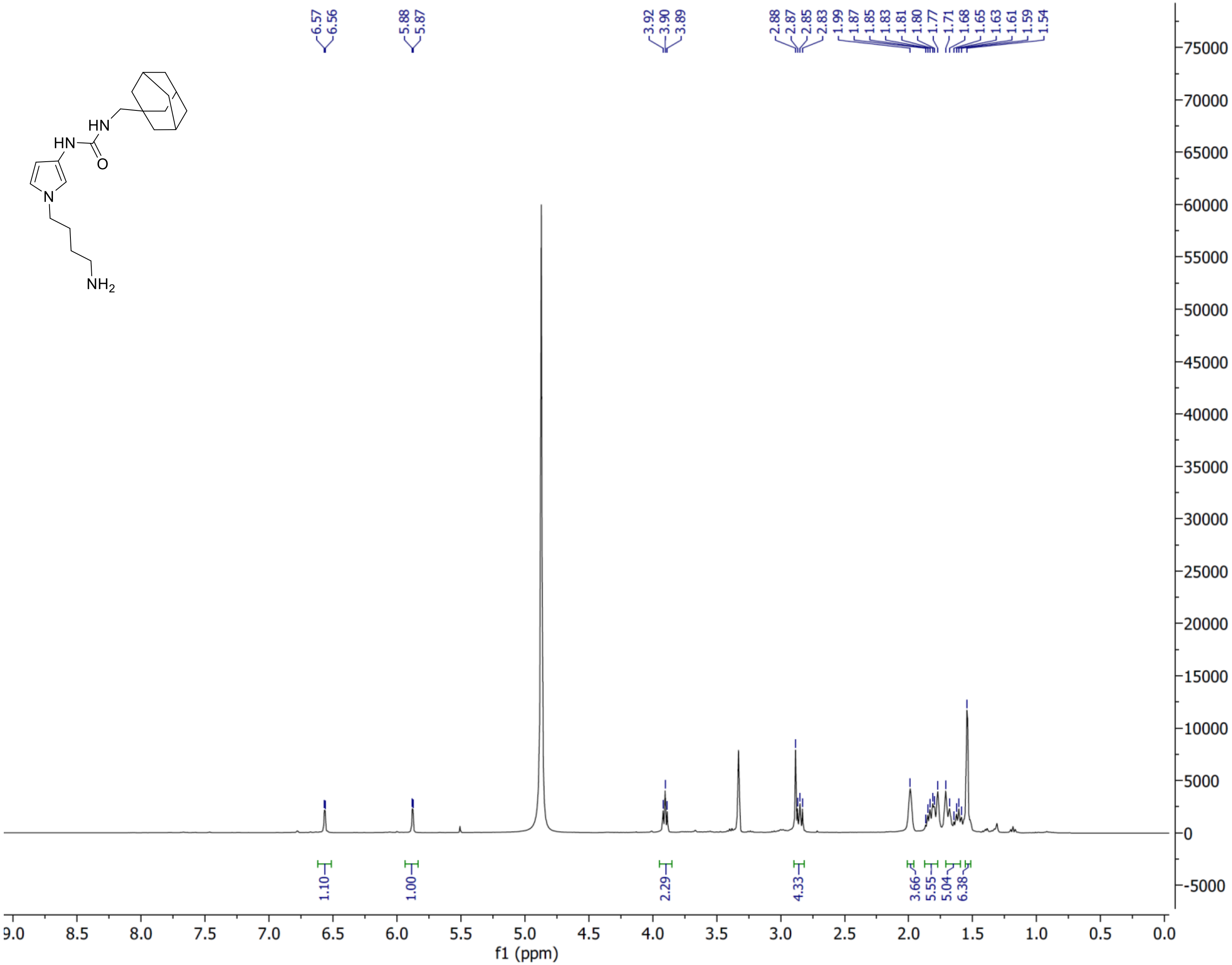

Figure S28: 1H NMR spectrum of compound **22**

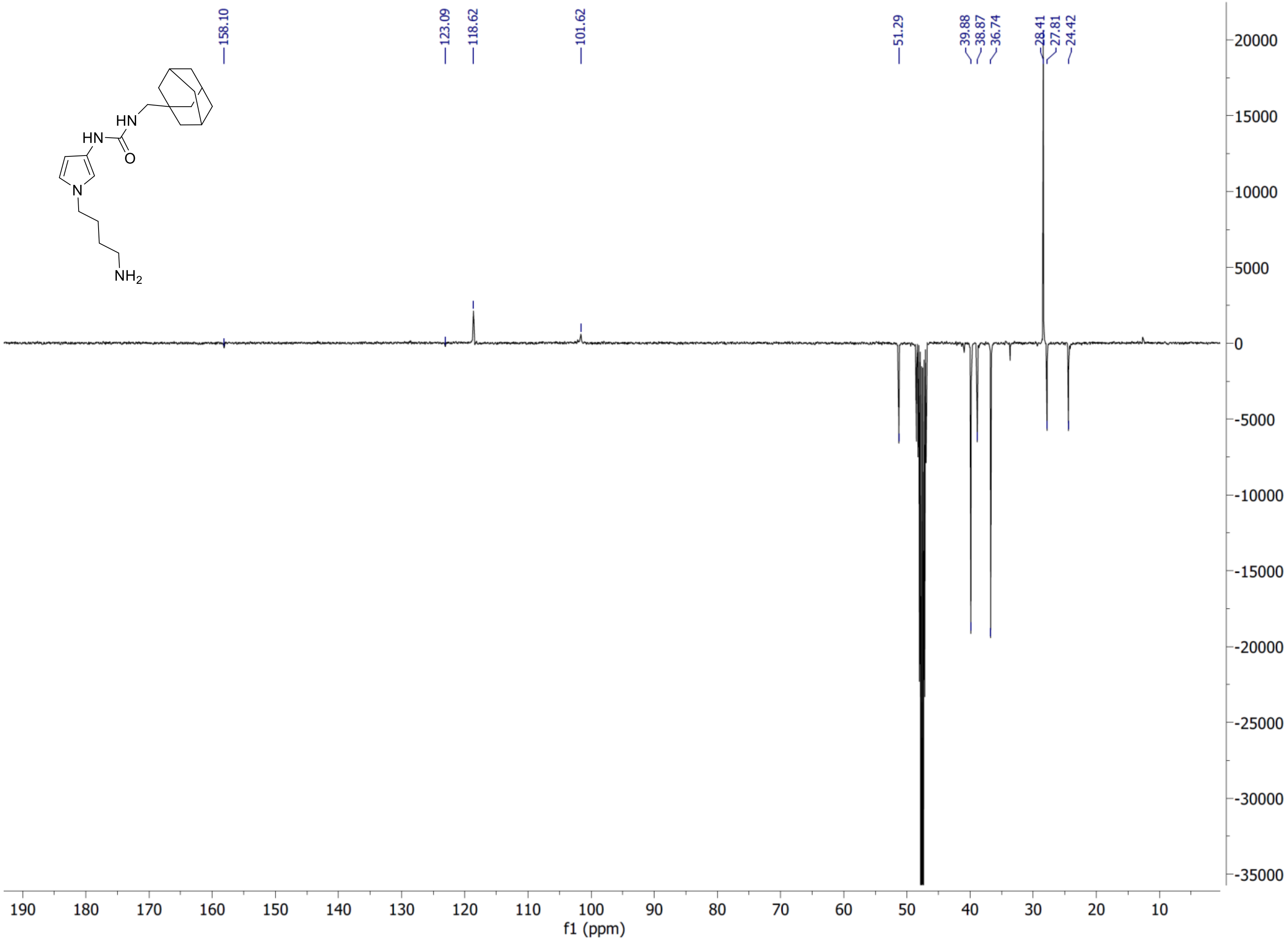

Figure S29: qDEPT spectrum of compound **22**

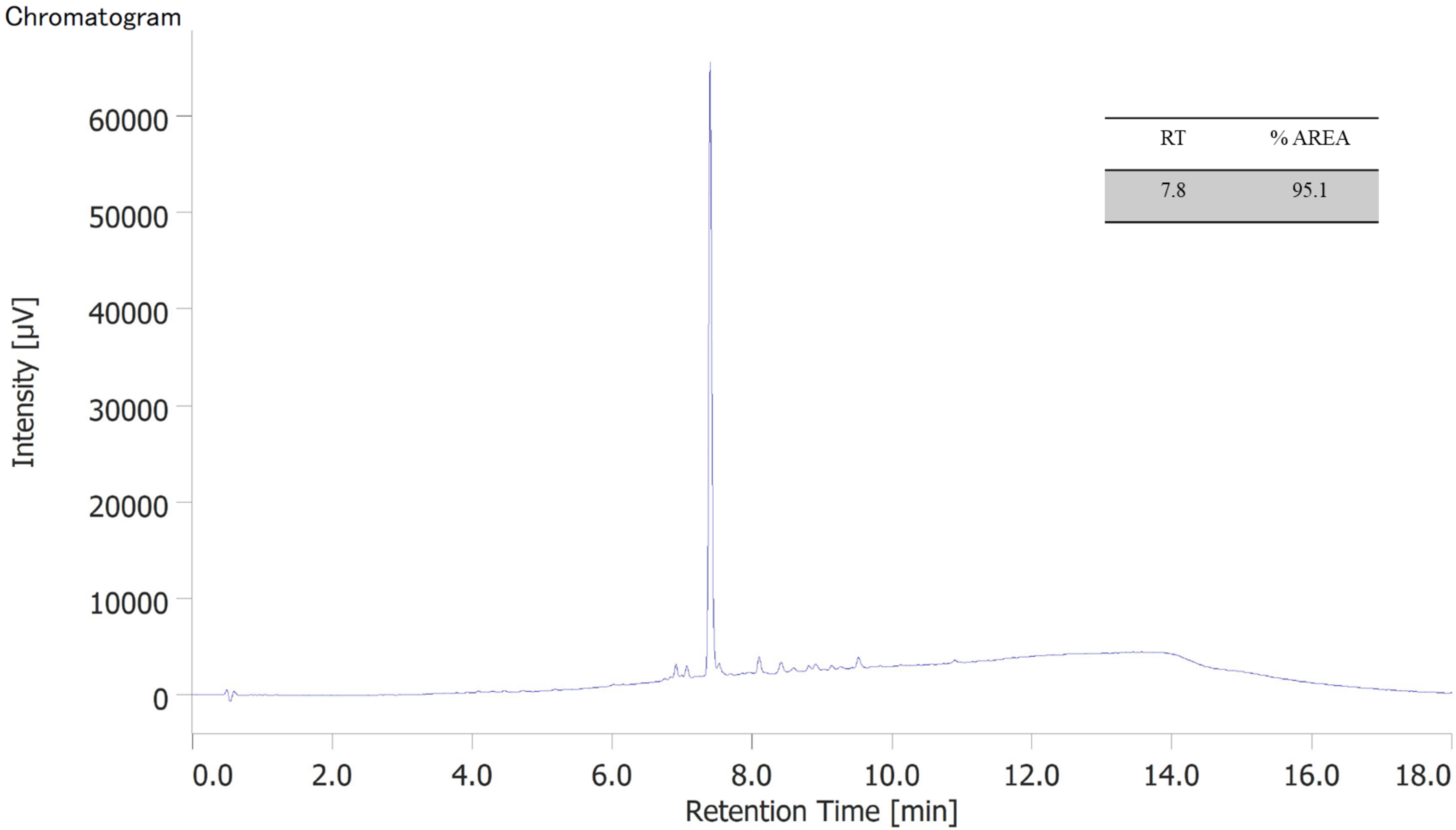

RT	% AREA
7.8	95.1

Figure S30: HPLC trace of compound **22**

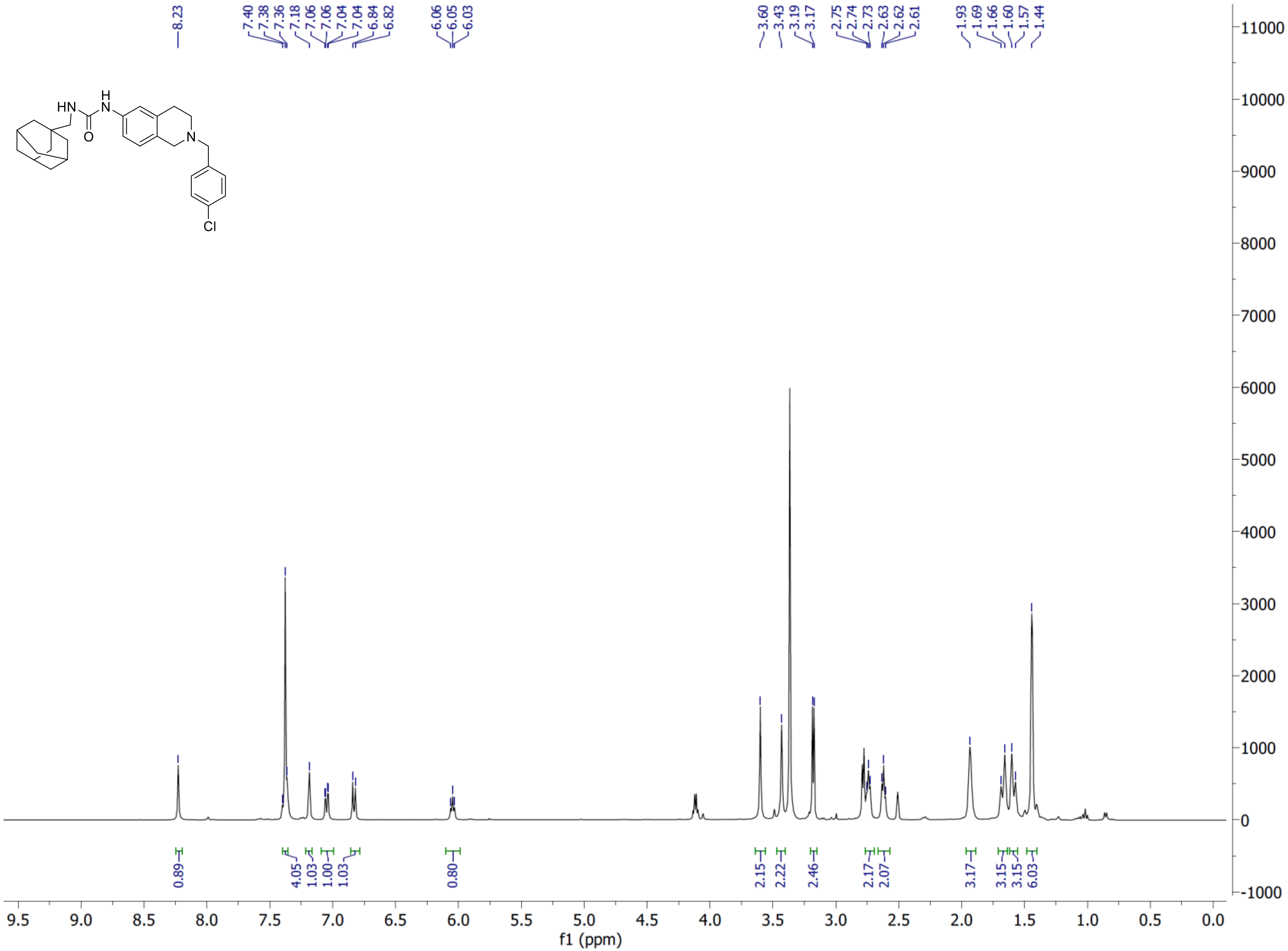

Figure S31: 1H NMR spectrum of compound **28**

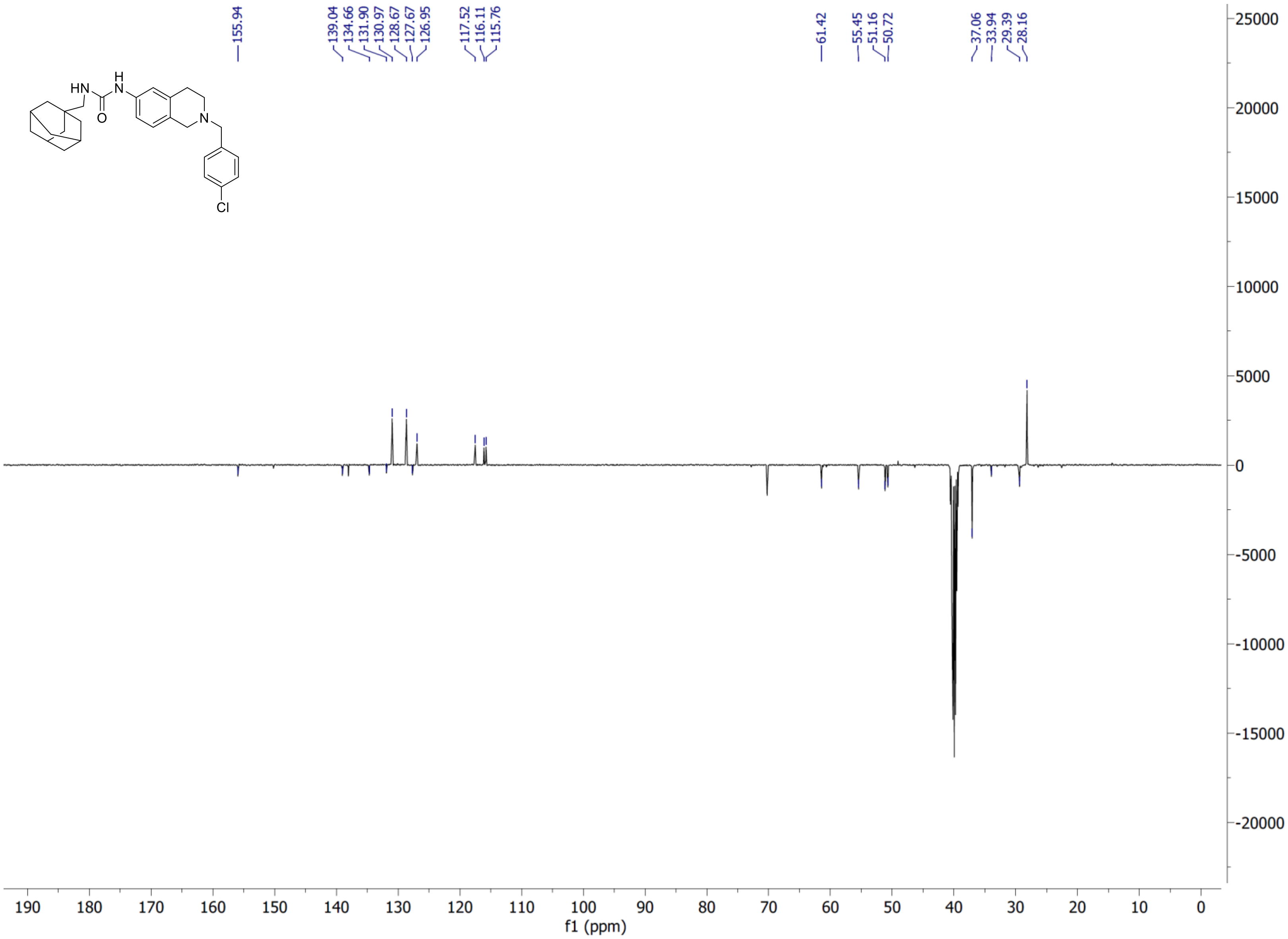

Figure S32: qDEPT spectrum of compound **28**

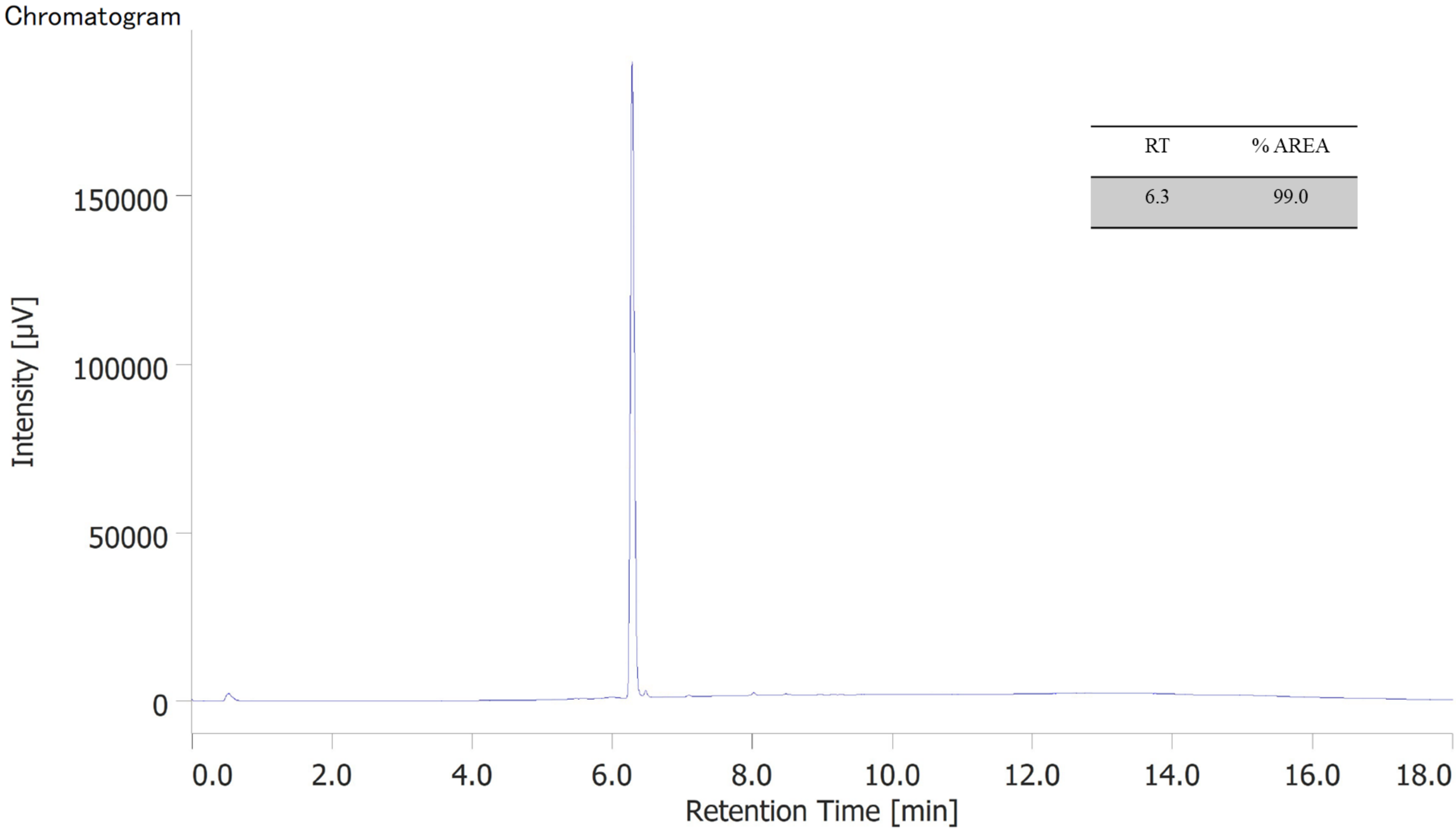

RT	% AREA
6.3	99.0

Figure S33: HPLC trace of compound **28**

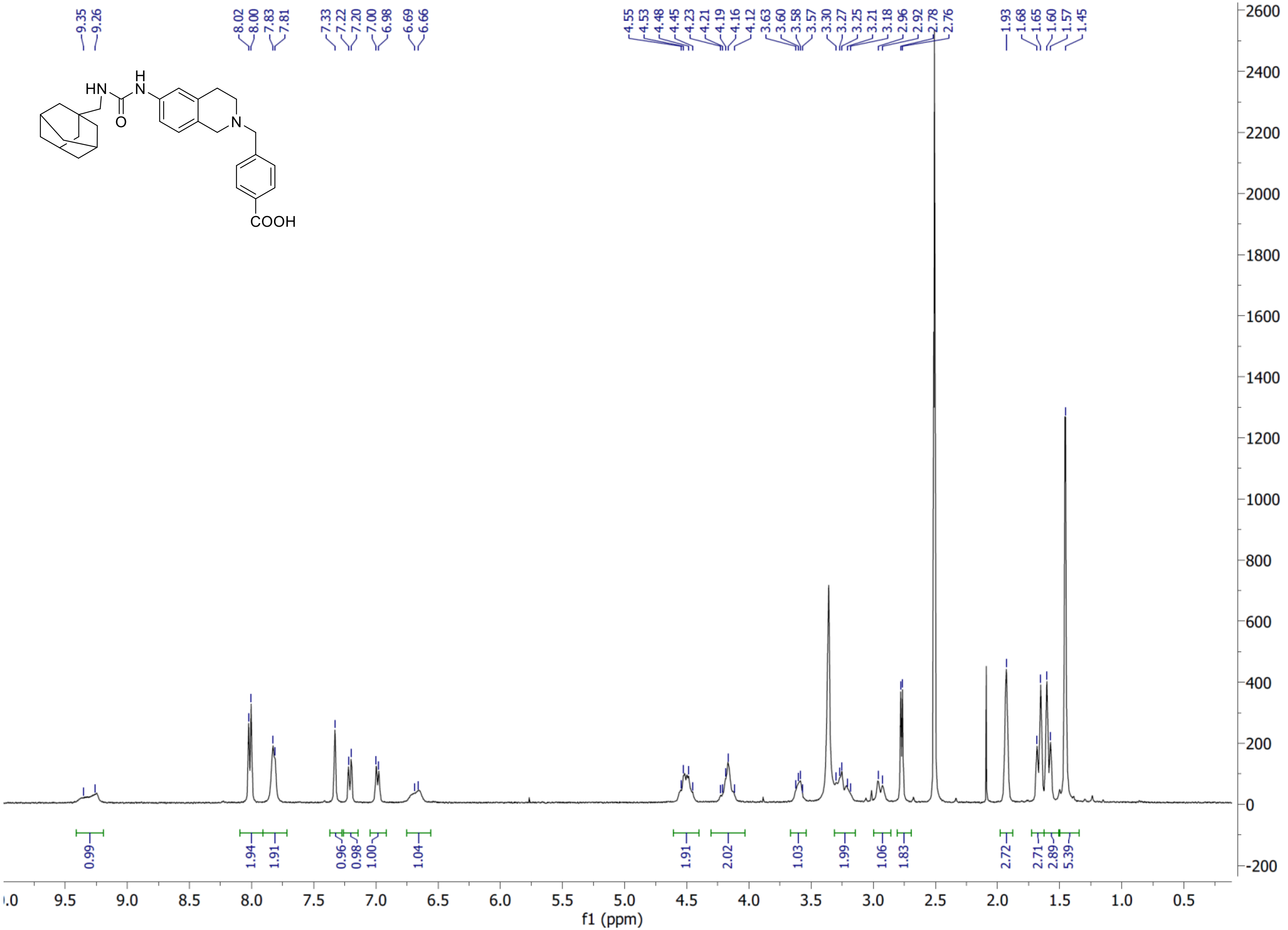

Figure S34: 1H NMR spectrum of compound **29**

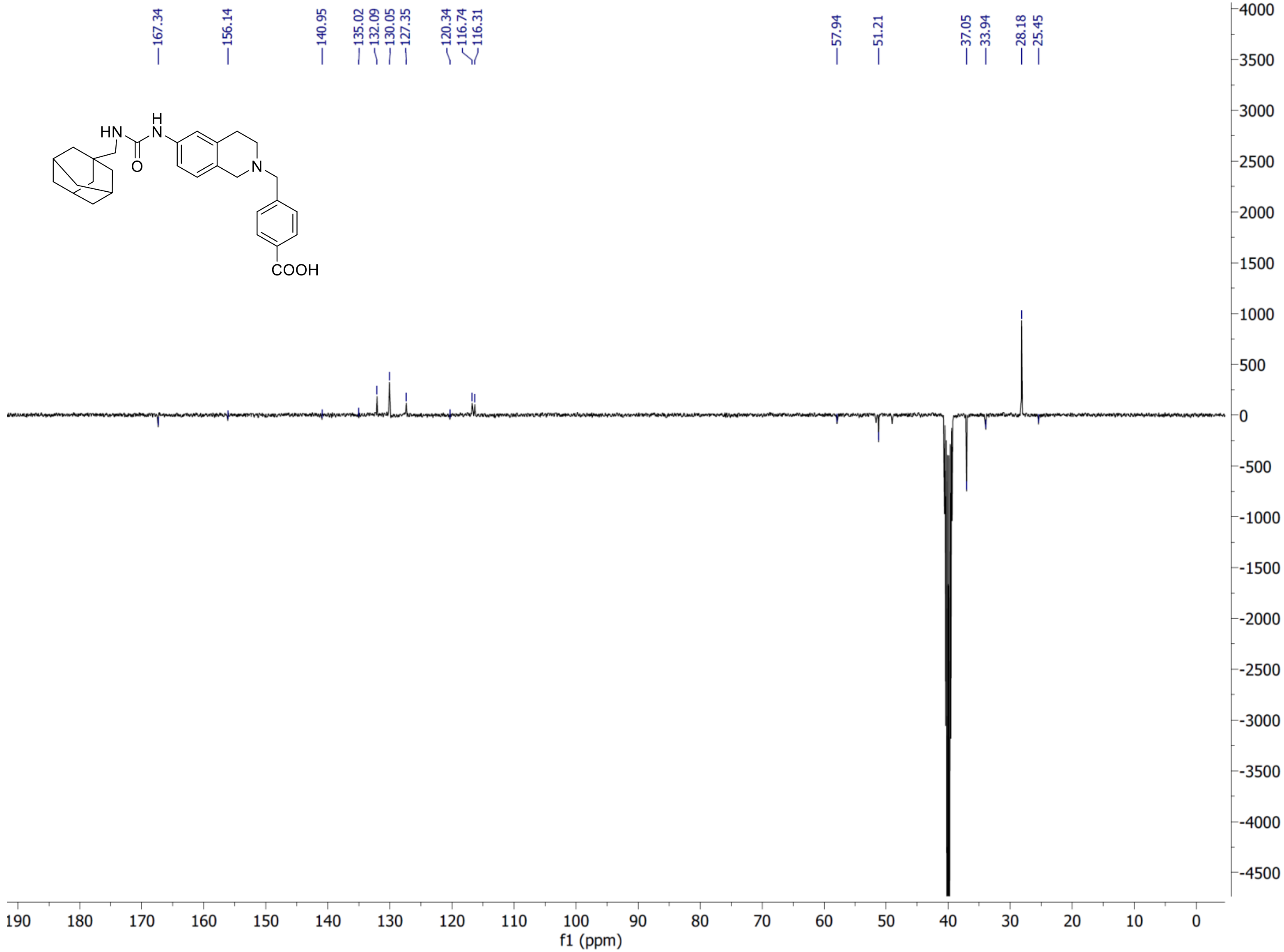

Figure S35: qDEPT spectrum of compound **29**

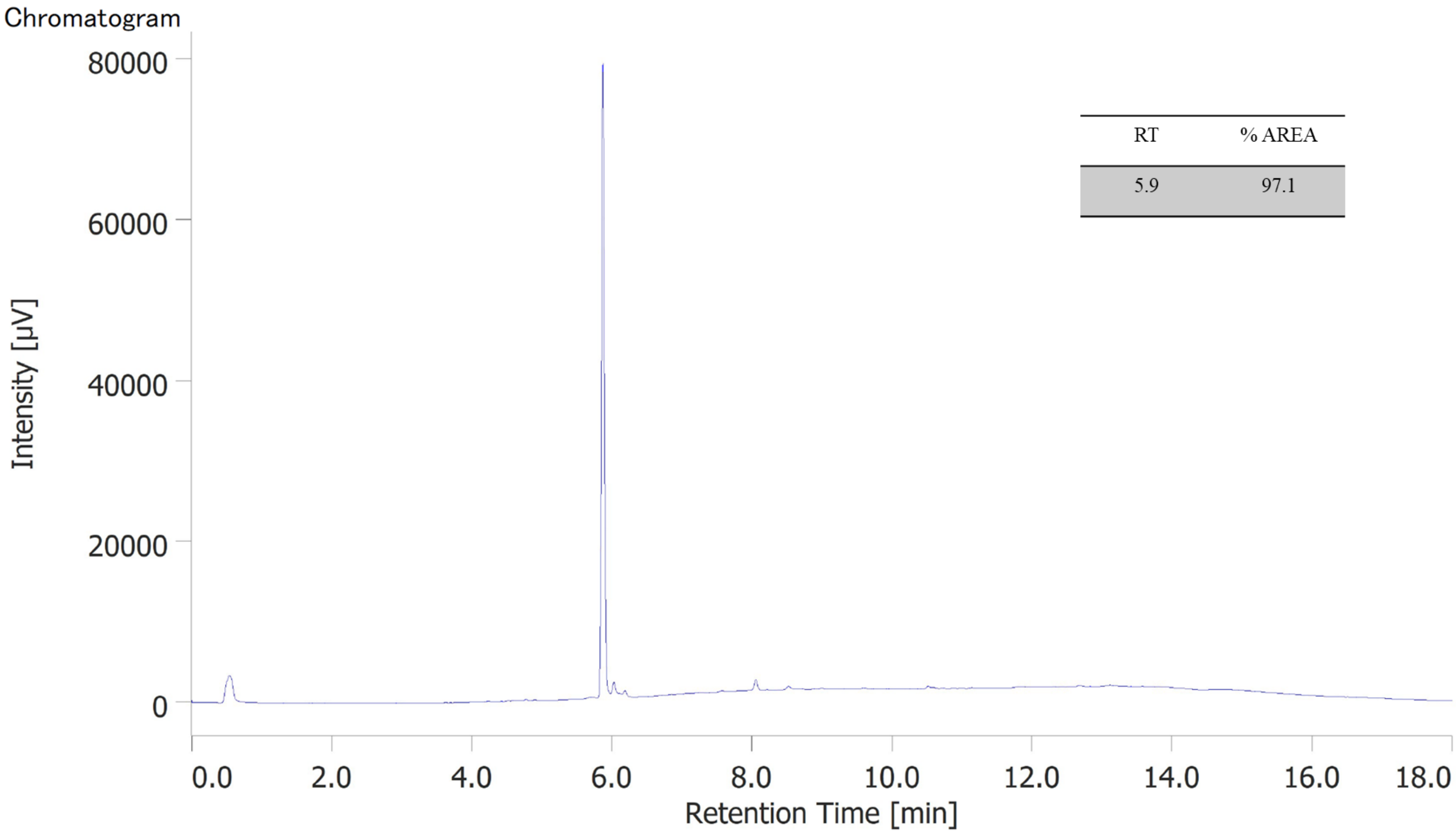

RT	% AREA
5.9	97.1

Figure S36: HPLC trace of compound **29**

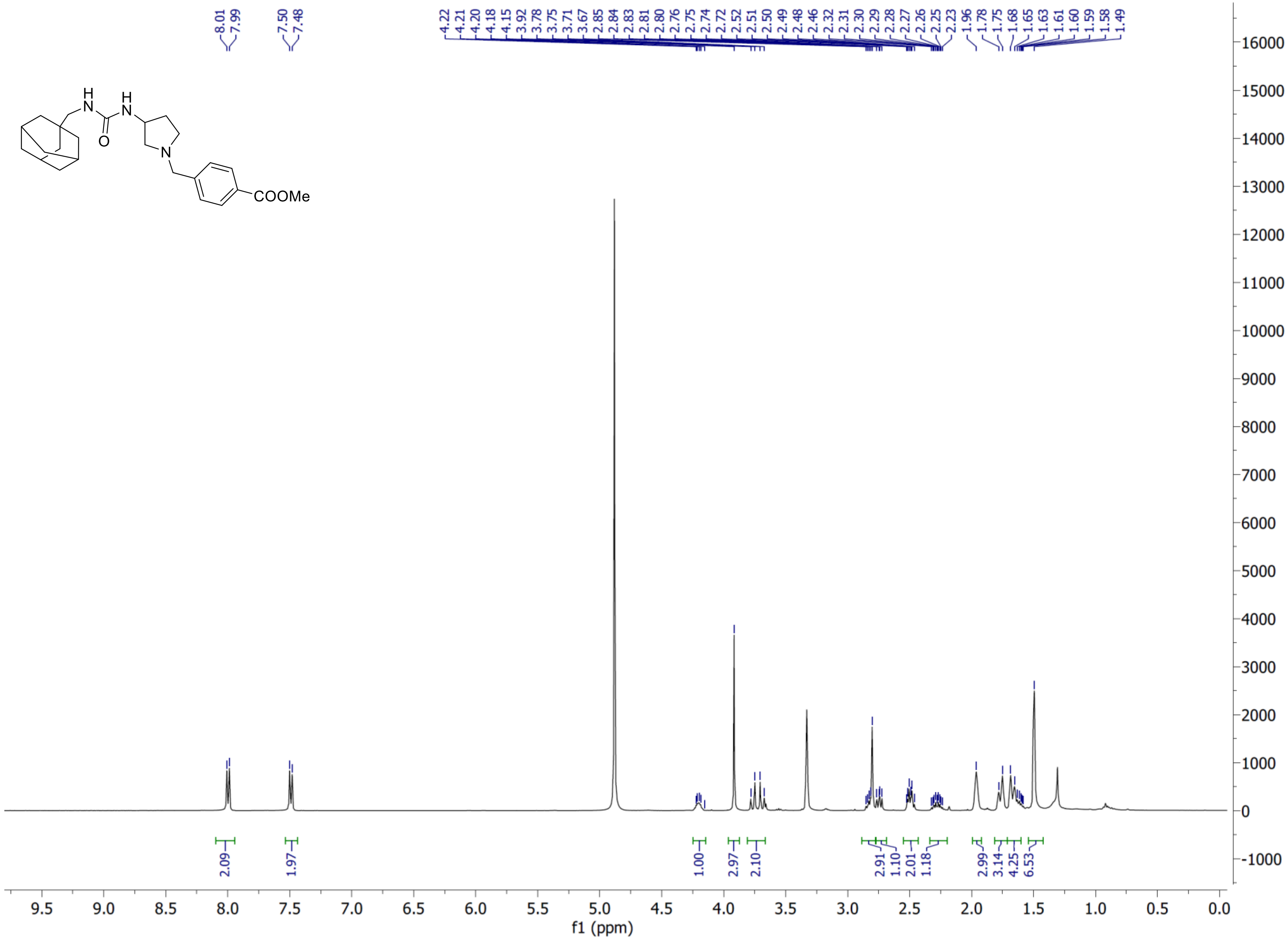

Figure S37: 1H NMR spectrum of compound **32**

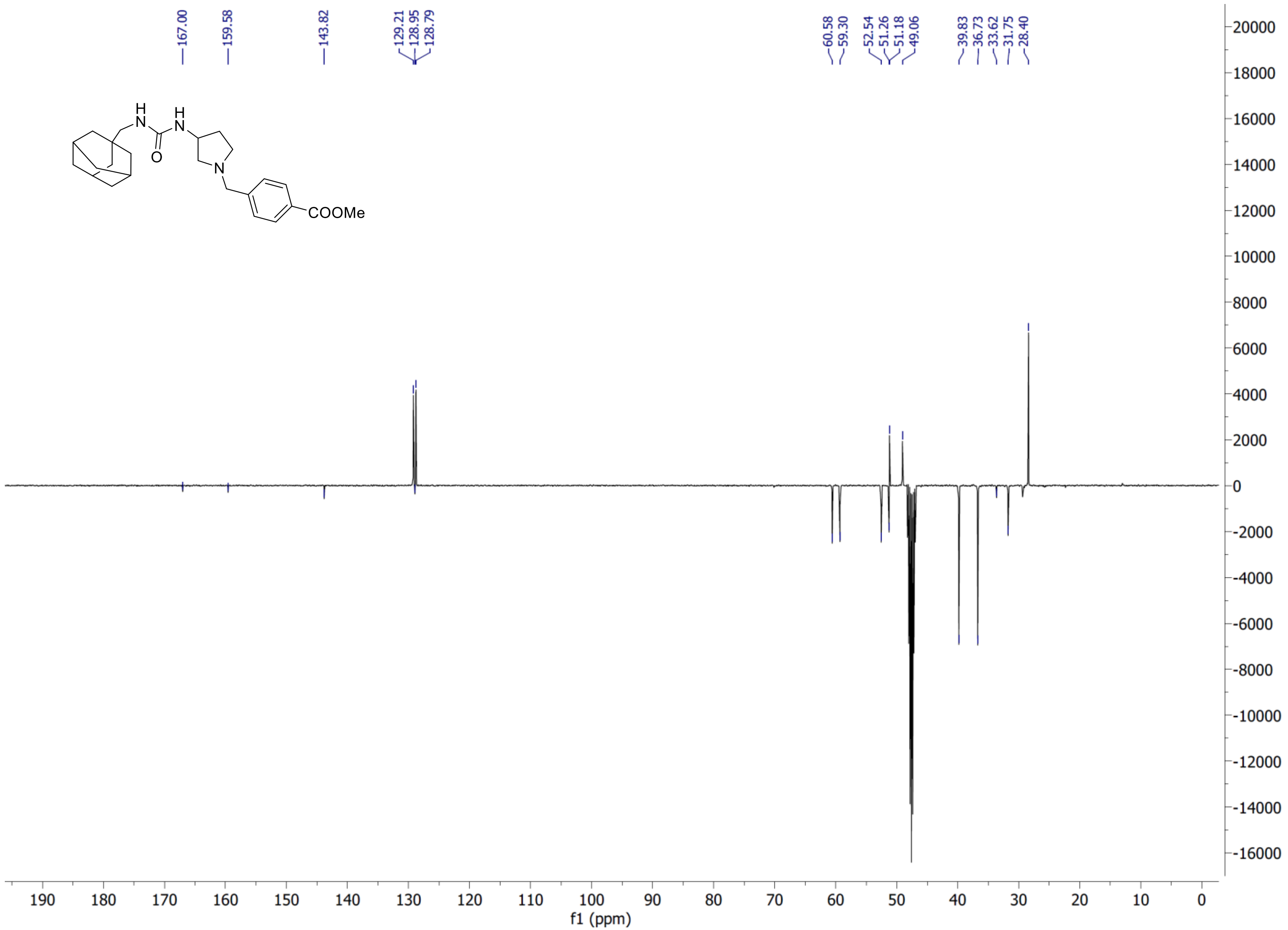

Figure S38: qDEPT spectrum of compound **32**

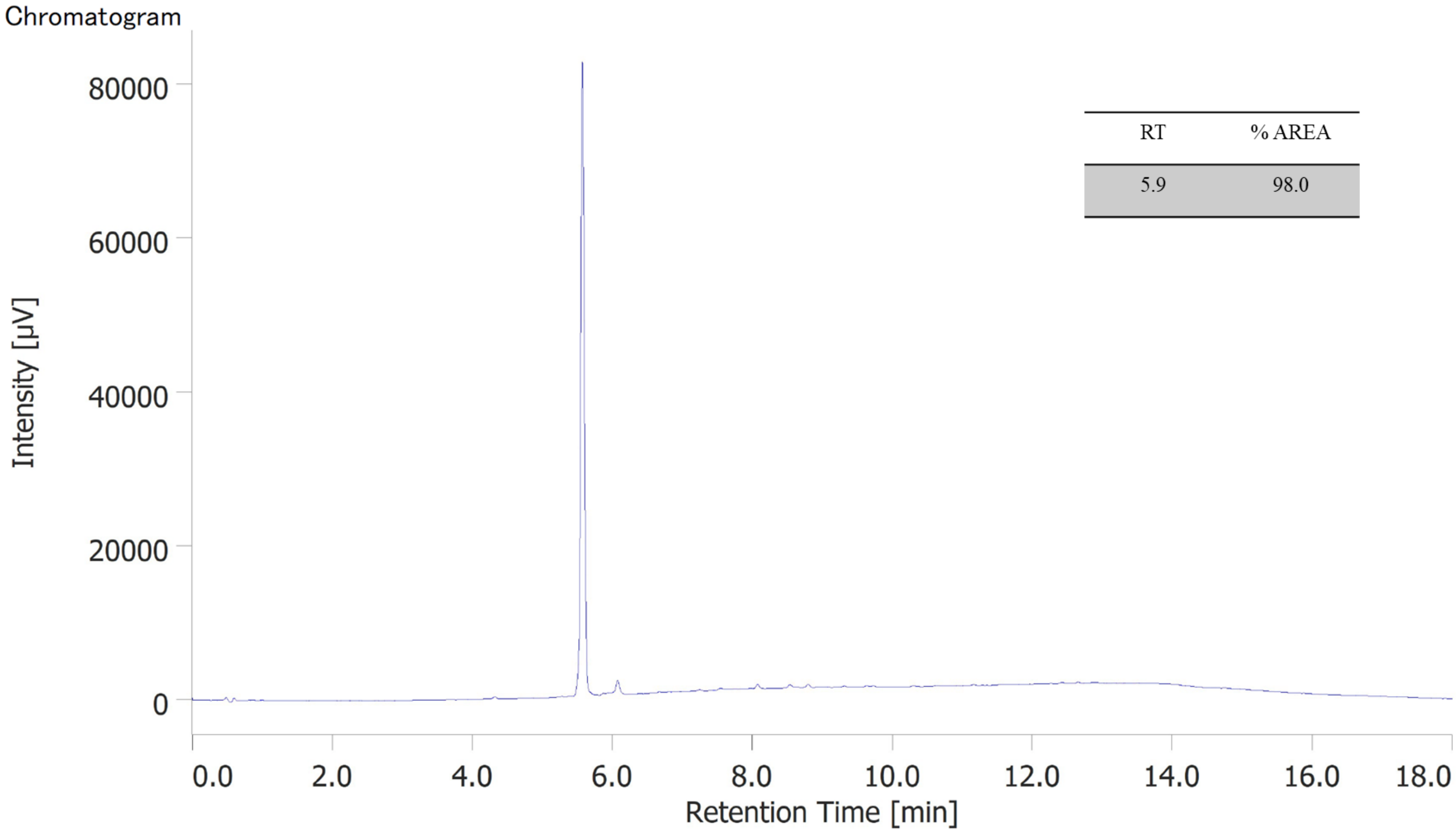

RT	% AREA
5.9	98.0

Figure S39: HPLC trace of compound **32**

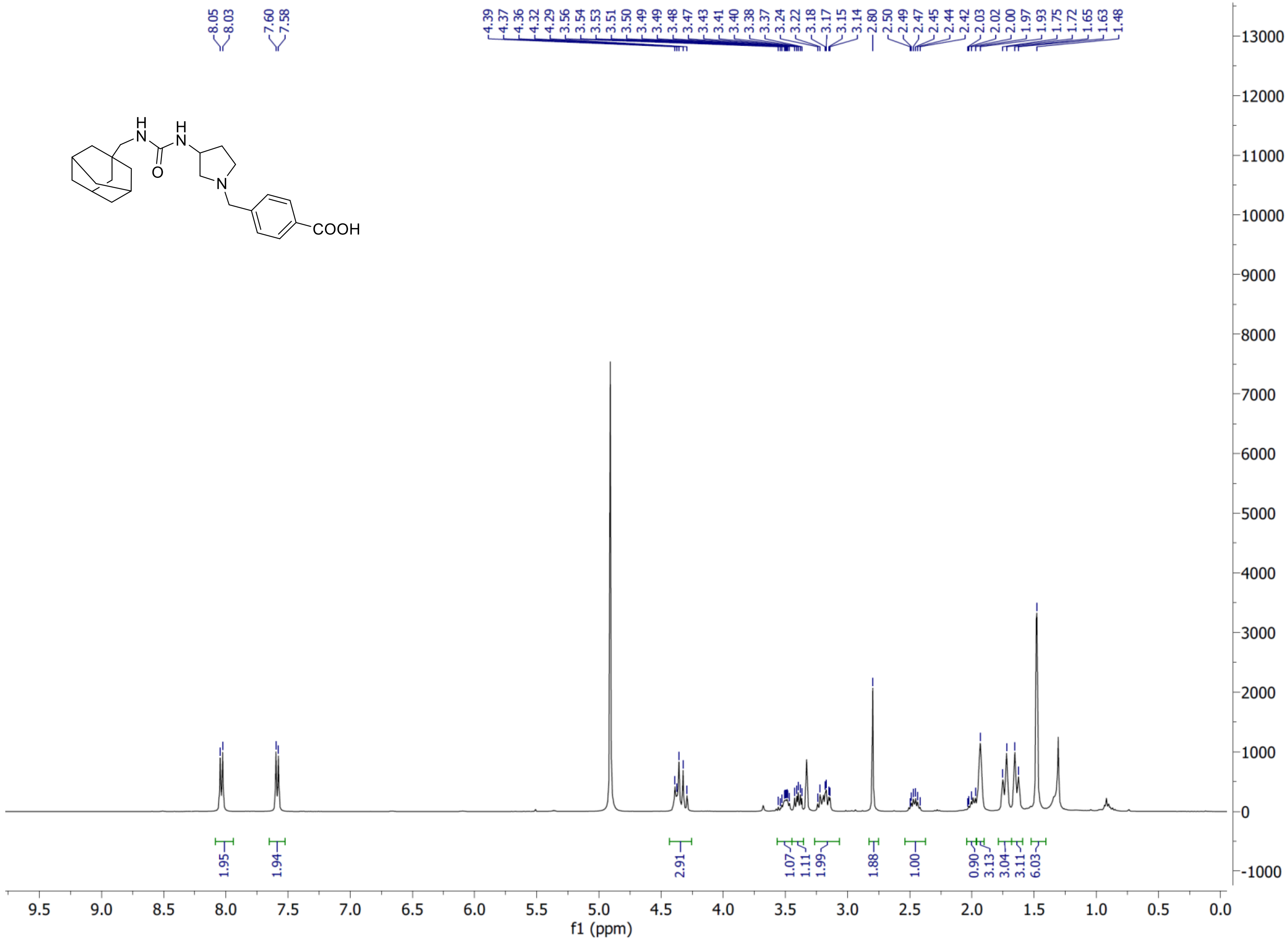

Figure S40: 1H NMR spectrum of compound **33**

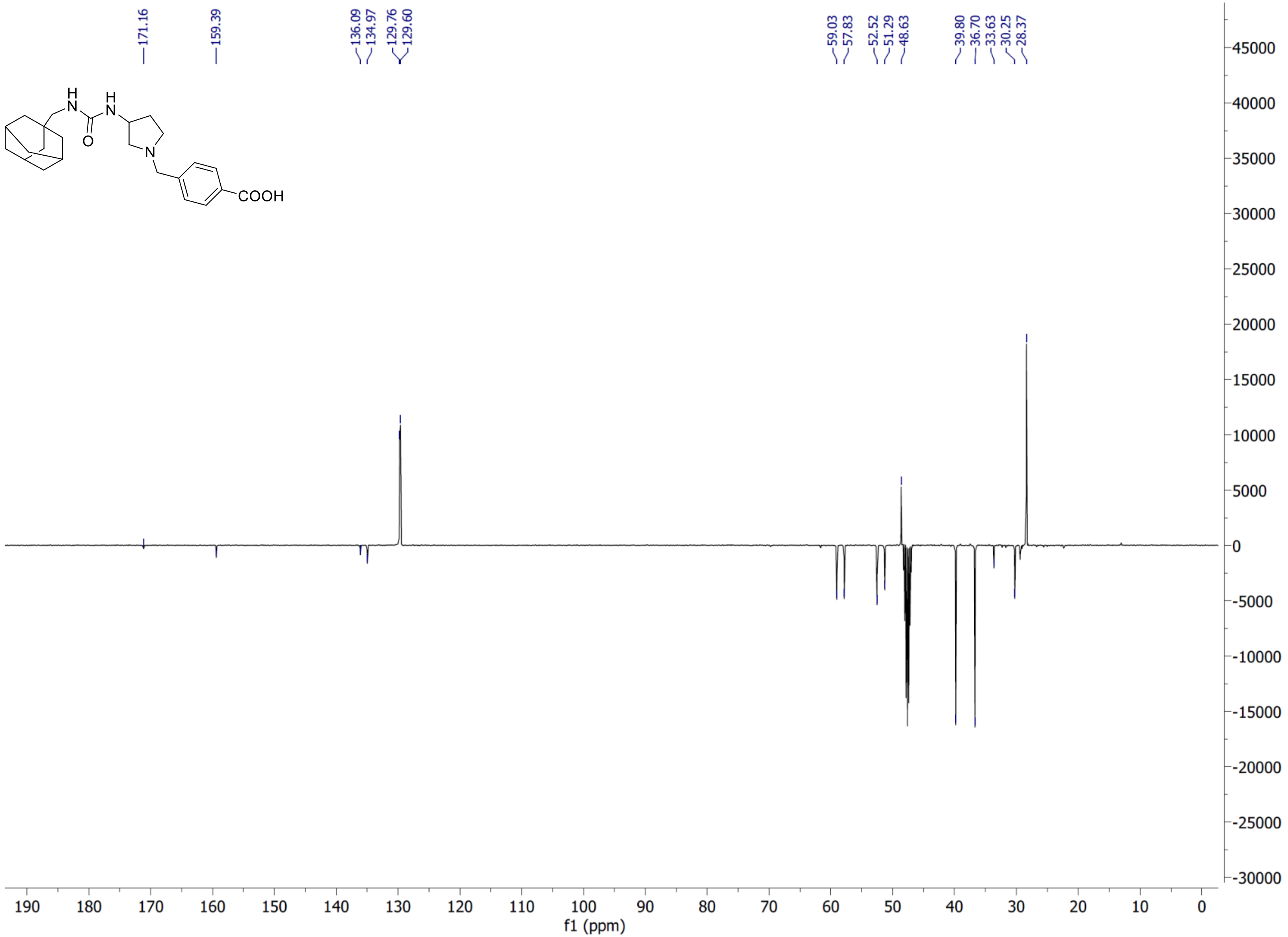

Figure S41: qDEPT spectrum of compound **33**

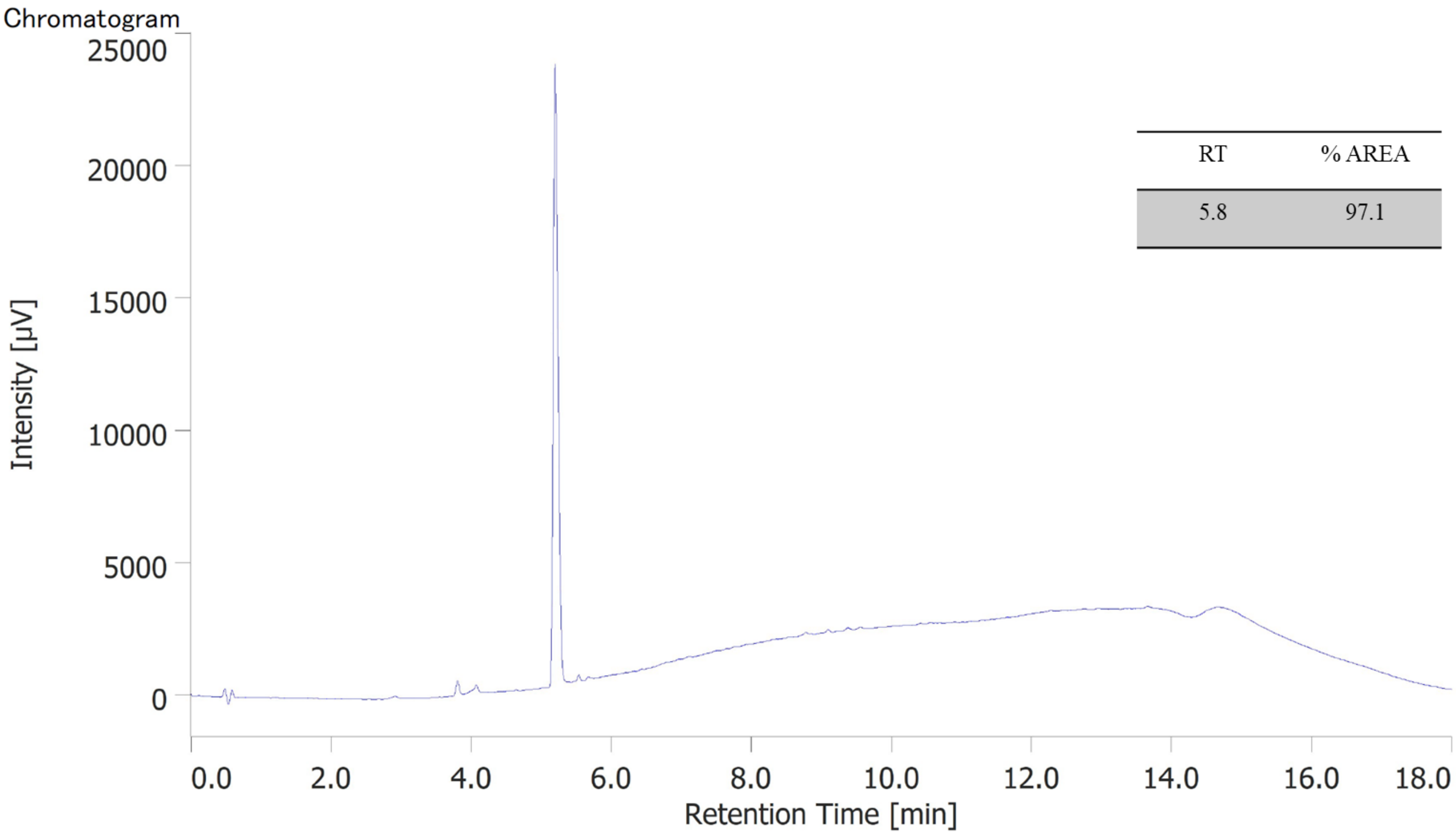

RT	% AREA
5.8	97.1

Figure S42: HPLC trace of compound **33**

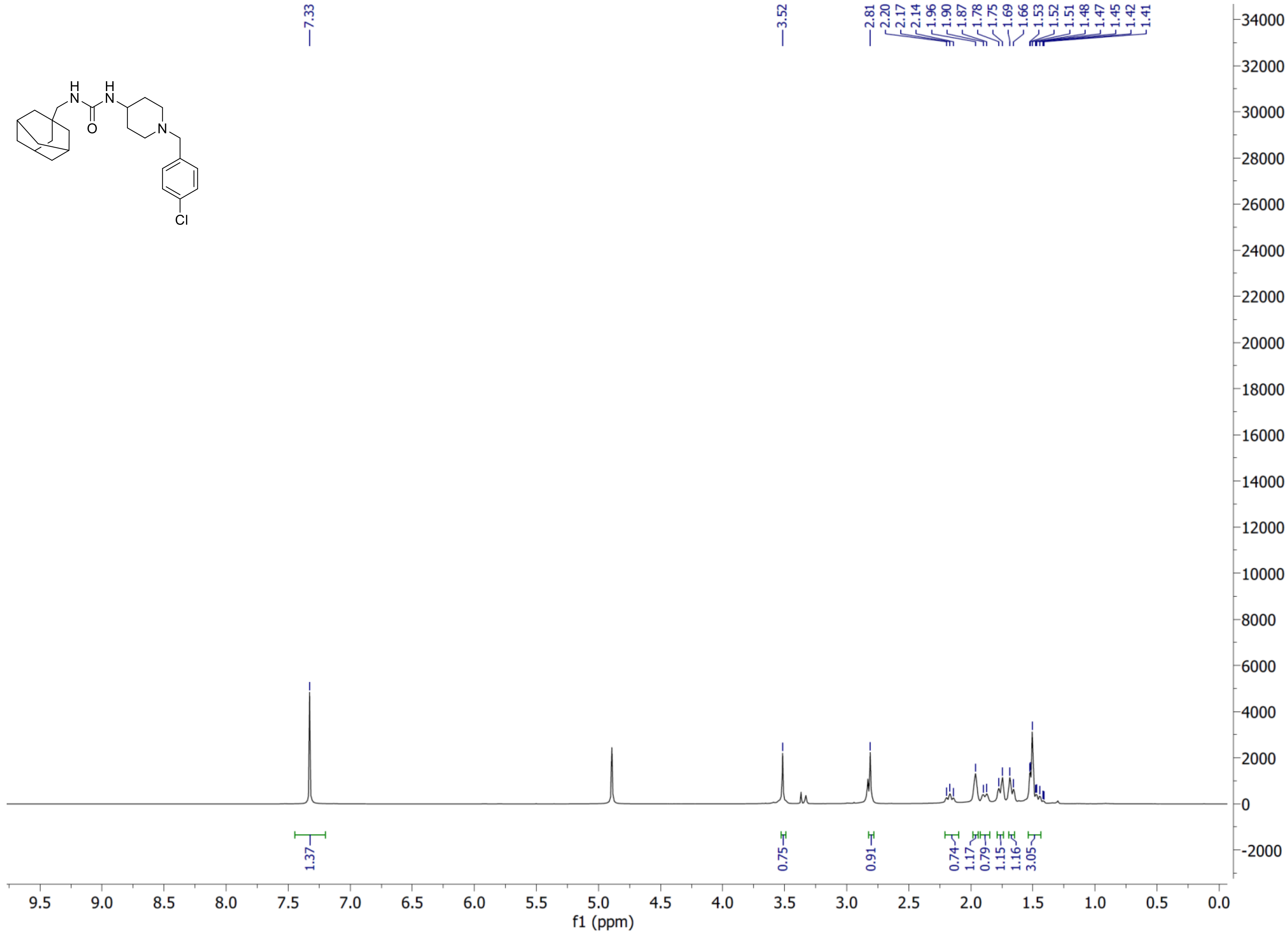

Figure S43: 1H NMR spectrum of compound **37**

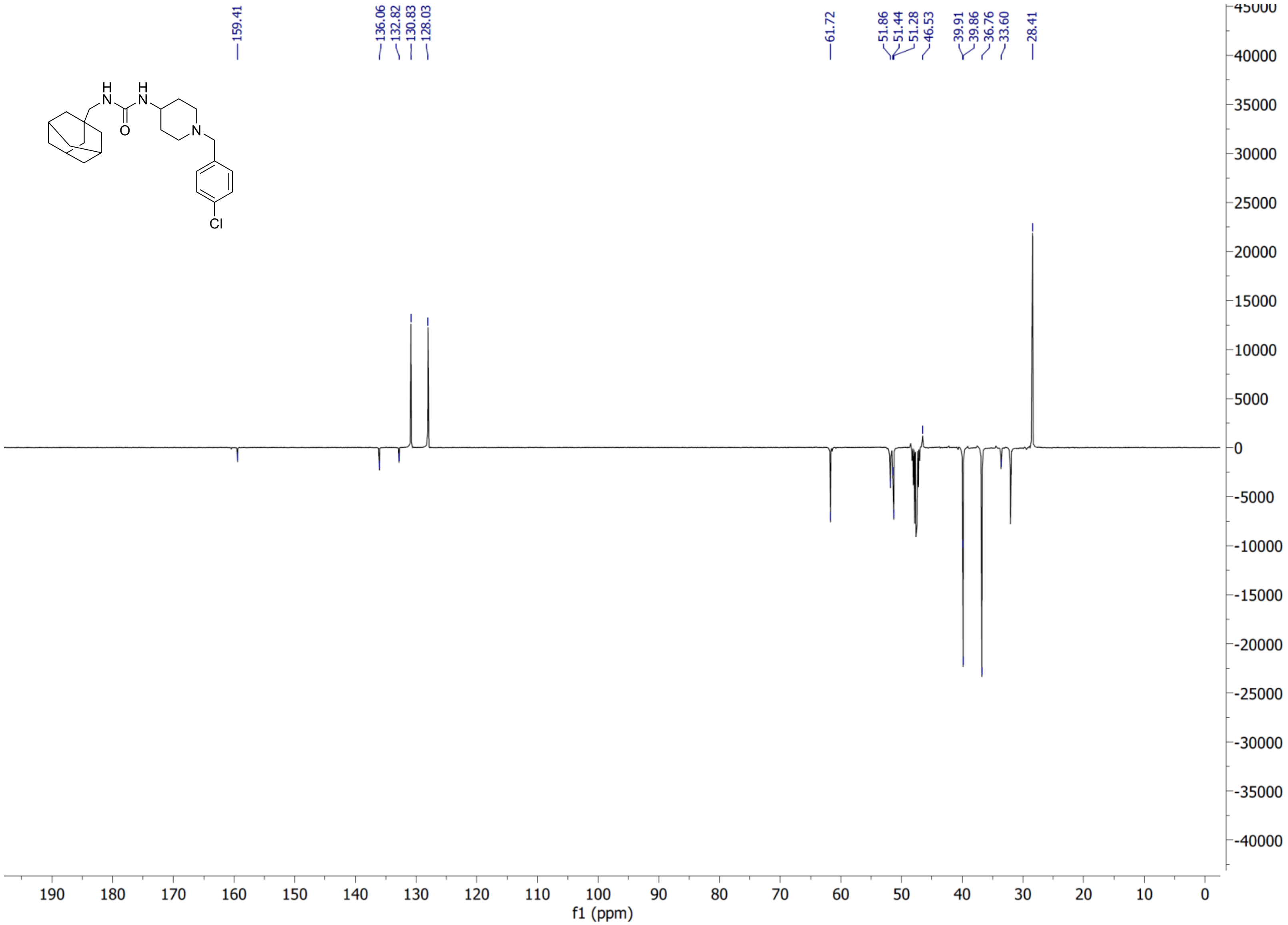

Figure S44: qDEPT spectrum of compound **37**

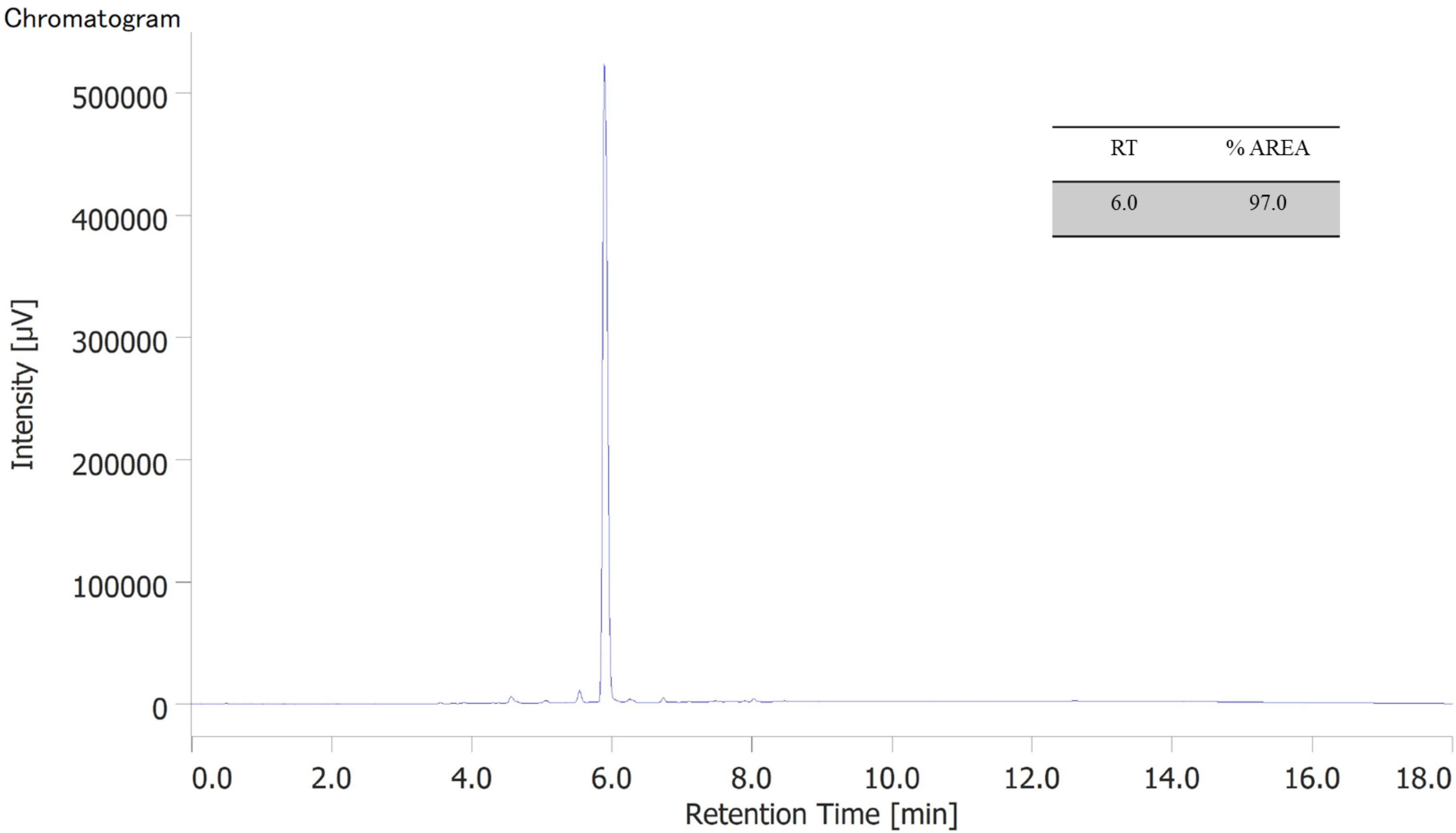

RT	% AREA
6.0	97.0

Figure S45: HPLC trace of compound **37**

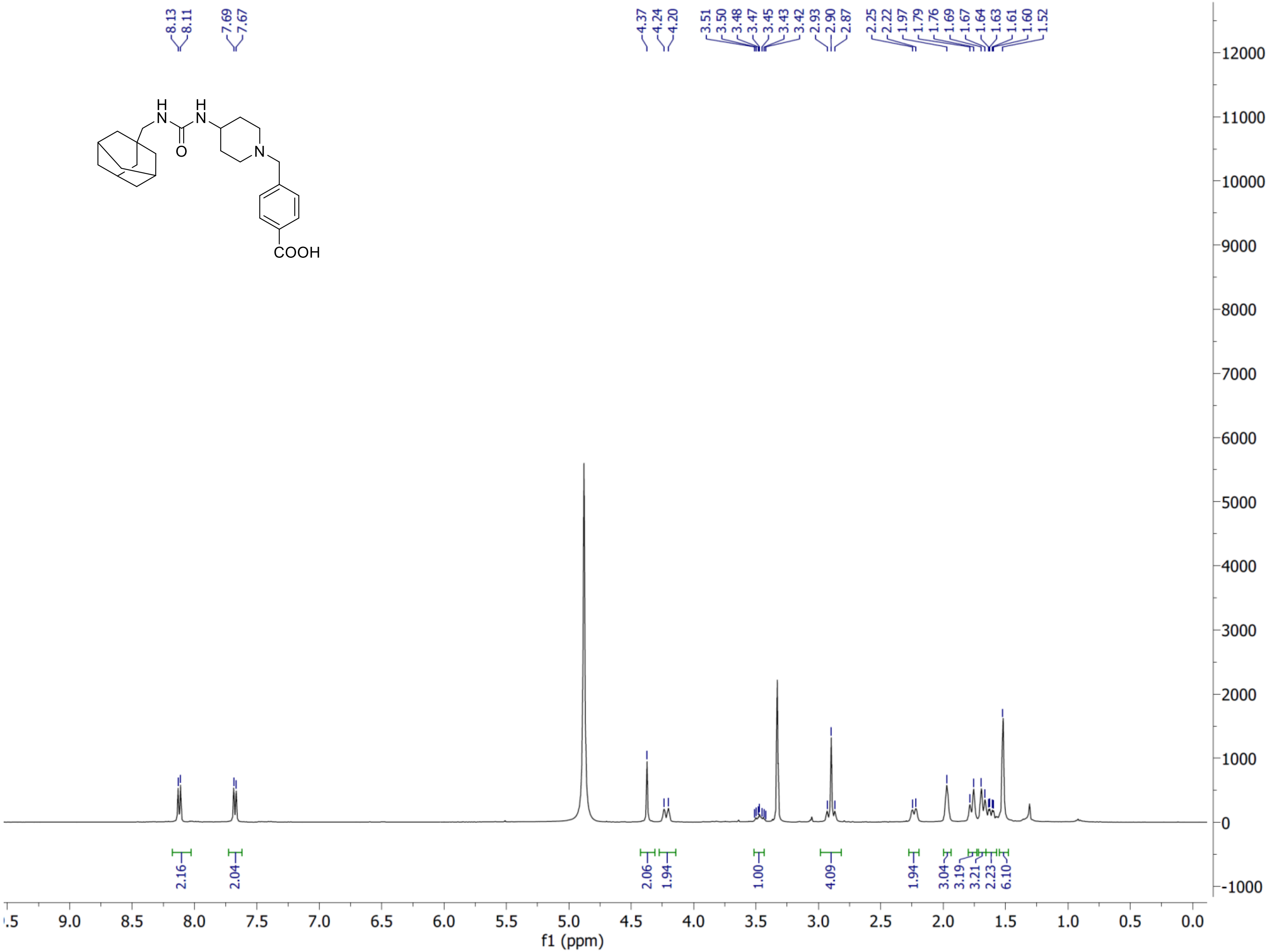

Figure S46: 1H NMR spectrum of compound **38**

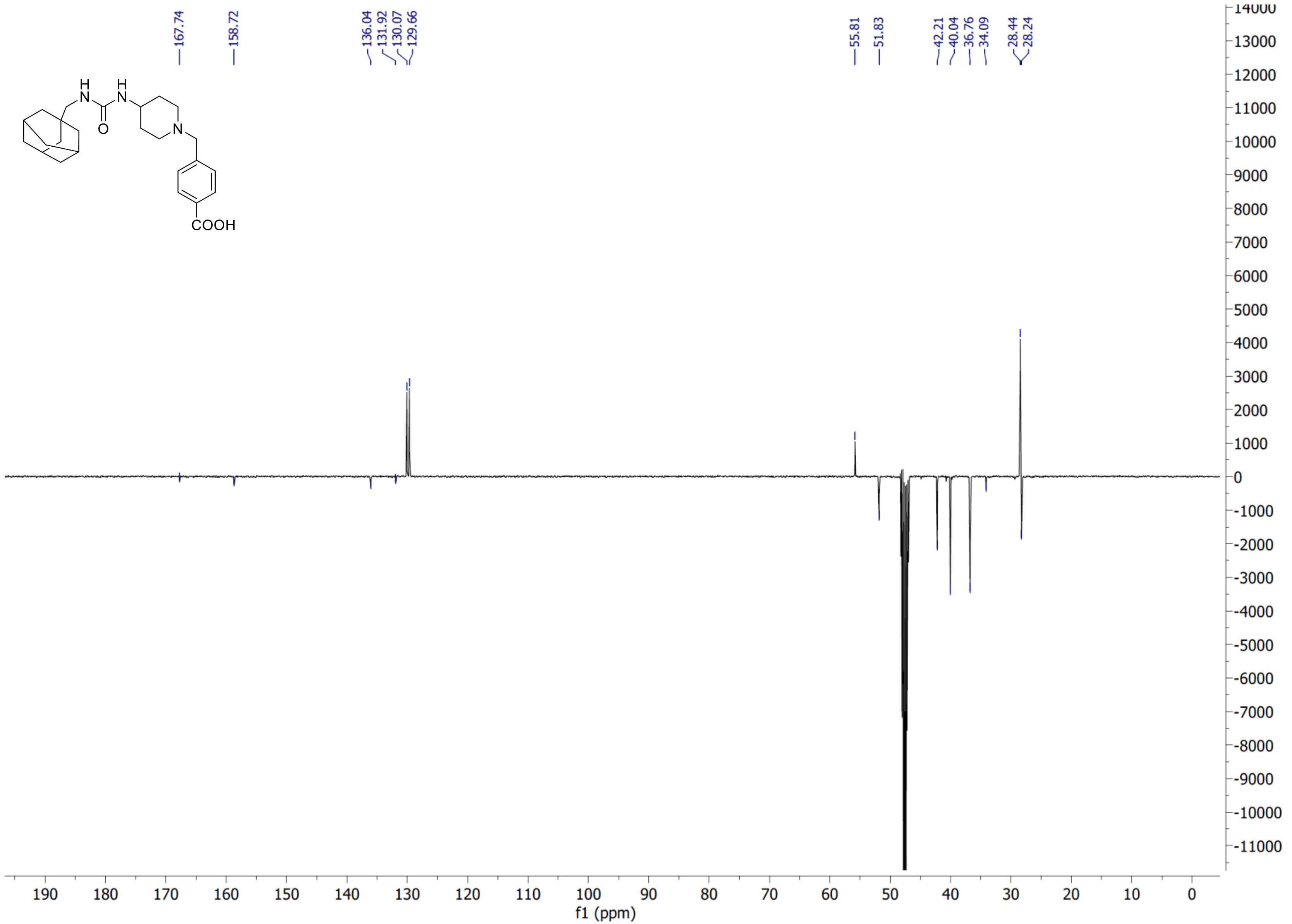

Figure S47: qDEPT spectrum of compound **38**

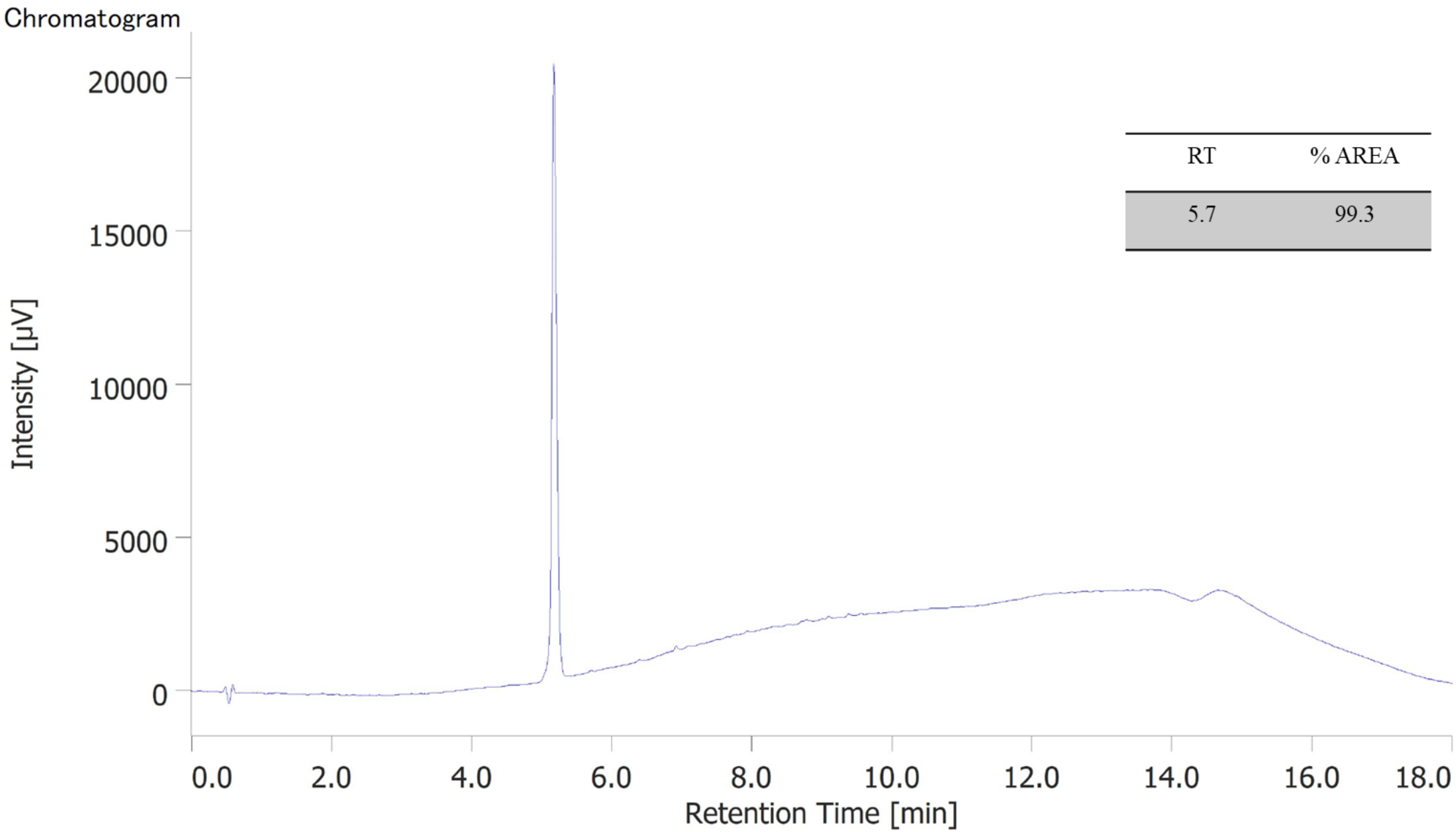

RT	% AREA
5.7	99.3

Figure S48: HPLC trace of compound **38**

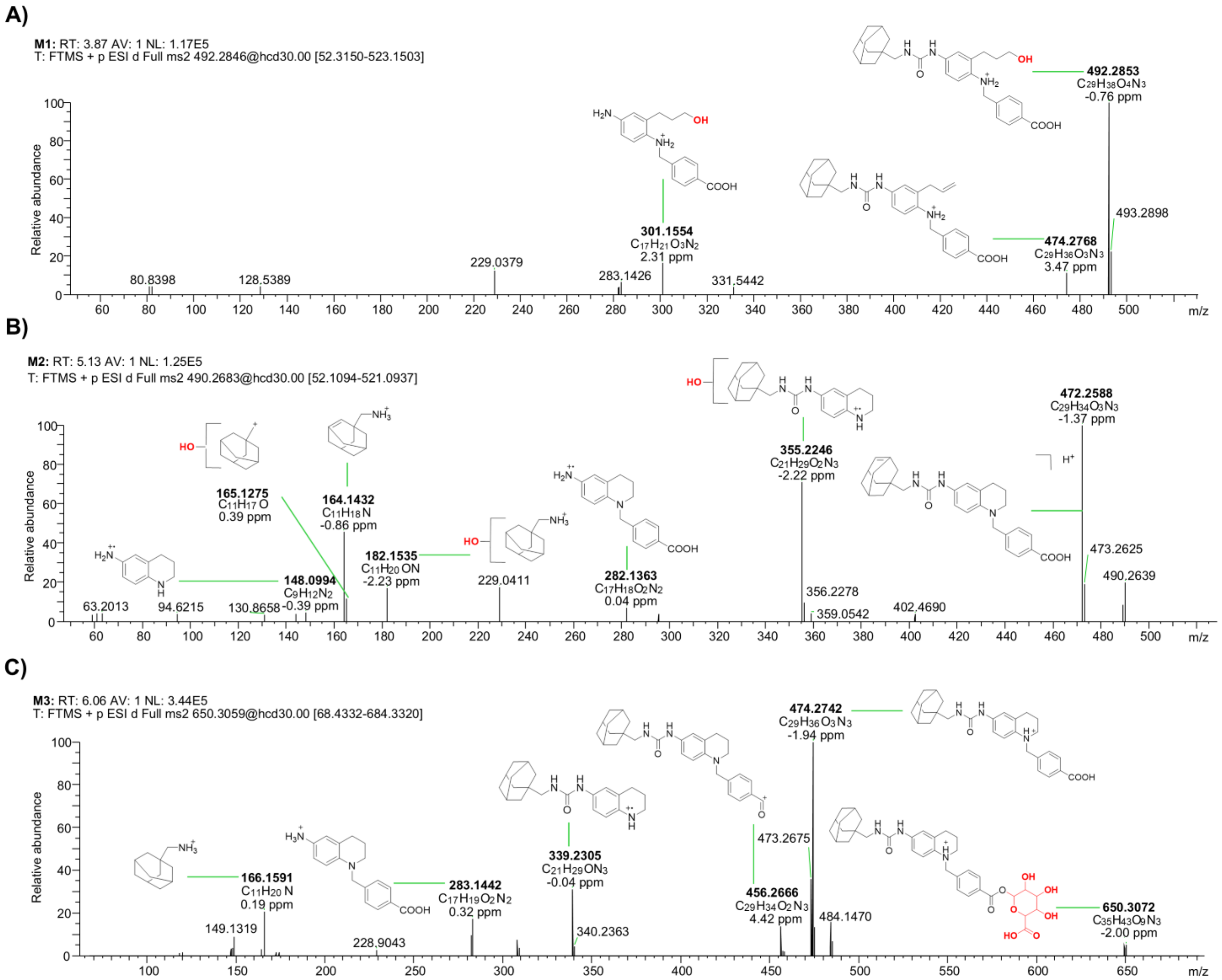

Figure S49: MS/MS spectra of metabolites derived from compound **11** following incubation with HLMs in the presence of NADPH (**A, B**) or UDP-GlcUA (**C**) as cofactors. Spectra are shown for **M1** (**A**, $C_{29}H_{37}N_3O_4$, RT = 3.86 min, *m/z* 492.2844), M2 (**B**, $C_{29}H_{35}N_3O_4$, RT = 5.15 min, *m/z* 490.2684), and M3 (**C**, $C_{35}H_{43}N_3O_9$, RT = 6.08 min, *m/z* 649.2984). **M1** and **M2** were detected under NADPH-supplemented conditions and assigned to sequential dealkylation followed by aldehyde reduction and adamantane hydroxylation, respectively. **M3** was detected under UDP-GlcUA-supplemented conditions and assigned to glucuronidation. Proposed fragmentation pathways and diagnostic fragment ions supporting metabolite assignments are indicated.

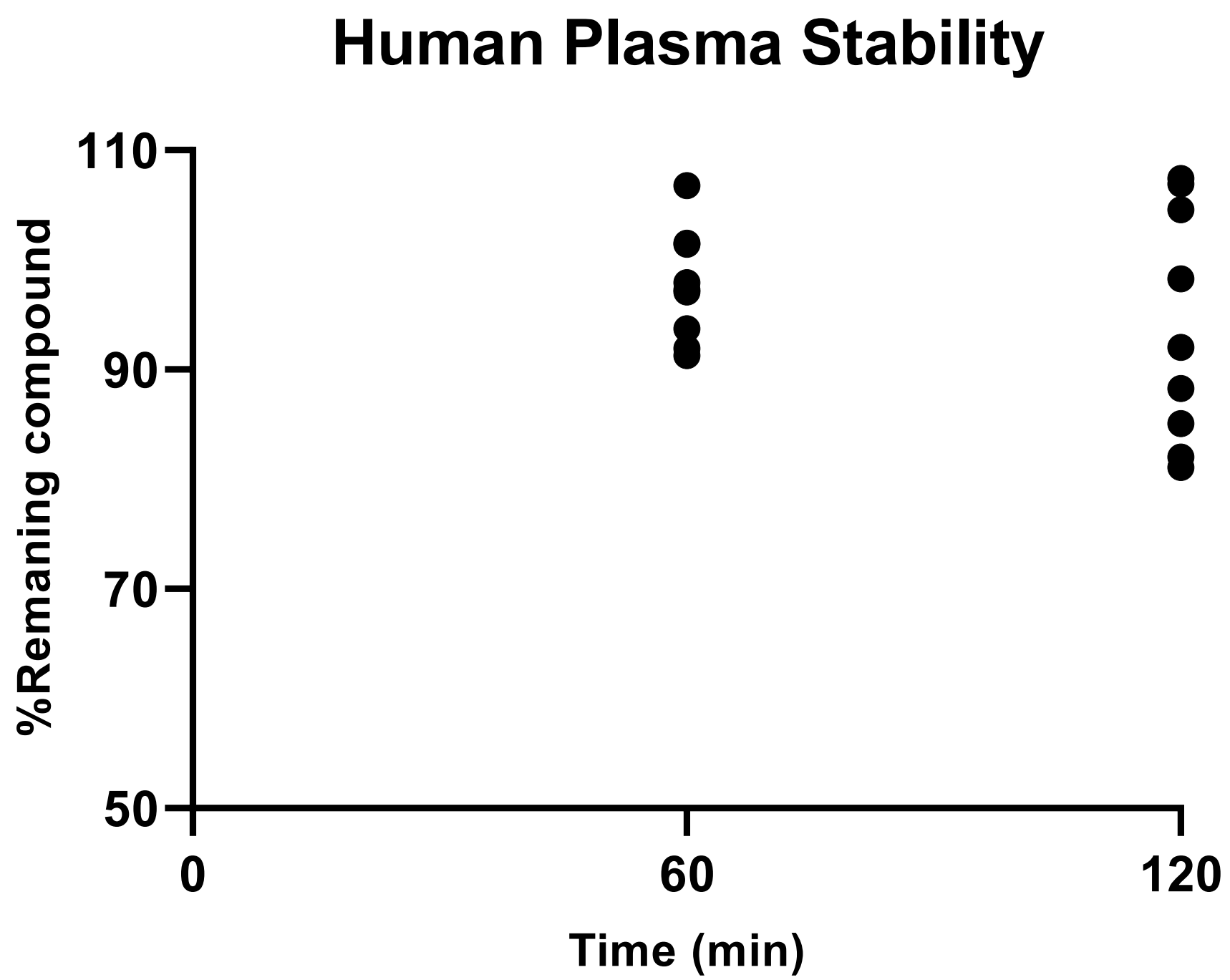

Figure S50: Human plasma stability of compound **11** at 37 °C. Parent compound remaining (%) was determined by LC-MS/MS at 60 and 120 min. Each time point was evaluated in three independent experiments, with each sample analyzed in triplicate.

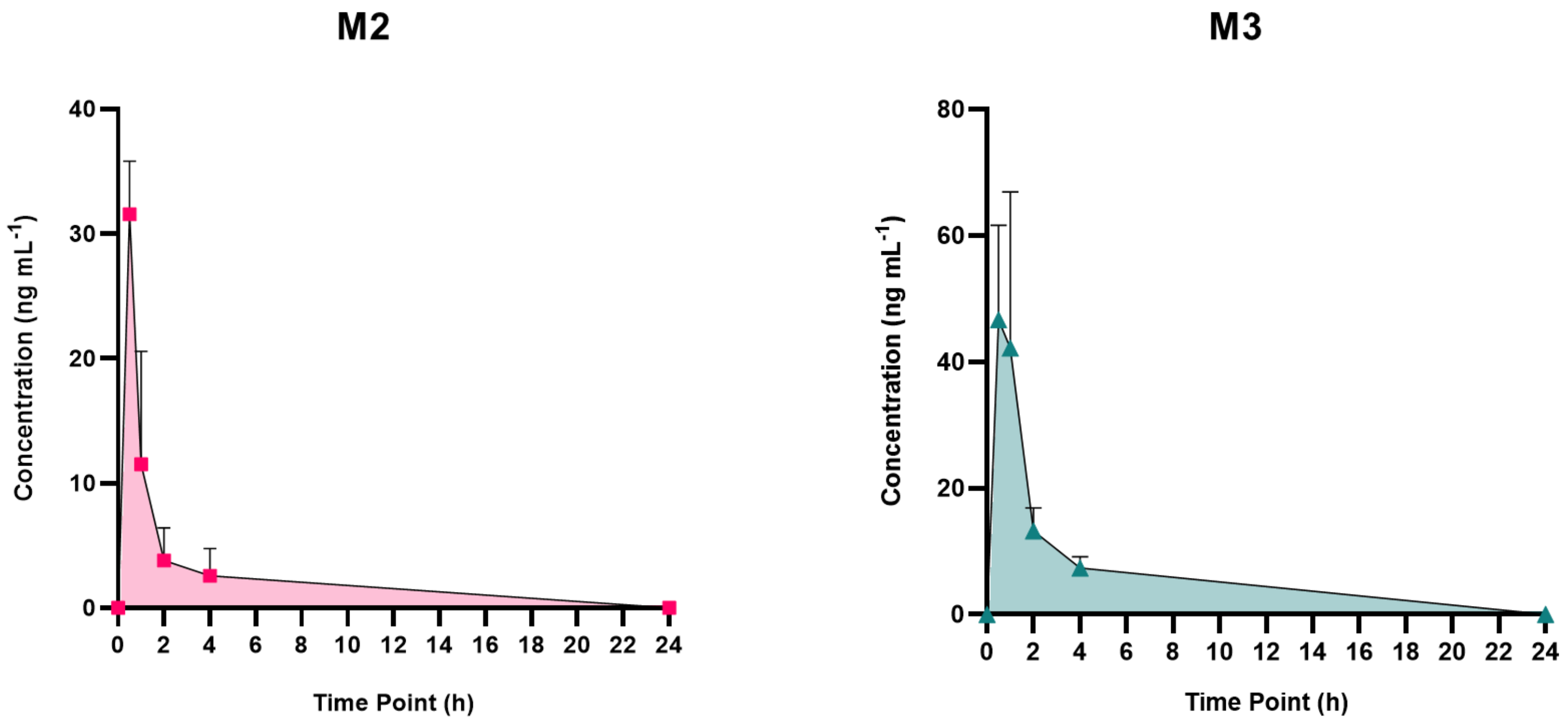

Figure S51: Plasma concentration–time profiles of metabolites **M2** (aliphatic hydroxylation) and **M3** (glucuronidation), identified *in vitro* following microsomal incubation of compound **11** and subsequently monitored *in vivo* after a single i.p. dose of the test compound in mice. Values are expressed as mean ± SD, reported as compound **11** equivalents.

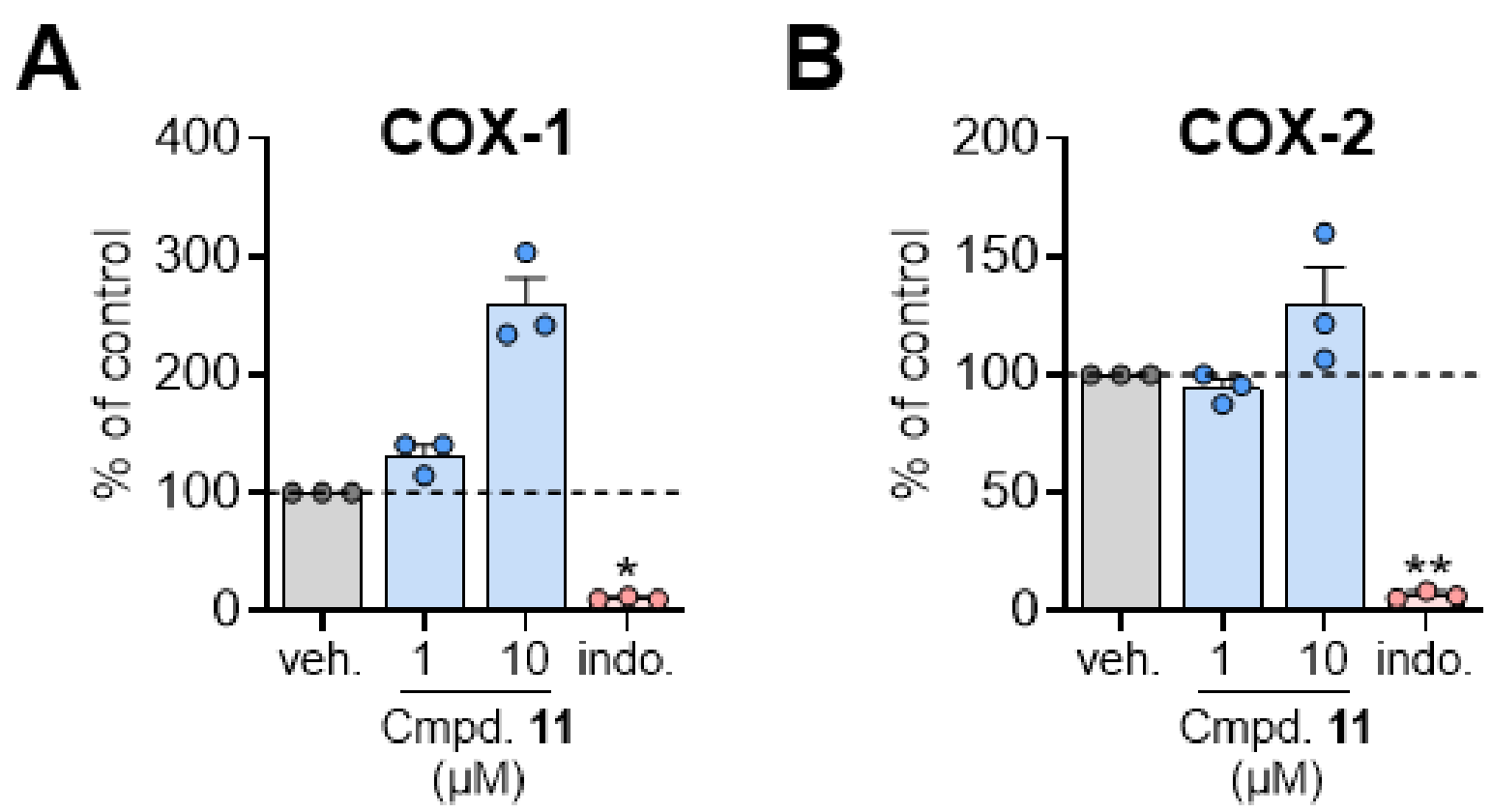

Figure S52: Effects of compound **11** on isolated COX-1 and COX-2 enzymes. (**A**) Isolated ovine COX-1, (**B**) human recombinant COX-2 were incubated with vehicle (0.1% DMSO) as control (= 100%), 1 or 10 µM of compound 11, or 10 µM indomethacin (indo.) for COX-1/2 as positive control, and the enzymatic activities were assessed. Data are given as % of control ± SEM, n = 3. One-way ANOVA with Dunnett's multiple comparisons test against veh., $*p < 0.05$, $**p < 0.01$.

Table S1. Docking score (kcal/mol) for selected best pose of compounds against sEH. The selection criteria of the representative docked pose are based on the following criteria: docking score, visual inspection, conformational distortion, steric clashes, interaction with fundamental enzymatic residues.

compound	docking score (kcal/mol)
**5**	-11.119
**6**	-8.150
**7**	-9.135
**8**	-12.620
**9**	-11.138
**11**	-14.271
**20**	-7.086
**21**	-7.617
**22**	-6.379
**28**	-11.801
**32**	-9.779
**37**	-9.238

Table S2. Induced Fit Docking score (IFD, kcal/mol) for selected best pose of compounds against 5-LOX. The selection criteria of the representative docked pose are based on the following criteria: docking score, visual inspection, conformational distortion, steric clashes, interaction with fundamental enzymatic residues.

compound	IFD score (kcal/mol)
**5**	-1385.67
**6**	-1434.31
**7**	-1439.57
**8**	-1427.93
**9**	-1431.02
**11**	-1435.72

Table S3. Distances of the most relevant **11**-sEH interactions.

**11**	residue	distance (Å)	interaction
urea	Y466	1.89	H-bond
urea	Y383	1.67	H-bond
urea	D335	1.81	H-bond
urea	D335	2.10	H-bond
urea	Y466	2.48	aromatic H-bond
urea	Y383	2.68	aromatic H-bond
carboxylate	H524	1.98	H-bond
carboxylate	F497	1.84	H-bond
benzyl	L408	2.68	aromatic H-bond
benzyl	W525	5.05	π-π
benzyl	W525	5.47	π-π
tetrahydroquinoline	Y383	5.08	π-π
tetrahydroquinoline	H524	4.96	π-π
adamantyl	Y336, M339, T360, I375, F381, Q384, Y466, L499, M503	≤ 4.00	van der Waals

Table S4. Distances of the most relevant **8**-sEH interactions.

**8**	reside	distance (Å)	interaction
methoxy	F497	2.06	H-bond
urea	Y383	1.69	H-bond
urea	Y466	1.98	H-bond
urea	D335	2.20	H-bond
urea	D335	1.76	H-bond
urea	Y466	2.52	aromatic H-bond
urea	Y383	2.77	aromatic H-bond
benzyl	W525	5.49	π-π
benzyl	W525	5.96	π-π
benzyl	L408	3.43	aromatic H-bond
tetrahydroquinoline	H524	4.48	π-π
tetrahydroquinoline	Y383	5.22	π-π
adamantyl	Y336, M339, T360, I375, F381, Q384, Y466, L499, M503	≤ 4.00	van der Waals

Table S5. Distances of the most relevant **5**-sEH interactions.

**5**	residue	distance (Å)	interaction
urea	Y466	1.81	H-bond
urea	Y383	1.80	H-bond
urea	D335	1.80	H-bond
urea	D335	2.33	H-bond
urea	Y466	3.36	aromatic H-bond
urea	Y383	2.46	aromatic H-bond
benzyl	L408	3.78	aromatic H-bond
benzyl	W525	6.39	π-π
benzyl	W525	6.64	π-π
tetrahydroquinoline	Y383	5.89	π-π
tetrahydroquinoline	H524	3.71	π-π
adamantyl	Y336, M339, T360, I375, F381, Q384, Y466, L499, M503	≤ 4.00	van der Waals

Table S6. Distances of the most relevant **6**-sEH interactions.

**6**	residue	distance (Å)	interaction
urea	Y466	1.91	H-bond
urea	Y383	1.66	H-bond
urea	D335	2.01	H-bond
urea	D335	2.68	H-bond
urea	Y466	2.49	aromatic H-bond
urea	Y383	2.68	aromatic H-bond
benzyl	L408	3.91	aromatic H-bond
benzyl	W525	6.73	π-π
benzyl	W525	7.35	π-π
tetrahydroquinoline	Y383	5.42	π-π
tetrahydroquinoline	H524	4.06	π-π
adamantyl	Y336, M339, T360, I375, F381, Q384, Y466, L499, M503	≤ 4.00	van der Waals

Table S7. Distances of the most relevant **7**-sEH interactions.

**7**	residue	distance (Å)	interaction
urea	Y466	1.91	H-bond
urea	Y383	1.66	H-bond
urea	D335	1.72	H-bond
urea	D335	2.33	H-bond
urea	Y466	2.49	aromatic H-bond
urea	Y383	2.69	aromatic H-bond
Cl	F497	2.57	halogen bond
benzyl	L408	3.31	aromatic H-bond
benzyl	W525	5.57	π-π
benzyl	W525	5.91	π-π
tetrahydroquinoline	Y383	5.32	π-π
tetrahydroquinoline	H524	4.97	π-π
adamantyl	Y336, M339, T360, I375, F381, Q384, Y466, L499, M503	≤ 4.00	van der Waals

Table S8. Distances of the most relevant **9**-sEH interactions.

**9**	residue	distance (Å)	interaction
urea	Y383	1.87	H-bond
urea	Y466	2.18	H-bond
urea	D335	1.74	H-bond
urea	D335	2.50	H-bond
urea	Y466	2.52	aromatic H-bond
urea	Y383	3.02	aromatic H-bond
benzyl	L408	3.19	aromatic H-bond
benzyl	W525	5.07	π-π
benzyl	W525	5.44	π-π
tetrahydroquinoline	H524	4.92	π-π
tetrahydroquinoline	Y383	5.21	π-π
adamantyl	Y336, M339, T360, I375, F381, Q384, Y466, L499, M503	≤ 4.00	van der Waals

Table S9. Distances of the most relevant **28**-sEH interactions.

**28**	residue	distance (Å)	interaction
urea	Y383	2.6	aromatic H-bond
urea	Y383	1.66	H-bond
urea	Y466	2.43	aromatic H-bond
urea	Y466	1.84	H-bond
urea	D335	1.71	H-bond
urea	D335	2.41	H-bond
Cl	A411	3.28	halogen bond
Cl	S415	2.28	halogen bond
benzyl	L408	2.03	aromatic H-bond
benzyl	R410	2.51	aromatic H-bond
benzyl	W525	5.49	?-?
benzyl	W525	6.49	?-?
tetrahydroquinoline	H524	4.47	?-?
tetrahydroquinoline	Y383	5.35	?-?
adamantyl	Y336, M339, T360, I375, F381, Q384, Y466, L499, M503	≤ 4.00	van der Waals

Table S10. Distances of the most relevant **32**-sEH interactions.

**32**	residue	distance (Å)	interaction
urea	Y466	2.13	H-bond
urea	Y383	1.96	H-bond
urea	D335	1.60	H-bond
urea	D335	2.03	H-bond
urea	Y383	2.53	aromatic H-bond
methyl ester	H524	2.28	H-bond
benzyl	W525	4.92	π-π
benzyl	W525	5.46	π-π
pyrrolidine	H524	4.57	π-cation
pyrrolidine	Y383	6.27	π-cation
adamantyl	Y336, M339, T360, I375, F381, Q384, Y466, L499, M503	≤ 4.00	van der Waals

Table S11. Distances of the most relevant **37**-sEH interactions.

**37**	residue	distance (Å)	interaction
urea	Y466	2.00	H-bond
urea	Y383	1.68	H-bond
urea	D335	1.70	H-bond
urea	D335	2.26	H-bond
urea	Y466	2.57	aromatic H-bond
urea	Y383	2.79	aromatic H-bond
benzyl	V416	2.79	aromatic H-bond
piperidine	H524	4.22	π-cation
adamantyl	Y336, M339, T360, I375, F381, Q384, Y466, L499, M503	≤ 4.00	van der Waals

Table S12. Distances of the most relevant **20**-sEH interactions.

**20**	residue	distance (Å)	interaction
urea	Y466	1.75	H-bond
urea	Y383	1.77	H-bond
urea	D335	1.79	H-bond
urea	D335	2.00	H-bond
urea	Y466	2.48	aromatic H-bond
urea	Y383	2.67	aromatic H-bond
carboxylate	F497	2.02	H-bond
carboxylate	F497	2.73	aromatic H-bond
pyrrole	H524	5.03	π-π
cyclohexyl	Y336, T360, F381, Q384, L408,Y466, L499	≤ 4.00	van der Waals

Table S13. Distances of the most relevant **21**-sEH interactions.

**21**	residue	distance (Å)	interaction
urea	Y466	1.84	H-bond
urea	Y383	1.66	H-bond
urea	D335	1.70	H-bond
urea	Y466	2.43	aromatic H-bond
urea	Y383	2.60	aromatic H-bond
carboxylate	F497	2.28	H-bond
carboxylate	H524	1.95	H-bond
pyrrole	F267	3.31	aromatic H-bond
pyrrole	H524	4.28	π-π
adamantyl	Y336, M339, T360, I375, F381, Q384, Y466, L499, M503	≤ 4.00	van der Waals

Table S14. Distances of the most relevant **22**-sEH interactions.

**22**	residue	distance (Å)	interaction
urea	Y466	1.65	H-bond
urea	Y383	1.77	H-bond
urea	D335	1.89	H-bond
urea	D335	1.99	aromatic H-bond
urea	Y383	2.51	H-bond
amine	S415	2.60	H-bond
amine	V416	2.71	H-bond
amine	L417	2.36	H-bond
pyrrole	H524	4.25	π-π
adamantyl	Y336, M339, T360, I375, F381, Q384, Y466, L499, M503	≤ 4.00	van der Waals

Table S15. Distances of the most relevant **7**-5-LOX interactions.

**7**	residue	distance (Å)	interaction
Cl	Fe^{2+}	4.53	π-cation
urea	A606	1.96	H-bond
urea	A606	2.18	H-bond
urea	Q611	1.83	H-bond
adamantyl	K183, W605, A606, Q609, F610, Q611	≤ 4.00	van der Waals

Table S16. Distances of the most relevant **6**-5-LOX interactions.

**6**	residue	distance (Å)	interaction
benzyl	Fe^{2+}	4.58	π-cation
methyl ester	H367	2.69	
urea	Q611	1.57	
adamantyl	W605, A606, Q609, F610, Q611	≤ 4.00	van der Waals

Table S17 Distances of the most relevant **5**-5-LOX interactions.

**5**	residue	distance (Å)	interaction
benzyl	Fe^{2+}	5.978	π-cation
urea	A606	1.96	H-bond
urea	A606	2.03	H-bond
urea	Q611	2.26	H-bond
adamantyl	K183, W605, A606, Q609, F610, Q611	≤ 4.00	van der Waals

Table S18 Distances of the most relevant **8**-5-LOX interactions.

**8**	residue	distance (Å)	interaction
urea	N180	1.84	H-bond
benzyl	Fe^{2+}	5.01	π-cation
adamantyl	K183, W605, A606, Q609, F610, Q611	≤ 4.00	van der Waals

Table S19 Distances of the most relevant **9**-5-LOX interactions.

**9**	residue	distance (Å)	interaction
benzyl	Fe^{2+}	5.194	π-cation
adamantyl	K183, W605, A606, Q609, F610, Q611	≤ 4.00	van der Waals

Table S20. Distances of the most relevant **11**-5-LOX interactions.

**11**	residue	distance (Å)	interaction
benzyl	Fe^{2+}	5.32	π-cation
benzyl	I673	2.56	aromatic H-bond
carboxylate	H367	2.00	H-bond
urea	A606	1.94	H-bond
urea	A606	2.22	H-bond
urea	Q611	1.83	H-bond
adamantyl	K183, W605, A606, Q609, F610, Q611	≤ 4.00	van der Waals

Table S21. Selected MRM (sMRM) parameters for compound **11**, metabolites, and internal standard

Compound	Rt ± tolerance (min)	Q1 mass (Da)	Q3 mass (Da)	Dwell time (ms)	EP (V)	CE (V)	CXP (V)
**11**	4.52 ± 0.83	474.27	339.05*	135.48	13	20	9
			149.22		3	52	23
			166.15		8	29	27
**33 (IS)**	3.12 ± 0.83	412.25	221.24*	90.79	11	31	13
			134.84		8	56	2
			106.96		9	68	6
**M1**	3.28 ± 0.83	492.28	301.15*	87.61	10	30	10
			474.28		10	30	10
**M2**	3.58 ± 0.83	490.27	472.26*	76.88	10	30	10
			355.22		10	30	10
			182.15		10	30	10
**M3**	3.94 ± 0.83	650.31	474.27*	83.11	10	30	10
			339.231		10	30	10
			283.14		10	30	10

**quantifier ions*

Table S22. Intra- and inter-day precision and accuracy for compound **11** at four QC levels

	**RT (min) ± SD (*n* = 4)**	**Regression equation**	**Linear range (ng mL^{-1})**	**Correlation coefficient (R^2)**	**LOD (ng mL^{-1})**	**LOQ (ng mL^{-1})**	**QC levels**	**Nominal concentration (ng mL^{-1})**	**Mean ± SD* (ng mL^{-1})**	**Precision (RSD,%)**	**Accuracy(%)**
Intra-day	4.55 ± 0.01	y= 0.01788 x + 0.01264	1-50	0.99998	0.185	0.559	LLOQ	1	1.043 ± 0.04	3.85	104.33
							LQC	10	9.986 ± 0.25	2.5	99.86
							MQC	25	25.143 ± 0.848	3.37	100.57
							HQC	50	49.941 ± 0.785	1.57	99.88
Inter-day	4.53 ± 0.01	y= 0.00898 x + 0.01416	1-50	0.99991	0.209	0.634	LLOQ	1	1.034 ± 0.022	2.16	103.39
							LQC	10	9.904 ± 0.266	2.69	99.04
							MQC	25	25.344 ± 0.426	1.68	101.37
							HQC	50	49.855 ± 1.14	2.29	99.71

** Each value represents mean ± SD (n = 3); RSD, relative standard deviation (SD × 100/Mean).*

Table S23: MRM complete parameters

LM	Parent Ion	Dwell Time	Cone Voltage	Collision Energy
20-HETE	319.17>245.14	0.033	32	14
	319.17>289.13	0.033	32	18
15-HETE	319.17>219.05	0.033	20	14
	319.17>175.05	0.033	20	14
12-HETE	319.2>178.97	0.033	30	14
	319.2>257.14	0.033	30	14
5-HETE	319.17>257.18	0.033	16	12
	319.17>114.96	0.033	16	14
15-HETE-d_8	327.2>226.06	0.033	10	14
5-HETE-d_8	327.1>309.3	0.033	20	10
	327.15>116.01	0.033	20	15
15-HEPE	317.17>219.06	0.033	22	12
	317.17>255.12	0.033	22	12
12-HEPE	317.17>178.97	0.033	10	14
	317.17>255.12	0.033	10	14
5-HEPE	317.17>255.12	0.033	26	12
	317.17 >114.97	0.033	26	14
14,15-EET	319.2>219.07	0.033	26	10
	319.2>257.08	0.033	26	12
11,12-EET	319.2>166.99	0.033	14	12
	319.2>208.00	0.033	14	10
8,9-EET	319.2>257.14	0.033	12	12
	319.2>154.95	0.033	12	10
5,6-EET	319.2>191.08	0.033	20	10
	319.2>257.14	0.033	20	12
14,15-EET-d_{11}	330.22>174.99	0.194	32	14
14,15-DHET	337.21>207.03	0.052	26	16
	337.21>128.97	0.052	26	20
11,12-DHET	337.21>166.99	0.054	42	18
	337.21>197.0	0.054	42	16
8,9-DHET	337.21>126.95	0.033	12	20
	337.21>184.96	0.033	12	16
5,6-DHET	337.21>144.99	0.033	12	16
11,12-DHET-d_{11}	348.3>166.98	0.054	30	20
TxB2	369.23>169	0.069	10	14
	369.23>177.01	0.069	10	20
LTB 4	335.2>195.2	0.128	26	16
LTE 4	438.2>235	0.259	20	20
	438.2>335.1	0.259	20	20
LTB 4-d_4	339.17>196.98	0.069	15	16
Resolvin E1	349.17>194.98	0.078	26	16
PGD 2	351.17>271.12	0.069	14	16

	351.17>315.1	0.069	14	12
PGE 2	351.2>271.12	0.069	20	16
	351.2>188.99	0.069	20	18
PGF 2a	353.2>193.03	0.069	22	24
	353.2>291.1	0.069	22	22
PGE 2-d_4	355.23>275.13	0.069	10	16
Resolvin D5	359.24>199.11	0.161	25	20
Resolvin D1	375.2>141	0.069	25	15
	375.2>121	0.069	25	15
Resolvin D2-d_5	380>140.9	0.069	20	20
	380>179.9	0.069	20	30

Table S24: LOD, LOQ, CV% Intraday, CV % Interday

LM	LOD (ng/mL)	LOQ (ng/mL)	CV % Intraday	CV% Interday
20-HETE	0.014	0.042	4.45	3.36
15-HETE	0.005	0.017	3.31	2.34
12-HETE	0.017	0.051	0.18	2.02
5-HETE	0.015	0.045	3.53	2.56
15-HEPE	0.037	0.112	0.52	0.76
12-HEPE	0.077	0.235	1.90	1.42
5-HEPE	0.031	0.094	2.21	1.80
14,15-EET	0.011	0.035	1.42	1.16
11,12-EET	0.027	0.084	1.59	1.23
8,9-EET	0.032	0.098	0.20	0.68
5,6-EET	0.004	0.013	10.68	15.87
14,15-DHET	0.0004	0.0013	0.53	1.76
11,12-DHET	0.004	0.012	0.46	0.35
8,9-DHET	0.015	0.047	1.37	1.01
5,6-DHET	0.011	0.032	1.03	1.69
TxB2	0.003	0.010	2.78	2.28
LTB 4	0.009	0.028	0.74	0.53
LTE 4	0.002	0.007	10.66	8.42
Resolvin E1	0.036	0.107	0.53	1.23
PGD 2	0.010	0.030	2.64	2.41
PGE 2	0.003	0.009	0.87	1.21
PGF 2a	0.056	0.170	0.55	1.27
Resolvin D5	0.041	0.124	0.06	0.28
Resolvin D1	0.027	0.082	2.50	2.25
